# Supplementary material for: Mapping multimorbidity in individuals with schizophrenia and bipolar disorders: evidence from the South London and Maudsley NHS Foundation Trust Biomedical Research Centre (SLAM BRC) case register
Source: BMJ Open. 2022 Jan 24;12(1):e054414. doi: 10.1136/bmjopen-2021-054414 (PMC8788233; doi:10.1136/bmjopen-2021-054414)
Supplement: Supplementary data [file bmjopen-2021-054414supp001.pdf]

Supplemental Material

Supplemental Tables

Supplemental Table 1. Meta-annotations performance results for Diagnosis and Status

Supplemental Table 2. Prevalence estimates and sex differences within the whole SMI cohort and SSD and BD subgroups.

Supplemental Table 3. Prevalence for each condition by age ranges at first SMI diagnosis for the whole SMI cohort and SSD and BD subgroups.

Supplemental Tables 4a, 4b and 4c. Prevalence for each condition across ethnicities for the whole SMI cohort and SSD and BD subgroups.

Supplemental Table 5. Social deprivation prevalence for each condition for the whole cohort and within SSD and BD.

Supplemental Table 6. Sociodemographic features of cohort in HoNOS subsample analysis

Supplemental Table 7. Associations between comorbidities and HoNOS scores in SSD.

Supplemental Table 8. Associations between comorbidities and HoNOS scores in BD.

Supplemental Figures

Supplemental Figure 1. Distribution of all conditions in the SMI cohort by SMI group.

Supplemental Figure 2. Prevalence rates for age at SMI diagnoses per condition and comparison between individuals with BD and SSD.

Appendix 1. SNOMED Container and Concept Level Groupings for physical health conditions included in this study.

Supplemental Tables

Supplemental Table 1. Meta-annotations performance results for Diagnosis and Status

**Meta-Annotation**	**Values**	**F1 (macro/weighted)**	**P (macro/weighted)**	**R (macro/weighted)**
Diagnosis	Patient / Other	0.94 / 0.94	0.95 / 0.95	0.92 / 0.94
Status	Affirmed / Other	0.89 / 0.98	0.94 / 0.98	0.85 / 0.98

Supplemental Table 2. Prevalence estimates and sex differences within the whole SMI cohort and SSD and BD subgroups.

	**Total Cohort**		**SSD**			**BD**		
	Female	Male	Female	Male	p-value	Female	Male	p- value
**Diabetes**	1282 (15.8)	1403 (15)	1021 (18.8)	1187 (15.6)	<.001	261 (9.7)	216 (12.1)	.190
**Hypertension *****	1325 (16.3)	1212 (12.9)	1060 (19.6)	1010 (13.3)	<.001	265 (9.8)	202 (11.4)	1.000
**Asthma ****	869 (10.7)	852 (9.1)	559 (10.3)	732 (9.6)	1.000	310 (11.5)	120 (6.7)	<.001
**Arthritis *****	657 (8.1)	297 (3.2)	471 (8.7)	231 (3.0)	<.001	186 (6.9)	66 (3.7)	<.001
**Epilepsy**	349 (4.3)	450 (4.8)	253 (4.7)	399 (5.3)	1.000	96 (3.6)	51 (2.9)	1.000
**Cerebrovascular diseases**	367 (4.5)	361 (3.9)	265 (4.9)	308 (4.1)	.499	102 (3.8)	53 (3.0)	1.000
**Eczema ***	331 (4.1)	285 (3.0)	238 (4.4)	241 (3.2)	<.01	93 (3.4)	44 (2.5)	1.000
**Migraine *****	369 (4.5)	194 (2.1)	217 (4.0)	155 (2.0)	<.001	152 (5.6)	39 (2.2)	<.001
**Ischaemic heart disease**	249 (3.1)	312 (3.3)	196 (3.6)	239 (3.1)	1.000	53 (2.0)	73 (4.2)	<.001
**Chronic Obstructive Lung diseases**	237 (2.9)	239 (2.5)	153 (2.8)	189 (2.5)	1.000	84 (3.1)	50 (2.8)	1.000
**Chronic Kidney disease**	158 (1.9)	121 (1.3)	104 (1.9)	75 (1.0)	<.001	54 (2.0)	46 (2.6)	1.000
**Parkinson disease**	113 (1.4)	153 (1.6)	81 (1.5)	120 (1.6)	1.000	32 (1.2)	33 (1.9)	1.000
**Heart failure**	123 (1.5)	99 (1.0)	105 (1.9)	82 (1.1)	.001	18 (0.7)	17 (1.0)	1.000
**Psoriasis**	76 (0.9)	103 (1.1)	45 (0.8)	84 (1.1)	1.000	31 (1.1)	19 (1.1)	1.000
**Atrial fibrillation**	65 (0.8)	68 (0.7)	50 (0.9)	50 (0.7)	1.000	15 (0.6)	18 (1.0)	1.000
**Transient Ischaemic Attack**	79 (1.0)	51 (0.5)	53 (1.0)	38 (0.5)	.037	26 (1.0)	13 (0.7)	1.000
**Inflammatory Bowel diseases**	26 (0.3)	14 (0.1)	18 (0.3)	7 (0.1)	.080	8 (0.3)	7 (0.4)	1.000

**Multiple Sclerosis**	17 (0.2)	15 (0.2)	9 (0.2)	10 (0.1)	-	8 (0.3)	5 (0.3)	-
**Chronic liver disease**	10 (0.1)	12 (0.1)	9	11 (0.1)	-	1 (0.0)	1 (0.1)	-
**Chronic Sinusitis**	3 (0.0)	3 (0.0)	3	3 (0.0)	-	0 (0.0)	0 (0.0)	-

Note. *** p < .001; ** p < .01; * p < .05 for comparisons between male and female groups in the whole SMI cohort

Supplemental Table 3. Prevalence for each condition for the whole cohort and diagnoses subgroups and across age ranges at first SMI diagnosis.

BMJ Publishing Group Limited (BMJ) disclaims all liability and responsibility arising from any reliance placed on this supplemental material which has been supplied by the author(s)

	**Total**					**SSD**						**BD**					
	**15-34**	**35-44**	**45-54**	**55-64**	**65+**	**15-34**	**35-44**	**45-54**	**55-64**	**65+**		**15-34**	**35-44**	**45-54**	**55-64**	**65+**	
**Total**	7497	3736	2783	1525	1959	5607	2792	2057	1067	1496		1890	944	726	458	463	
**Diabetes*** **	697 (9.3)	559 (15.0)	531 (19.1)	371 (24.3)	528 (27.0)	591 (10.5)	479 (17.2)	439 (21.3)	284 (26.6)	415 (27.7)	p < 0.001	106 (5.6)	80 (8.5)	92 (12.7)	87 (19.0)	113 (24.4)	p < 0.001
**Hypertension*** **	472 (6.3)	426 (11.4)	514 (18.5)	416 (27.)3	709 (36.2)	412 (7.3)	372 (13.3)	411 (20.0)	313 (29.3)	562 (37.6)	p < 0.001	60 (3.2)	54 (5.7)	103 (14.2)	103 (22.5)	147 (31.7)	p < 0.001
**Asthma*** **	820 (10.9)	347 (9.3)	297 (10.7)	126 (8.3)	132 (6.7)	635 (11.3)	248 (8.9)	218 (10.6)	89 (8.3)	101 (6.8)	p < 0.001	185 (9.8)	99 (10.5)	79 (10.9)	37 (8.1)	31 (6.7)	p = 1.000
**Arthritis*** **	125 (1.7)	154 (4.1)	229 (8.2)	167 (11.0)	279 (14.2)	93 (1.7)	120 (4.3)	159 (7.7)	118 (11.1)	212 (14.2)	p < 0.001	32 (1.7)	34 (3.6)	70 (9.6)	49 (10.7)	67 (14.5)	p < 0.001
**Epilepsy**	339 (4.5)	188 (5.0)	133 (4.8)	67 (4.4)	72 (3.7)	276 (4.9)	154 (5.5)	112 (5.4)	55 (5.2)	55 (3.7)	1.000	63 (3.3)	34 (3.6)	21 (2.9)	12 (2.6)	17 (3.7)	1.000
**CVA*** **	166 (2.2)	112 (3.0)	122 (4.4)	114 (7.5)	214 (10.9)	143 (2.6)	87 (3.1)	95 (4.6)	84 (7.9)	164 (11.0)	p < 0.001	23 (1.2)	25 (2.6)	27 (3.7)	30 (6.6)	50 (10.8)	p < 0.001
**Eczema**	310 (4.1)	115 (3.1)	79 (2.8)	50 (3.3)	62 (3.2)	244 (4.4)	88 (3.2)	58 (2.8)	37 (3.5)	52 (3.5)	0.120	66 (3.5)	27 (2.9)	21 (2.9)	13 (2.8)	10 (2.2)	1.000
**Migraine*** **	300 (4.0)	122 (3.3)	87 (3.1)	32 (2.1)	23 (1.2)	207 (3.7)	73 (2.6)	53 (2.6)	24 (2.2)	15 (1.0)	p < 0.001	93 (4.9)	49 (5.2)	34 (4.7)	8 (1.7)	8 (1.7)	0.011
**Isc heart disease*** **	111 (1.5)	73 (2.0)	105 (3.8)	95 (6.2)	177 (9.0)	96 (1.7)	60 (2.1)	80 (3.9)	65 (6.1)	134 (9.0)	p < 0.001	15 (0.8)	13 (1.4)	25 (3.4)	30 (6.6)	43 (9.3)	p < 0.001
**COPD*** **	39 (0.5)	58 (1.6)	119 (4.3)	115 (7.5)	145 (7.4)	29 (0.5)	40 (1.4)	87 (4.2)	82 (7.7)	104 (7.0)	p < 0.001	10 (0.5)	18 (1.9)	32 (4.4)	33 (7.2)	41 (8.9)	p < 0.001
**CKD*** **	12 (0.2)	18 (0.5)	41 (1.5)	55 (3.6)	153 (7.8)	9 (0.2)	14 (0.5)	28 (1.4)	33 (3.1)	95 (6.4)	p < 0.001	3 (0.2)	4 (0.4)	13 (1.8)	22 (4.8)	58 (12.5)	-
**PD*** **	70 (0.9)	33 (0.9)	37 (1.3)	41 (2.7)	85 (4.3)	57 (1.0)	22 (0.8)	27 (1.3)	32 (3.0)	63 (4.2)	p < 0.001	13 (0.7)	11 (1.2)	10 (1.4)	9 (2.0)	22 (4.8)	p < 0.001
**HF*** **	18 (0.2)	30 (0.8)	42 (1.5)	40 (2.6)	92 (4.7)	17 (0.3)	30 (1.1)	38 (1.8)	29 (2.7)	73 (4.9)	p < 0.001	1 (0.1)	0 (0.0)	4 (0.6)	11 (2.4)	19 (4.1)	-
**Psoriasis**	67 (0.9)	33 (0.9)	37 (1.3)	17 (1.1)	25 (1.3)	54 (1.0)	25 (0.9)	25 (1.2)	9 (0.8)	16 (1.1)	1.000	13 (0.7)	8 (0.8)	12 (1.7)	8 (1.7)	9 (1.9)	0.450
**Atrial fibrillation*** **	14 (0.2)	7 (0.2)	12 (0.4)	19 (1.2)	81 (4.1)	11 (0.2)	6 (0.2)	8 (0.4)	12 (0.1)	63 (4.2)	p < 0.001	3 (0.20	1 (0.1)	4 (0.6)	7 (1.5)	18 (3.9)	-
**TIA*** **	20 (0.3)	18 (0.5)	19 (0.7)	23 (1.5)	50 (2.6)	15 (0.3)	15 (0.5)	13 (0.6)	14 (1.3)	34 (2.3)	p < 0.001	5 (0.3)	3 (0.3)	6 (0.8)	9 (2.0)	16 (3.5)	-
**IBD**	9 (0.1)	8 (0.2)	12 (0.4)	6 (0.4)	5 (0.3)	4 (0.1)	6 (0.2)	7 (0.3)	3 (0.3)	5 (0.3)	-	5 (0.3)	2 (0.2)	5 (0.7)	3 (0.7)	0 (0.0)	-
**MS**	6 (0.1)	8 (0.2)	11 (0.4)	5 (0.3)	2 (0.1)	4 (0.1)	4 (0.1)	8 (0.4)	3 (0.3)	0 (0.0)	-	2 (0.1)	4 (0.4)	3 (0.4)	2 (0.4)	2 (0.4)	-

**Chronic liver disease**	5 (0.1)	7 (0.2)	6 (0.2)	3 (0.2)	1 (0.1)	5 (0.1)	7 (0.3)	5 (0.2)	2 (0.2)	1 (0.1)	-	0 (0.0)	0 (0.0)	1 (0.1)	1 (0.2)	0 (0.0)	-
**Chronic sinusitis**	5 (0.1)	0 (0.0)	1 (0.0)	0 (0.0)	0 (0.0)	5 (0.1)	0 (0.0)	1 (0.0)	0 (0.0)	0 (0.0)	-	0 (0.0)	0 (0.0)	0 (0.0)	0 (0.0)	0 (0.0)	-

Note. *** p < .001; ** p < .01; * p < .05 for comparisons between age groups within the SMI cohort.

Supplemental Tables 4. Prevalence for each condition for the whole cohort and diagnoses subgroups and across ethnicities

Table 4a. Prevalence for each condition for the whole cohort and ethnicity.

	**White British n=6243 (35.7%)**	**Irish n=346 (2.0%)**	**Black Caribbean n=3182 (18.2%)**	**Black African n=2094 (12.0%)**	**South Asian n=549 (3.1%)**	**Other[§] n=2846 (22.0%)**	**Unknown[§] n=1240 (7.1%)**	**Statistics**
**Diabetes**	837 (13.4)	55 (15.9)	757 (23.8)	382 (18.2)	125 (22.8)	461 (12.0)	69 (5.6)	χ^2 (4) = 172.49; *p* <0.001
**Hypertension**	804 (12.9)	63 (18.2)	678 (21.3)	430 (20.5)	102 (18.6)	400 (10.4)	60 (4.8)	χ^2 (4) = 137.98; *p* <0.001
**Asthma**	651 (10.4)	40 (11.6)	484 (15.2)	156 (7.4)	42 (7.7)	296 (7.7)	53 (4.3)	χ^2 (4) = 92.58; *p* <0.001
**Arthritis**	402 (6.4)	29 (8.4)	246 (7.7)	90 (4.3)	36 (6.6)	139 (3.6)	12 (1.0)	χ^2 (4) = 26.80; *p* <0.001
**Epilepsy**	339 (5.4)	26 (7.5)	164 (5.2)	77 (3.7)	17 (3.1)	147 (3.8)	29 (2.4)	χ^2 (4) = 19.02; *p* = 0.010
**Cerebrovascular accident**	274 (4.4)	20 (5.8)	184 (5.8)	92 (4.4)	25 (4.6)	116 (3.0)	17 (1.4)	χ^2 (4) = 10.60; *p* = 0.408
**Eczema**	222 (3.6)	7 (2.0)	185 (5.8)	61 (2.9)	17 (3.1)	108 (2.8)	16 (1.3)	χ^2 (4) = 41.93; *p* <0.001
**Migraine**	235 (3.8)	12 (3.5)	129 (4.1)	61 (2.9)	17 (3.1)	98 (2.5)	12 (1.0)	χ^2 (4) = 5.44; *p* >.99
**Ischemic heart disease**	261 (4.2)	13 (3.8)	108 (3.4)	48 (2.3)	19 (3.5)	99 (2.6)	13 (1.0)	χ^2 (4) = 16.74; *p* = 0.028
**COPD**	271 (4.3)	32 (9.2)	63 (2.0)	21 (1.0)	14 (2.6)	60 (1.6)	15 (1.2)	χ^2 (4) = 114.69; *p* <0.001
**CKD**	125 (2.0)	9 (2.6)	56 (1.8)	32 (1.5)	13 (2.4)	37 (1.0)	7 (0.6)	χ^2 (4) = 3.81; *p* >.99
**Parkinson's disease**	117 (1.9)	6 (1.7)	56 (1.8)	25 (1.2)	11 (2.0)	45 (1.2)	6 (0.5)	χ^2 (4) = 4.56; *p* >.99
**Heart failure**	88 (1.4)	8 (2.3)	55 (1.7)	29 (1.4)	8 (1.5)	31 (0.8)	3 (0.2)	χ^2 (4) = 3.16; *p* >.99
**Psoriasis**	103 (1.6)	4 (1.2)	12 (0.4)	6 (0.3)	5 (0.9)	44 (1.1)	5 (0.4)	*
**Atrial fibrillation**	69 (1.1)	9 (2.6)	23 (0.7)	11 (0.5)	2 (0.4)	15 (0.4)	4 (0.3)	*

**TIA**	58 (0.9)	7 (2.0)	24 (0.8)	15 (0.7)	7 (1.3)	13 (0.3)	6 (0.5)	*
**IBD**	24 (0.4)	1 (0.3)	2 (0.1)	2 (0.1)	1 (0.2)	9 (0.2)	1 (0.1)	*
**Multiple sclerosis**	21 (0.3)	1 (0.3)	3 (0.1)	1 (0.0)	0 (0.0)	4 (0.1)	2 (0.2)	*
**Chronic liver disease**	9 (0.1)	1 (0.3)	1 (0.0)	6 (0.3)	0 (0.0)	5 (0.1)	0 (0.0)	*
**Chronic sinusitis**	1 (0.0)	0 (0.0)	3 (0.1)	1 (0.0)	0 (0.0)	1 (0.0)	0 (0.0)	*

§Categories were dropped for statistical analysis. *χ2 test not performed due to small population sizes

Table 4b. Prevalence for each condition across ethnicities in SSD.

	**White British n=4008 (30.8%)**	**Irish n=240 (1.8%)**	**Black Caribbean n=2799 (21.5%)**	**Black African n=1886 (14.5%)**	**South Asian n=421 (3.2%)**	**Other§ n=2822 (21.7%)**	**Unknown§ n=843 (6.5%)**	**Statistics**
**Diabetes**	602 (15.0)	37 (15.4)	682 (24.4)	351 (18.6)	87 (20.7)	390 (13.8)	59 (7.0)	χ^2 (4) = 97.10; p <0.001
**Hypertension**	573 (14.3)	47 (19.6)	606 (21.7)	396 (21.0)	77 (18.3)	322 (11.4)	49 (5.8)	χ^2 (4) = 73.74; p <0.001
**Asthma**	443 (11.1)	23 (9.6)	408 (14.6)	134 (7.1)	26 (6.2)	217 (7.7)	40 (4.7)	χ^2 (4) = 75.96; p <0.001
**Arthritis**	252 (6.3)	17 (7.1)	206 (7.4)	86 (4.6)	29 (6.9)	103 (3.6)	9 (1.1)	χ^2 (4) = 15.48; p = 0.038
**Epilepsy**	261 (6.5)	23 (9.6)	141 (5.0)	70 (3.7)	14 (3.3)	117 (4.1)	26 (3.1)	χ^2 (4) = 32.45; p <0.001
**Cerebrovascular accident**	188 (4.7)	15 (6.2)	159 (5.7)	82 (4.3)	18 (4.3)	96 (3.4)	15 (1.8)	χ^2 (4) = 6.48; p >0.99
**Eczema**	145 (3.6)	4 (1.7)	159 (5.7)	54 (2.9)	12 (2.9)	93 (3.3)	12 (1.4)	χ^2 (4) = 33.32; p <0.001
**Migraine**	126 (3.1)	10 (4.2)	110 (3.9)	48 (2.5)	12 (2.9)	59 (2.1)	7 (0.8)	χ^2 (4) = 8.03; p = 0.904
**Ischemic heart disease**	187 (4.7)	9 (3.8)	98 (3.5)	46 (2.4)	13 (3.1)	74 (2.6)	8 (0.9)	χ^2 (4) = 19.15; p = 0.007
**COPD**	190 (4.7)	18 (7.5)	51 (1.8)	16 (0.8)	9 (2.1)	44 (1.6)	14 (1.7)	χ^2 (4) = 101.62; p <0.001
**CKD**	64 (1.6)	5 (2.1)	45 (1.6)	26 (1.4)	10 (2.4)	24 (0.9)	5 (0.6)	*
**Parkinson's disease**	79 (2.0)	4 (1.7)	49 (1.8)	21 (1.1)	6 (1.4)	36 (1.3)	6 (0.7)	*

**Heart failure**	65 (1.6)	8 (3.3)	50 (1.8)	28 (1.5)	7 (1.7)	26 (0.9)	3 (0.4)	*
**Psoriasis**	66 (1.6)	2 (0.8)	9 (0.3)	6 (0.3)	4 (1.0)	37 (1.3)	5 (0.6)	*
**Atrial fibrillation**	46 (1.1)	7 (2.9)	22 (0.8)	11 (0.6)	1 (0.2)	10 (0.4)	3 (0.4)	*
**TIA**	34 (0.8)	5 (2.1)	21 (0.8)	13 (0.7)	6 (1.4)	8 (0.3)	4 (0.5)	*
**IBD**	12 (0.3)	1 (0.4)	2 (0.1)	2 (0.1)	1 (0.2)	6 (0.2)	1 (0.1)	*
**Multiple sclerosis**	12 (0.3)	1 (0.4)	3 (0.1)	0 (0.0)	0 (0.0)	2 (0.1)	1 (0.1)	*
**Chronic liver disease**	7 (0.2)	1 (0.4)	1 (0.0)	6 (0.3)	0 (0.0)	5 (0.2)	0 (0.0)	*
**Chronic sinusitis**	1 (0.0)	0 (0.0)	3 (0.1)	1 (0.1)	0 (0.0)	1 (0.0)	0 (0.0)	*

§Categories were dropped for statistical analysis. *χ2 test not performed due to small population sizes

Table 4c. Prevalence for each condition across ethnicities in BD.

	**White British n=2235 (49.9%)**	**Irish n=106 (2.4%)**	**Black Caribbean n=383 (8.5%)**	**Black African n=208 (4.6%)**	**South Asian n=128 (2.9%)**	**Other§ n=1024 (22.9%)**	**Unknown§ n=397 (8.9%)**	**Statistics**
**Diabetes**	235 (10.5)	18 (17.0)	75 (19.6)	31 (14.9)	38 (29.7)	71 (6.9)	10 (2.5)	χ^2 (4) = 60.65; p <0.001
**Hypertension**	231 (10.3)	16 (15.1)	72 (18.8)	34 (16.3)	25 (19.5)	78 (7.6)	11 (2.8)	χ^2 (4) = 32.99; p <0.001
**Asthma**	208 (9.3)	17 (16.0)	76 (19.8)	22 (10.6)	16 (12.5)	79 (7.7)	13 (3.3)	χ^2 (4) = 39.95; p <0.001
**Arthritis**	150 (6.7)	12 (11.3)	40 (10.4)	4 (1.9)	7 (5.5)	36 (3.5)	3 (0.8)	χ^2 (4) = 19.09; p = 0.004
**Epilepsy**	78 (3.5)	3 (2.8)	23 (6.0)	7 (3.4)	3 (2.3)	30 (2.9)	3 (0.8)	*
**Cerebrovascular accident**	86 (3.8)	5 (4.7)	25 (6.5)	10 (4.8)	7 (5.5)	20 (2.0)	2 (0.5)	*
**Eczema**	77 (3.4)	3 (2.8)	26 (6.8)	7 (3.4)	5 (3.9)	15 (1.5)	4 (1.0)	*
**Migraine**	109 (4.9)	2 (1.9)	19 (5.0)	13 (6.2)	5 (3.9)	39 (3.8)	5 (1.3)	χ^2 (4) = 3.17; p >0.99
**Ischemic heart disease**	74 (3.3)	4 (3.8)	10 (2.6)	2 (1.0)	6 (4.7)	25 (2.4)	5 (1.3)	*

**COPD**	81 (3.6)	14 (13.2)	12 (3.1)	5 (2.4)	5 (3.9)	16 (1.6)	1 (0.3)	*
**CKD**	61 (2.7)	4 (3.8)	11 (2.9)	6 (2.9)	3 (2.3)	13 (1.3)	2 (0.5)	*
**Parkinson's disease**	38 (1.7)	2 (1.9)	7 (1.8)	4 (1.9)	5 (3.9)	9 (0.9)	0 (0.0)	*
**Heart failure**	23 (1.0)	0 (0.0)	5 (1.3)	1 (0.5)	1 (0.8)	5 (0.5)	0 (0.0)	*
**Psoriasis**	37 (1.7)	2 (1.9)	3 (0.8)	0 (0.0)	1 (0.8)	7 (0.7)	0 (0.0)	*
**Atrial fibrillation**	23 (1.0)	2 (1.9)	1 (0.3)	0 (0.0)	1 (0.8)	5 (0.5)	1 (0.3)	*
**TIA**	24 (1.1)	2 (1.9)	3 (0.8)	2 (1.0)	1 (0.8)	5 (0.5)	2 (0.5)	*
**IBD**	12 (0.5)	0 (0.0)	0 (0.0)	0 (0.0)	0 (0.0)	3 (0.3)	0 (0.0)	*
**Multiple sclerosis**	9 (0.4)	0 (0.0)	0 (0.0)	1 (0.5)	0 (0.0)	2 (0.2)	1 (0.3)	*
**Chronic liver disease**	2 (0.1)	0 (0.0)	0 (0.0)	0 (0.0)	0 (0.0)	0 (0.0)	0 (0.0)	*
**Chronic sinusitis**	0 (0.0)	0 (0.0)	0 (0.0)	0 (0.0)	0 (0.0)	0 (0.0)	0 (0.0)	*

[§]Categories were dropped for statistical analysis. *$\chi 2$ test not performed due to small population sizes

Supplemental Table 5. Social deprivation prevalence for each condition for the whole cohort and within SSD and BD.

	**Total cohort**						**SSD**							**AD**						
	**1 - least deprived n (%)**	**2 n (%)**	**3 n (%)**	**4 n (%)**	**5 - most deprived n (%)**	**Not stated§ n (%)**	**1 - least deprived n (%)**	**2 n (%)**	**3 n (%)**	**4 n (%)**	**5 - most deprived n (%)**	**Not stated§ n (%)**	**Chi square**	**1 - least deprived n (%)**	**2 n (%)**	**3 n (%)**	**4 n (%)**	**5 - most deprived n (%)**	**Not stated§ n (%)**	**Chi square**
**Total**	742 (100)	1384 (100)	3575 (100)	7073 (100)	4033 (100)	693 (100)	412 (100)	887 (100)	2503 (100)	5476 (100)	3204 (100)	537 (100)		330 (100)	497 (100)	1072 (100)	1597 (100)	829 (100)	156 (100)	
**Diabetes *****	62 (8.4)	169 (12.2)	528 (14.8)	1201 (17.0)	698 (17.3)	28 (4.0)	49 (11.9)	117 (13.2)	409 (16.3)	1019 (18.6)	594 (18.5)	20 (3.7)	$X^2(4)$ = 29.7; p<0.001	13 (3.9)	52 (10.5)	119 (11.1)	182 (11.4)	104 (12.5)	8 (5.1)	$X^2(4)$ = 19.4; p=0.007
**Hypertension *****	77 (10.4)	154 (11.1)	468 (13.1)	1189 (16.8)	627 (15.5)	22 (3.2)	51 (12.4)	108 (12.2)	368 (14.7)	1008 (18.4)	517 (16.1)	18 (3.4)	$X^2(4)$ = 37.8; p<0.001	26 (7.9)	46 (9.3)	100 (9.3)	181 (11.3)	110 (13.3)	4 (2.6)	$X^2(4)$ = 12.3; p=0.17
**Asthma *****	42 (5.7)	95 (6.9)	361 (10.1)	758 (10.7)	432 (10.7)	34 (4.9)	27 (6.6)	61 (6.9)	251 (10.0)	589 (10.8)	337 (10.5)	26 (4.8)	$X^2(4)$ = 19.0; p=0.01	15 (4.5)	34 (6.8)	110 (10.3)	169 (10.6)	95 (11.5)	8 (5.1)	$X^2(4)$ = 19.2; p=0.008
**Arthritis**	36 (4.9)	56 (4.0)	188 (5.3)	411 (5.8)	256 (6.3)	7 (1.0)	18 (4.4)	36 (4.1)	135 (5.4)	312 (5.7)	197 (6.1)	4 (0.7)	$X^2(4)$ = 7.30; p>0.99	18 (5.5)	20 (4.0)	53 (4.9)	99 (6.2)	59 (7.1)	3 (1.9)	$X^2(4)$ = 7.51; p>0.99
**Epilepsy**	32 (4.3)	50 (3.6)	160 (4.5)	344 (4.9)	202 (5.0)	11 (1.2)	24 (5.8)	40 (4.5)	123 (4.9)	292 (5.3)	164 (5.1)	9 (1.7)	$X^2(4)$ = 1.79; p>0.99	8 (2.4)	10 (2.0)	37 (3.5)	52 (3.3)	38 (4.6)	2 (1.3)	$X^2(4)$ = 7.59; p>0.99
**CVA**	19 (2.6)	52 (3.8)	146 (4.1)	328 (4.6)	180 (4.5)	3 (0.4)	12 (2.9)	33 (3.7)	107 (4.3)	272 (5.0)	146 (4.6)	3 (0.6)	$X^2(4)$ = 6.55; p>0.99	7 (2.1)	19 (3.8)	39 (3.6)	56 (3.5)	34 (4.1)	0	$X^2(4)$ = 2.80; p>0.99
**Eczema**	22 (3.0)	45 (3.3)	120 (3.4)	273 (3.9)	147 (3.6)	9 (1.3)	17 (4.1)	34 (3.8)	86 (3.4)	212 (3.9)	123 (3.8)	7 (1.3)	$X^2(4)$ = 1.11; p>0.99	5 (1.5)	11 (2.2)	34 (3.2)	61 (3.8)	24 (2.9)	2 (1.3)	$X^2(4)$ = 6.90; p>0.99
**Migraine**	17 (2.3)	39 (2.8)	121 (3.4)	243 (3.4)	140 (3.5)	4 (0.6)	11 (2.7)	20 (2.3)	63 (2.5)	176 (3.2)	99 (3.1)	3 (0.6)	$X^2(4)$ = 4.79; p>0.99	6 (1.8)	19 (3.8)	58 (5.4)	67 (4.2)	41 (4.9)	1 (0.6)	$X^2(4)$ = 8.94; p=0.69
**Ischaemic heart disease**	20 (2.7)	48 (3.5)	103 (2.9)	240 (3.4)	143 (3.5)	7 (1.0)	9 (2.2)	33 (3.7)	74 (3.0)	194 (3.5)	120 (3.7)	5 (0.9)	$X^2(4)$ = 4.99; p>0.99	11 (3.3)	15 (3.0)	29 (2.7)	46 (2.9)	23 (2.8)	2 (1.3)	$X^2(4)$ = 0.43; p>0.99
**COPD ****	6 (0.8)	35 (2.5)	83 (2.3)	208 (2.9)	139 (3.4)	5 (0.7)	3 (0.7)	22 (2.5)	59 (2.4)	159 (2.9)	95 (3.0)	4 (0.7)	$X^2(4)$ = 9.07; p=0.77	3 (0.9)	13 (2.6)	24 (2.2)	49 (3.1)	44 (5.3)	1 (0.6)	$X^2(4)$ = 21.9; p=0.002

**CKD**	8 (1.1)	22 (1.6)	46 (1.3)	128 (1.8)	74 (1.8)	1 (0.1)	4 (1.0)	9 (1.0)	28 (1.1)	81 (1.5)	56 (1.7)	1 (0.2)	$X^2(4) = 5.83$; p>0.99	4 (1.2)	13 (2.6)	18 (1.7)	47 (2.9)	18 (2.2)	0	$X^2(4) = 6.76$; p>0.99
**PD**	9 (1.2)	22 (1.6)	51 (1.4)	121 (1.7)	60 (1.5)	3 (0.4)	5 (1.2)	16 (1.8)	33 (1.3)	97 (1.8)	48 (1.5)	2 (0.4)	$X^2(4) = 3.13$; p>0.99	4 (1.2)	6 (1.2)	18 (1.7)	24 (1.5)	12 (1.4)	1 (0.6)	---
**Heart failure**	10 (1.3)	12 (0.9)	47 (1.3)	92 (1.3)	58 (1.4)	3 (0.4)	7 (1.7)	10 (1.1)	39 (1.6)	78 (1.4)	52 (1.6)	1 (0.2)	$X^2(4) = 1.54$; p=0.82	3 (0.9)	2 (0.4)	8 (0.7)	14 (0.9)	6 (0.7)	2 (1.3)	---
**Psoriasis**	11 (1.5)	12 (0.9)	33 (0.9)	71 (1.0)	48 (1.2)	4 (0.6)	4 (1.0)	6 (0.7)	24 (1.0)	54 (1.0)	37 (1.2)	4 (0.7)	---	7 (2.1)	6 (1.2)	9 (0.8)	17 (1.1)	11 (1.3)	0	---
**Atrial fibrillation**	10 (1.3)	11 (0.8)	27 (0.8)	53 (0.7)	29 (0.7)	3 (0.4)	6 (1.5)	7 (0.8)	21 (0.8)	42 (0.8)	23 (0.7)	1 (0.2)	---	4 (1.2)	4 (0.8)	6 (0.6)	11 (0.7)	6 (0.7)	2 (1.3)	---
**TIA**	3 (0.3)	17 (1.0)	27 (0.6)	55 (0.6)	28 (0.5)	0	1 (0.2)	9 (1.0)	16 (0.6)	43 (0.8)	22 (0.7)	0	---	2 (0.6)	8 (1.6)	11 (1.0)	12 (0.8)	6 (0.7)	0	---
**IBD**	1 (0.1)	4 (0.3)	10 (0.3)	12 (0.2)	13 (0.3)	0	0	1 (0.1)	7 (0.3)	7 (0.1)	10 (0.3)	0	---	1 (0.3)	3 (0.6)	3 (0.3)	5 (0.3)	3 (0.4)	0	---
**MS**	4 (0.5)	2 (0.1)	7 (0.2)	14 (0.2)	4 (0.1)	1 (0.1)	2 (0.5)	2 (0.2)	2 (0.1)	9 (0.2)	3 (0.1)	1 (0.2)	---	2 (0.6)	0	5 (0.5)	5 (0.3)	1 (0.1)	0	---
**Chronic liver disease**	0	1 (0.1)	8 (0.2)	8 (0.1)	5 (0.1)	0	0	0	8 (0.3)	8 (0.1)	4 (0.1)	0	---	0	1 (0.2)	0	0	1 (0.1)	0	---
**Chronic sinusitis**	0	0	1 (0.0)	2 (0.0)	3 (0.1)	0	0	0	1 (0.0)	2 (0.0)	3 (0.1)	0	---	0	0	0	0	0	0	---

*, p<0.05; **, p≤0.01; ***, p≤0.001; CVA, cerebrovascular accident; COPD, chronic obstructive lung disease; CKD, chronic kidney disease; PD, Parkinson's disease; TIA, transient ischemic attack; IBD, inflammatory bowel disease; MS, multiple sclerosis

Supplemental Table 6. Sociodemographic features of cohort in HoNOS subsample analysis

	**Total n (%)**	**SSD n (%)**	**BD n (%)**
**Totals n (%)**	13650 (100.0)	10384 (76.1)	3266 (23.9)
**Sex*** **			
Female	6537 (47.9)	4526 (69.2)	2011 (30.8)
Male	7112 (52.1)	5858 (82.4)	1254 (17.6)
**Age at first SMI diagnosis*** **			
15 – 34	5821 (42.6)	4420 (42.6)	1401 (42.9(
35 – 44	3430 (25.1)	2183 (75.3)	716 (24.7)
45 – 54	2082 (15.3)	1594 (76.6)	488 (23.4)
55 – 64	1089 (8.0)	811 (74.5)	278 (25.5)
65+	1759 (12.9)	1376 (13.3)	383 (11.7)
**Ethnicity*** **			
White British	4571 (33.5)	2961 (64.8)	1610 (35.2)
Black Caribbean	2849 (20.9)	2515 (88.3)	334 (11.7)
Black African	1852 (13.6)	1679 (90.7)	173 (9.3)
South Asian	436 (3.2)	338 (77.5)	98 (22.5)
Irish	286 (2.1)	197 (68.9)	89 (31.1)
Other§	3127 (22.9)	2327 (74.4)	800 (25.6)
Not stated§	529 (3.9)	367 (3.5)	162 (5.0)
**Index of multiple deprivation*** **			
1 (least deprivation)	380 (2.8)	237 (62.4)	143 (37.6)
2	883 (6.5)	581 (65.8)	302 (34.2)
3	2786 (20.4)	1994 (71.6)	792 (28.4)
4	5947 (43.6)	4654 (78.3)	1293 (21.7)
5 (most deprivation)	3308 (24.2)	2650 (80.1)	658 (19.9)
Unknown§	346 (2.5)	268 (77.5)	78 (22.5)
**Physical conditions*** **			
No mentions	7232 (53.0)	5320 (73.6)	1912 (26.4)
One	3295 (24.1)	2596 (78.8)	699 (21.2)
Two	1669 (12.2)	1340 (80.3)	329 (19.7)
Three or more	1454 (10.7)	1128 (77.6)	326 (22.4)

Note. *** $p < .001$ *for comparisons between BD and SSD groups.* §Not included in analyses.

Supplemental Table 7. Associations between comorbidities and HoNOS scores in SSD.

Comorbidity	HoNOS Score	Unadjusted	M1	M2a
	**Mean (SD)**	**B (95% CI)**	**B (95% CI)**	**B (95% CI)**
**Whole cohort**	10.75 (6.10)			
**Diabetes**	11.04 (6.10)	0.360 (0.067 – 0.654)**	0.224 (-0.073 – 0.522)	0.300 (-0.002 – 0.602)
Ref: No diabetes	10.68 (6.10)			
**Hypertension**	11.17 (6.05)	0.519 (0.220 – 0.818)***	0.264 (-0.048 – 0.575)	0.324 (0.006 – 0.641)*
Ref: No hypertension	10.65 (6.11)			
**Asthma**	11.19 (6.11)	0.492 (0.126 – 0.859)**	0.551 (0.185 – 0.918)**	0.530 (0.158 – 0.903)**
Ref: No asthma	10.70 (6.10)			
**Arthritis**	12.08 (6.21)	1.422 (0.948– 1.895)***	1.259 (0.775 – 1.742)***	1.274 (0.788 – 1.759)***
Ref: No arthritis	10.66 (6.08)			
**Epilepsy**	11.53 (6.24)	0.827 (0.316 – 1.337)***	0.850 (0.341 – 1.358)***	0.856 (0.341 – 1.370)***
Ref: No epilepsy	10.71 (6.09)			
**CVA**	11.87 (6.23)	1.178 (0.651 – 1.705)***	0.913 (0.383 – 1.443)***	0.921 (0.388 – 1.454)**
Ref: No CVA	10.69 (6.09)			
**Eczema**	11.48 (6.25)	0.763 (0.188 – 1.338)**	0.846 (0.273 – 1.420)**	0.754 (0.172 – 1.336)*
Ref: No eczema	10.72 (6.09)			
**Migraine**	10.71 (5.87)	-0.041 (-0.683 – 0.602)	0.196 (-0.447 – 0.839)	0.192 (-0.458 – 0.842)
Ref: No migraine	10.75 (6.11)			
**Ischaemic heart disease**	11.59 (5.91)	0.874 (0.274 – 1.474)**	0.520 (-0.083 – 1.124)	0.477 (-0.132 – 1.085)
Ref: No ischaemic heart disease	10.72 (6.11)			
**COPD**	12.15 (5.70)	1.444 (0.760– 2.128)***	1.045 (0.354 – 1.736)**	1.043 (0.343 – 1.742)**
Ref: No COPD	10.71 (6.11)			
**Number of comorbidities**		0.387 (0.294 – 0.481)***	0.328 (0.231 – 0.425)***	0.342 (0.243 – 0.441) ***
**1 or more comorbidities**	11.13 (6.11)	0.745 (0.511 –0.980)***	0.607 (0.367 – 0.847)***	0.657 (0.410 – 0.903)***
Ref: No comorbidities	10.39 (6.07)			
**2 or more comorbidities**	11.45 (6.16)	0.921 (0.646 – 1.196)***	0.747 (0.464 – 1.030)***	0.771 (0.484 – 1.059)***
Ref: Less than 1 comorbidities	10.53 (6.07)			

Supplemental Table8. Associations between comorbidities and HoNOS scores in BD.

Comorbidity	HoNOS Score	Unadjusted	M1	M2a
	Mean (SD)	B (95% CI)	B (95% CI)	B (95% CI)
**Whole cohort**	9.28 (5.77)			
**Diabetes**	10.941 (5.97)	1.304 (0.720 – 1.887)***	0.968 (0.370 – 1.565)**	0.961 (0.355 – 1.568)**
Ref: No diabetes	9.11 (5.72)			
**Hypertension**	10.13 (5.82)	0.972 (0.384 – 1.561)**	0.446 (-0.182 – 1.074)	0.277 (-0.359 – 0.912)
Ref: No hypertension	9.15 (5.76)			
**Asthma**	10.61 (6.27)	1.512 (0.900 – 2.124)***	1.597 (0.985 – 2.210)***	1.503 (0.887 – 2.120)***
Ref: No asthma	9.10 (5.68)			
**Arthritis**	11.81 (6.13)	2.725 (1.960 – 3.490)***	2.487 (1.702 – 3.273)***	2.396 (1.606 – 3.186)***
Ref: No arthritis	9.09 (5.70)			
**Epilepsy**	10.77 (5.96)	1.552 (0.513 – 2.591)**	1.587 (0.551 – 2.624)**	1.496 (0.466 – 2.527)**
Ref: No epilepsy	9.22 (5.76)			
**CVA**	11.60 (6.16)	2.425 (1.464 – 3.386)***	2.098 (1.124 – 3.071)***	2.030 (1.064 – 2.996)***
Ref: No CVA	9.17 (5.73)			
**Eczema**	9.83 (5.82)	0.578 (-0.446 – 1.603)	0.622 (-0.400 – 1.644)	0.427 (-0.603 – 1.458)
Ref: No eczema	9.26 (5.77)			
**Migraine**	9.55 (5.34)	0.284 (-0.586– 1.154)	0.523 (-0.350– 1.397)	0.527 (-0.346 – 1.400)
Ref: No migraine	9.26 (5.80)			
**Ischaemic heart disease**	11.84 (5.95)	2.649 (1.560 – 3.738)***	2.093 (0.987 – 3.200)***	2.204 (1.098 – 3.309)**
Ref: No ischaemic heart disease	9.19 (5.75)			
**COPD**	11.89 (5.99)	2.711 (1.679 – 3.743)***	2.298 (1.248 – 3.348)***	2.128 (1.077 – 3.179)***
Ref: No COPD	9.18 (5.74)			
**Number of comorbidities**		0.723 (0.566 – 0.879)***	0.661 (0.494 – 0.828)***	0.642 (0.473 – 0.811) ***
**1 or more comorbidities**	10.30 (5.91)	1.740 (1.343 –2.138)***	1.559 (1.143 – 1.974)***	1.457 (1.031 – 1.883)***
Ref: No comorbidities	8.56 (5.57)			
**2 or more comorbidities**	10.89 (5.99)	2.013 (1.524 – 2.503)***	1.797 (1.280 – 2.314)***	1.789 (1.267 – 2.311)***
Ref: Less than 1 comorbidities	8.88 (5.64)			

Supplemental Figure 1. Distribution of all conditions in the SMI cohort by SMI diagnoses

SMI (total)

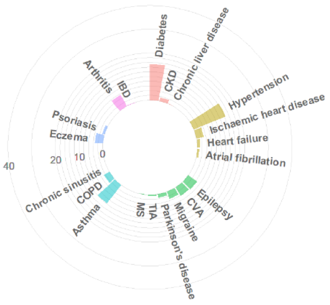

SMI (SSD)

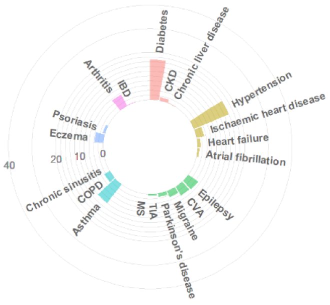

SMI (BD)

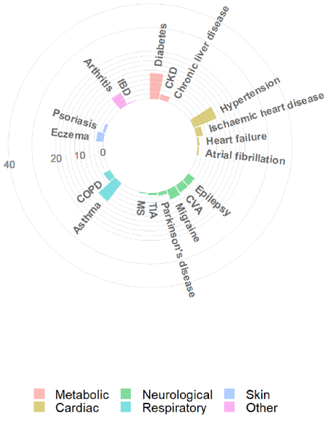

Supplemental Figure 2. Prevalence rates for age at SMI diagnoses per condition and comparison between individuals with BD and SSD.

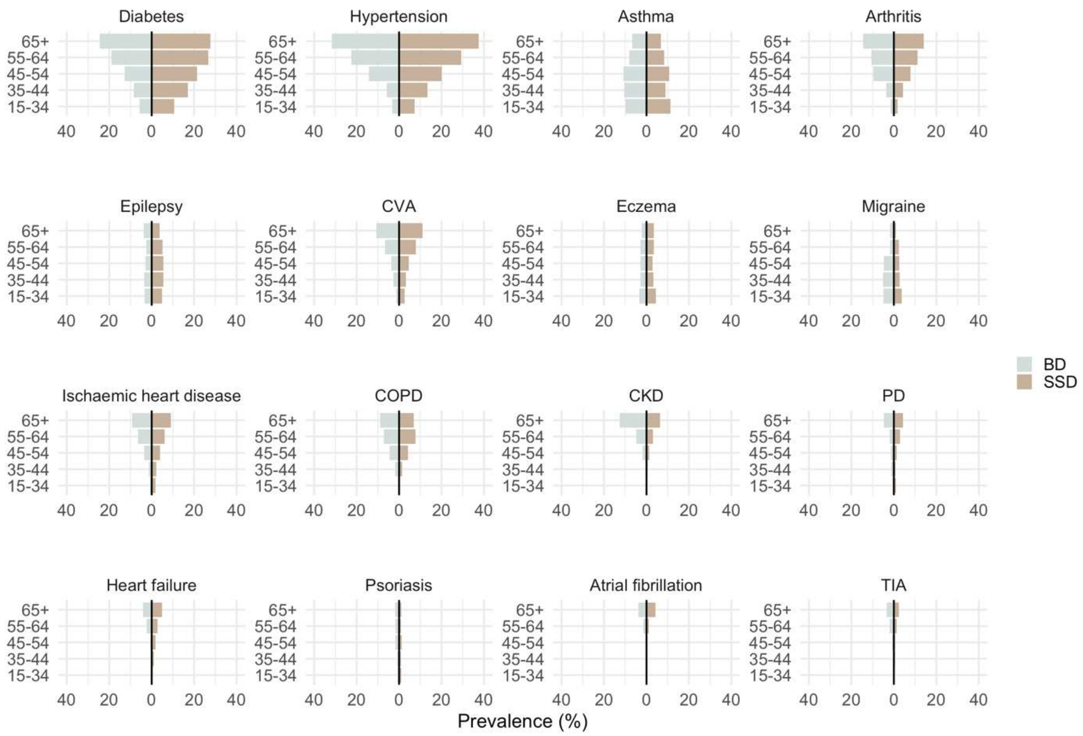

Appendix 1. SNOMED Container and Concept Level Groupings for physical health conditions included in this study.

Container Concept	Concepts
S-73211009 - Diabetes mellitus (disorder)	S-44054006 - Diabetes mellitus type 2 (disorder)<br>S-46635009 - Diabetes mellitus type 1 (disorder)<br>S-422088007 - Disorder of nervous system co-occurrent and due to diabetes mellitus (disorder)<br>S-25093002 - Disorder of eye co-occurrent and due to diabetes mellitus (disorder)<br>S-73211009 - Diabetes mellitus (disorder)
S-84114007 - Heart failure (disorder)	S-128404006 - Right heart failure (disorder)<br>S-48447003 - Chronic heart failure (disorder)<br>S-56675007 - Acute heart failure (disorder)<br>S-85232009 - Left heart failure (disorder)<br>S-42343007 - Congestive heart failure (disorder)<br>S-84114007 - Heart failure (disorder)
S-414545008 - Ischemic heart disease (disorder)	S-413439005 - Acute ischemic heart disease (disorder)<br>S-413838009 - Chronic ischemic heart disease (disorder)<br>S-194828000 - Angina (disorder)<br>S-22298006 - Myocardial infarction (disorder)<br>S-414545008 - Ischemic heart disease (disorder)
S-38341003 - Hypertensive disorder, systemic arterial (disorder)	S-31992008 - Secondary hypertension (disorder)<br>S-48146000 - Diastolic hypertension (disorder)<br>S-56218007 - Systolic hypertension (disorder)<br>S-59621000 - Essential hypertension (disorder)<br>S-38341003 - Hypertensive disorder, systemic arterial (disorder)
S-13645005 - Chronic obstructive lung disease (disorder)	S-195951007 - Acute exacerbation of chronic obstructive airways disease (disorder)<br>S-87433001 - Pulmonary emphysema (disorder)<br>S-13645005 - Chronic obstructive lung disease (disorder)
S-195967001 - Asthma (disorder)	S-195967001 - Asthma (disorder)
S-709044004 - Chronic kidney disease (disorder)	S-723190009 - Chronic renal insufficiency (disorder)<br>S-709044004 - Chronic kidney disease (disorder)

S-230690007 - Cerebrovascular accident (disorder)	S-25133001 - Completed stroke (disorder)<br>S-371040005 - Thrombotic stroke (disorder)<br>S-371041009 - Embolic stroke (disorder)<br>S-413102000 - Infarction of basal ganglia (disorder)<br>S-422504002 - Ischemic stroke (disorder)<br>S-723082006 - Silent cerebral infarct (disorder)<br>S-1078001000000105 - Haemorrhagic stroke (disorder)<br>S-230690007 - Cerebrovascular accident (disorder)
S-266257000 - Transient ischemic attack (disorder)	S-266257000 - Transient ischemic attack (disorder)
S-49049000 - Parkinson's disease (disorder)	S-49049000 - Parkinson's disease (disorder)
S-24700007 - Multiple sclerosis (disorder)	S-24700007 - Multiple sclerosis (disorder)
S-84757009 - Epilepsy (disorder)	S-352818000 - Tonic-clonic epilepsy (disorder)<br>S-19598007 - Generalized epilepsy (disorder)<br>S-230456007 - Status epilepticus (disorder)<br>S-509341000000107 - Petit-mal epilepsy (disorder)<br>S-84757009 - Epilepsy (disorder)
S-37796009 - Migraine (disorder)	S-37796009 - Migraine (disorder)<br>S-4473006 - Migraine with aura (disorder)<br>S-56097005 - Migraine without aura (disorder)
S-53741008 - Coronary arteriosclerosis (disorder)	S-810681000000101 - Coronary microvascular disease (disorder)<br><br>S-53741008 - Coronary arteriosclerosis (disorder)
S-49436004 - Atrial fibrillation (disorder)	S-49436004 - Atrial fibrillation (disorder)
S-40055000 - Chronic sinusitis (disorder)	S-40055000 - Chronic sinusitis (disorder)
S-24526004 - Inflammatory bowel disease (disorder)	S-24526004 - Inflammatory bowel disease (disorder)<br>S-397173003 - Crohn's disease of intestine (disorder)<br>S-64766004 - Ulcerative colitis (disorder)
S-328383001 - Chronic liver disease (disorder)	S-328383001 - Chronic liver disease (disorder)<br>S-76783007 - Chronic hepatitis (disorder)<br>S-79720007 - Chronic nonalcoholic liver disease (disorder)

	S-713181003 - Chronic alcoholic liver disease (disorder)
S-9014002 - Psoriasis (disorder)	S-9014002 - Psoriasis (disorder)
S-43116000 - Eczema (disorder)	S-43116000 - Eczema (disorder)
S-3723001 - Arthritis (disorder)	S-69896004 - Rheumatoid arthritis (disorder)<br>S-399112009 - Seronegative arthritis (disorder)<br>S-35908007 - Chronic arthritis (disorder)<br>S-11939005 - Acute arthritis (disorder)<br>S-3723001 - Arthritis (disorder)
