# Supplementary material for: Mapping multimorbidity in individuals with schizophrenia and bipolar disorders: evidence from the South London and Maudsley NHS Foundation Trust Biomedical Research Centre (SLAM BRC) case register
Source: BMJ Open. 2022 Jan 24;12(1):e054414. doi: 10.1136/bmjopen-2021-054414 (PMC8788233; doi:10.1136/bmjopen-2021-054414)
Supplement: Author's manuscript [file bmjopen-2021-054414.draft_revisions.pdf]

BMJ Open

BMJ Open is committed to open peer review. As part of this commitment we make the peer review history of every article we publish publicly available.

When an article is published we post the peer reviewers' comments and the authors' responses online. We also post the versions of the paper that were used during peer review. These are the versions that the peer review comments apply to.

The versions of the paper that follow are the versions that were submitted during the peer review process. They are not the versions of record or the final published versions. They should not be cited or distributed as the published version of this manuscript.

BMJ Open is an open access journal and the full, final, typeset and author-corrected version of record of the manuscript is available on our site with no access controls, subscription charges or pay-per-view fees (http://bmjopen.bmj.com).

If you have any questions on BMJ Open's open peer review process please email info.bmjopen@bmj.com

BMJ Open

Mapping Multimorbidity in Individuals with Schizophrenia and Bipolar Disorders: Evidence from the South London and Maudsley NHS Foundation Trust Biomedical Research Centre (SLAM BRC) Case Register

Journal:	*BMJ Open*
Manuscript ID	bmjopen-2021-054414
Article Type:	Original research
Date Submitted by the Author:	10-Jun-2021

Complete List of Authors:	Bendayan, Rebecca; King's College London, Biostatistics and Health Informatics, Institute of Psychiatry, Psychology and Neurosciences; NIHR Biomedical Research Centre at South London and Maudsley NHS Foundation Trust and King's College London, London, United Kingdom<br>Kraljevic, Zeljko; King's College London, Biostatistics and Health Informatics, Institute of Psychiatry, Psychology and Neuroscience<br>Shaari, Shaweena; NIHR Biomedical Research Centre at South London and Maudsley NHS Foundation Trust and King's College London, London, United Kingdom<br>Das-Munshi, Jayati; King's College London Institute of Psychiatry Psychology and Neuroscience, Psychological Medicine<br>Leipold, Leona; NIHR Biomedical Research Centre at South London and Maudsley NHS Foundation Trust and King's College London, London, United Kingdom<br>Chaturvedi, Jaya; King's College London, Biostatistics and Health Informatics, Institute of Psychiatry, Psychology and Neuroscience<br>Mirza, Luwaiza; NIHR Biomedical Research Centre at South London and Maudsley NHS Foundation Trust and King's College London, London, United Kingdom<br>Aldelemi, Sarah; NIHR Biomedical Research Centre at South London and Maudsley NHS Foundation Trust and King's College London, London, United Kingdom<br>Searle, Thomas; King's College London, Department of Biostatistics and Health Informatics<br>Chance, Natalia; NIHR Biomedical Research Centre at South London and Maudsley NHS Foundation Trust and King's College London, London, United Kingdom<br>Mascio, Aurelie; King's College London, Biostatistics and Health Informatics, Institute of Psychiatry, Psychology and Neuroscience<br>Skiada, Naoko; King's College London, Biostatistics and Health Informatics, Institute of Psychiatry, Psychology and Neuroscience<br>Wang, Tao; Department of Biostatistics and Health Informatics, Institute of Psychiatry, Psychology and Neuroscience, King's College London, London, United Kingdom<br>Roberts, Angus; King's College London, Biostatistics and Health Informatics, Institute of Psychiatry, Psychology and Neuroscience; NIHR Biomedical Research Centre at South London and Maudsley NHS Foundation Trust and King's College London, London, United Kingdom

	Stewart, Robert; King's College London, Psychological Medicine; NIHR Biomedical Research Centre at South London and Maudsley NHS Foundation Trust and King’s College London, London, United Kingdom<br>Bean, Daniel; King's College London, Biostatistics and Health Informatics<br>Dobson, Richard; King's College London,

Keywords:	Health informatics < BIOTECHNOLOGY & BIOINFORMATICS, EPIDEMIOLOGY, MENTAL HEALTH, PSYCHIATRY, PUBLIC HEALTH

SCHOLARONE™
Manuscripts

I, the Submitting Author has the right to grant and does grant on behalf of all authors of the Work (as defined in the below author licence), an exclusive licence and/or a non-exclusive licence for contributions from authors who are: i) UK Crown employees; ii) where BMJ has agreed a CC-BY licence shall apply, and/or iii) in accordance with the terms applicable for US Federal Government officers or employees acting as part of their official duties; on a worldwide, perpetual, irrevocable, royalty-free basis to BMJ Publishing Group Ltd ("BMJ") its licensees and where the relevant Journal is co-owned by BMJ to the co-owners of the Journal, to publish the Work in this journal and any other BMJ products and to exploit all rights, as set out in our licence.

The Submitting Author accepts and understands that any supply made under these terms is made by BMJ to the Submitting Author unless you are acting as an employee on behalf of your employer or a postgraduate student of an affiliated institution which is paying any applicable article publishing charge ("APC") for Open Access articles. Where the Submitting Author wishes to make the Work available on an Open Access basis (and intends to pay the relevant APC), the terms of reuse of such Open Access shall be governed by a Creative Commons licence – details of these licences and which Creative Commons licence will apply to this Work are set out in our licence referred to above.

Other than as permitted in any relevant BMJ Author's Self Archiving Policies, I confirm this Work has not been accepted for publication elsewhere, is not being considered for publication elsewhere and does not duplicate material already published. I confirm all authors consent to publication of this Work and authorise the granting of this licence.

For peer review only

Mapping Multimorbidity in Individuals with Schizophrenia and Bipolar Disorders: Evidence from the South London and Maudsley NHS Foundation Trust Biomedical Research Centre (SLAM BRC) Case Register

Rebecca Bendayan[1,2], Zeljko Kraljevic[2], Shaweena Shaari[1], Jayati Das-Munshi[3], Leona Leipold[1], Jaya Chaturvedi[2], Luwaiza Mirza[1], Sarah Aldelemi[1], Tom Searle[2], Natalia Chance[1], Aurelie Mascio[2], Naoko Skiada[2], Angus Roberts[1,2], Robert Stewart[1,3], Daniel Bean[2,4], Richard Dobson[1,2,5]

[1]NIHR Biomedical Research Centre at South London and Maudsley NHS Foundation Trust and King's College London, London, United Kingdom

[2]Department of Biostatistics and Health Informatics, Institute of Psychiatry, Psychology and Neuroscience, King's College London, London, United Kingdom

[3]Department of Psychological Medicine, Institute of Psychiatry, Psychology and Neuroscience, King's College London, London, United Kingdom

[4]Health Data Research UK London, University College London, London, United Kingdom

[5]Institute of Health Informatics, University College London, London, United Kingdom

Word count: 3920

Correspondence:
Rebecca Bendayan / ORCID: 0000-0003-1461-556X
E-mail: rebecca.bendayan@kcl.ac.uk
NIHR Maudsley Biomedical Research Centre
Department of Biostatistics & Health Informatics
SGDP Centre, IoPPN, Box PO 80
De Crespigny Park, Denmark Hill
London SE5 8AF, UNITED KINGDOM

For peer review only

Abstract

Objectives: The first aim of this study was to design and develop a valid and replicable strategy to extract physical health conditions from clinical notes which are common in mental health services. Then, we examined the prevalence of these conditions in individuals with SMI and compared their individual and combined prevalence in individuals with bipolar (BD) and schizophrenia spectrum disorders (SSD).

Design: Observational study.

Setting: Secondary mental healthcare services from South London

Participants: Our maximal sample comprised 17,500 individuals aged 15 years or older who had received a primary or secondary SMI diagnosis (ICD-10, F20-31) between 2007 and 2018.

Measures: We designed and implemented a data extraction strategy for 21 common physical comorbidities using a natural language processing pipeline, MedCAT. Associations were investigated with sex, age at SMI diagnosis, ethnicity and social deprivation for the whole cohort and the BD and SSD subgroups. Linear regression models were used to examine associations with disability measured by the Health of Nations Outcome Scale (HoNOS).

Results: Physical health data was extracted, achieving precision rates (F1) above 0.90 for all conditions. The ten most prevalent conditions were diabetes, hypertension, asthma, arthritis, epilepsy, cerebrovascular accident, eczema, migraine, ischaemic heart disease and COPD. The most prevalent combination in this population included diabetes, hypertension and asthma, regardless of their SMI diagnoses.

Conclusions: Our data extraction strategy was found to be adequate to extract physical health data from clinical notes, which is essential for future multimorbidity research using text records. We found that around 40% of our cohort had multimorbidity from which 20% had complex

For peer review only

multimorbidity (two or more physical conditions besides SMI). Sex, age, ethnicity and social deprivation were found to be key to understand their heterogeneity and their differential contribution to disability levels in this population. These outputs have direct implications for researchers and clinicians.

Strengths and limitations of this study

- We designed and implemented a data extraction strategy with good which showed high performance rates and allowed us to unlock data from 21 physical health conditions from around 15m clinical documents with free text.
- We mapped how these health conditions are distributed across sex, age, ethnicity, social disadvantage and severe mental illness diagnoses in a sample of 17500 patients from one of Europe's largest providers of secondary mental healthcare, serving a geographic catchment of approximately 1.32 million residents in London.
- The ten most prevalent conditions in this SMI cohort were diabetes, hypertension, asthma, arthritis, epilepsy, cerebrovascular accident, eczema, migraine, ischaemic heart disease and COPD. The most prevalent combination in this population included diabetes, hypertension and asthma, regardless of their SMI diagnoses.
- Multimorbidity (2 or more conditions) is associated with high levels of disability in this population and independent reports of any of the ten most prevalent conditions are also associated with high levels of disability.
- Further research is needed to understand potential explanatory pathways for the association between multimorbidity and disability in this population.

For peer review only

Introduction

Two thirds of the deaths in individuals with severe mental illness (SMI) are potentially explained by the increased risk of multimorbidity in this population.[1–4] However, multimorbidity research in this population is still scarce[5] compounded by the limited availability of physical health data in SMI samples, increased non-response rates in surveys,[6] and physical health information in secondary mental health care data primarily hidden in free text fields.

Most research to date on physical health in SMI populations has focused on cardiometabolic risk factors which are considered leading contributors to cardiovascular diseases in individuals with SMI,[7–10] or specific conditions such as immune-mediated inflammatory diseases (e.g., inflammatory bowel diseases, psoriasis),[11–13] multiple sclerosis, epilepsy or migraine.[14,15] This condition-specific vision limits our understanding of multimorbidity in SMI and studies that consider a larger number of conditions are needed. However, there are only a few studies which have considered multiple health conditions.[4,16,17] Woodhead et al.[4] showed an increased risk in multimorbidity in SMI patients, but only found epilepsy to be more prevalent as an individual condition. Kugathasan et al.[17] investigated combinations of diseases in schizophrenia at organ system level and found that 31% had complex multimorbidity with the most prevalent pairs including neurologic-endocrine, neurologic-respiratory and neurologic-viral. Similarly, epilepsy and arthritis was one of the most prevalent combinations found by Dorrington et al.[16] Although these studies included multiple health conditions and confirmed the need of investigating further multimorbidity in SMI, they are still not comparable to multimorbidity studies in general populations[18–20] and did not investigate potential differences between individuals with schizophrenia spectrum disorders (SSD) and bipolar disorders (BD). Understanding different multimorbidity combinations between those groups could contribute to the ongoing debate around

For peer review only

potential underlying biological mechanisms.[21–23] Ultimately, SSD and BD have been established as significant drivers of disability[24] and deficits in physical health have been implicated in the perpetuation of impairments in functional capacity and performance,[25,26] however, research into the relationship between multimorbidity and disability in SMI is limited.

Within this context, our first aim was to design and develop a suitable strategy to extract information on physical health conditions from free text mental health records data which could be easily replicated in future multimorbidity research using similar resources. Our second objective was to examine the prevalence of these conditions and their most prevalent combinations in SMI and any differences across relevant sociodemographic factors and across SMI diagnoses (SSD vs BD). Our third objective was to investigate the association of overall multimorbidity and specific physical health conditions with levels of disability measured using the Health of the Nation Outcome Scales (HoNOS).

Methods

Setting and sample

Patient data were extracted via the Clinical Record Interactive Search (CRIS), a case register platform that contains de-identified mental healthcare electronic health record data from the South London and Maudsley Trust NHS Foundation Trust (SLaM). SLaM is one of Europe's largest providers of secondary mental healthcare, serving a geographic catchment of approximately 1.32 million residents, and providing almost complete coverage of secondary mental healthcare provision to all age groups. Since 2007, fully electronic clinical records have been deployed in SLaM, and data from these are accessible via CRIS system which allows searching and retrieval of anonymized full records for over 500,000 cases currently represented in the system.[27]

For peer review only - http://bmjopen.bmj.com/site/about/guidelines.xhtml

Our sample (N=17500) consisted of all individuals aged 15 years or older who had received a primary or secondary SMI diagnosis between 2007 and 2018 (International Classification of Mental and Behavioural Disorders 10th edition [ICD-10][28] codes F20-31). As one of our objectives was to compare SSD (F20-29) and BD (F30-31), individuals who over those 10 years of follow-up had diagnoses within both categories were excluded (n=804). Excluded individuals were more likely to be female, under the age of 35 at first SMI diagnosis recorded, Black ethnicity and have higher levels of social deprivation.

Physical health conditions

Definitions and Information extraction. To maximise comparability we sought to extract the following 21 physical health conditions representing chronic conditions commonly collected in multimorbidity studies using primary care data:[18–20] diabetes mellitus, heart failure, ischemic heart diseases, hypertension, coronary arteriosclerosis, chronic obstructive pulmonary disease (COPD), asthma, chronic kidney disease, cerebrovascular accident, transient ischemic attack, Parkinson's disease, multiple sclerosis, epilepsy, migraine, atrial fibrillation, chronic sinusitis, inflammatory bowel disease, chronic liver diseases, psoriasis, eczema and arthritis. These were mapped to SNOMED codes where the top concept was the group identifier and then all direct children of that concept were examined and individually reviewed by two clinicians (Appendix 1). Physical health conditions were ascertained from data reported in text records from CRIS since 2007 until 1st August 2019 for each individual resulting in around 15m documents.

To extract SNOMED concepts from the free text we used MedCAT,[29] a medical concept annotation toolkit capable of named entity recognition linking (NER+L) with contextualization. The base model used is described in Kraljevic et al.,[29] and has shown very good performance (F1=0.90). In a first step, the base model was enriched with concept names from UMLS with the

For peer review only - http://bmjopen.bmj.com/site/about/guidelines.xhtml

purpose of increasing recall and potentially catching all different name-forms for each concept. In a second step, to increase precision, MedCAT was trained in an unsupervised fashion on all the available documents; and in a third step, all the free text was annotated for the chosen SNOMED concepts. For each condition, 300 documents were randomly extracted, which resulted in a total of 6300 annotated documents.

Annotation of physical health conditions. To ensure consistent, high-quality gold standard and training data, we developed annotation guidelines based on series of iterative discussions including clinical and technical expertise. These guidelines, available upon request, were piloted and refined in preliminary stages. A relevant instance was defined as a mention of a physical health condition experienced by the patient and not negated. Each MedCAT detection was first validated as either correct/wrong - meaning the portion of text that was detected by MedCAT was either a correct/wrong detection of the relevant concept. Correct detections were further annotated with contextual annotations (or meta-annotations) for 'Diagnosis' and 'Status'. Diagnosis was used to determine if the detected concept is a patient related diagnosis, and Status if the detected concept is affirmed. Eight annotators were trained for this task and given the same instructions. MedCATtrainer[30] was used to facilitate manual annotations and each document was double annotated. Disagreements between annotators were further evaluated and resolved by a third annotator.

Training and validation. Once the dataset was annotated it was split into a training and validation set. For NER+L, 70% of the dataset was used for training and 30% for validation. For meta-annotations, 80% was used for training and 20% for validation. Hyperparameter optimization in both cases used a 10-fold cross validation on the training set.

For peer review only

Socio-demographics. Extracted data included sex, age at SMI diagnosis (15-24, 25-34, 35-44, 45-54, 55-64, 65-74, 75+), and ethnicity (White British, Irish, Black Caribbean (including mixed White and Black Caribbean and any other Black background), Black African (including mixed White and Black African), South Asian (Indian, Pakistani and Bangladeshi) and Other). Index of Multiple Deprivation (IMD) was extracted as a measure of neighbourhood socioeconomic status at the level of the 2011 Lower Layer Super Output Area (LSOA11; a standard postal unit with an average 1500 residents) corresponding to the individual's address at time of SMI diagnosis. Using the IMD, each LSOA11 is ranked from 1 (most deprived) to 32,844 (least deprived) based on seven Census-derived indicators, which was subsequently divided into quintiles.[31]

Disability. Disability was measured using Health of the Nation Outcome Scales (HoNOS):[32] a clinician-rated tool developed to measure health and social functioning. It includes 12 subscales: agitated behaviour; non-accidental self-injury; problem drinking or drug taking; cognitive problems; physical illness problems; problems associated with hallucinations or delusions; problems associated with depression; other mental and behaviour problems; problems with relationships; problems with activities of daily living; problems with living conditions; and problems with occupation and activities.[32] Total adjusted HoNOS scores of individuals at the first SMI diagnosis recorded in CRIS, or closest to that time, were used in this study. Higher scores for HoNOS indicate higher levels of impairment in the individual's functioning.

Statistical Analyses

To explore the suitability of MedCAT for extracting these physical health conditions from this cohort (objective 1), inter-rater agreement estimates were computed and performance, precision and recall per condition were estimated.

For peer review only

To examine the prevalence of these conditions in SMI across relevant factors and compare the most prevalent multimorbidity combinations for individuals with BD and SSD (objective 2). Descriptive statistics were derived for all the variables. Chi-square tests and Fisher's exact tests, with Bonferroni correction for multiple comparisons, were performed to explore associations with covariates differences between BD and SSD.

To address our third objective (to investigate the association of multimorbidity and specific physical health with levels of disability), we performed series of hierarchical linear regressions. Models were adjusted by age and sex (Model 1), and then additionally adjusted by IMD (Model 2a) or SMI diagnosis (Model 2b). All analyses were performed using R 4.0.3 and RStudio 1.3.1093.

Patient and Public Involvement Statement

When designing this project, the Data Linkage Service User and Carer Advisory Group was consulted and followed up presenting preliminary results. This is a well-established Patient and Public Involvement Group set up by the Biomedical Research Center (BRC) at South London and Maudsley Trust NHS Foundation Trust (SLaM).[33]

Results

Inter-annotator agreement and model validation for data extraction

For each physical health condition, 300 documents were annotated to create a gold standard and training data specific to each condition. All 6300 instances across 21 health conditions were double annotated yielding an average inter-annotator agreement of 97% for NER+L, 82.70% for the meta-annotation Diagnosis and 78.08% for the meta-annotation Status. Precision, recall and F1 metrics of each modelled physical health condition are shown in Table 1. Coronary arteriosclerosis was not extracted as the number of positive mentions was too small for training and validation. Overall

meta-annotations performance results showed good performance for Diagnosis and Status (Supplemental Table 1).

PLEASE INSERT TABLE 1

Mapping of physical health conditions and comparison between SSD and BD

Our sample consisted of 17,500 individuals with SMI, of whom 74.4% were diagnosed with SSD and 25.6% with BD. A slight majority were male (53.6%), and most individuals had their first SMI diagnoses report under the age of 35 (42.8%). The White British group accounted for 35.7%, with Black Caribbean (18.2%) and Black African (12.0%) groups being the next two largest groups and South Asian and Irish groups were the smallest, with 3.1 and 2.0% respectively (Table 2). There were high levels of deprivation in the cohort, with over 60% falling into the lowest two national quintiles. Around 40% had at least one mention of a physical health condition and around 20% had two or more physical conditions. There were significant differences between BD and SSD for most of the socio-demographic characteristics and number of physical health conditions (Table 2 and Figure 1). Individuals with SSD were more likely to be men, from ethnic minorities, living in more deprived neighbourhoods and had a higher number of physical health conditions recorded compared those with BD.

The three most common physical health conditions recorded were diabetes, hypertension and asthma (15.3%, 14.5% and 9.8% respectively), regardless of the specific SMI diagnoses (Supplemental Figure 1 within SSD and BD). When we compared individuals with SSD and BD, we found that the top 10 most prevalent health conditions were similar between groups but diabetes (SSD 17% vs BD 10.7%), hypertension (SSD 15.9% vs BD 10.4%) and epilepsy (SSD 5.0% vs BD 3.3%) prevalence rates were slightly higher for individuals with SSD while individuals with BD showed higher prevalence rates of migraine (BD 4.3% vs SSD 2.9%).

For peer review only

PLEASE INSERT TABLE 2 AND FIGURE 1 HERE

When we explored differences by sex in the whole cohort, we found that women were more likely to report hypertension, asthma, arthritis, eczema, and migraine compared to men (Supplemental Table 2). Within the individuals with SSD, women were in addition found to be more likely to report higher rates of diabetes, chronic kidney disease (CKD), heart failure and transient ischaemic attack (TIA). Within individuals with BD, sex differences were only found for asthma, arthritis, migraine and ischemic heart disease. Women with BD were more likely to report asthma, arthritis and migraine while men with BD were more likely to report ischemic heart disease.

With regards to differences across age groups, we found higher prevalence rates of diabetes, hypertension, arthritis, cerebrovascular accident, ischaemic heart disease, COPD, CKD, Parkinson's disease, heart failure, atrial fibrillation and TIA in individuals in older age ranges, while asthma and migraine were more prevalent in those in younger age ranges (Supplemental Table 3, Supplemental Figure 2). Similar results within individuals with SSD and BD (except for asthma and cerebrovascular accident in BD).

We found differences for ethnicity in individuals with diabetes, hypertension, asthma, arthritis, epilepsy, eczema, ischaemic heart disease, COPD and cerebrovascular accident (Figure 2, Supplemental Tables 4). Individuals from Black or South Asian minorities were more likely to show higher prevalence rates of diabetes and hypertension compared to those White British or Irish. Black Caribbean showed the higher prevalence rates of asthma or eczema among all other groups. Arthritis, COPD, epilepsy and IHD seem to be slightly more prevalent in White British or Irish, with epilepsy showing the highest prevalence rates among Irish. Similar trends were found within SSD and BD subgroups with diabetes rates higher in South Asians with BD (29.7%) compared to South Asians with SSD (20.7%); while diabetes rates in Black Caribbean with SSD

For peer review only

(24.4%) were higher than in those with BD (19.6%). With regards to social deprivation, we found that individuals with diabetes, hypertension, asthma, and COPD were more likely to be higher levels of deprivation compared to those that did not have these specific conditions (Supplemental Table 5).

PLEASE INSERT FIGURE 2 AROUND HERE

Multimorbidity combinations for the whole cohort and SSD and BD subgroups

Table 3 summarizes the ten most common physical comorbidities in patients with SMI, their prevalence, the mean number of comorbidities, and the three most frequently associated comorbidities, for the total cohort and by SMI diagnosis. While there were no clear differences in the mean number of comorbidities by SMI diagnosis, the presence of one physical condition predisposed individuals to at least one other condition; the mean number of comorbidities in the total cohort was 0.74, jumping to at least 2.20 in the presence of one of the ten most common comorbidities. The three most commonly associated physical comorbidities remained relatively consistent by SMI diagnosis, with a few exceptions. The prevalence of associated comorbidities with epilepsy are lower in BD than SSD, a fairly different comorbidity profile in migraine between SMI diagnoses and a lower rate of diabetes in individuals with comorbid BD and COPD (when compared to SSD). The most common combination of conditions included diabetes, hypertension and asthma, regardless of their SMI diagnoses. Most individuals with these combinations of conditions were also likely to have arthritis. Figures 3, 4 and 5 show the most prevalent conditions for individuals with SMI and comorbid diabetes, hypertension and asthma, respectively.

PLEASE INSERT TABLE 3 AND FIGURES 3,4 AND 5 AROUND HERE

Association with disability

For peer review only - http://bmjopen.bmj.com/site/about/guidelines.xhtml

HoNOS descriptive statistics for the whole SMI cohort (Mean=10.40, SD=6.06) and the ten most common physical comorbidities are shown in Table 4. Regression analyses showed that individuals with any of these conditions (except migraine) showed higher HoNOS scores, even after adjustments for age, sex, IMD or SMI diagnoses. We also examined whether simple and complex multimorbidity was also associated with HoNOS total score and we found that a strong positive association minimally attenuated after adjustments. Similar socio-demographics and trends were found within BD and SSD groups (Supplemental Tables 6-8). However, associations for diabetes, hypertension and ischaemic heart disease were fully attenuated after adjustments in the SSD group and associations for hypertension were also fully attenuated after adjustments in the BD group.

PLEASE INSERT TABLE 4 AROUND HERE

Discussion

The first objective of this study was to design and develop a suitable strategy to extract physical health conditions which could be easily replicated in future multimorbidity research using mental health electronic health records. The NLP strategy using MedCAT provided very good performance estimates for all the conditions extracted, which supports its suitability to extract data on physical health conditions from mental health clinical notes. These findings are consistent with previous research which has used MedCAT to extract data from hospital settings.[34,35] This resource should help to facilitate and promote research on multimorbidity using mental health records, in general, and has the potential for direct replication in other mental health trusts that have already deployed CRIS platforms.

Our second objective was to examine the prevalence of these conditions in SMI individuals and compare the most prevalent multimorbidity combinations between individuals with BD and SSD.

For peer review only - http://bmjopen.bmj.com/site/about/guidelines.xhtml

When we examined differences in socio-demographic variables by diagnosis, our findings were largely consistent with previous research,[3,36–39] although associations between ethnicity and BD are less established.[40] With regards to sociodemographic differences, we found that women with SSD were more likely to have diabetes, CKD, heart failure and TIA compared to men with SSD; and women with BD were more likely to have asthma, arthritis and migraine compared to men with BD. Previous research in this population showed mixed results. Some studies found higher prevalence of hypertension in women with SSD[41] and diabetes in women in BD,[42] and others did not find relevant sex differences.[43] Our findings suggest that there could be an increased risk for diabetes and hypertension for females with an SMI diagnosis, especially in SSD. Further research in this line is needed.

Ethnic differences were found for diabetes, hypertension, asthma, arthritis, epilepsy, eczema, ischaemic heart disease, COPD and cerebrovascular accident. Individuals from Black or South Asian minorities were more likely to show higher prevalence rates of diabetes and hypertension compared to White British or Irish. Black Caribbean showed the higher prevalence rates of asthma or eczema among all other groups. Arthritis, COPD, epilepsy and IHD seem to be slightly more prevalent in White British or Irish, with epilepsy showing the highest prevalence rates among Irish. Similar trends were found within SSD and BD subgroups with diabetes rates higher in South Asians with BD compared to South Asians with SSD, while diabetes rates in Black Caribbean with SSD were higher than in those with BD. These results largely mirror previous research in ethnicity.[44–50] When we examined social deprivation, individuals with diabetes, hypertension, asthma, and COPD were more likely to report the highest levels of deprivation regardless of their SMI diagnoses. Similar to ethnicity, these results are also consistent with findings in the general

For peer review only - http://bmjopen.bmj.com/site/about/guidelines.xhtml

population where higher levels of social deprivation are found in those with comorbid diabetes,[51,52] hypertension,[53] asthma[54] or COPD.[55]

Overall, in the whole SMI cohort, around 40% of the individuals had at least one mention of a physical health condition and close to 20% had two or more physical conditions, which could be labelled as complex multimorbidity. These findings provide evidence to support previous research suggestions about the increased probability of multimorbidity in this population.[3,17,56–59] Absolute numbers of physical health conditions were higher in patients with SSD than those with BD. Although direct comparisons require caution, our findings partially contrast with previous reports of higher number of physical comorbidities in individuals with BD.[3,38,39,41] Overall, the top ten most prevalent conditions in our SMI cohort were diabetes, hypertension, asthma, arthritis, epilepsy, cerebrovascular accident, eczema, migraine, ischaemic heart disease and COPD; and the most common combination of conditions included diabetes, hypertension and asthma, regardless of their SMI diagnoses. Moreover, those that had complex multimorbidity were also more likely to have cardiometabolic comorbidities such as diabetes and hypertension, which suggests that the cardiometabolic pathway might be one of the key explanatory mechanism underlying the association between physical multimorbidity and severe mental illness.[60,61] Future research should explore further these potential independent contribution of this pathway when focusing on individuals with complex multimorbidity. Furthermore, arthritis was the most frequent subsequent comorbidity for those with diabetes, hypertension and/or asthma, however for those with SSD and asthma, eczema slightly displaced arthritis in terms of prevalence. These findings might suggest that potential differences between SSD and BD phenotypes could be linked to underlying inflammatory pathways. Future research focusing on inflammatory biomarkers could be key to further our understanding of the potential differences between SSD and BD.

In addition, we examined the association between the top ten most prevalent conditions and disability levels. We found that not only multimorbidity was clearly associated with higher levels of disability but having any of these specific conditions was associated with higher levels of disability, even after adjustments for age, sex, deprivation or SMI diagnoses. Similar results were found when we further examined the associations between multimorbidity and disability within SSD and BD groups. When we examined the independent association of each physical health condition and disability within groups, our results suggested that that socio-demographic factors could have a greater impact in these associations in individuals with SSD. Although our results are not directly comparable with previous studies, they are in line with findings in previous research in ageing[62] or some specific SMI populations.[63,64] Further research is needed to understand the potential shared drivers of disability in individuals with BD and these conditions, in general, and diabetes and BD, in particular.

One of the main strengths of this study is the large comprehensive cohort of people with SMI drawn from a population with a high ethnic diversity, addressing the neglect of both ethnic minority groups and SMI in multimorbidity research. This is a key advantage of using EHRs from a large secondary mental health care provider and having the benefits of a data extraction strategy for accessing data on physical health conditions from the text fields of clinical notes. MedCAT development and deployment in CRIS text records will hopefully promote and facilitate future research in mental healthcare. However, further research is needed to validate this strategy in other EHRs sources using free text. Although our results are promising and the comparability of the findings with previous research provides some evidence of validity, further research is also needed to examine the cross-validity using primary care structured fields data. Furthermore, it is also important to note that individuals with more severe SMI may have more comprehensive textual

For peer review only

data, so that our findings might be less representative of highly functioning individuals with less severe SMI. In addition, we acknowledge that although the conditions considered are within the most considered in multimorbidity research, future studies should consider a larger number of conditions and include rare diseases. It should be noted that our study is one of the first, to our knowledge, to compare the associations between physical health comorbidities and disability in this traditionally neglected population and HoNOS is a widely used measure in secondary mental health services in the UK which provides us a general overview of disability in this population. However, we acknowledge that further research with more objective measures of disability is also needed to drive future policy in this population. To sum up, our study provides an overview of the most prevalent health conditions in SMI and underlines the need for further research into the origins of multimorbidity in this population, considering in more detail the nature of the SMI both in terms of severity and in terms of constituent diagnoses and/or symptomatic phenotype, given the apparent differences between BD and SSD. Our findings highlight multimorbidity as a driver of disability in this population, which also requires further mechanistic evaluation.

For peer review only

Acknowledgments: We would like to thank Megan Pritchard and Mathew Broadbent for their unvaluable contribution and support.

Funding statement:

RB is funded in part by grant MR/R016372/1 for the King's College London MRC Skills Development Fellowship programme funded by the UK Medical Research Council (MRC) and by grant IS-BRC-1215-20018 for the National Institute for Health Research (NIHR) Biomedical Research Centre at South London and Maudsley NHS Foundation Trust and King's College London. JD is funded by the Health Foundation working together with the Academy of Medical Sciences, for a Clinician Scientist Fellowship and by the ESRC in relation to the SEP-MD study [ES/S002715/1] and part supported by the ESRC Centre for Society and Mental Health at King's College London [ESRC Reference: ES/S012567/1]. DB is funded by a UKRI Innovation Fellowship as part of Health Data Research UK MR/S00310X/. AM is funded by Takeda California, Inc. RD, RS, AR are part-funded by the National Institute for Health Research (NIHR) Biomedical Research Centre at South London and Maudsley NHS Foundation Trust and King's College London. RD's work is supported by 1.National Institute for Health Research (NIHR) Biomedical Research Centre at South London and Maudsley NHS Foundation Trust and King's College London. 2. Health Data Research UK, which is funded by the UK Medical Research Council, Engineering and Physical Sciences Research Council, Economic and Social Research Council, Department of Health and Social Care (England), Chief Scientist Office of the Scottish Government Health and Social Care Directorates, Health and Social Care Research and Development Division (Welsh Government), Public Health Agency (Northern Ireland), British Heart Foundation and Wellcome Trust. 3. The National Institute for Health Research University

For peer review only

College London Hospitals Biomedical Research Centre. This paper represents independent research funded by the National Institute for Health Research (NIHR) Biomedical Research Centre at South London and Maudsley NHS Foundation Trust and King's College London and by the Health Foundation. The views expressed are those of the author(s) and not necessarily those of the NHS, the NIHR, the Department of Health, the MRC, ESRC or King's College London.

Authors contributions: RB conceived and designed the study. ZK, RB and RS designed, validated the data extraction strategy. ZK, TS and AM developed the natural language processing algorithm and interface for the annotations. RB, ZK, JC, LM, NC, TS, AM, NS and TW were annotators. RB, ZK, SS, LL, JC, SA and DB performed the data analyses and interpreted the results. RS and JD provided clinically relevant input over all the stages. RB, ZK and SS drafted the first version of the manuscript and all authors critically reviewed the manuscript and contributed to writing the final version.

Data sharing statement: Due to the confidential nature of free-text data, we are unable to make patient-level data available. This project was approved by the CRIS Oversight Committee which is responsible for ensuring all research applications comply with ethical and legal guidelines. The CRIS system enables access to anonymised electronic patient records for secondary analysis from SLaM and has full ethical approvals. CRIS was developed with extensive involvement from service users and adheres to strict governance frameworks managed by service users. It has passed a robust ethics approval pro-cess acutely attentive to the use of patient data. Specifically, this system was approved as a dataset for secondary data analysis on this basis by Oxfordshire Research Ethics Committee C (08/H06060/71). The data is de-identified and used in a data-secure format

and all patients have the choice to opt-out of their anonymized data being used. Approval for data access can only be provided from the CRIS Oversight Committee at SLaM.

Competing interest statements: No competing interests.

References

1 Firth J, Rosenbaum S, Galletly C, *et al.* Protecting physical health in people with mental illness – Authors' reply. *Lancet Psychiatry* 2019;**6**:890–1. doi:10.1016/S2215-0366(19)30387-6

2 Lawrence D, Kisely S, Pais J. The Epidemiology of Excess Mortality in People with Mental Illness. *Can J Psychiatry* 2010;**55**:752–60. doi:10.1177/070674371005501202

3 Reilly S, Olier I, Planner C, *et al.* Inequalities in physical comorbidity: a longitudinal comparative cohort study of people with severe mental illness in the UK. *BMJ Open* 2015;**5**:e009010. doi:10.1136/bmjopen-2015-009010

4 Woodhead C, Ashworth M, Schofield P, *et al.* Patterns of physical co-/multi-morbidity among patients with serious mental illness: a London borough-based cross-sectional study. *BMC Fam Pract* 2014;**15**:117. doi:10.1186/1471-2296-15-117

5 Filipcic IŠ, Bajic Ž, Filipcic I. The onset and accumulation of physical multimorbidity in severe and common mental disorders. *Curr Opin Psychiatry* 2020;**33**:484–90. doi:10.1097/YCO.0000000000000635

6 Cheung KL, ten Klooster PM, Smit C, *et al.* The impact of non-response bias due to sampling in public health studies: A comparison of voluntary versus mandatory recruitment in a Dutch national survey on adolescent health. *BMC Public Health* 2017;**17**. doi:10.1186/s12889-017-4189-8

For peer review only

7 Correll CU, Detraux J, Lepeleire JD, *et al.* Effects of antipsychotics, antidepressants and mood stabilizers on risk for physical diseases in people with schizophrenia, depression and bipolar disorder. *World Psychiatry* 2015;**14**:119–36. doi:https://doi.org/10.1002/wps.20204

8 Correll CU, Solmi M, Veronese N, *et al.* Prevalence, incidence and mortality from cardiovascular disease in patients with pooled and specific severe mental illness: a large-scale meta-analysis of 3,211,768 patients and 113,383,368 controls. *World Psychiatry* 2017;**16**:163–80. doi:https://doi.org/10.1002/wps.20420

9 DE HERT M, CORRELL CU, BOBES J, *et al.* Physical illness in patients with severe mental disorders. I. Prevalence, impact of medications and disparities in health care. *World Psychiatry* 2011;**10**:52–77.

10 Fan Z, Wu Y, Shen J, *et al.* Schizophrenia and the risk of cardiovascular diseases: A meta-analysis of thirteen cohort studies. *J Psychiatr Res* 2013;**47**:1549–56. doi:10.1016/j.jpsychires.2013.07.011

11 Pouget JG, Schizophrenia Working Group of the Psychiatric Genomics Consortium, Han B, *et al.* Cross-disorder analysis of schizophrenia and 19 immune-mediated diseases identifies shared genetic risk. *Hum Mol Genet* 2019;**28**:3498–513. doi:10.1093/hmg/ddz145

12 Rosenblat JD, McIntyre RS. Are medical comorbid conditions of bipolar disorder due to immune dysfunction? *Acta Psychiatr Scand* 2015;**132**:180–91. doi:https://doi.org/10.1111/acps.12414

13 Tylee DS, Sun J, Hess JL, *et al.* Genetic correlations among psychiatric and immune-related phenotypes based on genome-wide association data. *Am J Med Genet B Neuropsychiatr Genet* 2018;**177**:641–57. doi:https://doi.org/10.1002/ajmg.b.32652

For peer review only

14 Leo RJ, Singh J. Migraine headache and bipolar disorder comorbidity: A systematic review of the literature and clinical implications. *Scand J Pain* 2016;**11**:136–45. doi:10.1016/j.sjpain.2015.12.002

15 McKay KA, Tremlett H, Fisk JD, *et al.* Psychiatric comorbidity is associated with disability progression in multiple sclerosis. *Neurology* 2018;**90**:e1316–23. doi:10.1212/WNL.0000000000005302

16 Dorrington S, Carr E, Stevelink SAM, *et al.* Multimorbidity and fit note receipt in working-age adults with long-term health conditions. *Psychol Med* undefined/ed;:1–10. doi:10.1017/S0033291720002937

17 Kugathasan P, Wu H, Gaughran F, *et al.* Association of physical health multimorbidity with mortality in people with schizophrenia spectrum disorders: Using a novel semantic search system that captures physical diseases in electronic patient records. *Schizophr Res* 2020;**216**:408–15. doi:10.1016/j.schres.2019.10.061

18 Barnett K, Mercer SW, Norbury M, *et al.* Epidemiology of multimorbidity and implications for health care, research, and medical education: a cross-sectional study. *The Lancet* 2012;**380**:37–43. doi:10.1016/S0140-6736(12)60240-2

19 Cassell A, Edwards D, Harshfield A, *et al.* The epidemiology of multimorbidity in primary care: a retrospective cohort study. *Br J Gen Pract* 2018;**68**:e245–51. doi:10.3399/bjgp18X695465

20 Kuan V, Denaxas S, Gonzalez-Izquierdo A, *et al.* A chronological map of 308 physical and mental health conditions from 4 million individuals in the English National Health Service. *Lancet Digit Health* 2019;**1**:e63–77. doi:10.1016/S2589-7500(19)30012-3

For peer review only

21 Altamura AC, Buoli M, Pozzoli S. Role of immunological factors in the pathophysiology and diagnosis of bipolar disorder: Comparison with schizophrenia. *Psychiatry Clin Neurosci* 2014;**68**:21–36. doi:https://doi.org/10.1111/pcn.12089

22 Sahu A, Chowdhury HA, Gaikwad M, *et al.* Integrative network analysis identifies differential regulation of neuroimmune system in Schizophrenia and Bipolar disorder. *Brain Behav Immun - Health* 2020;**2**:100023. doi:10.1016/j.bbih.2019.100023

23 Walker J, Curtis V, Shaw P, *et al.* Schizophrenia and bipolar disorder are distinguished mainly by differences in neurodevelopment. *Neurotox Res* 2002;**4**:427. doi:10.1080/1029842021000022070

24 WHO | The world health report 2001 - Mental Health: New Understanding, New Hope. WHO. https://www.who.int/whr/2001/en/ (accessed 4 Jun 2021).

25 Prediction of disability in schizophrenia: Symptoms, cognition, and self-assessment - Philip D. Harvey, Martin T. Strassnig, Juliet Silberstein, 2019. https://journals.sagepub.com/doi/full/10.1177/2043808719865693 (accessed 4 Jun 2021).

26 Sanchez-Moreno J, Martinez-Aran A, Tabarés-Seisdedos R, *et al.* Functioning and Disability in Bipolar Disorder: An Extensive Review. *Psychother Psychosom* 2009;**78**:285–97. doi:10.1159/000228249

27 Stewart R, Soremekun M, Perera G, *et al.* The South London and Maudsley NHS Foundation Trust Biomedical Research Centre (SLAM BRC) case register: development and descriptive data. *BMC Psychiatry* 2009;**9**:51. doi:10.1186/1471-244X-9-51

28 ICD-10 Version:2016. https://icd.who.int/browse10/2016/en (accessed 9 Jun 2021).

For peer review only

29 Kraljevic Z, Searle T, Shek A, *et al.* Multi-domain Clinical Natural Language Processing with MedCAT: the Medical Concept Annotation Toolkit. *ArXiv201001165 Cs* Published Online First: 25 March 2021.http://arxiv.org/abs/2010.01165 (accessed 4 Jun 2021).

30 Searle T, Kraljevic Z, Bendayan R, *et al.* MedCATTrainer: A Biomedical Free Text Annotation Interface with Active Learning and Research Use Case Specific Customisation. *ArXiv190707322 Cs* Published Online First: 16 July 2019.http://arxiv.org/abs/1907.07322 (accessed 19 Aug 2020).

31 English indices of deprivation 2019: technical report. GOV.UK. https://www.gov.uk/government/publications/english-indices-of-deprivation-2019-technical-report (accessed 4 Jun 2021).

32 Wing JK, Beevor AS, Curtis RH, *et al.* Health of the Nation Outcome Scales (HoNOS): Research and development. *Br J Psychiatry* 1998;**172**:11–8. doi:10.1192/bjp.172.1.11

33 Jewell A, Pritchard M, Barrett K, *et al.* The Maudsley Biomedical Research Centre (BRC) data linkage service user and carer advisory group: creating and sustaining a successful patient and public involvement group to guide research in a complex area. *Res Involv Engagem* 2019;**5**:20. doi:10.1186/s40900-019-0152-4

34 Bean DM, Kraljevic Z, Searle T, *et al.* Angiotensin-converting enzyme inhibitors and angiotensin II receptor blockers are not associated with severe COVID-19 infection in a multi-site UK acute hospital trust. *Eur J Heart Fail* 2020;**22**:967–74. doi:https://doi.org/10.1002/ejhf.1924

35 Carr E, Bendayan R, Bean D, *et al.* Evaluation and improvement of the National Early Warning Score (NEWS2) for COVID-19: a multi-hospital study. *BMC Med* 2021;**19**:23. doi:10.1186/s12916-020-01893-3

For peer review only

36 Birgenheir DG, Ilgen MA, Bohnert ASB, *et al.* Pain conditions among veterans with schizophrenia or bipolar disorder. *Gen Hosp Psychiatry* 2013;**35**:480–4. doi:10.1016/j.genhosppsych.2013.03.019

37 Hardoon S, Hayes JF, Blackburn R, *et al.* Recording of Severe Mental Illness in United Kingdom Primary Care, 2000–2010. *PLOS ONE* 2013;**8**:e82365. doi:10.1371/journal.pone.0082365

38 Smith DJ, Langan J, McLean G, *et al.* Schizophrenia is associated with excess multiple physical-health comorbidities but low levels of recorded cardiovascular disease in primary care: cross-sectional study. *BMJ Open* 2013;**3**:e002808. doi:10.1136/bmjopen-2013-002808

39 Smith DJ, Martin D, McLean G, *et al.* Multimorbidity in bipolar disorder and undertreatment of cardiovascular disease: a cross sectional study. *BMC Med* 2013;**11**:263. doi:10.1186/1741-7015-11-263

40 Halvorsrud K, Nazroo J, Otis M, *et al.* Ethnic inequalities in the incidence of diagnosis of severe mental illness in England: a systematic review and new meta-analyses for non-affective and affective psychoses. *Soc Psychiatry Psychiatr Epidemiol* 2019;**54**:1311–23. doi:10.1007/s00127-019-01758-y

41 Oreški I, Jakovljević M, Aukst-Margetić B, *et al.* Comorbidity and multimorbidity in patients with schizophrenia and bipolar disorder: similarities and differencies. *Psychiatr Danub* 2012;**24**:80–5.

42 Patel RS, Virani S, Saeed H, *et al.* Gender Differences and Comorbidities in U.S. Adults with Bipolar Disorder. *Brain Sci* 2018;**8**:168. doi:10.3390/brainsci8090168

43 Severe mental illness (SMI) and physical health inequalities: briefing. GOV.UK. https://www.gov.uk/government/publications/severe-mental-illness-smi-physical-health-

For peer review only

inequalities/severe-mental-illness-and-physical-health-inequalities-briefing (accessed 4 Jun 2021).

44 Das-Munshi J, Ashworth M, Dewey ME, *et al.* Type 2 diabetes mellitus in people with severe mental illness: inequalities by ethnicity and age. Cross-sectional analysis of 588 408 records from the UK. *Diabet Med* 2017;**34**:916–24. doi:https://doi.org/10.1111/dme.13298

45 Gaughran F, Stahl D, Stringer D, *et al.* Effect of lifestyle, medication and ethnicity on cardiometabolic risk in the year following the first episode of psychosis: prospective cohort study. *Br J Psychiatry* 2019;**215**:712–9. doi:10.1192/bjp.2019.159

46 Lusignan S de, Alexander H, Broderick C, *et al.* The epidemiology of eczema in children and adults in England: A population-based study using primary care data. *Clin Exp Allergy* 2021;**51**:471–82. doi:https://doi.org/10.1111/cea.13784

47 Gilkes A, Ashworth M, Schofield P, *et al.* Does COPD risk vary by ethnicity? A retrospective cross-sectional study. *Int J Chron Obstruct Pulmon Dis* 2016;**11**:739–46. doi:10.2147/COPD.S96391

48 Godsland IF, Johnston DG, Chaturvedi N. Mechanisms of Disease: lessons from ethnicity in the role of triglyceride metabolism in ischemic heart disease. *Nat Clin Pract Endocrinol Metab* 2007;**3**:530–8. doi:10.1038/ncpendmet0530

49 Health Survey for England - 2004: Health of ethnic minorities, Headline results. NHS Digit. https://digital.nhs.uk/data-and-information/publications/statistical/health-survey-for-england/health-survey-for-england-2004-health-of-ethnic-minorities-headline-results (accessed 4 Jun 2021).

50 Public Health Profiles - PHE. https://fingertips.phe.org.uk/ (accessed 4 Jun 2021).

For peer review only

51 Zghebi SS, Steinke DT, Carr MJ, *et al.* Examining trends in type 2 diabetes incidence, prevalence and mortality in the UK between 2004 and 2014. *Diabetes Obes Metab* 2017;**19**:1537–45. doi:https://doi.org/10.1111/dom.12964

52 Diabetes Prevention Programme, 2017-18 Diagnoses and Demographics. NHS Digit. https://digital.nhs.uk/data-and-information/publications/statistical/national-diabetes-audit/diabetes-prevention-programme-2017-18 (accessed 4 Jun 2021).

53 Leng B, Jin Y, Li G, *et al.* Socioeconomic status and hypertension: a meta-analysis. *J Hypertens* 2015;**33**:221–9. doi:10.1097/HJH.0000000000000428

54 Gupta RP, Mukherjee M, Sheikh A, *et al.* Persistent variations in national asthma mortality, hospital admissions and prevalence by socioeconomic status and region in England. *Thorax* 2018;**73**:706–12. doi:10.1136/thoraxjnl-2017-210714

55 Simpson CR, Hippisley-Cox J, Sheikh A. Trends in the epidemiology of chronic obstructive pulmonary disease in England: a national study of 51 804 patients. *Br J Gen Pract* 2010;**60**:e277–84. doi:10.3399/bjgp10X514729

56 Carney CP, Jones L, Woolson RF. Medical comorbidity in women and men with schizophrenia. *J Gen Intern Med* 2006;**21**:1133–7. doi:10.1111/j.1525-1497.2006.00563.x

57 Gabilondo A, Alonso-Moran E, Nuño-Solinis R, *et al.* Comorbidities with chronic physical conditions and gender profiles of illness in schizophrenia. Results from PREST, a new health dataset. *J Psychosom Res* 2017;**93**:102–9. doi:10.1016/j.jpsychores.2016.12.011

58 Kugathasan P, Stubbs B, Aagaard J, *et al.* Increased mortality from somatic multimorbidity in patients with schizophrenia: a Danish nationwide cohort study. *Acta Psychiatr Scand* 2019;**140**:340–8. doi:10.1111/acps.13076

For peer review only

59 Severe mental illness (SMI) and physical health inequalities: briefing. GOV.UK. https://www.gov.uk/government/publications/severe-mental-illness-smi-physical-health-inequalities/severe-mental-illness-and-physical-health-inequalities-briefing (accessed 3 Mar 2020).

60 Firth J, Siddiqi N, Koyanagi A, *et al.* The Lancet Psychiatry Commission: a blueprint for protecting physical health in people with mental illness. *Lancet Psychiatry* 2019;**6**:675–712. doi:10.1016/S2215-0366(19)30132-4

61 Henderson DC, Vincenzi B, Andrea NV, *et al.* Pathophysiological mechanisms of increased cardiometabolic risk in people with schizophrenia and other severe mental illnesses. *Lancet Psychiatry* 2015;**2**:452–64. doi:10.1016/S2215-0366(15)00115-7

62 Quiñones AR, Markwardt S, Botoseneanu A. Multimorbidity Combinations and Disability in Older Adults. *J Gerontol A Biol Sci Med Sci* 2016;**71**:823–30. doi:10.1093/gerona/glw035

63 Desalegn D, Girma S, Abdeta T. Quality of life and its association with current substance use, medication non-adherence and clinical factors of people with schizophrenia in Southwest Ethiopia: a hospital-based cross-sectional study. *Health Qual Life Outcomes* 2020;**18**. doi:10.1186/s12955-020-01340-0

64 McIntyre RS, Konarski JZ, Soczynska JK, *et al.* Medical Comorbidity in Bipolar Disorder: Implications for Functional Outcomes and Health Service Utilization. *Psychiatr Serv* 2006;**57**:1140–4. doi:10.1176/ps.2006.57.8.1140

For peer review only

Table 1. MedCAT performance F1, precision and recall estimates for each physical health conditions.

Physical Health Condition	F1	Precision	Recall
Diabetes mellitus	0.98	0.99	0.98
Heart failure	0.97	0.97	0.96
Ischemic heart disease	0.98	0.97	0.99
Hypertensive disorder, systemic arterial	0.97	0.97	0.96
Chronic obstructive lung disease	0.94	0.97	0.92
Asthma	1.00	1.00	1.00
Chronic kidney disease	1.00	1.00	0.99
Cerebrovascular accident	0.96	0.94	0.98
Transient ischemic attack	0.91	0.82	1.00
Parkinson's disease	0.94	0.88	1.00
Multiple sclerosis	1.00	1.00	1.00
Epilepsy	0.93	1.00	0.85
Migraine	1.00	1.00	1.00
Atrial fibrillation	0.98	1.00	0.96
Chronic sinusitis	0.98	0.97	1.00
Inflammatory bowel disease	0.96	1.00	0.92
Chronic liver disease	1.00	1.00	1.00
Psoriasis	1.00	1.00	1.00
Eczema	0.94	1.00	0.88
Arthritis	1.00	1.00	1.00

For peer review only

Table 2. Socio-demographic characteristics and prevalence for physical health conditions for total cohort (N=17500) and by SMI diagnosis.

	Total	SSD	BD
**N (%)**	17500	13019 (74.4)	4481 (25.6)
**Sex*** **			
Female	8123 (46.4)	5421 (41.6)	2702 (60.3)
Male	9374 (53.6)	7596 (58.3)	1778 (39.7)
**Age at first SMI diagnosis*** **			
15 – 34	7497 (42.8)	5607 (43.1)	1890 (42.2)
35 – 44	3736 (21.3)	2792 (21.4)	944 (21.1)
45 – 54	2783 (15.9)	2057 (15.8)	726 (16.2)
55 – 64	1525 (8.7)	1067 (8.2)	458 (10.2)
65+	1959 (11.2)	1496 (11.5)	463 (10.3)
**Ethnicity*** **			
White British	6243 (35.7)	4008 (30.8)	2235 (49.9)
Black Caribbean	3182 (18.2)	2799 (21.5)	383 (8.5)
Black African	2094 (12.0)	1886 (14.5)	208 (4.6)
South Asian	549 (3.1)	421 (3.2)	128 (2.9)
Irish	346 (2.0)	240 (1.8)	106 (2.4)
Other	3846 (22.0)	2822 (21.7)	1024 (22.9)
Not stated	1240 (7.1)	843 (6.5)	397 (8.9)
**Index of multiple deprivation*** **			
1 (less deprived)	742 (4.2)	412 (3.2)	330 (7.4)
2	1384 (7.9)	887 (6.8)	497 (11.1)
3	3575 (20.4)	2503 (19.2)	1072 (23.9)
4	7073 (40.4)	5476 (42.1)	1597 (35.6)
5 (more deprived)	4033 (23.0)	3204 (24.6)	829 (18.5)
Unknown	693 (4.0)	537 (4.1)	156 (3.5)
**Number of conditions*** **			
No mentions	10468 (59.8)	7540 (57.9)	2928 (65.3)
One	3733 (21.3)	2888 (22.2)	845 (18.9)
Two	1795 (10.3)	1429 (11.0)	366 (8.2)
Three or more	1504 (8.6)	1162 (8.9)	342 (7.6)
**Physical conditions*** **			
Diabetes***	2686 (15.3)	2208 (17.0)	478 (10.7)
Hypertension***	2537 (14.5)	2070 (15.9)	467 (10.4)
Asthma	1722 (9.8)	1291 (9.9)	431 (9.6)
Arthritis	954 (5.5)	702 (5.4)	252 (5.6)
Epilepsy***	799 (4.6)	652 (5.0)	147 (3.3)
Cerebrovascular accident	728 (4.2)	573 (4.4)	155 (3.5)
Eczema	616 (3.5)	479 (3.7)	137 (3.1)

For peer review only

Migraine***	564 (3.2)	372 (2.9)	192 (4.3)
Ischemic heart disease	561 (3.2)	435 (3.3)	126 (2.8)
Chronic Obstructive Pulmonary disease	476 (2.7)	342 (2.6)	134 (3.0)
Chronic kidney disease*	279 (1.6)	179 (1.4)	100 (2.2)
Parkinson's disease	266 (1.5)	201 (1.5)	65 (1.5)
Heart failure**	222 (1.3)	187 (1.4)	35 (0.8)
Psoriasis	179 (1.0)	129 (1.0)	50 (1.1)
Atrial fibrillation	133 (0.8)	100 (0.8)	33 (0.7)
Transient Ischaemic Attack	130 (0.7)	91 (0.7)	39 (0.9)
Inflammatory Bowel Disease	40 (0.2)	25 (0.2)	15 (0.3)
Multiple sclerosis	32 (0.2)	19 (0.1)	13 (0.3)
Chronic liver disease	22 (0.1)	20 (0.2)	2 (0.0)
Chronic sinusitis	6 (0.0)	6 (0.0)	0 (0.0)

Note. *** $p < .001$; ** $p < .01$; * $p < .05$ for comparisons between BD and SSD groups

For peer review only

Table 3. Ten most prevalent conditions and associated comorbidities of the total cohort and by SMI diagnosis.

Condition		Preva-lence	Mean # of comorbidities	Three most frequent associated comorbidities		
	Total	-	0.74	1. Diabetes (15.3%)	2. HTN (14.5%)	3. Asthma (9.8%)
	SSD	-	0.77	1. Diabetes (17.0%)	2. HTN (15.9%)	3. Asthma (9.9%)
	BD	-	0.64	1. Diabetes (10.7%)	2. HTN (10.4%)	3. Asthma (9.6%)
**Diabetes**	*Total*	15.3%	2.35	1. HTN (42.1%)	2. Asthma (16.9%)	3. Arthritis (13.2%)
	SSD	17.0%	2.33	1. HTN (42.9%)	2. Asthma (16.3%)	3. Arthritis (12.7%)
	BD	10.7%	2.45	1. HTN (38.3%)	2. Asthma (19.5%)	3. Arthritis (15.5%)
**Hypertension**	*Total*	14.5%	2.50	1. Diabetes (44.5%)	2. Asthma (16.3%)	3. Arthritis (15.2%)
	SSD	15.9%	2.47	1. Diabetes (45.7%)	2. Asthma (16.1%)	3. Arthritis (14.7%)
	BD	10.4%	2.62	1. Diabetes (39.2%)	2. Arthritis (17.3 %)	3. Asthma (17.1%)
**Asthma**	*Total*	9.8%	2.27	1. Diabetes (26.3%)	2. HTN (24.0%)	3. Arthritis (11.9%)
	SSD	9.9%	2.27	1. Diabetes (27.9%)	2. HTN (25.9%)	3. Eczema (11.2%)
	BD	9.6%	2.29	1. Diabetes (21.6%)	2. HTN (18.6%)	3. Arthritis (15.1%)
**Arthritis**	*Total*	5.5%	2.78	1. HTN (40.5%)	2. Diabetes (37.1%)	3. Asthma (21.5%)
	SSD	5.4%	2.78	1. HTN (43.4%)	2. Diabetes (39.9%)	3. Asthma (19.9%)
	BD	5.6%	2.80	1. HTN (32.1%)	2. Diabetes (29.4%)	3. Asthma (25.8%)
**Epilepsy**	*Total*	4.6%	2.40	1. HTN (25.5%)	2. Diabetes (24.8%)	3. Asthma (21.4%)
	SSD	5.0%	2.42	1. HTN (27.3%)	2. Diabetes (26.4%)	3. Asthma (21.8%)
	BD	3.3%	2.31	1. Asthma (19.7%)	2. Diabetes (17.7%)	2. HTN (17.7%)
**CVA**	*Total*	4.2%	2.89	1. HTN (42.3%)	2. Diabetes (38.6%)	3. Asthma (15.4%)
	SSD	4.4%	2.83	1. HTN (42.8%)	2. Diabetes (38.9%)	3. Asthma (14.0%)
	BD	3.5%	3.09	1. HTN (40.6%)	2. Diabetes (37.4%)	3. Asthma (21.9)
**Eczema**	*Total*	3.5%	2.50	1. Asthma (32.5%)	2. Diabetes (26.9%)	3. HTN (23.1%)
	SSD	3.7%	2.45	1. Asthma (30.3%)	2. Diabetes (28.4%)	3. HTN (22.5%)
	BD	3.1%	2.64	1. Asthma (40.1%)	2. HTN (24.8%)	3. Diabetes (21.9%)
**Migraine**	*Total*	3.2%	2.20	1. Asthma (23.6%)	2. Diabetes (20.0%)	3. HTN (18.4%)
	SSD	2.9%	2.23	1. Diabetes (23.4%)	2. Asthma (21.5%)	3. HTN (20.2%)
	BD	4.3%	2.15	1. Asthma (27.6%)	2. HTN (15.1%)	3. Arthritis (14.1%)
**Ischaemic heart disease**	*Total*	3.2%	3.27	1. HTN (49.0%)	2. Diabetes (43.3%)	3. Asthma (20.3%)
	SSD	3.3%	3.28	1. HTN (51.0%)	2. Diabetes (44.6%)	3. Asthma (20.2%)
	BD	2.8%	3.24	1. HTN (42.1%)	2. Diabetes (38.9%)	3. Arthritis (22.2%)
**COPD**	*Total*	2.7%	3.22	1. HTN (39.7%)	2. Diabetes (38.9%)	3. Asthma (35.7%)
	SSD	2.6%	3.20	1. Diabetes (41.2%)	2. HTN (39.2%)	3. Asthma (35.7%)
	BD	3.0%	3.25	1. HTN (41.0%)	2. Asthma (35.8%)	3. Diabetes (32.8%)

Note. SSD: Schizophrenia Spectrum disorders. BD: Bipolar disorders. HTN: hypertension

Table 4. Associations between specific comorbidities, multimorbidity and HoNOS scores. Models were adjusted by age and sex (Model 1), and then additionally adjusted by IMD (Model 2a) or SMI diagnosis (Model 2b).

	HoNOS	Unadjusted	Model 1	Model 2a	Model 2b
	**Mean (SD)**	**B (95% CI)**	**B (95% CI)**	**B (95% CI)**	**B (95% CI)**
**Physical comorbidities**					
**Diabetes**	10.93 (6.08)	0.652 (0.389 – 0.914)***	0.464 (0.198 – 0.730)***	0.485 (0.215 – 0.755)***	0.359 (0.094 – 0.625)**
Ref: No diabetes	10.28 (6.05)				
**Hypertension**	10.99 (6.02)	0.713 (0.446 – 0.980)***	0.401 (0.123 – 0.680)**	0.368 (0.084 – 0.652)*	0.297 (0.019 – 0.576)*
Ref: No hypertension	10.27 (6.06)				
**Asthma**	11.05 (6.16)	0.734 (0.417 – 1.051)***	0.806 (0.490 – 1.121)***	0.770 (0.450 – 1.091)***	0.804 (0.489 – 1.118)***
Ref: No asthma	10.31 (6.04)				
**Arthritis**	12.01 (6.19)	1.728 (1.322 – 2.134)***	1.570 (1.156 – 1.984)***	1.558 (1.142 – 1.974)***	1.567 (1.155 – 1.980)***
Ref: No arthritis	10.28 (6.03)				
**Epilepsy**	11.40 (6.19)	1.055 (0.596 – 1.514)***	1.066 (0.609 – 1.523)***	1.089 (0.628 – 1.549)***	0.982 (0.527 – 1.437)***
Ref: No epilepsy	10.35 (6.05)				
**CVA**	11.81 (6.22)	1.487 (1.022 – 1.951)***	1.195 (0.728 – 1.662)***	1.180 (0.712 – 1.649)***	1.162 (0.697 – 1.627)***
Ref: No CVA	10.33 (6.04)				
**Eczema**	11.12 (6.19)	0.753 (0.249 – 1.257)**	0.838 (0.336 – 1.340)***	0.728 (0.220 – 1.237)**	0.799 (0.299 – 1.299)**
Ref: No eczema	10.37 (6.05)				
**Migraine**	10.33 (5.73)	-0.078 (-0.600 – 0.445)	0.212 (-0.311 – 0.734)	0.227 (-0.300 – 0.754)	0.302 (-0.218 – 0.823)
Ref: No migraine	10.40 (6.07)				
**Ischaemic heart disease**	11.64 (5.92)	1.294 (0.766 – 1.822)***	0.864 (0.332 – 1.395)***	0.853 (0.318 – 1.387)**	0.849 (0.320 – 1.379)**
Ref: No ischaemic heart disease	10.35 (6.06)				
**COPD**	12.08 (5.78)	1.733 (1.158 – 2.309)***	1.326 (0.745 – 1.908)***	1.336 (0.750 – 1.923)***	1.397 (0.818 – 1.977)***
Ref: No COPD	10.34 (6.06)				
**Multimorbidity**					

**Number of comorbidities**		0.487 (0.406 – 0.567)***	0.423 (0.339 – 0.507)***	0.424 (0.339 – 0.510) ***	0.404 (0.320 – 0.488)***
**1 or more comorbidities**	10.96 (6.08)	1.053 (0.850 – 1.256)***	0.893 (0.685 – 1.101)***	0.895 (0.681 – 1.109)***	0.823 (0.615 – 1.031)***
Ref: No comorbidities	9.90 (5.99)				
**2 or more comorbidities**	11.34 (6.13)	1.214 (0.973 – 1.455)***	1.021 (0.772 – 1.269)***	1.020 (0.768 – 1.272)***	0.968 (0.720 – 1.216)***
Ref: Less than 1 comorbidities	10.12 (6.01)				

Note. *** $p < .001$; ** $p < .01$; * $p < .05$

For peer review only

Figure 1. Comparison of number of physical health comorbidities (a) and specific physical comorbidities (b) by SMI diagnosis.

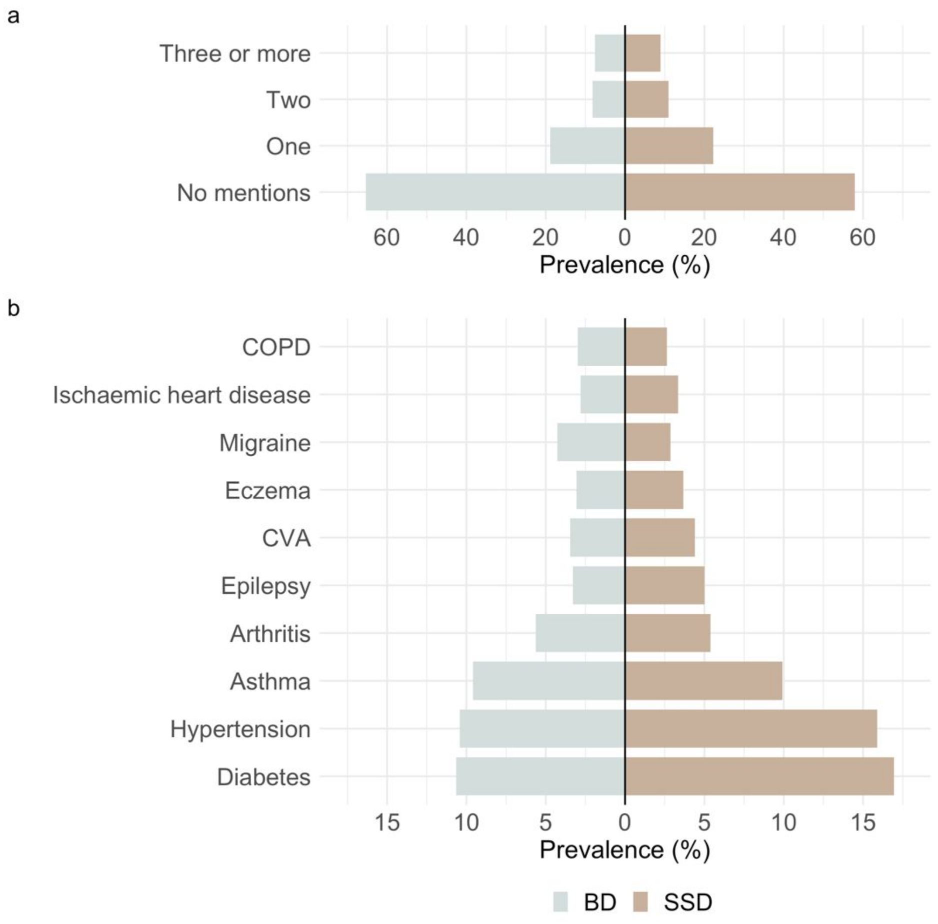

only

Figure 2. Prevalence of the most prevalent physical health conditions across ethnicities within the SMI cohort and the SSD and BD subgroups.

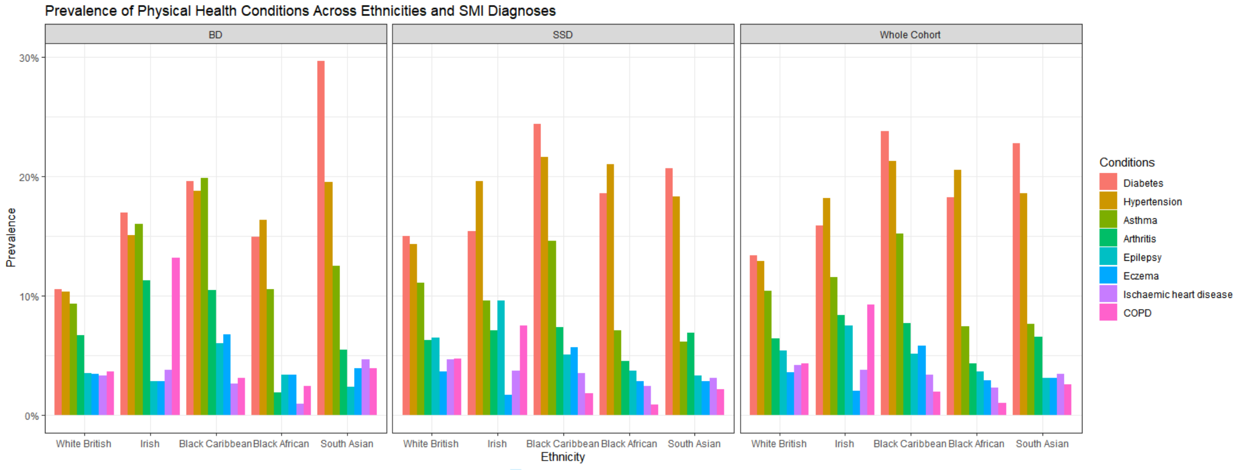

For peer review only

Figure 3. Visualization of most prevalent comorbidities in individuals with SMI and comorbid diabetes.

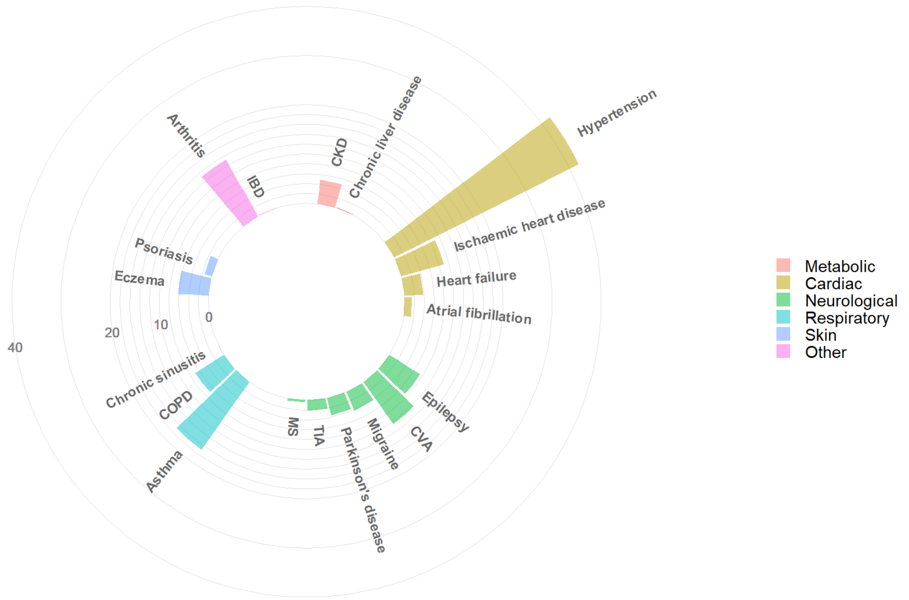

For peer review only - http://bmjopen.bmj.com/site/about/guidelines.xhtml

Figure 4. Visualization of most prevalent comorbidities in individuals with SMI and comorbid hypertension.

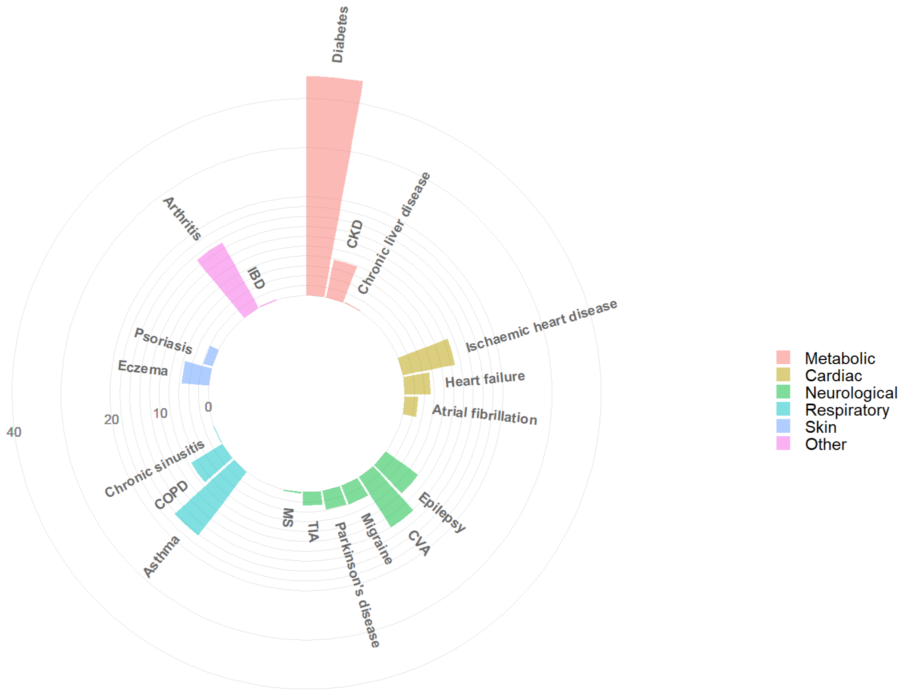

iew only

Figure 5. Visualization of most prevalent comorbidities in individuals with SMI and comorbid asthma.

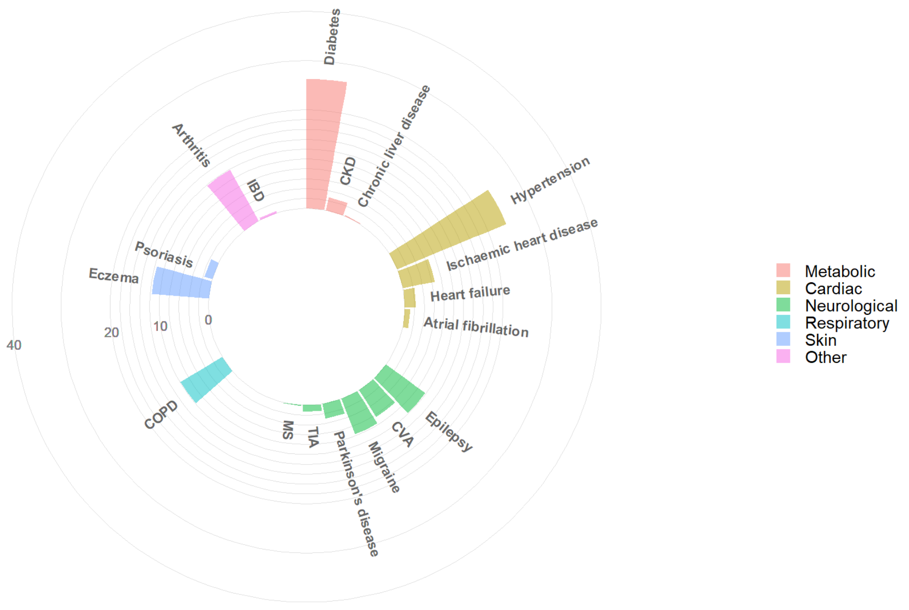

For peer review only

Supplemental Material

Supplemental Tables

Supplemental Table 1. Meta-annotations performance results for Diagnosis and Status

Supplemental Table 2. Prevalence estimates and sex differences within the whole SMI cohort and SSD and BD subgroups.

Supplemental Table 3. Prevalence for each condition by age ranges at first SMI diagnosis for the whole SMI cohort and SSD and BD subgroups.

Supplemental Tables 4a, 4b and 4c. Prevalence for each condition across ethnicities for the whole SMI cohort and SSD and BD subgroups.

Supplemental Table 5. Social deprivation prevalence for each condition for the whole cohort and within SSD and BD.

Supplemental Table 6. Sociodemographic features of cohort in HoNOS subsample analysis

Supplemental Table 7. Associations between comorbidities and HoNOS scores in SSD.

Supplemental Table 8. Associations between comorbidities and HoNOS scores in BD.

Supplemental Figures

Supplemental Figure 1. Distribution of all conditions in the SMI cohort by SMI group.

Supplemental Figure 2. Prevalence rates for age at SMI diagnoses per condition and comparison between individuals with BD and SSD.

Appendix 1. SNOMED Container and Concept Level Groupings for physical health conditions included in this study.

For peer review only

Supplemental Tables

Supplemental Table 1. Meta-annotations performance results for Diagnosis and Status

Meta-Annotation	Values	F1 (macro/weighted)	P (macro/weighted)	R (macro/weighted)
Diagnosis	Patient / Other	0.94 / 0.94	0.95 / 0.95	0.92 / 0.94
Status	Affirmed / Other	0.89 / 0.98	0.94 / 0.98	0.85 / 0.98

For peer review only

Supplemental Table 2. Prevalence estimates and sex differences within the whole SMI cohort and SSD and BD subgroups.

	Total Cohort		SSD			BD		
	Female	Male	Female	Male	p-value	Female	Male	p- value
**Diabetes**	1282 (15.8)	1403 (15)	1021 (18.8)	1187 (15.6)	<.001	261 (9.7)	216 (12.1)	.190
**Hypertension *****	1325 (16.3)	1212 (12.9)	1060 (19.6)	1010 (13.3)	<.001	265 (9.8)	202 (11.4)	1.000
**Asthma ****	869 (10.7)	852 (9.1)	559 (10.3)	732 (9.6)	1.000	310 (11.5)	120 (6.7)	<.001
**Arthritis *****	657 (8.1)	297 (3.2)	471 (8.7)	231 (3.0)	<.001	186 (6.9)	66 (3.7)	<.001
**Epilepsy**	349 (4.3)	450 (4.8)	253 (4.7)	399 (5.3)	1.000	96 (3.6)	51 (2.9)	1.000
**Cerebrovascular diseases**	367 (4.5)	361 (3.9)	265 (4.9)	308 (4.1)	.499	102 (3.8)	53 (3.0)	1.000
**Eczema ***	331 (4.1)	285 (3.0)	238 (4.4)	241 (3.2)	<.01	93 (3.4)	44 (2.5)	1.000
**Migraine *****	369 (4.5)	194 (2.1)	217 (4.0)	155 (2.0)	<.001	152 (5.6)	39 (2.2)	<.001
**Ischaemic heart disease**	249 (3.1)	312 (3.3)	196 (3.6)	239 (3.1)	1.000	53 (2.0)	73 (4.2)	<.001
**Chronic Obstructive Lung diseases**	237 (2.9)	239 (2.5)	153 (2.8)	189 (2.5)	1.000	84 (3.1)	50 (2.8)	1.000
**Chronic Kidney disease**	158 (1.9)	121 (1.3)	104 (1.9)	75 (1.0)	<.001	54 (2.0)	46 (2.6)	1.000
**Parkinson disease**	113 (1.4)	153 (1.6)	81 (1.5)	120 (1.6)	1.000	32 (1.2)	33 (1.9)	1.000
**Heart failure**	123 (1.5)	99 (1.0)	105 (1.9)	82 (1.1)	.001	18 (0.7)	17 (1.0)	1.000
**Psoriasis**	76 (0.9)	103 (1.1)	45 (0.8)	84 (1.1)	1.000	31 (1.1)	19 (1.1)	1.000
**Atrial fibrillation**	65 (0.8)	68 (0.7)	50 (0.9)	50 (0.7)	1.000	15 (0.6)	18 (1.0)	1.000
**Transient Ischaemic Attack**	79 (1.0)	51 (0.5)	53 (1.0)	38 (0.5)	.037	26 (1.0)	13 (0.7)	1.000
**Inflammatory Bowel diseases**	26 (0.3)	14 (0.1)	18 (0.3)	7 (0.1)	.080	8 (0.3)	7 (0.4)	1.000

For peer review only

**Multiple Sclerosis**	17 (0.2)	15 (0.2)	9 (0.2)	10 (0.1)	-	8 (0.3)	5 (0.3)	-
**Chronic liver disease**	10 (0.1)	12 (0.1)	9	11 (0.1)	-	1 (0.0)	1 (0.1)	-
**Chronic Sinusitis**	3 (0.0)	3 (0.0)	3	3 (0.0)	-	0 (0.0)	0 (0.0)	-

Note. *** p < .001; ** p < .01; * p < .05 for comparisons between male and female groups in the whole SMI cohort

For peer review only

Supplemental Table 3. Prevalence for each condition for the whole cohort and diagnoses subgroups and across age ranges at first SMI diagnosis.

For peer review only

	Total					SSD						BD					
	15-34	35-44	45-54	55-64	65+	15-34	35-44	45-54	55-64	65+		15-34	35-44	45-54	55-64	65+	
**Total**	7497	3736	2783	1525	1959	5607	2792	2057	1067	1496		1890	944	726	458	463	
**Diabetes*** **	697 (9.3)	559 (15.0)	531 (19.1)	371 (24.3)	528 (27.0)	591 (10.5)	479 (17.2)	439 (21.3)	284 (26.6)	415 (27.7)	p < 0.001	106 (5.6)	80 (8.5)	92 (12.7)	87 (19.0)	113 (24.4)	p < 0.001
**Hypertension*** **	472 (6.3)	426 (11.4)	514 (18.5)	416 (27.)3	709 (36.2)	412 (7.3)	372 (13.3)	411 (20.0)	313 (29.3)	562 (37.6)	p < 0.001	60 (3.2)	54 (5.7)	103 (14.2)	103 (22.5)	147 (31.7)	p < 0.001
**Asthma*** **	820 (10.9)	347 (9.3)	297 (10.7)	126 (8.3)	132 (6.7)	635 (11.3)	248 (8.9)	218 (10.6)	89 (8.3)	101 (6.8)	p < 0.001	185 (9.8)	99 (10.5)	79 (10.9)	37 (8.1)	31 (6.7)	p = 1.000
**Arthritis*** **	125 (1.7)	154 (4.1)	229 (8.2)	167 (11.0)	279 (14.2)	93 (1.7)	120 (4.3)	159 (7.7)	118 (11.1)	212 (14.2)	p < 0.001	32 (1.7)	34 (3.6)	70 (9.6)	49 (10.7)	67 (14.5)	p < 0.001
**Epilepsy**	339 (4.5)	188 (5.0)	133 (4.8)	67 (4.4)	72 (3.7)	276 (4.9)	154 (5.5)	112 (5.4)	55 (5.2)	55 (3.7)	1.000	63 (3.3)	34 (3.6)	21 (2.9)	12 (2.6)	17 (3.7)	1.000
**CVA*** **	166 (2.2)	112 (3.0)	122 (4.4)	114 (7.5)	214 (10.9)	143 (2.6)	87 (3.1)	95 (4.6)	84 (7.9)	164 (11.0)	p < 0.001	23 (1.2)	25 (2.6)	27 (3.7)	30 (6.6)	50 (10.8)	p < 0.001
**Eczema**	310 (4.1)	115 (3.1)	79 (2.8)	50 (3.3)	62 (3.2)	244 (4.4)	88 (3.2)	58 (2.8)	37 (3.5)	52 (3.5)	0.120	66 (3.5)	27 (2.9)	21 (2.9)	13 (2.8)	10 (2.2)	1.000
**Migraine*** **	300 (4.0)	122 (3.3)	87 (3.1)	32 (2.1)	23 (1.2)	207 (3.7)	73 (2.6)	53 (2.6)	24 (2.2)	15 (1.0)	p < 0.001	93 (4.9)	49 (5.2)	34 (4.7)	8 (1.7)	8 (1.7)	0.011
**Isc heart disease*** **	111 (1.5)	73 (2.0)	105 (3.8)	95 (6.2)	177 (9.0)	96 (1.7)	60 (2.1)	80 (3.9)	65 (6.1)	134 (9.0)	p < 0.001	15 (0.8)	13 (1.4)	25 (3.4)	30 (6.6)	43 (9.3)	p < 0.001
**COPD*** **	39 (0.5)	58 (1.6)	119 (4.3)	115 (7.5)	145 (7.4)	29 (0.5)	40 (1.4)	87 (4.2)	82 (7.7)	104 (7.0)	p < 0.001	10 (0.5)	18 (1.9)	32 (4.4)	33 (7.2)	41 (8.9)	p < 0.001
**CKD*** **	12 (0.2)	18 (0.5)	41 (1.5)	55 (3.6)	153 (7.8)	9 (0.2)	14 (0.5)	28 (1.4)	33 (3.1)	95 (6.4)	p < 0.001	3 (0.2)	4 (0.4)	13 (1.8)	22 (4.8)	58 (12.5)	-
**PD*** **	70 (0.9)	33 (0.9)	37 (1.3)	41 (2.7)	85 (4.3)	57 (1.0)	22 (0.8)	27 (1.3)	32 (3.0)	63 (4.2)	p < 0.001	13 (0.7)	11 (1.2)	10 (1.4)	9 (2.0)	22 (4.8)	p < 0.001
**HF*** **	18 (0.2)	30 (0.8)	42 (1.5)	40 (2.6)	92 (4.7)	17 (0.3)	30 (1.1)	38 (1.8)	29 (2.7)	73 (4.9)	p < 0.001	1 (0.1)	0 (0.0)	4 (0.6)	11 (2.4)	19 (4.1)	-
**Psoriasis**	67 (0.9)	33 (0.9)	37 (1.3)	17 (1.1)	25 (1.3)	54 (1.0)	25 (0.9)	25 (1.2)	9 (0.8)	16 (1.1)	1.000	13 (0.7)	8 (0.8)	12 (1.7)	8 (1.7)	9 (1.9)	0.450
**Atrial fibrillation*** **	14 (0.2)	7 (0.2)	12 (0.4)	19 (1.2)	81 (4.1)	11 (0.2)	6 (0.2)	8 (0.4)	12 (0.1)	63 (4.2)	p < 0.001	3 (0.20	1 (0.1)	4 (0.6)	7 (1.5)	18 (3.9)	-
**TIA*** **	20 (0.3)	18 (0.5)	19 (0.7)	23 (1.5)	50 (2.6)	15 (0.3)	15 (0.5)	13 (0.6)	14 (1.3)	34 (2.3)	p < 0.001	5 (0.3)	3 (0.3)	6 (0.8)	9 (2.0)	16 (3.5)	-
**IBD**	9 (0.1)	8 (0.2)	12 (0.4)	6 (0.4)	5 (0.3)	4 (0.1)	6 (0.2)	7 (0.3)	3 (0.3)	5 (0.3)	-	5 (0.3)	2 (0.2)	5 (0.7)	3 (0.7)	0 (0.0)	-
**MS**	6 (0.1)	8 (0.2)	11 (0.4)	5 (0.3)	2 (0.1)	4 (0.1)	4 (0.1)	8 (0.4)	3 (0.3)	0 (0.0)	-	2 (0.1)	4 (0.4)	3 (0.4)	2 (0.4)	2 (0.4)	-

For peer review only

**Chronic liver disease**	5 (0.1)	7 (0.2)	6 (0.2)	3 (0.2)	1 (0.1)	5 (0.1)	7 (0.3)	5 (0.2)	2 (0.2)	1 (0.1)	-	0 (0.0)	0 (0.0)	1 (0.1)	1 (0.2)	0 (0.0)	-
**Chronic sinusitis**	5 (0.1)	0 (0.0)	1 (0.0)	0 (0.0)	0 (0.0)	5 (0.1)	0 (0.0)	1 (0.0)	0 (0.0)	0 (0.0)	-	0 (0.0)	0 (0.0)	0 (0.0)	0 (0.0)	0 (0.0)	-

Note. *** $p < .001$; ** $p < .01$; * $p < .05$ for comparisons between age groups within the SMI cohort.

For peer review only

For peer review only - http://bmjopen.bmj.com/site/about/guidelines.xhtml

Supplemental Tables 4. Prevalence for each condition for the whole cohort and diagnoses subgroups and across ethnicities

Table 4a. Prevalence for each condition for the whole cohort and ethnicity.

	White British n=6243 (35.7%)	Irish n=346 (2.0%)	Black Caribbean n=3182 (18.2%)	Black African n=2094 (12.0%)	South Asian n=549 (3.1%)	Other[§] n=2846 (22.0%)	Unknown[§] n=1240 (7.1%)	Statistics
**Diabetes**	837 (13.4)	55 (15.9)	757 (23.8)	382 (18.2)	125 (22.8)	461 (12.0)	69 (5.6)	χ^2 (4) = 172.49; *p* <0.001
**Hypertension**	804 (12.9)	63 (18.2)	678 (21.3)	430 (20.5)	102 (18.6)	400 (10.4)	60 (4.8)	χ^2 (4) = 137.98; *p* <0.001
**Asthma**	651 (10.4)	40 (11.6)	484 (15.2)	156 (7.4)	42 (7.7)	296 (7.7)	53 (4.3)	χ^2 (4) = 92.58; *p* <0.001
**Arthritis**	402 (6.4)	29 (8.4)	246 (7.7)	90 (4.3)	36 (6.6)	139 (3.6)	12 (1.0)	χ^2 (4) = 26.80; *p* <0.001
**Epilepsy**	339 (5.4)	26 (7.5)	164 (5.2)	77 (3.7)	17 (3.1)	147 (3.8)	29 (2.4)	χ^2 (4) = 19.02; *p* = 0.010
**Cerebrovascular accident**	274 (4.4)	20 (5.8)	184 (5.8)	92 (4.4)	25 (4.6)	116 (3.0)	17 (1.4)	χ^2 (4) = 10.60; *p* = 0.408
**Eczema**	222 (3.6)	7 (2.0)	185 (5.8)	61 (2.9)	17 (3.1)	108 (2.8)	16 (1.3)	χ^2 (4) = 41.93; *p* <0.001
**Migraine**	235 (3.8)	12 (3.5)	129 (4.1)	61 (2.9)	17 (3.1)	98 (2.5)	12 (1.0)	χ^2 (4) = 5.44; *p* >.99
**Ischemic heart disease**	261 (4.2)	13 (3.8)	108 (3.4)	48 (2.3)	19 (3.5)	99 (2.6)	13 (1.0)	χ^2 (4) = 16.74; *p* = 0.028
**COPD**	271 (4.3)	32 (9.2)	63 (2.0)	21 (1.0)	14 (2.6)	60 (1.6)	15 (1.2)	χ^2 (4) = 114.69; *p* <0.001
**CKD**	125 (2.0)	9 (2.6)	56 (1.8)	32 (1.5)	13 (2.4)	37 (1.0)	7 (0.6)	χ^2 (4) = 3.81; *p* >.99
**Parkinson's disease**	117 (1.9)	6 (1.7)	56 (1.8)	25 (1.2)	11 (2.0)	45 (1.2)	6 (0.5)	χ^2 (4) = 4.56; *p* >.99
**Heart failure**	88 (1.4)	8 (2.3)	55 (1.7)	29 (1.4)	8 (1.5)	31 (0.8)	3 (0.2)	χ^2 (4) = 3.16; *p* >.99
**Psoriasis**	103 (1.6)	4 (1.2)	12 (0.4)	6 (0.3)	5 (0.9)	44 (1.1)	5 (0.4)	*
**Atrial fibrillation**	69 (1.1)	9 (2.6)	23 (0.7)	11 (0.5)	2 (0.4)	15 (0.4)	4 (0.3)	*

For peer review only

**TIA**	58 (0.9)	7 (2.0)	24 (0.8)	15 (0.7)	7 (1.3)	13 (0.3)	6 (0.5)	*
**IBD**	24 (0.4)	1 (0.3)	2 (0.1)	2 (0.1)	1 (0.2)	9 (0.2)	1 (0.1)	*
**Multiple sclerosis**	21 (0.3)	1 (0.3)	3 (0.1)	1 (0.0)	0 (0.0)	4 (0.1)	2 (0.2)	*
**Chronic liver disease**	9 (0.1)	1 (0.3)	1 (0.0)	6 (0.3)	0 (0.0)	5 (0.1)	0 (0.0)	*
**Chronic sinusitis**	1 (0.0)	0 (0.0)	3 (0.1)	1 (0.0)	0 (0.0)	1 (0.0)	0 (0.0)	*

[§]Categories were dropped for statistical analysis. *χ2 test not performed due to small population sizes

For peer review only

Table 4b. Prevalence for each condition across ethnicities in SSD.

	White British n=4008 (30.8%)	Irish n=240 (1.8%)	Black Caribbean n=2799 (21.5%)	Black African n=1886 (14.5%)	South Asian n=421 (3.2%)	Other[§] n=2822 (21.7%)	Unknown[§] n=843 (6.5%)	Statistics
**Diabetes**	602 (15.0)	37 (15.4)	682 (24.4)	351 (18.6)	87 (20.7)	390 (13.8)	59 (7.0)	χ^2 (4) = 97.10; p <0.001
**Hypertension**	573 (14.3)	47 (19.6)	606 (21.7)	396 (21.0)	77 (18.3)	322 (11.4)	49 (5.8)	χ^2 (4) = 73.74; p <0.001
**Asthma**	443 (11.1)	23 (9.6)	408 (14.6)	134 (7.1)	26 (6.2)	217 (7.7)	40 (4.7)	χ^2 (4) = 75.96; p <0.001
**Arthritis**	252 (6.3)	17 (7.1)	206 (7.4)	86 (4.6)	29 (6.9)	103 (3.6)	9 (1.1)	χ^2 (4) = 15.48; p = 0.038
**Epilepsy**	261 (6.5)	23 (9.6)	141 (5.0)	70 (3.7)	14 (3.3)	117 (4.1)	26 (3.1)	χ^2 (4) = 32.45; p <0.001
**Cerebrovascular accident**	188 (4.7)	15 (6.2)	159 (5.7)	82 (4.3)	18 (4.3)	96 (3.4)	15 (1.8)	χ^2 (4) = 6.48; p >0.99
**Eczema**	145 (3.6)	4 (1.7)	159 (5.7)	54 (2.9)	12 (2.9)	93 (3.3)	12 (1.4)	χ^2 (4) = 33.32; p <0.001
**Migraine**	126 (3.1)	10 (4.2)	110 (3.9)	48 (2.5)	12 (2.9)	59 (2.1)	7 (0.8)	χ^2 (4) = 8.03; p = 0.904
**Ischemic heart disease**	187 (4.7)	9 (3.8)	98 (3.5)	46 (2.4)	13 (3.1)	74 (2.6)	8 (0.9)	χ^2 (4) = 19.15; p = 0.007
**COPD**	190 (4.7)	18 (7.5)	51 (1.8)	16 (0.8)	9 (2.1)	44 (1.6)	14 (1.7)	χ^2 (4) = 101.62; p <0.001
**CKD**	64 (1.6)	5 (2.1)	45 (1.6)	26 (1.4)	10 (2.4)	24 (0.9)	5 (0.6)	*
**Parkinson's disease**	79 (2.0)	4 (1.7)	49 (1.8)	21 (1.1)	6 (1.4)	36 (1.3)	6 (0.7)	*

**Heart failure**	65 (1.6)	8 (3.3)	50 (1.8)	28 (1.5)	7 (1.7)	26 (0.9)	3 (0.4)	*
**Psoriasis**	66 (1.6)	2 (0.8)	9 (0.3)	6 (0.3)	4 (1.0)	37 (1.3)	5 (0.6)	*
**Atrial fibrillation**	46 (1.1)	7 (2.9)	22 (0.8)	11 (0.6)	1 (0.2)	10 (0.4)	3 (0.4)	*
**TIA**	34 (0.8)	5 (2.1)	21 (0.8)	13 (0.7)	6 (1.4)	8 (0.3)	4 (0.5)	*
**IBD**	12 (0.3)	1 (0.4)	2 (0.1)	2 (0.1)	1 (0.2)	6 (0.2)	1 (0.1)	*
**Multiple sclerosis**	12 (0.3)	1 (0.4)	3 (0.1)	0 (0.0)	0 (0.0)	2 (0.1)	1 (0.1)	*
**Chronic liver disease**	7 (0.2)	1 (0.4)	1 (0.0)	6 (0.3)	0 (0.0)	5 (0.2)	0 (0.0)	*
**Chronic sinusitis**	1 (0.0)	0 (0.0)	3 (0.1)	1 (0.1)	0 (0.0)	1 (0.0)	0 (0.0)	*

[§]Categories were dropped for statistical analysis. *χ2 test not performed due to small population sizes

Table 4c. Prevalence for each condition across ethnicities in BD.

	**White British n=2235 (49.9%)**	**Irish n=106 (2.4%)**	**Black Caribbean n=383 (8.5%)**	**Black African n=208 (4.6%)**	**South Asian n=128 (2.9%)**	**Other[§] n=1024 (22.9%)**	**Unknown[§] n=397 (8.9%)**	**Statistics**
**Diabetes**	235 (10.5)	18 (17.0)	75 (19.6)	31 (14.9)	38 (29.7)	71 (6.9)	10 (2.5)	χ^2 (4) = 60.65; p <0.001
**Hypertension**	231 (10.3)	16 (15.1)	72 (18.8)	34 (16.3)	25 (19.5)	78 (7.6)	11 (2.8)	χ^2 (4) = 32.99; p <0.001
**Asthma**	208 (9.3)	17 (16.0)	76 (19.8)	22 (10.6)	16 (12.5)	79 (7.7)	13 (3.3)	χ^2 (4) = 39.95; p <0.001
**Arthritis**	150 (6.7)	12 (11.3)	40 (10.4)	4 (1.9)	7 (5.5)	36 (3.5)	3 (0.8)	χ^2 (4) = 19.09; p = 0.004
**Epilepsy**	78 (3.5)	3 (2.8)	23 (6.0)	7 (3.4)	3 (2.3)	30 (2.9)	3 (0.8)	*
**Cerebrovascular accident**	86 (3.8)	5 (4.7)	25 (6.5)	10 (4.8)	7 (5.5)	20 (2.0)	2 (0.5)	*
**Eczema**	77 (3.4)	3 (2.8)	26 (6.8)	7 (3.4)	5 (3.9)	15 (1.5)	4 (1.0)	*
**Migraine**	109 (4.9)	2 (1.9)	19 (5.0)	13 (6.2)	5 (3.9)	39 (3.8)	5 (1.3)	χ^2 (4) = 3.17; p >0.99
**Ischemic heart disease**	74 (3.3)	4 (3.8)	10 (2.6)	2 (1.0)	6 (4.7)	25 (2.4)	5 (1.3)	*

For peer review only

**COPD**	81 (3.6)	14 (13.2)	12 (3.1)	5 (2.4)	5 (3.9)	16 (1.6)	1 (0.3)	*
**CKD**	61 (2.7)	4 (3.8)	11 (2.9)	6 (2.9)	3 (2.3)	13 (1.3)	2 (0.5)	*
**Parkinson's disease**	38 (1.7)	2 (1.9)	7 (1.8)	4 (1.9)	5 (3.9)	9 (0.9)	0 (0.0)	*
**Heart failure**	23 (1.0)	0 (0.0)	5 (1.3)	1 (0.5)	1 (0.8)	5 (0.5)	0 (0.0)	*
**Psoriasis**	37 (1.7)	2 (1.9)	3 (0.8)	0 (0.0)	1 (0.8)	7 (0.7)	0 (0.0)	*
**Atrial fibrillation**	23 (1.0)	2 (1.9)	1 (0.3)	0 (0.0)	1 (0.8)	5 (0.5)	1 (0.3)	*
**TIA**	24 (1.1)	2 (1.9)	3 (0.8)	2 (1.0)	1 (0.8)	5 (0.5)	2 (0.5)	*
**IBD**	12 (0.5)	0 (0.0)	0 (0.0)	0 (0.0)	0 (0.0)	3 (0.3)	0 (0.0)	*
**Multiple sclerosis**	9 (0.4)	0 (0.0)	0 (0.0)	1 (0.5)	0 (0.0)	2 (0.2)	1 (0.3)	*
**Chronic liver disease**	2 (0.1)	0 (0.0)	0 (0.0)	0 (0.0)	0 (0.0)	0 (0.0)	0 (0.0)	*
**Chronic sinusitis**	0 (0.0)	0 (0.0)	0 (0.0)	0 (0.0)	0 (0.0)	0 (0.0)	0 (0.0)	*

[§]Categories were dropped for statistical analysis. *χ2 test not performed due to small population sizes

For peer review only

Supplemental Table 5. Social deprivation prevalence for each condition for the whole cohort and within SSD and BD.

	Total cohort						SSD							AD						
	**1 - least deprived n (%)**	**2 n (%)**	**3 n (%)**	**4 n (%)**	**5 - most deprived n (%)**	**Not stated§ n (%)**	**1 - least deprived n (%)**	**2 n (%)**	**3 n (%)**	**4 n (%)**	**5 - most deprived n (%)**	**Not stated§ n (%)**	**Chi square**	**1 - least deprived n (%)**	**2 n (%)**	**3 n (%)**	**4 n (%)**	**5 - most deprived n (%)**	**Not stated§ n (%)**	**Chi square**
**Total**	742 (100)	1384 (100)	3575 (100)	7073 (100)	4033 (100)	693 (100)	412 (100)	887 (100)	2503 (100)	5476 (100)	3204 (100)	537 (100)		330 (100)	497 (100)	1072 (100)	1597 (100)	829 (100)	156 (100)	
**Diabetes *****	62 (8.4)	169 (12.2)	528 (14.8)	1201 (17.0)	698 (17.3)	28 (4.0)	49 (11.9)	117 (13.2)	409 (16.3)	1019 (18.6)	594 (18.5)	20 (3.7)	$X^2(4)$ = 29.7; p<0.001	13 (3.9)	52 (10.5)	119 (11.1)	182 (11.4)	104 (12.5)	8 (5.1)	$X^2(4)$ = 19.4; p=0.007
**Hypertension *****	77 (10.4)	154 (11.1)	468 (13.1)	1189 (16.8)	627 (15.5)	22 (3.2)	51 (12.4)	108 (12.2)	368 (14.7)	1008 (18.4)	517 (16.1)	18 (3.4)	$X^2(4)$ = 37.8; p<0.001	26 (7.9)	46 (9.3)	100 (9.3)	181 (11.3)	110 (13.3)	4 (2.6)	$X^2(4)$ = 12.3; p=0.17
**Asthma *****	42 (5.7)	95 (6.9)	361 (10.1)	758 (10.7)	432 (10.7)	34 (4.9)	27 (6.6)	61 (6.9)	251 (10.0)	589 (10.8)	337 (10.5)	26 (4.8)	$X^2(4)$ = 19.0; p=0.01	15 (4.5)	34 (6.8)	110 (10.3)	169 (10.6)	95 (11.5)	8 (5.1)	$X^2(4)$ = 19.2; p=0.008
**Arthritis**	36 (4.9)	56 (4.0)	188 (5.3)	411 (5.8)	256 (6.3)	7 (1.0)	18 (4.4)	36 (4.1)	135 (5.4)	312 (5.7)	197 (6.1)	4 (0.7)	$X^2(4)$ = 7.30; p>0.99	18 (5.5)	20 (4.0)	53 (4.9)	99 (6.2)	59 (7.1)	3 (1.9)	$X^2(4)$ = 7.51; p>0.99
**Epilepsy**	32 (4.3)	50 (3.6)	160 (4.5)	344 (4.9)	202 (5.0)	11 (1.2)	24 (5.8)	40 (4.5)	123 (4.9)	292 (5.3)	164 (5.1)	9 (1.7)	$X^2(4)$ = 1.79; p>0.99	8 (2.4)	10 (2.0)	37 (3.5)	52 (3.3)	38 (4.6)	2 (1.3)	$X^2(4)$ = 7.59; p>0.99
**CVA**	19 (2.6)	52 (3.8)	146 (4.1)	328 (4.6)	180 (4.5)	3 (0.4)	12 (2.9)	33 (3.7)	107 (4.3)	272 (5.0)	146 (4.6)	3 (0.6)	$X^2(4)$ = 6.55; p>0.99	7 (2.1)	19 (3.8)	39 (3.6)	56 (3.5)	34 (4.1)	0	$X^2(4)$ = 2.80; p>0.99
**Eczema**	22 (3.0)	45 (3.3)	120 (3.4)	273 (3.9)	147 (3.6)	9 (1.3)	17 (4.1)	34 (3.8)	86 (3.4)	212 (3.9)	123 (3.8)	7 (1.3)	$X^2(4)$ = 1.11; p>0.99	5 (1.5)	11 (2.2)	34 (3.2)	61 (3.8)	24 (2.9)	2 (1.3)	$X^2(4)$ = 6.90; p>0.99
**Migraine**	17 (2.3)	39 (2.8)	121 (3.4)	243 (3.4)	140 (3.5)	4 (0.6)	11 (2.7)	20 (2.3)	63 (2.5)	176 (3.2)	99 (3.1)	3 (0.6)	$X^2(4)$ = 4.79; p>0.99	6 (1.8)	19 (3.8)	58 (5.4)	67 (4.2)	41 (4.9)	1 (0.6)	$X^2(4)$ = 8.94; p=0.69
**Ischaemic heart disease**	20 (2.7)	48 (3.5)	103 (2.9)	240 (3.4)	143 (3.5)	7 (1.0)	9 (2.2)	33 (3.7)	74 (3.0)	194 (3.5)	120 (3.7)	5 (0.9)	$X^2(4)$ = 4.99; p>0.99	11 (3.3)	15 (3.0)	29 (2.7)	46 (2.9)	23 (2.8)	2 (1.3)	$X^2(4)$ = 0.43; p>0.99
**COPD ****	6 (0.8)	35 (2.5)	83 (2.3)	208 (2.9)	139 (3.4)	5 (0.7)	3 (0.7)	22 (2.5)	59 (2.4)	159 (2.9)	95 (3.0)	4 (0.7)	$X^2(4)$ = 9.07; p=0.77	3 (0.9)	13 (2.6)	24 (2.2)	49 (3.1)	44 (5.3)	1 (0.6)	$X^2(4)$ = 21.9; p=0.002

For peer review only

**CKD**	8 (1.1)	22 (1.6)	46 (1.3)	128 (1.8)	74 (1.8)	1 (0.1)	4 (1.0)	9 (1.0)	28 (1.1)	81 (1.5)	56 (1.7)	1 (0.2)	$X^2(4) = 5.83$; $p>0.99$	4 (1.2)	13 (2.6)	18 (1.7)	47 (2.9)	18 (2.2)	0	$X^2(4) = 6.76$; $p>0.99$
**PD**	9 (1.2)	22 (1.6)	51 (1.4)	121 (1.7)	60 (1.5)	3 (0.4)	5 (1.2)	16 (1.8)	33 (1.3)	97 (1.8)	48 (1.5)	2 (0.4)	$X^2(4) = 3.13$; $p>0.99$	4 (1.2)	6 (1.2)	18 (1.7)	24 (1.5)	12 (1.4)	1 (0.6)	---
**Heart failure**	10 (1.3)	12 (0.9)	47 (1.3)	92 (1.3)	58 (1.4)	3 (0.4)	7 (1.7)	10 (1.1)	39 (1.6)	78 (1.4)	52 (1.6)	1 (0.2)	$X^2(4) = 1.54$; $p=0.82$	3 (0.9)	2 (0.4)	8 (0.7)	14 (0.9)	6 (0.7)	2 (1.3)	---
**Psoriasis**	11 (1.5)	12 (0.9)	33 (0.9)	71 (1.0)	48 (1.2)	4 (0.6)	4 (1.0)	6 (0.7)	24 (1.0)	54 (1.0)	37 (1.2)	4 (0.7)	---	7 (2.1)	6 (1.2)	9 (0.8)	17 (1.1)	11 (1.3)	0	---
**Atrial fibrillation**	10 (1.3)	11 (0.8)	27 (0.8)	53 (0.7)	29 (0.7)	3 (0.4)	6 (1.5)	7 (0.8)	21 (0.8)	42 (0.8)	23 (0.7)	1 (0.2)	---	4 (1.2)	4 (0.8)	6 (0.6)	11 (0.7)	6 (0.7)	2 (1.3)	---
**TIA**	3 (0.3)	17 (1.0)	27 (0.6)	55 (0.6)	28 (0.5)	0	1 (0.2)	9 (1.0)	16 (0.6)	43 (0.8)	22 (0.7)	0	---	2 (0.6)	8 (1.6)	11 (1.0)	12 (0.8)	6 (0.7)	0	---
**IBD**	1 (0.1)	4 (0.3)	10 (0.3)	12 (0.2)	13 (0.3)	0	0	1 (0.1)	7 (0.3)	7 (0.1)	10 (0.3)	0	---	1 (0.3)	3 (0.6)	3 (0.3)	5 (0.3)	3 (0.4)	0	---
**MS**	4 (0.5)	2 (0.1)	7 (0.2)	14 (0.2)	4 (0.1)	1 (0.1)	2 (0.5)	2 (0.2)	2 (0.1)	9 (0.2)	3 (0.1)	1 (0.2)	---	2 (0.6)	0	5 (0.5)	5 (0.3)	1 (0.1)	0	---
**Chronic liver disease**	0	1 (0.1)	8 (0.2)	8 (0.1)	5 (0.1)	0	0	0	8 (0.3)	8 (0.1)	4 (0.1)	0	---	0	1 (0.2)	0	0	1 (0.1)	0	---
**Chronic sinusitis**	0	0	1 (0.0)	2 (0.0)	3 (0.1)	0	0	0	1 (0.0)	2 (0.0)	3 (0.1)	0	---	0	0	0	0	0	0	---

*, $p<0.05$; **, $p\leq0.01$; ***, $p\leq0.001$; CVA, cerebrovascular accident; COPD, chronic obstructive lung disease; CKD, chronic kidney disease; PD, Parkinson's disease; TIA, transient ischemic attack; IBD, inflammatory bowel disease; MS, multiple sclerosis

For peer review only

For peer review only

Supplemental Table 6. Sociodemographic features of cohort in HoNOS subsample analysis

	Total n (%)	SSD n (%)	BD n (%)
**Totals n (%)**	13650 (100.0)	10384 (76.1)	3266 (23.9)
**Sex*** **			
Female	6537 (47.9)	4526 (69.2)	2011 (30.8)
Male	7112 (52.1)	5858 (82.4)	1254 (17.6)
**Age at first SMI diagnosis*** **			
15 – 34	5821 (42.6)	4420 (42.6)	1401 (42.9(
35 – 44	3430 (25.1)	2183 (75.3)	716 (24.7)
45 – 54	2082 (15.3)	1594 (76.6)	488 (23.4)
55 – 64	1089 (8.0)	811 (74.5)	278 (25.5)
65+	1759 (12.9)	1376 (13.3)	383 (11.7)
**Ethnicity*** **			
White British	4571 (33.5)	2961 (64.8)	1610 (35.2)
Black Caribbean	2849 (20.9)	2515 (88.3)	334 (11.7)
Black African	1852 (13.6)	1679 (90.7)	173 (9.3)
South Asian	436 (3.2)	338 (77.5)	98 (22.5)
Irish	286 (2.1)	197 (68.9)	89 (31.1)
Other§	3127 (22.9)	2327 (74.4)	800 (25.6)
Not stated§	529 (3.9)	367 (3.5)	162 (5.0)
**Index of multiple deprivation*** **			
1 (least deprivation)	380 (2.8)	237 (62.4)	143 (37.6)
2	883 (6.5)	581 (65.8)	302 (34.2)
3	2786 (20.4)	1994 (71.6)	792 (28.4)
4	5947 (43.6)	4654 (78.3)	1293 (21.7)
5 (most deprivation)	3308 (24.2)	2650 (80.1)	658 (19.9)
Unknown§	346 (2.5)	268 (77.5)	78 (22.5)
**Physical conditions*** **			
No mentions	7232 (53.0)	5320 (73.6)	1912 (26.4)
One	3295 (24.1)	2596 (78.8)	699 (21.2)
Two	1669 (12.2)	1340 (80.3)	329 (19.7)
Three or more	1454 (10.7)	1128 (77.6)	326 (22.4)

Note. *** $p < .001$ *for comparisons between BD and SSD groups.* §Not included in analyses.

For peer review only

Supplemental Table 7. Associations between comorbidities and HoNOS scores in SSD.

Comorbidity	HoNOS Score	Unadjusted	M1	M2a
	**Mean (SD)**	**B (95% CI)**	**B (95% CI)**	**B (95% CI)**
**Whole cohort**	10.75 (6.10)			
**Diabetes**	11.04 (6.10)	0.360 (0.067 – 0.654)**	0.224 (-0.073 – 0.522)	0.300 (-0.002 – 0.602)
Ref: No diabetes	10.68 (6.10)			
**Hypertension**	11.17 (6.05)	0.519 (0.220 – 0.818)***	0.264 (-0.048 – 0.575)	0.324 (0.006 – 0.641)*
Ref: No hypertension	10.65 (6.11)			
**Asthma**	11.19 (6.11)	0.492 (0.126 – 0.859)**	0.551 (0.185 – 0.918)**	0.530 (0.158 – 0.903)**
Ref: No asthma	10.70 (6.10)			
**Arthritis**	12.08 (6.21)	1.422 (0.948– 1.895)***	1.259 (0.775 – 1.742)***	1.274 (0.788 – 1.759)***
Ref: No arthritis	10.66 (6.08)			
**Epilepsy**	11.53 (6.24)	0.827 (0.316 – 1.337)***	0.850 (0.341 – 1.358)***	0.856 (0.341 – 1.370)***
Ref: No epilepsy	10.71 (6.09)			
**CVA**	11.87 (6.23)	1.178 (0.651 – 1.705)***	0.913 (0.383 – 1.443)***	0.921 (0.388 – 1.454)**
Ref: No CVA	10.69 (6.09)			
**Eczema**	11.48 (6.25)	0.763 (0.188 – 1.338)**	0.846 (0.273 – 1.420)**	0.754 (0.172 – 1.336)*
Ref: No eczema	10.72 (6.09)			
**Migraine**	10.71 (5.87)	-0.041 (-0.683 – 0.602)	0.196 (-0.447 – 0.839)	0.192 (-0.458 – 0.842)
Ref: No migraine	10.75 (6.11)			
**Ischaemic heart disease**	11.59 (5.91)	0.874 (0.274 – 1.474)**	0.520 (-0.083 – 1.124)	0.477 (-0.132 – 1.085)
Ref: No ischaemic heart disease	10.72 (6.11)			
**COPD**	12.15 (5.70)	1.444 (0.760– 2.128)***	1.045 (0.354 – 1.736)**	1.043 (0.343 – 1.742)**
Ref: No COPD	10.71 (6.11)			
**Number of comorbidities**		0.387 (0.294 – 0.481)***	0.328 (0.231 – 0.425)***	0.342 (0.243 – 0.441) ***
**1 or more comorbidities**	11.13 (6.11)	0.745 (0.511 –0.980)***	0.607 (0.367 – 0.847)***	0.657 (0.410 – 0.903)***
Ref: No comorbidities	10.39 (6.07)			
**2 or more comorbidities**	11.45 (6.16)	0.921 (0.646 – 1.196)***	0.747 (0.464 – 1.030)***	0.771 (0.484 – 1.059)***
Ref: Less than 1 comorbidities	10.53 (6.07)			

Supplemental Table8. Associations between comorbidities and HoNOS scores in BD.

Comorbidity	HoNOS Score	Unadjusted	M1	M2a
	**Mean (SD)**	**B (95% CI)**	**B (95% CI)**	**B (95% CI)**
**Whole cohort**	9.28 (5.77)			
**Diabetes**	10.941 (5.97)	1.304 (0.720 – 1.887)***	0.968 (0.370 – 1.565)**	0.961 (0.355 – 1.568)**
Ref: No diabetes	9.11 (5.72)			
**Hypertension**	10.13 (5.82)	0.972 (0.384 – 1.561)**	0.446 (-0.182 – 1.074)	0.277 (-0.359 – 0.912)
Ref: No hypertension	9.15 (5.76)			
**Asthma**	10.61 (6.27)	1.512 (0.900 – 2.124)***	1.597 (0.985 – 2.210)***	1.503 (0.887 – 2.120)***
Ref: No asthma	9.10 (5.68)			
**Arthritis**	11.81 (6.13)	2.725 (1.960 – 3.490)***	2.487 (1.702 – 3.273)***	2.396 (1.606 – 3.186)***
Ref: No arthritis	9.09 (5.70)			
**Epilepsy**	10.77 (5.96)	1.552 (0.513 – 2.591)**	1.587 (0.551 – 2.624)**	1.496 (0.466 – 2.527)**
Ref: No epilepsy	9.22 (5.76)			
**CVA**	11.60 (6.16)	2.425 (1.464 – 3.386)***	2.098 (1.124 – 3.071)***	2.030 (1.064 – 2.996)***
Ref: No CVA	9.17 (5.73)			
**Eczema**	9.83 (5.82)	0.578 (-0.446 – 1.603)	0.622 (-0.400 – 1.644)	0.427 (-0.603 – 1.458)
Ref: No eczema	9.26 (5.77)			
**Migraine**	9.55 (5.34)	0.284 (-0.586– 1.154)	0.523 (-0.350– 1.397)	0.527 (-0.346 – 1.400)
Ref: No migraine	9.26 (5.80)			
**Ischaemic heart disease**	11.84 (5.95)	2.649 (1.560 – 3.738)***	2.093 (0.987 – 3.200)***	2.204 (1.098 – 3.309)**
Ref: No ischaemic heart disease	9.19 (5.75)			
**COPD**	11.89 (5.99)	2.711 (1.679 – 3.743)***	2.298 (1.248 – 3.348)***	2.128 (1.077 – 3.179)***
Ref: No COPD	9.18 (5.74)			
**Number of comorbidities**		0.723 (0.566 – 0.879)***	0.661 (0.494 – 0.828)***	0.642 (0.473 – 0.811) ***
**1 or more comorbidities**	10.30 (5.91)	1.740 (1.343 –2.138)***	1.559 (1.143 – 1.974)***	1.457 (1.031 – 1.883)***
Ref: No comorbidities	8.56 (5.57)			
**2 or more comorbidities**	10.89 (5.99)	2.013 (1.524 – 2.503)***	1.797 (1.280 – 2.314)***	1.789 (1.267 – 2.311)***
Ref: Less than 1 comorbidities	8.88 (5.64)			

Supplemental Figure 1. Distribution of all conditions in the SMI cohort by SMI diagnoses

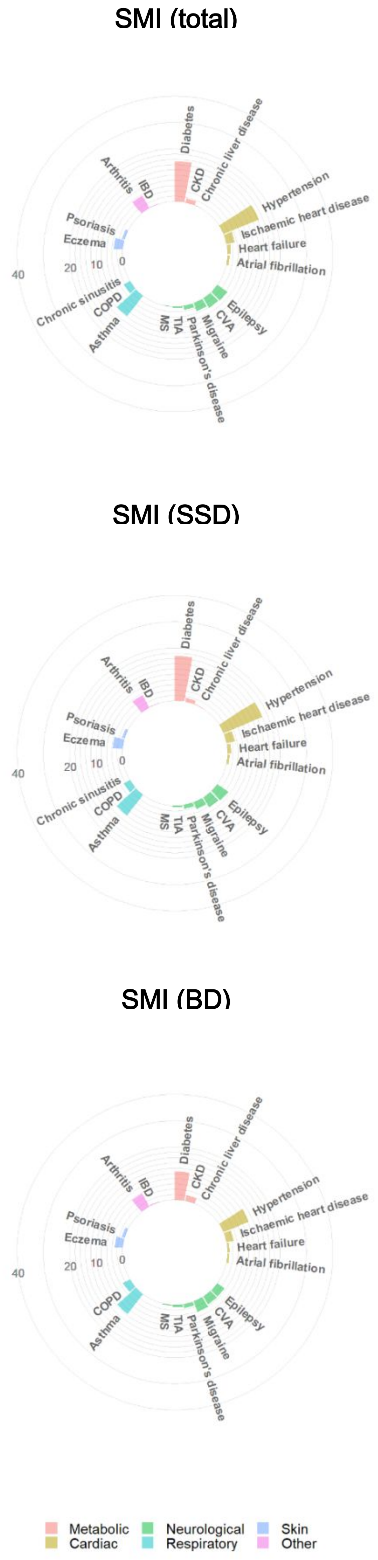

Supplemental Figure 2. Prevalence rates for age at SMI diagnoses per condition and comparison between individuals with BD and SSD.

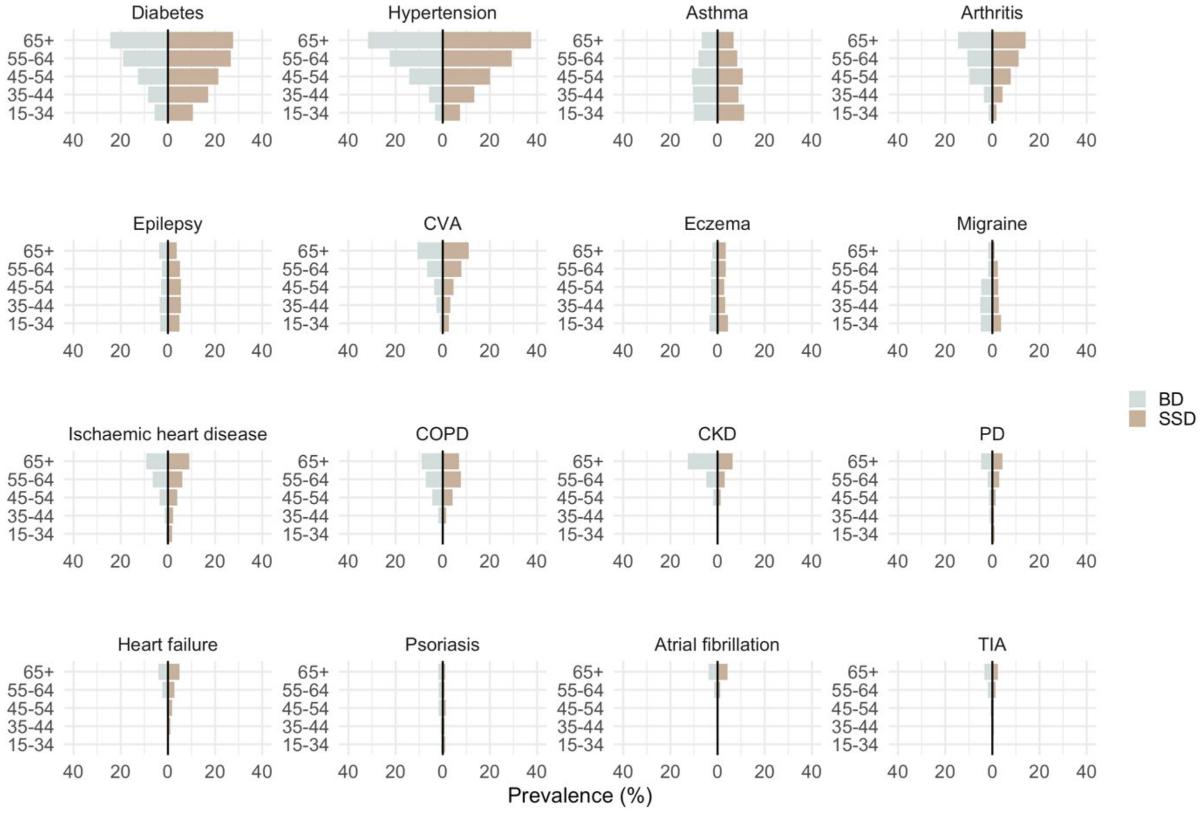

Appendix 1. SNOMED Container and Concept Level Groupings for physical health conditions included in this study.

Container Concept	Concepts
S-73211009 - Diabetes mellitus (disorder)	S-44054006 - Diabetes mellitus type 2 (disorder)<br>S-46635009 - Diabetes mellitus type 1 (disorder)<br>S-422088007 - Disorder of nervous system co-occurrent and due to diabetes mellitus (disorder)<br>S-25093002 - Disorder of eye co-occurrent and due to diabetes mellitus (disorder)<br>S-73211009 - Diabetes mellitus (disorder)
S-84114007 - Heart failure (disorder)	S-128404006 - Right heart failure (disorder)<br>S-48447003 - Chronic heart failure (disorder)<br>S-56675007 - Acute heart failure (disorder)<br>S-85232009 - Left heart failure (disorder)<br>S-42343007 - Congestive heart failure (disorder)<br>S-84114007 - Heart failure (disorder)
S-414545008 - Ischemic heart disease (disorder)	S-413439005 - Acute ischemic heart disease (disorder)<br>S-413838009 - Chronic ischemic heart disease (disorder)<br>S-194828000 - Angina (disorder)<br>S-22298006 - Myocardial infarction (disorder)<br>S-414545008 - Ischemic heart disease (disorder)
S-38341003 - Hypertensive disorder, systemic arterial (disorder)	S-31992008 - Secondary hypertension (disorder)<br>S-48146000 - Diastolic hypertension (disorder)<br>S-56218007 - Systolic hypertension (disorder)<br>S-59621000 - Essential hypertension (disorder)<br>S-38341003 - Hypertensive disorder, systemic arterial (disorder)
S-13645005 - Chronic obstructive lung disease (disorder)	S-195951007 - Acute exacerbation of chronic obstructive airways disease (disorder)<br>S-87433001 - Pulmonary emphysema (disorder)<br>S-13645005 - Chronic obstructive lung disease (disorder)
S-195967001 - Asthma (disorder)	S-195967001 - Asthma (disorder)
S-709044004 - Chronic kidney disease (disorder)	S-723190009 - Chronic renal insufficiency (disorder)<br>S-709044004 - Chronic kidney disease (disorder)

For peer review only

S-230690007 - Cerebrovascular accident (disorder)	S-25133001 - Completed stroke (disorder)<br>S-371040005 - Thrombotic stroke (disorder)<br>S-371041009 - Embolic stroke (disorder)<br>S-413102000 - Infarction of basal ganglia (disorder)<br>S-422504002 - Ischemic stroke (disorder)<br>S-723082006 - Silent cerebral infarct (disorder)<br>S-1078001000000105 - Haemorrhagic stroke (disorder)<br>S-230690007 - Cerebrovascular accident (disorder)
S-266257000 - Transient ischemic attack (disorder)	S-266257000 - Transient ischemic attack (disorder)
S-49049000 - Parkinson's disease (disorder)	S-49049000 - Parkinson's disease (disorder)
S-24700007 - Multiple sclerosis (disorder)	S-24700007 - Multiple sclerosis (disorder)
S-84757009 - Epilepsy (disorder)	S-352818000 - Tonic-clonic epilepsy (disorder)<br>S-19598007 - Generalized epilepsy (disorder)<br>S-230456007 - Status epilepticus (disorder)<br>S-509341000000107 - Petit-mal epilepsy (disorder)<br>S-84757009 - Epilepsy (disorder)
S-37796009 - Migraine (disorder)	S-37796009 - Migraine (disorder)<br>S-4473006 - Migraine with aura (disorder)<br>S-56097005 - Migraine without aura (disorder)
S-53741008 - Coronary arteriosclerosis (disorder)	S-810681000000101 - Coronary microvascular disease (disorder)<br><br>S-53741008 - Coronary arteriosclerosis (disorder)
S-49436004 - Atrial fibrillation (disorder)	S-49436004 - Atrial fibrillation (disorder)
S-40055000 - Chronic sinusitis (disorder)	S-40055000 - Chronic sinusitis (disorder)
S-24526004 - Inflammatory bowel disease (disorder)	S-24526004 - Inflammatory bowel disease (disorder)<br>S-397173003 - Crohn's disease of intestine (disorder)<br>S-64766004 - Ulcerative colitis (disorder)
S-328383001 - Chronic liver disease (disorder)	S-328383001 - Chronic liver disease (disorder)<br>S-76783007 - Chronic hepatitis (disorder)<br>S-79720007 - Chronic nonalcoholic liver disease (disorder)

For peer review only

	S-713181003 - Chronic alcoholic liver disease (disorder)
S-9014002 - Psoriasis (disorder)	S-9014002 - Psoriasis (disorder)
S-43116000 - Eczema (disorder)	S-43116000 - Eczema (disorder)
S-3723001 - Arthritis (disorder)	S-69896004 - Rheumatoid arthritis (disorder)<br>S-399112009 - Seronegative arthritis (disorder)<br>S-35908007 - Chronic arthritis (disorder)<br>S-11939005 - Acute arthritis (disorder)<br>S-3723001 - Arthritis (disorder)

For peer review only

STROBE Statement—checklist of items that should be included in reports of observational studies

	Item No	Recommendation	Page No
**Title and abstract**	1	(*a*) Indicate the study's design with a commonly used term in the title or the abstract	1
		(*b*) Provide in the abstract an informative and balanced summary of what was done and what was found	2
**Introduction**			
Background/rationale	2	Explain the scientific background and rationale for the investigation being reported	4,5
Objectives	3	State specific objectives, including any prespecified hypotheses	5
**Methods**			
Study design	4	Present key elements of study design early in the paper	5,6
Setting	5	Describe the setting, locations, and relevant dates, including periods of recruitment, exposure, follow-up, and data collection	5,6
Participants	6	(*a*) *Cohort study*—Give the eligibility criteria, and the sources and methods of selection of participants. Describe methods of follow-up<br>*Case-control study*—Give the eligibility criteria, and the sources and methods of case ascertainment and control selection. Give the rationale for the choice of cases and controls<br>*Cross-sectional study*—Give the eligibility criteria, and the sources and methods of selection of participants	5
		(*b*) *Cohort study*—For matched studies, give matching criteria and number of exposed and unexposed<br>*Case-control study*—For matched studies, give matching criteria and the number of controls per case	
Variables	7	Clearly define all outcomes, exposures, predictors, potential confounders, and effect modifiers. Give diagnostic criteria, if applicable	6-8
Data sources/ measurement	8*	For each variable of interest, give sources of data and details of methods of assessment (measurement). Describe comparability of assessment methods if there is more than one group	*6-8*
Bias	9	Describe any efforts to address potential sources of bias	6-9
Study size	10	Explain how the study size was arrived at	5,6
Quantitative variables	11	Explain how quantitative variables were handled in the analyses. If applicable, describe which groupings were chosen and why	6-8
Statistical methods	12	(*a*) Describe all statistical methods, including those used to control for confounding	8,9
		(*b*) Describe any methods used to examine subgroups and interactions	8,9
		(*c*) Explain how missing data were addressed	8,9
		(*d*) *Cohort study*—If applicable, explain how loss to follow-up was addressed<br>*Case-control study*—If applicable, explain how matching of cases and controls was addressed<br>*Cross-sectional study*—If applicable, describe analytical methods taking account of sampling strategy	8,9
		(*e*) Describe any sensitivity analyses	8,9

Continued on next page

For peer review only - http://bmjopen.bmj.com/site/about/guidelines.xhtml

**Results**			
Participants	13*	(a) Report numbers of individuals at each stage of study—eg numbers potentially eligible, examined for eligibility, confirmed eligible, included in the study, completing follow-up, and analysed	10
		(b) Give reasons for non-participation at each stage	
		(c) Consider use of a flow diagram	
Descriptive data	14*	(a) Give characteristics of study participants (eg demographic, clinical, social) and information on exposures and potential confounders	10
		(b) Indicate number of participants with missing data for each variable of interest	
		(c) *Cohort study*—Summarise follow-up time (eg, average and total amount)	
Outcome data	15*	*Cohort study*—Report numbers of outcome events or summary measures over time	
		Case-control study—Report numbers in each exposure category, or summary measures of exposure	
		Cross-sectional study—Report numbers of outcome events or summary measures	*9-13*
Main results	16	(*a*) Give unadjusted estimates and, if applicable, confounder-adjusted estimates and their precision (eg, 95% confidence interval). Make clear which confounders were adjusted for and why they were included	9-13
		(*b*) Report category boundaries when continuous variables were categorized	
		(*c*) If relevant, consider translating estimates of relative risk into absolute risk for a meaningful time period	
Other analyses	17	Report other analyses done—eg analyses of subgroups and interactions, and sensitivity analyses	Supp
**Discussion**			
Key results	18	Summarise key results with reference to study objectives	13,14
Limitations	19	Discuss limitations of the study, taking into account sources of potential bias or imprecision. Discuss both direction and magnitude of any potential bias	16
Interpretation	20	Give a cautious overall interpretation of results considering objectives, limitations, multiplicity of analyses, results from similar studies, and other relevant evidence	13-17
Generalisability	21	Discuss the generalisability (external validity) of the study results	16,17
**Other information**			
Funding	22	Give the source of funding and the role of the funders for the present study and, if applicable, for the original study on which the present article is based	18

*Give information separately for cases and controls in case-control studies and, if applicable, for exposed and unexposed groups in cohort and cross-sectional studies.

Note: An Explanation and Elaboration article discusses each checklist item and gives methodological background and published examples of transparent reporting. The STROBE checklist is best used in conjunction with this article (freely available on the Web sites of PLoS Medicine at http://www.plosmedicine.org/, Annals of Internal Medicine at http://www.annals.org/, and Epidemiology at http://www.epidem.com/). Information on the STROBE Initiative is available at www.strobe-statement.org.

For peer review only - http://bmjopen.bmj.com/site/about/guidelines.xhtml

BMJ Open

Mapping Multimorbidity in Individuals with Schizophrenia and Bipolar Disorders: Evidence from the South London and Maudsley NHS Foundation Trust Biomedical Research Centre (SLAM BRC) Case Register

Journal:	*BMJ Open*
Manuscript ID	bmjopen-2021-054414.R1
Article Type:	Original research
Date Submitted by the Author:	16-Sep-2021

Complete List of Authors:	Bendayan, Rebecca; King's College London, Biostatistics and Health Informatics, Institute of Psychiatry, Psychology and Neurosciences; NIHR Biomedical Research Centre at South London and Maudsley NHS Foundation Trust and King's College London, London, United Kingdom<br>Kraljevic, Zeljko; King's College London, Biostatistics and Health Informatics, Institute of Psychiatry, Psychology and Neuroscience<br>Shaari, Shaweena; NIHR Biomedical Research Centre at South London and Maudsley NHS Foundation Trust and King's College London, London, United Kingdom<br>Das-Munshi, Jayati; King's College London Institute of Psychiatry Psychology and Neuroscience, Psychological Medicine<br>Leipold, Leona; NIHR Biomedical Research Centre at South London and Maudsley NHS Foundation Trust and King's College London, London, United Kingdom<br>Chaturvedi, Jaya; King's College London, Biostatistics and Health Informatics, Institute of Psychiatry, Psychology and Neuroscience<br>Mirza, Luwaiza; NIHR Biomedical Research Centre at South London and Maudsley NHS Foundation Trust and King's College London, London, United Kingdom<br>Aldelemi, Sarah; NIHR Biomedical Research Centre at South London and Maudsley NHS Foundation Trust and King's College London, London, United Kingdom<br>Searle, Thomas; King's College London, Department of Biostatistics and Health Informatics<br>Chance, Natalia; NIHR Biomedical Research Centre at South London and Maudsley NHS Foundation Trust and King's College London, London, United Kingdom<br>Mascio, Aurelie; King's College London, Biostatistics and Health Informatics, Institute of Psychiatry, Psychology and Neuroscience<br>Skiada, Naoko; King's College London, Biostatistics and Health Informatics, Institute of Psychiatry, Psychology and Neuroscience<br>Wang, Tao; Department of Biostatistics and Health Informatics, Institute of Psychiatry, Psychology and Neuroscience, King's College London, London, United Kingdom<br>Roberts, Angus; King's College London, Biostatistics and Health Informatics, Institute of Psychiatry, Psychology and Neuroscience; NIHR Biomedical Research Centre at South London and Maudsley NHS Foundation Trust and King's College London, London, United Kingdom

	Stewart, Robert; King's College London, Psychological Medicine; NIHR Biomedical Research Centre at South London and Maudsley NHS Foundation Trust and King’s College London, London, United Kingdom<br>Bean, Daniel; King's College London, Biostatistics and Health Informatics<br>Dobson, Richard; King's College London,
**Primary Subject Heading**:	Mental health
Secondary Subject Heading:	Public health, Epidemiology, Health informatics
Keywords:	Health informatics < BIOTECHNOLOGY & BIOINFORMATICS, EPIDEMIOLOGY, MENTAL HEALTH, PSYCHIATRY, PUBLIC HEALTH

SCHOLARONE™
Manuscripts

I, the Submitting Author has the right to grant and does grant on behalf of all authors of the Work (as defined in the below author licence), an exclusive licence and/or a non-exclusive licence for contributions from authors who are: i) UK Crown employees; ii) where BMJ has agreed a CC-BY licence shall apply, and/or iii) in accordance with the terms applicable for US Federal Government officers or employees acting as part of their official duties; on a worldwide, perpetual, irrevocable, royalty-free basis to BMJ Publishing Group Ltd ("BMJ") its licensees and where the relevant Journal is co-owned by BMJ to the co-owners of the Journal, to publish the Work in this journal and any other BMJ products and to exploit all rights, as set out in our licence.

The Submitting Author accepts and understands that any supply made under these terms is made by BMJ to the Submitting Author unless you are acting as an employee on behalf of your employer or a postgraduate student of an affiliated institution which is paying any applicable article publishing charge ("APC") for Open Access articles. Where the Submitting Author wishes to make the Work available on an Open Access basis (and intends to pay the relevant APC), the terms of reuse of such Open Access shall be governed by a Creative Commons licence – details of these licences and which Creative Commons licence will apply to this Work are set out in our licence referred to above.

Other than as permitted in any relevant BMJ Author's Self Archiving Policies, I confirm this Work has not been accepted for publication elsewhere, is not being considered for publication elsewhere and does not duplicate material already published. I confirm all authors consent to publication of this Work and authorise the granting of this licence.

For peer review only

Mapping Multimorbidity in Individuals with Schizophrenia and Bipolar Disorders: Evidence from the South London and Maudsley NHS Foundation Trust Biomedical Research Centre (SLAM BRC) Case Register

Rebecca Bendayan[1,2], Zeljko Kraljevic[2], Shaweena Shaari[1], Jayati Das-Munshi[3], Leona Leipold[1], Jaya Chaturvedi[2], Luwaiza Mirza[1], Sarah Aldelemi[1], Thomas Searle[2], Natalia Chance[1], Aurelie Mascio[2], Naoko Skiada[2], Tao Wang[2], Angus Roberts[1,2], Robert Stewart[1,3], Daniel Bean[2,4], Richard Dobson[1,2,5]

[1]NIHR Biomedical Research Centre at South London and Maudsley NHS Foundation Trust, London, United Kingdom

[2]Department of Biostatistics and Health Informatics, Institute of Psychiatry, Psychology and Neuroscience, King's College London, London, United Kingdom

[3]Department of Psychological Medicine, Institute of Psychiatry, Psychology and Neuroscience, King's College London, London, United Kingdom

[4]Health Data Research UK London, University College London, London, United Kingdom

[5]Institute of Health Informatics, University College London, London, United Kingdom

Word count: 3920

Correspondence:
Rebecca Bendayan / ORCID: 0000-0003-1461-556X
E-mail: rebecca.bendayan@kcl.ac.uk
NIHR Maudsley Biomedical Research Centre
Department of Biostatistics & Health Informatics
SGDP Centre, IoPPN, Box PO 80
De Crespigny Park, Denmark Hill
London SE5 8AF, UNITED KINGDOM

For peer review only

Abstract

Objectives: The first aim of this study was to design and develop a valid and replicable strategy to extract physical health conditions from clinical notes which are common in mental health services. Then, we examined the prevalence of these conditions in individuals with SMI and compared their individual and combined prevalence in individuals with bipolar (BD) and schizophrenia spectrum disorders (SSD).

Design: Observational study.

Setting: Secondary mental healthcare services from South London

Participants: Our maximal sample comprised 17,500 individuals aged 15 years or older who had received a primary or secondary SMI diagnosis (ICD-10, F20-31) between 2007 and 2018.

Measures: We designed and implemented a data extraction strategy for 21 common physical comorbidities using a natural language processing pipeline, MedCAT. Associations were investigated with sex, age at SMI diagnosis, ethnicity and social deprivation for the whole cohort and the BD and SSD subgroups. Linear regression models were used to examine associations with disability measured by the Health of Nations Outcome Scale (HoNOS).

Results: Physical health data was extracted, achieving precision rates (F1) above 0.90 for all conditions. The ten most prevalent conditions were diabetes, hypertension, asthma, arthritis, epilepsy, cerebrovascular accident, eczema, migraine, ischaemic heart disease and COPD. The most prevalent combination in this population included diabetes, hypertension and asthma, regardless of their SMI diagnoses.

Conclusions: Our data extraction strategy was found to be adequate to extract physical health data from clinical notes, which is essential for future multimorbidity research using text records. We found that around 40% of our cohort had multimorbidity from which 20% had complex

For peer review only

multimorbidity (two or more physical conditions besides SMI). Sex, age, ethnicity and social deprivation were found to be key to understand their heterogeneity and their differential contribution to disability levels in this population. These outputs have direct implications for researchers and clinicians.

Strengths and limitations of this study

- We designed and implemented a data extraction strategy with good which showed high performance rates and allowed us to unlock data from 21 physical health conditions from around 15m clinical documents with free text.
- We mapped how these health conditions are distributed across sex, age, ethnicity, social disadvantage and severe mental illness diagnoses in a sample of 17500 patients from one of Europe's largest providers of secondary mental healthcare, serving a geographic catchment of approximately 1.32 million residents in London.
- The association between multimorbidity (2 or more conditions) and disability was examined. Further research is needed to understand potential explanatory pathways for the association between multimorbidity and disability in this population.
- This study focuses on a cohort of individuals with SMI which limits direct comparisons with other mental health conditions and/or general population.
- Although some of the most frequent physical comorbidities were extracted, some specific health conditions required to compute standard comorbidity scores (e.g., Charlson and Exlihauser comorbidity indexes) were not included in this study.

For peer review only

Introduction

Two thirds of the deaths in individuals with severe mental illness (SMI) are potentially explained by the increased risk of multimorbidity in this population.[1–4] However, multimorbidity research in this population is still scarce[5] compounded by the limited availability of physical health data in SMI samples, increased non-response rates in surveys,[6] and physical health information in secondary mental health care data primarily hidden in free text fields.

Most research to date on physical health in SMI populations has focused on cardiometabolic risk factors which are considered leading contributors to cardiovascular diseases in individuals with SMI,[7–10] or specific conditions such as immune-mediated inflammatory diseases (e.g., inflammatory bowel diseases, psoriasis),[11–13] multiple sclerosis, epilepsy or migraine.[14,15] This condition-specific vision limits our understanding of multimorbidity in SMI and studies that consider a larger number of conditions are needed. However, there are only a few studies which have considered multiple health conditions.[4,16,17] Woodhead et al.[4] showed an increased risk in multimorbidity in SMI patients, but only found epilepsy to be more prevalent as an individual condition. Kugathasan et al.[17] investigated combinations of diseases in schizophrenia at organ system level and found that 31% had complex multimorbidity with the most prevalent pairs including neurologic-endocrine, neurologic-respiratory and neurologic-viral. Similarly, epilepsy and arthritis was one of the most prevalent combinations found by Dorrington et al.[16] Although these studies included multiple health conditions and confirmed the need of investigating further multimorbidity in SMI, they are still not comparable to multimorbidity studies in general populations[18–20] and did not investigate potential differences between individuals with schizophrenia spectrum disorders (SSD) and bipolar disorders (BD). Understanding different multimorbidity combinations between those groups could contribute to the ongoing debate around

For peer review only

potential underlying biological mechanisms.[21–23] Ultimately, SSD and BD have been established as significant drivers of disability[24] and deficits in physical health have been implicated in the perpetuation of impairments in functional capacity and performance,[25,26] however, research into the relationship between multimorbidity and disability in SMI is limited.

Within this context, our first aim was to design and develop a suitable strategy to extract information on physical health conditions from free text mental health records data which could be easily replicated in future multimorbidity research using similar resources. Our second objective was to examine the prevalence of these conditions and their most prevalent combinations in SMI and any differences across relevant sociodemographic factors and across SMI diagnoses (SSD vs BD). Our third objective was to investigate the association of overall multimorbidity and specific physical health conditions with levels of disability measured using the Health of the Nation Outcome Scales (HoNOS).

Methods

Setting and sample

Patient data were extracted via the Clinical Record Interactive Search (CRIS), a case register platform that contains de-identified mental healthcare electronic health record data from the South London and Maudsley Trust NHS Foundation Trust (SLaM). SLaM is one of Europe's largest providers of secondary mental healthcare, serving a geographic catchment of approximately 1.32 million residents, and providing almost complete coverage of secondary mental healthcare provision to all age groups. Since 2007, fully electronic clinical records have been deployed in SLaM, and data from these are accessible via CRIS system which allows searching and retrieval of anonymized full records for over 500,000 cases currently represented in the system.[27]

For peer review only

Our sample (N=17500) consisted of all individuals aged 15 years or older who had received a primary or secondary SMI diagnosis between 2007 and 2018 (International Classification of Mental and Behavioural Disorders 10th edition [ICD-10][28] codes F20-31). As one of our objectives was to compare SSD (F20-29) and BD (F30-31), individuals who over those 10 years of follow-up had diagnoses within both categories were excluded (n=804). Excluded individuals were more likely to be female, under the age of 35 at first SMI diagnosis recorded, Black ethnicity and have higher levels of social deprivation.

Physical health conditions

Definitions and Information extraction. To maximise comparability we sought to extract the following 21 physical health conditions representing chronic conditions commonly collected in multimorbidity studies using primary care data:[18–20] diabetes mellitus, heart failure, ischemic heart diseases, hypertension, coronary arteriosclerosis, chronic obstructive pulmonary disease (COPD), asthma, chronic kidney disease, cerebrovascular accident, transient ischemic attack, Parkinson's disease, multiple sclerosis, epilepsy, migraine, atrial fibrillation, chronic sinusitis, inflammatory bowel disease, chronic liver diseases, psoriasis, eczema and arthritis. These were mapped to SNOMED codes where the top concept was the group identifier and then all direct children of that concept were examined and individually reviewed by two clinicians (Appendix 1). Physical health conditions were ascertained from data reported in text records from CRIS since 2007 until 1st August 2019 for each individual resulting in around 15m documents.

To extract SNOMED concepts from the free text we used MedCAT,[29] a medical concept annotation toolkit capable of named entity recognition linking (NER+L) with contextualization. The base model used is described in Kraljevic et al.,[29] and has shown very good performance (F1=0.90). In a first step, the base model was enriched with concept names from UMLS with the

For peer review only - http://bmjopen.bmj.com/site/about/guidelines.xhtml

purpose of increasing recall and potentially catching all different name-forms for each concept. In a second step, to increase precision, MedCAT was trained in an unsupervised fashion on all the available documents; and in a third step, all the free text was annotated for the chosen SNOMED concepts. For each condition, 300 documents were randomly extracted, which resulted in a total of 6300 annotated documents.

Annotation of physical health conditions. To ensure consistent, high-quality gold standard and training data, we developed annotation guidelines based on series of iterative discussions including clinical and technical expertise. These guidelines, available upon request, were piloted and refined in preliminary stages. A relevant instance was defined as a mention of a physical health condition experienced by the patient and not negated. Each MedCAT detection was first validated as either correct/wrong - meaning the portion of text that was detected by MedCAT was either a correct/wrong detection of the relevant concept. Correct detections were further annotated with contextual annotations (or meta-annotations) for 'Diagnosis' and 'Status'. Diagnosis was used to determine if the detected concept is a patient related diagnosis, and Status if the detected concept is affirmed. Eight annotators were trained for this task and given the same instructions. MedCATtrainer[30] was used to facilitate manual annotations and each document was double annotated. Disagreements between annotators were further evaluated and resolved by a third annotator.

Training and validation. Once the dataset was annotated it was split into a training and validation set. For NER+L, 70% of the dataset was used for training and 30% for validation. For meta-annotations, 80% was used for training and 20% for validation. Hyperparameter optimization in both cases used a 10-fold cross validation on the training set.

For peer review only

Socio-demographics. Extracted data included sex, age at SMI diagnosis (15-24, 25-34, 35-44, 45-54, 55-64, 65-74, 75+), and ethnicity (White British, Irish, Black Caribbean (including mixed White and Black Caribbean and any other Black background), Black African (including mixed White and Black African), South Asian (Indian, Pakistani and Bangladeshi) and Other). Index of Multiple Deprivation (IMD) was extracted as a measure of neighbourhood socioeconomic status at the level of the 2011 Lower Layer Super Output Area (LSOA11; a standard postal unit with an average 1500 residents) corresponding to the individual's address at time of SMI diagnosis. Using the IMD, each LSOA11 is ranked from 1 (most deprived) to 32,844 (least deprived) based on seven Census-derived indicators, which was subsequently divided into quintiles.[31]

Disability. Disability was measured using Health of the Nation Outcome Scales (HoNOS):[32] a clinician-rated tool developed to measure health and social functioning. It includes 12 subscales: agitated behaviour; non-accidental self-injury; problem drinking or drug taking; cognitive problems; physical illness problems; problems associated with hallucinations or delusions; problems associated with depression; other mental and behaviour problems; problems with relationships; problems with activities of daily living; problems with living conditions; and problems with occupation and activities.[32] Total adjusted HoNOS scores of individuals at the first SMI diagnosis recorded in CRIS, or closest to that time, were used in this study. Higher scores for HoNOS indicate higher levels of impairment in the individual's functioning.

Statistical Analyses

To explore the suitability of MedCAT for extracting these physical health conditions from this cohort (objective 1), inter-rater agreement estimates were computed and performance, precision and recall per condition were estimated.

For peer review only

To examine the prevalence of these conditions in SMI across relevant factors and compare the most prevalent multimorbidity combinations for individuals with BD and SSD (objective 2). Descriptive statistics were derived for all the variables. Chi-square tests and Fisher's exact tests, with Bonferroni correction for multiple comparisons, were performed to explore associations with covariates differences between BD and SSD.

To address our third objective (to investigate the association of multimorbidity and specific physical health with levels of disability), we performed series of hierarchical linear regressions. Models were adjusted by age and sex (Model 1), and then additionally adjusted by IMD (Model 2a) or SMI diagnosis (Model 2b). All analyses were performed using R 4.0.3 and RStudio 1.3.1093.

Patient and Public Involvement Statement

When designing this project, the Data Linkage Service User and Carer Advisory Group was consulted and followed up presenting preliminary results. This is a well-established Patient and Public Involvement Group set up by the Biomedical Research Center (BRC) at South London and Maudsley Trust NHS Foundation Trust (SLaM).[33]

Results

Inter-annotator agreement and model validation for data extraction

For each physical health condition, 300 documents were annotated to create a gold standard and training data specific to each condition. All 6300 instances across 21 health conditions were double annotated yielding an average inter-annotator agreement of 97% for NER+L, 82.70% for the meta-annotation Diagnosis and 78.08% for the meta-annotation Status. Precision, recall and F1 metrics of each modelled physical health condition are shown in Table 1. Coronary arteriosclerosis was not extracted as the number of positive mentions was too small for training and validation. Overall

meta-annotations performance results showed good performance for Diagnosis and Status (Supplemental Table 1).

PLEASE INSERT TABLE 1

Mapping of physical health conditions and comparison between SSD and BD

Our sample consisted of 17,500 individuals with SMI, of whom 74.4% were diagnosed with SSD and 25.6% with BD. A slight majority were male (53.6%), and most individuals had their first SMI diagnoses report under the age of 35 (42.8%). The White British group accounted for 35.7%, with Black Caribbean (18.2%) and Black African (12.0%) groups being the next two largest groups and South Asian and Irish groups were the smallest, with 3.1 and 2.0% respectively (Table 2). There were high levels of deprivation in the cohort, with over 60% falling into the lowest two national quintiles. Around 40% had at least one mention of a physical health condition and around 20% had two or more physical conditions. There were significant differences between BD and SSD for most of the socio-demographic characteristics and number of physical health conditions (Table 2 and Figure 1). Individuals with SSD were more likely to be men, from ethnic minorities, living in more deprived neighbourhoods and had a higher number of physical health conditions recorded compared those with BD.

The three most common physical health conditions recorded were diabetes, hypertension and asthma (15.3%, 14.5% and 9.8% respectively), regardless of the specific SMI diagnoses (Supplemental Figure 1 within SSD and BD). When we compared individuals with SSD and BD, we found that the top 10 most prevalent health conditions were similar between groups but diabetes (SSD 17% vs BD 10.7%), hypertension (SSD 15.9% vs BD 10.4%) and epilepsy (SSD 5.0% vs BD 3.3%) prevalence rates were slightly higher for individuals with SSD while individuals with BD showed higher prevalence rates of migraine (BD 4.3% vs SSD 2.9%).

For peer review only

PLEASE INSERT TABLE 2 AND FIGURE 1 HERE

When we explored differences by sex in the whole cohort, we found that women were more likely to report hypertension, asthma, arthritis, eczema, and migraine compared to men (Supplemental Table 2). Within the individuals with SSD, women were in addition found to be more likely to report higher rates of diabetes, chronic kidney disease (CKD), heart failure and transient ischaemic attack (TIA). Within individuals with BD, sex differences were only found for asthma, arthritis, migraine and ischemic heart disease. Women with BD were more likely to report asthma, arthritis and migraine while men with BD were more likely to report ischemic heart disease.

With regards to differences across age groups, we found higher prevalence rates of diabetes, hypertension, arthritis, cerebrovascular accident, ischaemic heart disease, COPD, CKD, Parkinson's disease, heart failure, atrial fibrillation and TIA in individuals in older age ranges, while asthma and migraine were more prevalent in those in younger age ranges (Supplemental Table 3, Supplemental Figure 2). Similar results within individuals with SSD and BD (except for asthma and cerebrovascular accident in BD).

We found differences for ethnicity in individuals with diabetes, hypertension, asthma, arthritis, epilepsy, eczema, ischaemic heart disease, COPD and cerebrovascular accident (Figure 2, Supplemental Tables 4). Individuals from Black or South Asian minorities were more likely to show higher prevalence rates of diabetes and hypertension compared to those White British or Irish. Black Caribbean showed the higher prevalence rates of asthma or eczema among all other groups. Arthritis, COPD, epilepsy and IHD seem to be slightly more prevalent in White British or Irish, with epilepsy showing the highest prevalence rates among Irish. Similar trends were found within SSD and BD subgroups with diabetes rates higher in South Asians with BD (29.7%) compared to South Asians with SSD (20.7%); while diabetes rates in Black Caribbean with SSD

For peer review only

(24.4%) were higher than in those with BD (19.6%). With regards to social deprivation, we found that individuals with diabetes, hypertension, asthma, and COPD were more likely to be higher levels of deprivation compared to those that did not have these specific conditions (Supplemental Table 5).

PLEASE INSERT FIGURE 2 AROUND HERE

Multimorbidity combinations for the whole cohort and SSD and BD subgroups

Table 3 summarizes the ten most common physical comorbidities in patients with SMI, their prevalence, the mean number of comorbidities, and the three most frequently associated comorbidities, for the total cohort and by SMI diagnosis. While there were no clear differences in the mean number of comorbidities by SMI diagnosis, the presence of one physical condition predisposed individuals to at least one other condition; the mean number of comorbidities in the total cohort was 0.74, jumping to at least 2.20 in the presence of one of the ten most common comorbidities. The three most commonly associated physical comorbidities remained relatively consistent by SMI diagnosis, with a few exceptions. The prevalence of associated comorbidities with epilepsy are lower in BD than SSD, a fairly different comorbidity profile in migraine between SMI diagnoses and a lower rate of diabetes in individuals with comorbid BD and COPD (when compared to SSD). The most common combination of conditions included diabetes, hypertension and asthma, regardless of their SMI diagnoses. Most individuals with these combinations of conditions were also likely to have arthritis. Figures 3, 4 and 5 show the most prevalent conditions for individuals with SMI and comorbid diabetes, hypertension and asthma, respectively.

PLEASE INSERT TABLE 3 AND FIGURES 3,4 AND 5 AROUND HERE

Association with disability

For peer review only

HoNOS descriptive statistics for the whole SMI cohort (Mean=10.40, SD=6.06) and the ten most common physical comorbidities are shown in Table 4. Regression analyses showed that individuals with any of these conditions (except migraine) showed higher HoNOS scores, even after adjustments for age, sex, IMD or SMI diagnoses. We also examined whether simple and complex multimorbidity was also associated with HoNOS total score and we found that a strong positive association minimally attenuated after adjustments. Similar socio-demographics and trends were found within BD and SSD groups (Supplemental Tables 6-8). However, associations for diabetes, hypertension and ischaemic heart disease were fully attenuated after adjustments in the SSD group and associations for hypertension were also fully attenuated after adjustments in the BD group.

PLEASE INSERT TABLE 4 AROUND HERE

Discussion

The first objective of this study was to design and develop a suitable strategy to extract physical health conditions which could be easily replicated in future multimorbidity research using mental health electronic health records. The NLP strategy using MedCAT provided very good performance estimates for all the conditions extracted, which supports its suitability to extract data on physical health conditions from mental health clinical notes. These findings are consistent with previous research which has used MedCAT to extract data from hospital settings.[34,35] This resource should help to facilitate and promote research on multimorbidity using mental health records, in general, and has the potential for direct replication in other mental health trusts that have already deployed CRIS platforms.

Our second objective was to examine the prevalence of these conditions in SMI individuals and compare the most prevalent multimorbidity combinations between individuals with BD and SSD.

When we examined differences in socio-demographic variables by diagnosis, our findings were largely consistent with previous research,[3,36–39] although associations between ethnicity and BD are less established.[40] With regards to sociodemographic differences, we found that women with SSD were more likely to have diabetes, CKD, heart failure and TIA compared to men with SSD; and women with BD were more likely to have asthma, arthritis and migraine compared to men with BD. Previous research in this population showed mixed results. Some studies found higher prevalence of hypertension in women with SSD[41] and diabetes in women in BD,[42] and others did not find relevant sex differences.[43] Our findings suggest that there could be an increased risk for diabetes and hypertension for females with an SMI diagnosis, especially in SSD. Further research in this line is needed.

Ethnic differences were found for diabetes, hypertension, asthma, arthritis, epilepsy, eczema, ischaemic heart disease, COPD and cerebrovascular accident. Individuals from Black or South Asian minorities were more likely to show higher prevalence rates of diabetes and hypertension compared to White British or Irish. Black Caribbean showed the higher prevalence rates of asthma or eczema among all other groups. Arthritis, COPD, epilepsy and IHD seem to be slightly more prevalent in White British or Irish, with epilepsy showing the highest prevalence rates among Irish. Similar trends were found within SSD and BD subgroups with diabetes rates higher in South Asians with BD compared to South Asians with SSD, while diabetes rates in Black Caribbean with SSD were higher than in those with BD. These results largely mirror previous research in ethnicity.[44–50] When we examined social deprivation, individuals with diabetes, hypertension, asthma, and COPD were more likely to report the highest levels of deprivation regardless of their SMI diagnoses. Similar to ethnicity, these results are also consistent with findings in the general

For peer review only

population where higher levels of social deprivation are found in those with comorbid diabetes,[51,52] hypertension,[53] asthma[54] or COPD.[55]

Overall, in the whole SMI cohort, around 40% of the individuals had at least one mention of a physical health condition and close to 20% had two or more physical conditions, which could be labelled as complex multimorbidity. These findings provide evidence to support previous research suggestions about the increased probability of multimorbidity in this population.[3,17,56–59] Absolute numbers of physical health conditions were higher in patients with SSD than those with BD. Although direct comparisons require caution, our findings partially contrast with previous reports of higher number of physical comorbidities in individuals with BD.[3,38,39,41] Overall, the top ten most prevalent conditions in our SMI cohort were diabetes, hypertension, asthma, arthritis, epilepsy, cerebrovascular accident, eczema, migraine, ischaemic heart disease and COPD; and the most common combination of conditions included diabetes, hypertension and asthma, regardless of their SMI diagnoses. Moreover, those that had complex multimorbidity were also more likely to have cardiometabolic comorbidities such as diabetes and hypertension, which suggests that the cardiometabolic pathway might be one of the key explanatory mechanism underlying the association between physical multimorbidity and severe mental illness.[60,61] Future research should explore further these potential independent contribution of this pathway when focusing on individuals with complex multimorbidity. Furthermore, arthritis was the most frequent subsequent comorbidity for those with diabetes, hypertension and/or asthma, however for those with SSD and asthma, eczema slightly displaced arthritis in terms of prevalence. These findings might suggest that potential differences between SSD and BD phenotypes could be linked to underlying inflammatory pathways. Future research focusing on inflammatory biomarkers could be key to further our understanding of the potential differences between SSD and BD.

For peer review only

In addition, we examined the association between the top ten most prevalent conditions and disability levels. We found that not only multimorbidity was clearly associated with higher levels of disability but having any of these specific conditions was associated with higher levels of disability, even after adjustments for age, sex, deprivation or SMI diagnoses. Similar results were found when we further examined the associations between multimorbidity and disability within SSD and BD groups. When we examined the independent association of each physical health condition and disability within groups, our results suggested that that socio-demographic factors could have a greater impact in these associations in individuals with SSD. Although our results are not directly comparable with previous studies, they are in line with findings in previous research in ageing[62] or some specific SMI populations.[63,64] Further research is needed to understand the potential shared drivers of disability in individuals with BD and these conditions, in general, and diabetes and BD, in particular.

One of the main strengths of this study is the large comprehensive cohort of people with SMI drawn from a population with a high ethnic diversity, addressing the neglect of both ethnic minority groups and SMI in multimorbidity research. This is a key advantage of using EHRs from a large secondary mental health care provider and having the benefits of a data extraction strategy for accessing data on physical health conditions from the text fields of clinical notes. MedCAT development and deployment in CRIS text records will hopefully promote and facilitate future research in mental healthcare. However, further research is needed to validate this strategy in other EHRs sources using free text. Although our results are promising and the comparability of the findings with previous research provides some evidence of validity, further research is also needed to examine the cross-validity using primary care structured fields data. The present study is limited to individuals with SMI which does not allow us to compare comorbidity figures of SMI with other

For peer review only - http://bmjopen.bmj.com/site/about/guidelines.xhtml

common mental disorders and/or the general population. Future studies should replicate our data extraction strategy in other sex and age matched cohorts and explore the potential risks for subsequent health conditions maximizing the longitudinal nature of this data source. Furthermore, it is also important to note that individuals with more severe SMI may have more comprehensive textual data, so that our findings might be less representative of highly functioning individuals with less severe SMI. In addition, we acknowledge that although the conditions considered are within the most considered in multimorbidity research, future studies should consider a larger number of conditions and include rare diseases and all the conditions needed to calculate standard comorbidity scores such as Charlson or Exlihauser indexes which were not considered in this study (e.g., hemiplegia or paraplegia, peptic ulcer disease or AIDS/HIV). It should be noted that our study is one of the first, to our knowledge, to compare the associations between physical health comorbidities and disability in this traditionally neglected population and HoNOS is a widely used measure in secondary mental health services in the UK which provides us a general overview of disability in this population. However, we acknowledge that further research with more objective measures of disability is also needed to drive future policy in this population. To sum up, our study provides an overview of the most prevalent health conditions in SMI and underlines the need for further research into the origins of multimorbidity in this population, considering in more detail the nature of the SMI both in terms of severity and in terms of constituent diagnoses and/or symptomatic phenotype, given the apparent differences between BD and SSD. Our findings highlight multimorbidity as a driver of disability in this population, which also requires further mechanistic evaluation.

For peer review only

Acknowledgments: We would like to thank Megan Pritchard and Mathew Broadbent for their unvaluable contribution and support.

Funding statement:

RB is funded in part by grant MR/R016372/1 for the King's College London MRC Skills Development Fellowship programme funded by the UK Medical Research Council (MRC) and by grant IS-BRC-1215-20018 for the National Institute for Health Research (NIHR) Biomedical Research Centre at South London and Maudsley NHS Foundation Trust and King's College London. JD is funded by the Health Foundation working together with the Academy of Medical Sciences, for a Clinician Scientist Fellowship and by the ESRC in relation to the SEP-MD study [ES/S002715/1] and part supported by the ESRC Centre for Society and Mental Health at King's College London [ESRC Reference: ES/S012567/1]. DB is funded by a UKRI Innovation Fellowship as part of Health Data Research UK MR/S00310X/. AM is funded by Takeda California, Inc. RD, RS, AR are part-funded by the National Institute for Health Research (NIHR) Biomedical Research Centre at South London and Maudsley NHS Foundation Trust and King's College London. RD's work is supported by 1.National Institute for Health Research (NIHR) Biomedical Research Centre at South London and Maudsley NHS Foundation Trust and King's College London. 2. Health Data Research UK, which is funded by the UK Medical Research Council, Engineering and Physical Sciences Research Council, Economic and Social Research Council, Department of Health and Social Care (England), Chief Scientist Office of the Scottish Government Health and Social Care Directorates, Health and Social Care Research and Development Division (Welsh Government), Public Health Agency (Northern Ireland), British Heart Foundation and Wellcome Trust. 3. The National Institute for Health Research University College London Hospitals Biomedical Research Centre. This paper represents independent

For peer review only

research funded by the National Institute for Health Research (NIHR) Biomedical Research Centre at South London and Maudsley NHS Foundation Trust and King's College London and by the Health Foundation. The views expressed are those of the author(s) and not necessarily those of the NHS, the NIHR, the Department of Health, the MRC, ESRC or King's College London.

Authors contributions: RB conceived and designed the study. ZK, RB, AR and RS designed, validated the data extraction strategy. ZK, TS and AM developed the natural language processing algorithm and interface for the annotations. RB, ZK, JC, LM, NC, TS, AM, NS and TW were annotators. RB, ZK, SS, LL, JC, SA, RD and DB performed the data analyses and/or interpreted the results. RS and JD provided clinically relevant input over all the stages. RB, ZK and SS drafted the first version of the manuscript and all authors critically reviewed the manuscript and contributed to writing the final version.

Data sharing statement: Due to the confidential nature of free-text data, we are unable to make patient-level data available. CRIS was developed with extensive involvement from service users and adheres to strict governance frameworks managed by service users. It has passed a robust ethics approval pro-cess acutely attentive to the use of patient data. Specifically, this system was approved as a dataset for secondary data analysis on this basis by Oxfordshire Research Ethics Committee C (08/H06060/71). The data is de-identified and used in a data-secure format and all patients have the choice to opt-out of their anonymized data being used. Approval for data access can only be provided from the CRIS Oversight Committee at SLaM.

Competing interest statements: No competing interests.

Ethics statement: This project was approved by the CRIS Oversight Committee which is responsible for ensuring all research applications comply with ethical and legal guidelines. The

CRIS system enables access to anonymised electronic patient records for secondary analysis from SLaM and has full ethical approvals.

References

1 Firth J, Rosenbaum S, Galletly C, *et al.* Protecting physical health in people with mental illness – Authors' reply. *Lancet Psychiatry* 2019;**6**:890–1. doi:10.1016/S2215-0366(19)30387-6

2 Lawrence D, Kisely S, Pais J. The Epidemiology of Excess Mortality in People with Mental Illness. *Can J Psychiatry* 2010;**55**:752–60. doi:10.1177/070674371005501202

3 Reilly S, Olier I, Planner C, *et al.* Inequalities in physical comorbidity: a longitudinal comparative cohort study of people with severe mental illness in the UK. *BMJ Open* 2015;**5**:e009010. doi:10.1136/bmjopen-2015-009010

4 Woodhead C, Ashworth M, Schofield P, *et al.* Patterns of physical co-/multi-morbidity among patients with serious mental illness: a London borough-based cross-sectional study. *BMC Fam Pract* 2014;**15**:117. doi:10.1186/1471-2296-15-117

5 Filipcic IŠ, Bajic Ž, Filipcic I. The onset and accumulation of physical multimorbidity in severe and common mental disorders. *Curr Opin Psychiatry* 2020;**33**:484–90. doi:10.1097/YCO.0000000000000635

6 Cheung KL, ten Klooster PM, Smit C, *et al.* The impact of non-response bias due to sampling in public health studies: A comparison of voluntary versus mandatory recruitment in a Dutch national survey on adolescent health. *BMC Public Health* 2017;**17**. doi:10.1186/s12889-017-4189-8

For peer review only

7 Correll CU, Detraux J, Lepeleire JD, *et al.* Effects of antipsychotics, antidepressants and mood stabilizers on risk for physical diseases in people with schizophrenia, depression and bipolar disorder. *World Psychiatry* 2015;**14**:119–36. doi:https://doi.org/10.1002/wps.20204

8 Correll CU, Solmi M, Veronese N, *et al.* Prevalence, incidence and mortality from cardiovascular disease in patients with pooled and specific severe mental illness: a large-scale meta-analysis of 3,211,768 patients and 113,383,368 controls. *World Psychiatry* 2017;**16**:163–80. doi:https://doi.org/10.1002/wps.20420

9 DE HERT M, CORRELL CU, BOBES J, *et al.* Physical illness in patients with severe mental disorders. I. Prevalence, impact of medications and disparities in health care. *World Psychiatry* 2011;**10**:52–77.

10 Fan Z, Wu Y, Shen J, *et al.* Schizophrenia and the risk of cardiovascular diseases: A meta-analysis of thirteen cohort studies. *J Psychiatr Res* 2013;**47**:1549–56. doi:10.1016/j.jpsychires.2013.07.011

11 Pouget JG, Schizophrenia Working Group of the Psychiatric Genomics Consortium, Han B, *et al.* Cross-disorder analysis of schizophrenia and 19 immune-mediated diseases identifies shared genetic risk. *Hum Mol Genet* 2019;**28**:3498–513. doi:10.1093/hmg/ddz145

12 Rosenblat JD, McIntyre RS. Are medical comorbid conditions of bipolar disorder due to immune dysfunction? *Acta Psychiatr Scand* 2015;**132**:180–91. doi:https://doi.org/10.1111/acps.12414

13 Tylee DS, Sun J, Hess JL, *et al.* Genetic correlations among psychiatric and immune-related phenotypes based on genome-wide association data. *Am J Med Genet B Neuropsychiatr Genet* 2018;**177**:641–57. doi:https://doi.org/10.1002/ajmg.b.32652

For peer review only

14 Leo RJ, Singh J. Migraine headache and bipolar disorder comorbidity: A systematic review of the literature and clinical implications. *Scand J Pain* 2016;**11**:136–45. doi:10.1016/j.sjpain.2015.12.002

15 McKay KA, Tremlett H, Fisk JD, *et al.* Psychiatric comorbidity is associated with disability progression in multiple sclerosis. *Neurology* 2018;**90**:e1316–23. doi:10.1212/WNL.0000000000005302

16 Dorrington S, Carr E, Stevelink SAM, *et al.* Multimorbidity and fit note receipt in working-age adults with long-term health conditions. *Psychol Med* undefined/ed;:1–10. doi:10.1017/S0033291720002937

17 Kugathasan P, Wu H, Gaughran F, *et al.* Association of physical health multimorbidity with mortality in people with schizophrenia spectrum disorders: Using a novel semantic search system that captures physical diseases in electronic patient records. *Schizophr Res* 2020;**216**:408–15. doi:10.1016/j.schres.2019.10.061

18 Barnett K, Mercer SW, Norbury M, *et al.* Epidemiology of multimorbidity and implications for health care, research, and medical education: a cross-sectional study. *The Lancet* 2012;**380**:37–43. doi:10.1016/S0140-6736(12)60240-2

19 Cassell A, Edwards D, Harshfield A, *et al.* The epidemiology of multimorbidity in primary care: a retrospective cohort study. *Br J Gen Pract* 2018;**68**:e245–51. doi:10.3399/bjgp18X695465

20 Kuan V, Denaxas S, Gonzalez-Izquierdo A, *et al.* A chronological map of 308 physical and mental health conditions from 4 million individuals in the English National Health Service. *Lancet Digit Health* 2019;**1**:e63–77. doi:10.1016/S2589-7500(19)30012-3

For peer review only

21 Altamura AC, Buoli M, Pozzoli S. Role of immunological factors in the pathophysiology and diagnosis of bipolar disorder: Comparison with schizophrenia. *Psychiatry Clin Neurosci* 2014;**68**:21–36. doi:https://doi.org/10.1111/pcn.12089

22 Sahu A, Chowdhury HA, Gaikwad M, *et al.* Integrative network analysis identifies differential regulation of neuroimmune system in Schizophrenia and Bipolar disorder. *Brain Behav Immun - Health* 2020;**2**:100023. doi:10.1016/j.bbih.2019.100023

23 Walker J, Curtis V, Shaw P, *et al.* Schizophrenia and bipolar disorder are distinguished mainly by differences in neurodevelopment. *Neurotox Res* 2002;**4**:427. doi:10.1080/1029842021000022070

24 WHO | The world health report 2001 - Mental Health: New Understanding, New Hope. WHO. https://www.who.int/whr/2001/en/ (accessed 4 Jun 2021).

25 Prediction of disability in schizophrenia: Symptoms, cognition, and self-assessment - Philip D. Harvey, Martin T. Strassnig, Juliet Silberstein, 2019. https://journals.sagepub.com/doi/full/10.1177/2043808719865693 (accessed 4 Jun 2021).

26 Sanchez-Moreno J, Martinez-Aran A, Tabarés-Seisdedos R, *et al.* Functioning and Disability in Bipolar Disorder: An Extensive Review. *Psychother Psychosom* 2009;**78**:285–97. doi:10.1159/000228249

27 Stewart R, Soremekun M, Perera G, *et al.* The South London and Maudsley NHS Foundation Trust Biomedical Research Centre (SLAM BRC) case register: development and descriptive data. *BMC Psychiatry* 2009;**9**:51. doi:10.1186/1471-244X-9-51

28 ICD-10 Version:2016. https://icd.who.int/browse10/2016/en (accessed 9 Jun 2021).

For peer review only

29 Kraljevic Z, Searle T, Shek A, *et al.* Multi-domain Clinical Natural Language Processing with MedCAT: the Medical Concept Annotation Toolkit. *ArXiv201001165 Cs* Published Online First: 25 March 2021.http://arxiv.org/abs/2010.01165 (accessed 4 Jun 2021).

30 Searle T, Kraljevic Z, Bendayan R, *et al.* MedCATTrainer: A Biomedical Free Text Annotation Interface with Active Learning and Research Use Case Specific Customisation. *ArXiv190707322 Cs* Published Online First: 16 July 2019.http://arxiv.org/abs/1907.07322 (accessed 19 Aug 2020).

31 English indices of deprivation 2019: technical report. GOV.UK. https://www.gov.uk/government/publications/english-indices-of-deprivation-2019-technical-report (accessed 4 Jun 2021).

32 Wing JK, Beevor AS, Curtis RH, *et al.* Health of the Nation Outcome Scales (HoNOS): Research and development. *Br J Psychiatry* 1998;**172**:11–8. doi:10.1192/bjp.172.1.11

33 Jewell A, Pritchard M, Barrett K, *et al.* The Maudsley Biomedical Research Centre (BRC) data linkage service user and carer advisory group: creating and sustaining a successful patient and public involvement group to guide research in a complex area. *Res Involv Engagem* 2019;**5**:20. doi:10.1186/s40900-019-0152-4

34 Bean DM, Kraljevic Z, Searle T, *et al.* Angiotensin-converting enzyme inhibitors and angiotensin II receptor blockers are not associated with severe COVID-19 infection in a multi-site UK acute hospital trust. *Eur J Heart Fail* 2020;**22**:967–74. doi:https://doi.org/10.1002/ejhf.1924

35 Carr E, Bendayan R, Bean D, *et al.* Evaluation and improvement of the National Early Warning Score (NEWS2) for COVID-19: a multi-hospital study. *BMC Med* 2021;**19**:23. doi:10.1186/s12916-020-01893-3

For peer review only

36 Birgenheir DG, Ilgen MA, Bohnert ASB, *et al.* Pain conditions among veterans with schizophrenia or bipolar disorder. *Gen Hosp Psychiatry* 2013;**35**:480–4. doi:10.1016/j.genhosppsych.2013.03.019

37 Hardoon S, Hayes JF, Blackburn R, *et al.* Recording of Severe Mental Illness in United Kingdom Primary Care, 2000–2010. *PLOS ONE* 2013;**8**:e82365. doi:10.1371/journal.pone.0082365

38 Smith DJ, Langan J, McLean G, *et al.* Schizophrenia is associated with excess multiple physical-health comorbidities but low levels of recorded cardiovascular disease in primary care: cross-sectional study. *BMJ Open* 2013;**3**:e002808. doi:10.1136/bmjopen-2013-002808

39 Smith DJ, Martin D, McLean G, *et al.* Multimorbidity in bipolar disorder and undertreatment of cardiovascular disease: a cross sectional study. *BMC Med* 2013;**11**:263. doi:10.1186/1741-7015-11-263

40 Halvorsrud K, Nazroo J, Otis M, *et al.* Ethnic inequalities in the incidence of diagnosis of severe mental illness in England: a systematic review and new meta-analyses for non-affective and affective psychoses. *Soc Psychiatry Psychiatr Epidemiol* 2019;**54**:1311–23. doi:10.1007/s00127-019-01758-y

41 Oreški I, Jakovljević M, Aukst-Margetić B, *et al.* Comorbidity and multimorbidity in patients with schizophrenia and bipolar disorder: similarities and differencies. *Psychiatr Danub* 2012;**24**:80–5.

42 Patel RS, Virani S, Saeed H, *et al.* Gender Differences and Comorbidities in U.S. Adults with Bipolar Disorder. *Brain Sci* 2018;**8**:168. doi:10.3390/brainsci8090168

43 Severe mental illness (SMI) and physical health inequalities: briefing. GOV.UK. https://www.gov.uk/government/publications/severe-mental-illness-smi-physical-health-

For peer review only

inequalities/severe-mental-illness-and-physical-health-inequalities-briefing (accessed 4 Jun 2021).

44 Das-Munshi J, Ashworth M, Dewey ME, *et al.* Type 2 diabetes mellitus in people with severe mental illness: inequalities by ethnicity and age. Cross-sectional analysis of 588 408 records from the UK. *Diabet Med* 2017;**34**:916–24. doi:https://doi.org/10.1111/dme.13298

45 Gaughran F, Stahl D, Stringer D, *et al.* Effect of lifestyle, medication and ethnicity on cardiometabolic risk in the year following the first episode of psychosis: prospective cohort study. *Br J Psychiatry* 2019;**215**:712–9. doi:10.1192/bjp.2019.159

46 Lusignan S de, Alexander H, Broderick C, *et al.* The epidemiology of eczema in children and adults in England: A population-based study using primary care data. *Clin Exp Allergy* 2021;**51**:471–82. doi:https://doi.org/10.1111/cea.13784

47 Gilkes A, Ashworth M, Schofield P, *et al.* Does COPD risk vary by ethnicity? A retrospective cross-sectional study. *Int J Chron Obstruct Pulmon Dis* 2016;**11**:739–46. doi:10.2147/COPD.S96391

48 Godsland IF, Johnston DG, Chaturvedi N. Mechanisms of Disease: lessons from ethnicity in the role of triglyceride metabolism in ischemic heart disease. *Nat Clin Pract Endocrinol Metab* 2007;**3**:530–8. doi:10.1038/ncpendmet0530

49 Health Survey for England - 2004: Health of ethnic minorities, Headline results. NHS Digit. https://digital.nhs.uk/data-and-information/publications/statistical/health-survey-for-england/health-survey-for-england-2004-health-of-ethnic-minorities-headline-results (accessed 4 Jun 2021).

50 Public Health Profiles - PHE. https://fingertips.phe.org.uk/ (accessed 4 Jun 2021).

For peer review only

51 Zghebi SS, Steinke DT, Carr MJ, *et al.* Examining trends in type 2 diabetes incidence, prevalence and mortality in the UK between 2004 and 2014. *Diabetes Obes Metab* 2017;**19**:1537–45. doi:https://doi.org/10.1111/dom.12964

52 Diabetes Prevention Programme, 2017-18 Diagnoses and Demographics. NHS Digit. https://digital.nhs.uk/data-and-information/publications/statistical/national-diabetes-audit/diabetes-prevention-programme-2017-18 (accessed 4 Jun 2021).

53 Leng B, Jin Y, Li G, *et al.* Socioeconomic status and hypertension: a meta-analysis. *J Hypertens* 2015;**33**:221–9. doi:10.1097/HJH.0000000000000428

54 Gupta RP, Mukherjee M, Sheikh A, *et al.* Persistent variations in national asthma mortality, hospital admissions and prevalence by socioeconomic status and region in England. *Thorax* 2018;**73**:706–12. doi:10.1136/thoraxjnl-2017-210714

55 Simpson CR, Hippisley-Cox J, Sheikh A. Trends in the epidemiology of chronic obstructive pulmonary disease in England: a national study of 51 804 patients. *Br J Gen Pract* 2010;**60**:e277–84. doi:10.3399/bjgp10X514729

56 Carney CP, Jones L, Woolson RF. Medical comorbidity in women and men with schizophrenia. *J Gen Intern Med* 2006;**21**:1133–7. doi:10.1111/j.1525-1497.2006.00563.x

57 Gabilondo A, Alonso-Moran E, Nuño-Solinis R, *et al.* Comorbidities with chronic physical conditions and gender profiles of illness in schizophrenia. Results from PREST, a new health dataset. *J Psychosom Res* 2017;**93**:102–9. doi:10.1016/j.jpsychores.2016.12.011

58 Kugathasan P, Stubbs B, Aagaard J, *et al.* Increased mortality from somatic multimorbidity in patients with schizophrenia: a Danish nationwide cohort study. *Acta Psychiatr Scand* 2019;**140**:340–8. doi:10.1111/acps.13076

For peer review only

59 Severe mental illness (SMI) and physical health inequalities: briefing. GOV.UK. https://www.gov.uk/government/publications/severe-mental-illness-smi-physical-health-inequalities/severe-mental-illness-and-physical-health-inequalities-briefing (accessed 3 Mar 2020).

60 Firth J, Siddiqi N, Koyanagi A, *et al.* The Lancet Psychiatry Commission: a blueprint for protecting physical health in people with mental illness. *Lancet Psychiatry* 2019;**6**:675–712. doi:10.1016/S2215-0366(19)30132-4

61 Henderson DC, Vincenzi B, Andrea NV, *et al.* Pathophysiological mechanisms of increased cardiometabolic risk in people with schizophrenia and other severe mental illnesses. *Lancet Psychiatry* 2015;**2**:452–64. doi:10.1016/S2215-0366(15)00115-7

62 Quiñones AR, Markwardt S, Botoseneanu A. Multimorbidity Combinations and Disability in Older Adults. *J Gerontol A Biol Sci Med Sci* 2016;**71**:823–30. doi:10.1093/gerona/glw035

63 Desalegn D, Girma S, Abdeta T. Quality of life and its association with current substance use, medication non-adherence and clinical factors of people with schizophrenia in Southwest Ethiopia: a hospital-based cross-sectional study. *Health Qual Life Outcomes* 2020;**18**. doi:10.1186/s12955-020-01340-0

64 McIntyre RS, Konarski JZ, Soczynska JK, *et al.* Medical Comorbidity in Bipolar Disorder: Implications for Functional Outcomes and Health Service Utilization. *Psychiatr Serv* 2006;**57**:1140–4. doi:10.1176/ps.2006.57.8.1140

For peer review only

Table 1. MedCAT performance F1, precision and recall estimates for each physical health conditions.

**Physical Health Condition**	**F1**	**Precision**	**Recall**
Diabetes mellitus	0.98	0.99	0.98
Heart failure	0.97	0.97	0.96
Ischemic heart disease	0.98	0.97	0.99
Hypertensive disorder, systemic arterial	0.97	0.97	0.96
Chronic obstructive lung disease	0.94	0.97	0.92
Asthma	1.00	1.00	1.00
Chronic kidney disease	1.00	1.00	0.99
Cerebrovascular accident	0.96	0.94	0.98
Transient ischemic attack	0.91	0.82	1.00
Parkinson's disease	0.94	0.88	1.00
Multiple sclerosis	1.00	1.00	1.00
Epilepsy	0.93	1.00	0.85
Migraine	1.00	1.00	1.00
Atrial fibrillation	0.98	1.00	0.96
Chronic sinusitis	0.98	0.97	1.00
Inflammatory bowel disease	0.96	1.00	0.92
Chronic liver disease	1.00	1.00	1.00
Psoriasis	1.00	1.00	1.00
Eczema	0.94	1.00	0.88
Arthritis	1.00	1.00	1.00

For peer review only

Table 2. Socio-demographic characteristics and prevalence for physical health conditions for total cohort (N=17500) and by SMI diagnosis.

	Total	SSD	BD
**N (%)**	17500	13019 (74.4)	4481 (25.6)
**Sex*** **			
Female	8123 (46.4)	5421 (41.6)	2702 (60.3)
Male	9374 (53.6)	7596 (58.3)	1778 (39.7)
**Age at first SMI diagnosis*** **			
15 – 34	7497 (42.8)	5607 (43.1)	1890 (42.2)
35 – 44	3736 (21.3)	2792 (21.4)	944 (21.1)
45 – 54	2783 (15.9)	2057 (15.8)	726 (16.2)
55 – 64	1525 (8.7)	1067 (8.2)	458 (10.2)
65+	1959 (11.2)	1496 (11.5)	463 (10.3)
**Ethnicity*** **			
White British	6243 (35.7)	4008 (30.8)	2235 (49.9)
Black Caribbean	3182 (18.2)	2799 (21.5)	383 (8.5)
Black African	2094 (12.0)	1886 (14.5)	208 (4.6)
South Asian	549 (3.1)	421 (3.2)	128 (2.9)
Irish	346 (2.0)	240 (1.8)	106 (2.4)
Other	3846 (22.0)	2822 (21.7)	1024 (22.9)
Not stated	1240 (7.1)	843 (6.5)	397 (8.9)
**Index of multiple deprivation*** **			
1 (less deprived)	742 (4.2)	412 (3.2)	330 (7.4)
2	1384 (7.9)	887 (6.8)	497 (11.1)
3	3575 (20.4)	2503 (19.2)	1072 (23.9)
4	7073 (40.4)	5476 (42.1)	1597 (35.6)
5 (more deprived)	4033 (23.0)	3204 (24.6)	829 (18.5)
Unknown	693 (4.0)	537 (4.1)	156 (3.5)
**Number of conditions*** **			
No mentions	10468 (59.8)	7540 (57.9)	2928 (65.3)
One	3733 (21.3)	2888 (22.2)	845 (18.9)
Two	1795 (10.3)	1429 (11.0)	366 (8.2)
Three or more	1504 (8.6)	1162 (8.9)	342 (7.6)
**Physical conditions*** **			
Diabetes***	2686 (15.3)	2208 (17.0)	478 (10.7)
Hypertension***	2537 (14.5)	2070 (15.9)	467 (10.4)
Asthma	1722 (9.8)	1291 (9.9)	431 (9.6)
Arthritis	954 (5.5)	702 (5.4)	252 (5.6)
Epilepsy***	799 (4.6)	652 (5.0)	147 (3.3)
Cerebrovascular accident	728 (4.2)	573 (4.4)	155 (3.5)
Eczema	616 (3.5)	479 (3.7)	137 (3.1)

For peer review only

Migraine***	564 (3.2)	372 (2.9)	192 (4.3)
Ischemic heart disease	561 (3.2)	435 (3.3)	126 (2.8)
Chronic Obstructive Pulmonary disease	476 (2.7)	342 (2.6)	134 (3.0)
Chronic kidney disease*	279 (1.6)	179 (1.4)	100 (2.2)
Parkinson's disease	266 (1.5)	201 (1.5)	65 (1.5)
Heart failure**	222 (1.3)	187 (1.4)	35 (0.8)
Psoriasis	179 (1.0)	129 (1.0)	50 (1.1)
Atrial fibrillation	133 (0.8)	100 (0.8)	33 (0.7)
Transient Ischaemic Attack	130 (0.7)	91 (0.7)	39 (0.9)
Inflammatory Bowel Disease	40 (0.2)	25 (0.2)	15 (0.3)
Multiple sclerosis	32 (0.2)	19 (0.1)	13 (0.3)
Chronic liver disease	22 (0.1)	20 (0.2)	2 (0.0)
Chronic sinusitis	6 (0.0)	6 (0.0)	0 (0.0)

Note. *** $p < .001$; ** $p < .01$; * $p < .05$ for comparisons between BD and SSD groups

For peer review only

Table 3. Ten most prevalent conditions and associated comorbidities of the total cohort and by SMI diagnosis.

Condition		Preva-lence	Mean # of comorbidities	Three most frequent associated comorbidities		
	Total	-	0.74	1. Diabetes (15.3%)	2. HTN (14.5%)	3. Asthma (9.8%)
	SSD	-	0.77	1. Diabetes (17.0%)	2. HTN (15.9%)	3. Asthma (9.9%)
	BD	-	0.64	1. Diabetes (10.7%)	2. HTN (10.4%)	3. Asthma (9.6%)
**Diabetes**	*Total*	15.3%	2.35	1. HTN (42.1%)	2. Asthma (16.9%)	3. Arthritis (13.2%)
	SSD	17.0%	2.33	1. HTN (42.9%)	2. Asthma (16.3%)	3. Arthritis (12.7%)
	BD	10.7%	2.45	1. HTN (38.3%)	2. Asthma (19.5%)	3. Arthritis (15.5%)
**Hypertension**	*Total*	14.5%	2.50	1. Diabetes (44.5%)	2. Asthma (16.3%)	3. Arthritis (15.2%)
	SSD	15.9%	2.47	1. Diabetes (45.7%)	2. Asthma (16.1%)	3. Arthritis (14.7%)
	BD	10.4%	2.62	1. Diabetes (39.2%)	2. Arthritis (17.3 %)	3. Asthma (17.1%)
**Asthma**	*Total*	9.8%	2.27	1. Diabetes (26.3%)	2. HTN (24.0%)	3. Arthritis (11.9%)
	SSD	9.9%	2.27	1. Diabetes (27.9%)	2. HTN (25.9%)	3. Eczema (11.2%)
	BD	9.6%	2.29	1. Diabetes (21.6%)	2. HTN (18.6%)	3. Arthritis (15.1%)
**Arthritis**	*Total*	5.5%	2.78	1. HTN (40.5%)	2. Diabetes (37.1%)	3. Asthma (21.5%)
	SSD	5.4%	2.78	1. HTN (43.4%)	2. Diabetes (39.9%)	3. Asthma (19.9%)
	BD	5.6%	2.80	1. HTN (32.1%)	2. Diabetes (29.4%)	3. Asthma (25.8%)
**Epilepsy**	*Total*	4.6%	2.40	1. HTN (25.5%)	2. Diabetes (24.8%)	3. Asthma (21.4%)
	SSD	5.0%	2.42	1. HTN (27.3%)	2. Diabetes (26.4%)	3. Asthma (21.8%)
	BD	3.3%	2.31	1. Asthma (19.7%)	2. Diabetes (17.7%)	2. HTN (17.7%)
**CVA**	*Total*	4.2%	2.89	1. HTN (42.3%)	2. Diabetes (38.6%)	3. Asthma (15.4%)
	SSD	4.4%	2.83	1. HTN (42.8%)	2. Diabetes (38.9%)	3. Asthma (14.0%)
	BD	3.5%	3.09	1. HTN (40.6%)	2. Diabetes (37.4%)	3. Asthma (21.9)
**Eczema**	*Total*	3.5%	2.50	1. Asthma (32.5%)	2. Diabetes (26.9%)	3. HTN (23.1%)
	SSD	3.7%	2.45	1. Asthma (30.3%)	2. Diabetes (28.4%)	3. HTN (22.5%)
	BD	3.1%	2.64	1. Asthma (40.1%)	2. HTN (24.8%)	3. Diabetes (21.9%)
**Migraine**	*Total*	3.2%	2.20	1. Asthma (23.6%)	2. Diabetes (20.0%)	3. HTN (18.4%)
	SSD	2.9%	2.23	1. Diabetes (23.4%)	2. Asthma (21.5%)	3. HTN (20.2%)
	BD	4.3%	2.15	1. Asthma (27.6%)	2. HTN (15.1%)	3. Arthritis (14.1%)
**Ischaemic heart disease**	*Total*	3.2%	3.27	1. HTN (49.0%)	2. Diabetes (43.3%)	3. Asthma (20.3%)
	SSD	3.3%	3.28	1. HTN (51.0%)	2. Diabetes (44.6%)	3. Asthma (20.2%)
	BD	2.8%	3.24	1. HTN (42.1%)	2. Diabetes (38.9%)	3. Arthritis (22.2%)
**COPD**	*Total*	2.7%	3.22	1. HTN (39.7%)	2. Diabetes (38.9%)	3. Asthma (35.7%)
	SSD	2.6%	3.20	1. Diabetes (41.2%)	2. HTN (39.2%)	3. Asthma (35.7%)
	BD	3.0%	3.25	1. HTN (41.0%)	2. Asthma (35.8%)	3. Diabetes (32.8%)

Note. SSD: Schizophrenia Spectrum disorders. BD: Bipolar disorders. HTN: hypertension

Table 4. Associations between specific comorbidities, multimorbidity and HoNOS scores. Models were adjusted by age and sex (Model 1), and then additionally adjusted by IMD (Model 2a) or SMI diagnosis (Model 2b).

	HoNOS	Unadjusted	Model 1	Model 2a	Model 2b
	Mean (SD)	B (95% CI)	B (95% CI)	B (95% CI)	B (95% CI)
**Physical comorbidities**					
**Diabetes**	10.93 (6.08)	0.652 (0.389 – 0.914)***	0.464 (0.198 – 0.730)***	0.485 (0.215 – 0.755)***	0.359 (0.094 – 0.625)**
Ref: No diabetes	10.28 (6.05)				
**Hypertension**	10.99 (6.02)	0.713 (0.446 – 0.980)***	0.401 (0.123 – 0.680)**	0.368 (0.084 – 0.652)*	0.297 (0.019 – 0.576)*
Ref: No hypertension	10.27 (6.06)				
**Asthma**	11.05 (6.16)	0.734 (0.417 – 1.051)***	0.806 (0.490 – 1.121)***	0.770 (0.450 – 1.091)***	0.804 (0.489 – 1.118)***
Ref: No asthma	10.31 (6.04)				
**Arthritis**	12.01 (6.19)	1.728 (1.322 – 2.134)***	1.570 (1.156 – 1.984)***	1.558 (1.142 – 1.974)***	1.567 (1.155 – 1.980)***
Ref: No arthritis	10.28 (6.03)				
**Epilepsy**	11.40 (6.19)	1.055 (0.596 – 1.514)***	1.066 (0.609 – 1.523)***	1.089 (0.628 – 1.549)***	0.982 (0.527 – 1.437)***
Ref: No epilepsy	10.35 (6.05)				
**CVA**	11.81 (6.22)	1.487 (1.022 – 1.951)***	1.195 (0.728 – 1.662)***	1.180 (0.712 – 1.649)***	1.162 (0.697 – 1.627)***
Ref: No CVA	10.33 (6.04)				
**Eczema**	11.12 (6.19)	0.753 (0.249 – 1.257)**	0.838 (0.336 – 1.340)***	0.728 (0.220 – 1.237)**	0.799 (0.299 – 1.299)**
Ref: No eczema	10.37 (6.05)				
**Migraine**	10.33 (5.73)	-0.078 (-0.600 – 0.445)	0.212 (-0.311 – 0.734)	0.227 (-0.300 – 0.754)	0.302 (-0.218 – 0.823)
Ref: No migraine	10.40 (6.07)				
**Ischaemic heart disease**	11.64 (5.92)	1.294 (0.766 – 1.822)***	0.864 (0.332 – 1.395)***	0.853 (0.318 – 1.387)**	0.849 (0.320 – 1.379)**
Ref: No ischaemic heart disease	10.35 (6.06)				
**COPD**	12.08 (5.78)	1.733 (1.158 – 2.309)***	1.326 (0.745 – 1.908)***	1.336 (0.750 – 1.923)***	1.397 (0.818 – 1.977)***
Ref: No COPD	10.34 (6.06)				
**Multimorbidity**					

**Number of comorbidities**		0.487 (0.406 – 0.567)***	0.423 (0.339 – 0.507)***	0.424 (0.339 – 0.510) ***	0.404 (0.320 – 0.488)***
**1 or more comorbidities**	10.96 (6.08)	1.053 (0.850 – 1.256)***	0.893 (0.685 – 1.101)***	0.895 (0.681 – 1.109)***	0.823 (0.615 – 1.031)***
Ref: No comorbidities	9.90 (5.99)				
**2 or more comorbidities**	11.34 (6.13)	1.214 (0.973 – 1.455)***	1.021 (0.772 – 1.269)***	1.020 (0.768 – 1.272)***	0.968 (0.720 – 1.216)***
Ref: Less than 1 comorbidities	10.12 (6.01)				

Note. *** p < .001; ** p < .01; * p < .05

For peer review only

Figure 1. Comparison of number of physical health comorbidities (a) and specific physical comorbidities (b) by SMI diagnosis.

Figure 2. Prevalence of the most prevalent physical health conditions across ethnicities within the SMI cohort and the SSD and BD subgroups.

Figure 3. Visualization of most prevalent comorbidities in individuals with SMI and comorbid diabetes.

Figure 4. Visualization of most prevalent comorbidities in individuals with SMI and comorbid hypertension.

Figure 5. Visualization of most prevalent comorbidities in individuals with SMI and comorbid asthma.

For peer review only

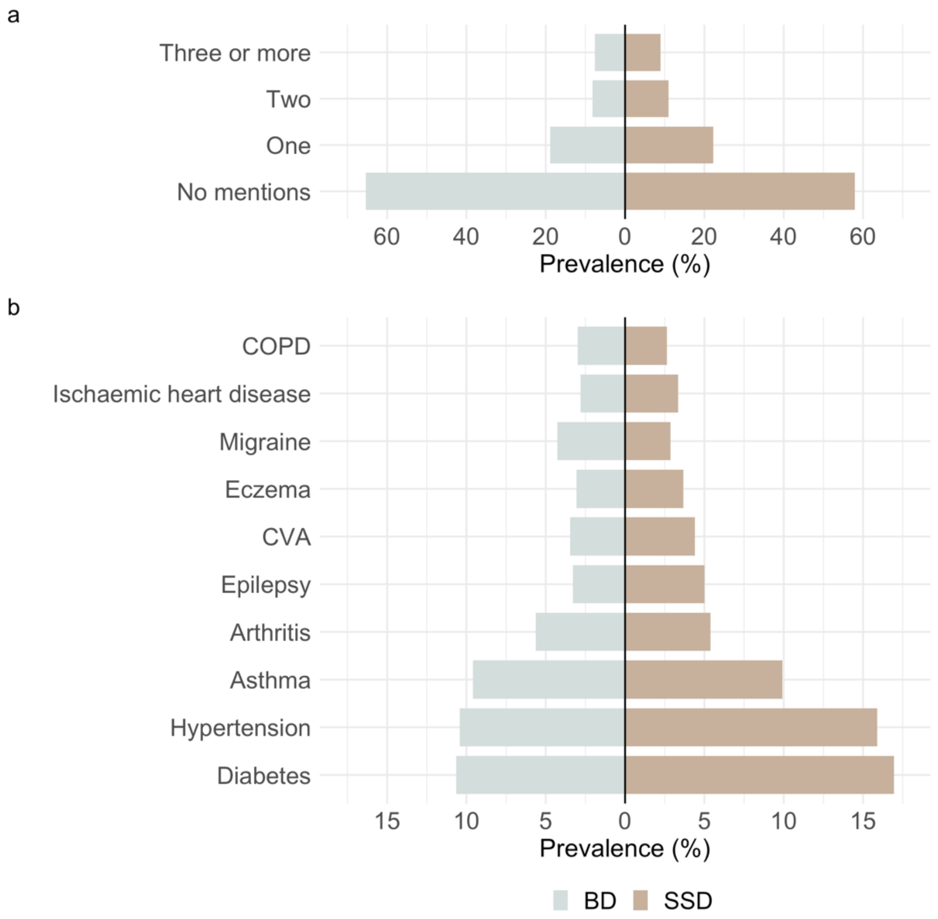

For peer review only

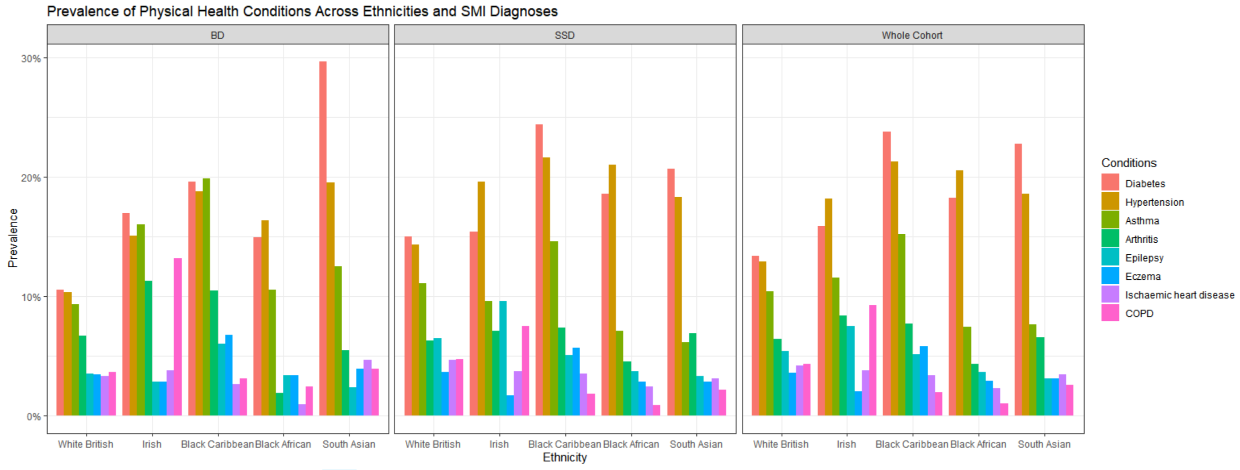

Figure 3. Visualization of most prevalent comorbidities in individuals with SMI and comorbid diabetes.

For peer review only

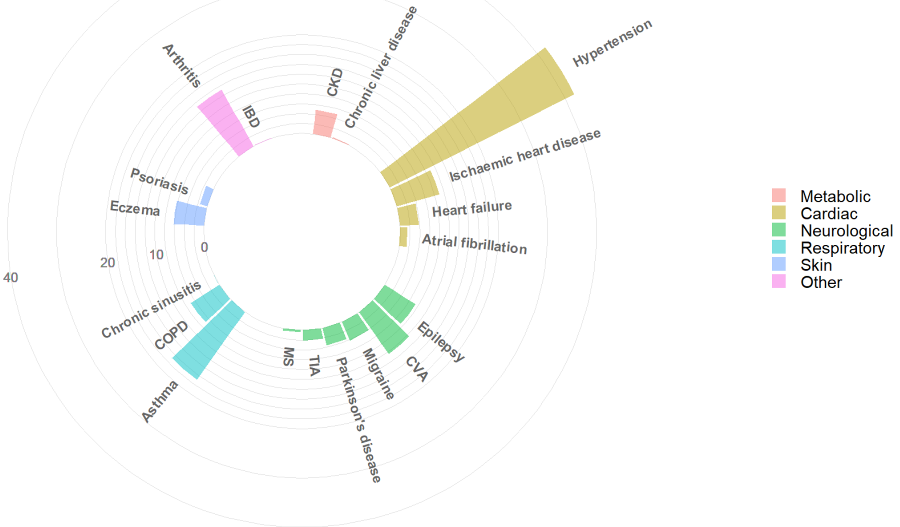

Figure 4. Visualization of most prevalent comorbidities in individuals with SMI and comorbid hypertension.

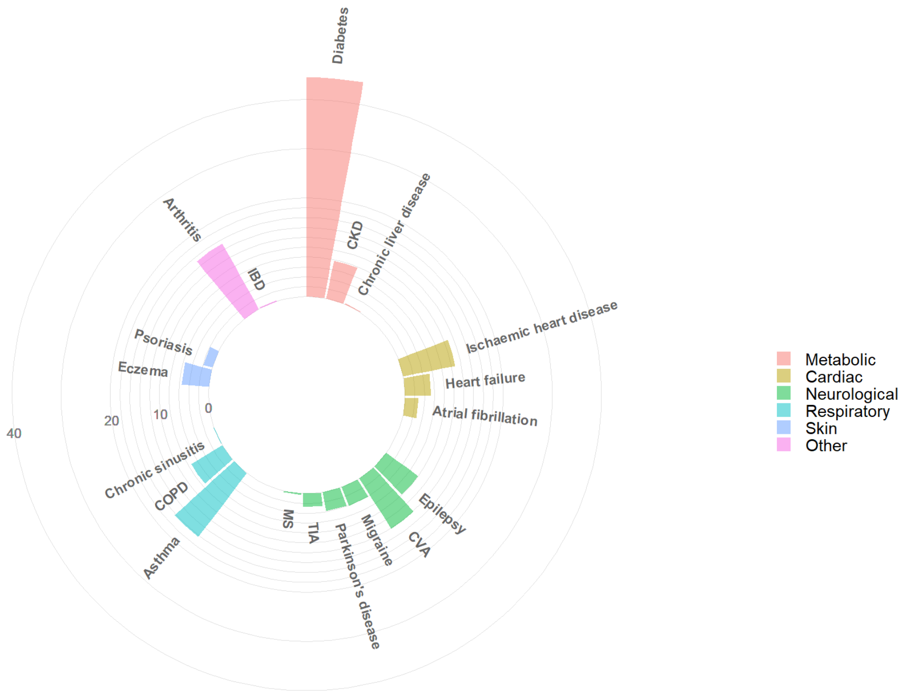

Figure 5. Visualization of most prevalent comorbidities in individuals with SMI and comorbid asthma.

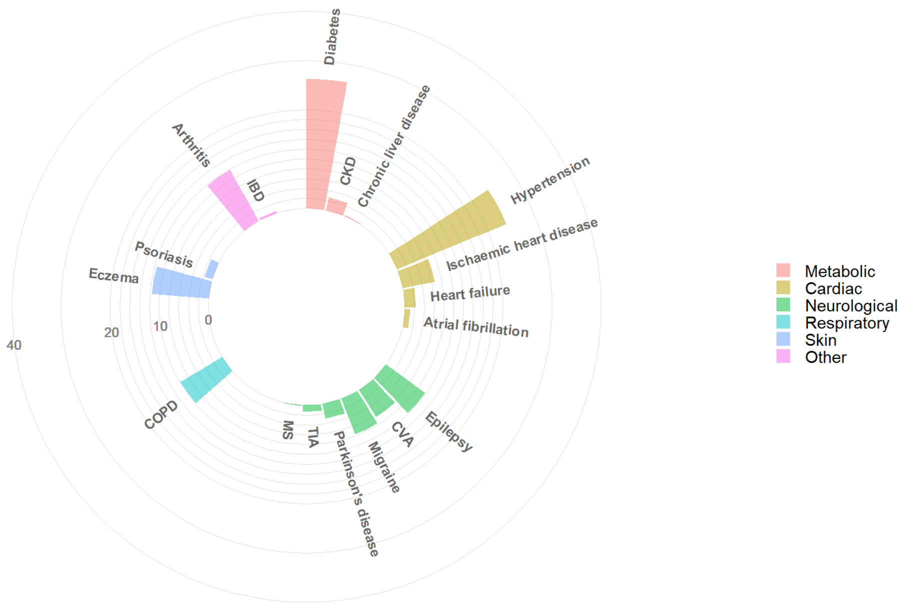

For peer review only

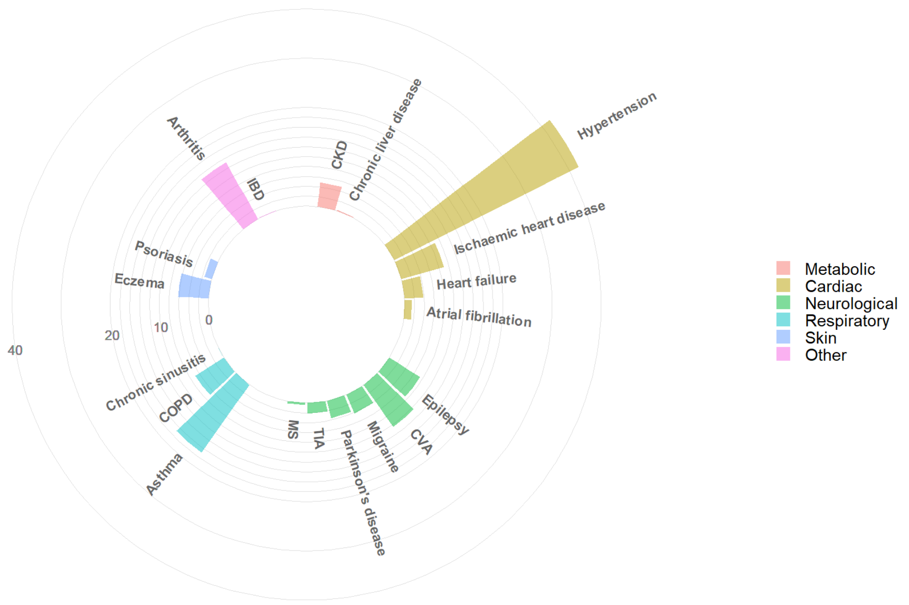

Hypertension
Ischaemic heart disease
Heart failure
Atrial fibrillation
Epilepsy
CVA
Migraine
Parkinson's disease
TIA
MS
Asthma
COPD
Chronic sinusitis
Eczema
Psoriasis
IBD
Arthritis
CKD
Chronic liver disease
0
10
20
40
Metabolic
Cardiac
Neurological
Respiratory
Skin
Other

review only

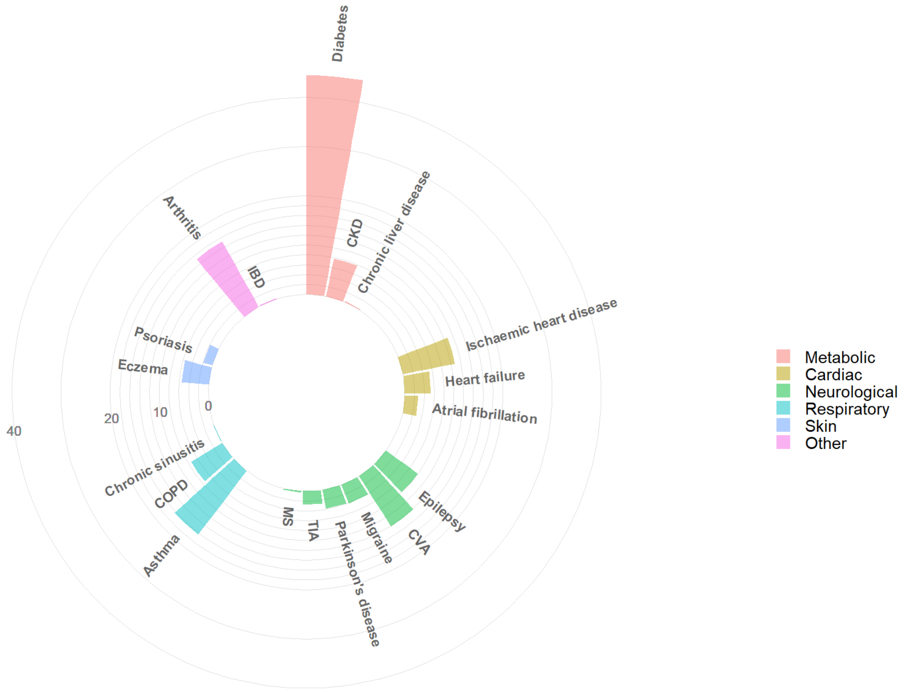
Diabetes
CKD
Chronic liver disease
Ischaemic heart disease
Heart failure
Atrial fibrillation
Epilepsy
CVA
Migraine
Parkinson's disease
TIA
MS
Asthma
COPD
Chronic sinusitis
Eczema
Psoriasis
IBD
Arthritis
40
20
10
0
Metabolic
Cardiac
Neurological
Respiratory
Skin
Other

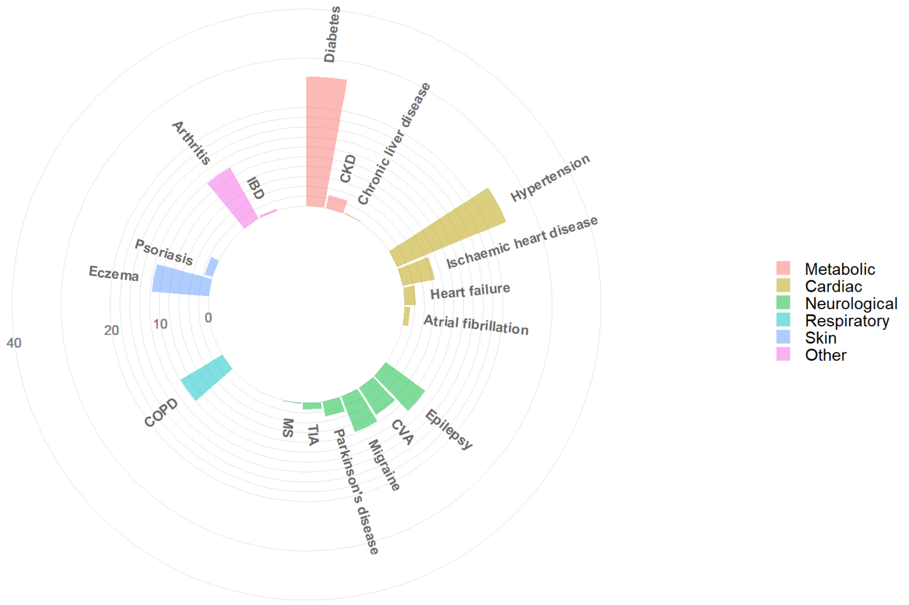
Diabetes
CKD
Chronic liver disease
Hypertension
Ischaemic heart disease
Heart failure
Atrial fibrillation
Epilepsy
CVA
Migraine
Parkinson's disease
TIA
MS
COPD
Eczema
Psoriasis
IBD
Arthritis
0
10
20
40
Metabolic
Cardiac
Neurological
Respiratory
Skin
Other

review only

Supplemental Material

Supplemental Tables

Supplemental Table 1. Meta-annotations performance results for Diagnosis and Status

Supplemental Table 2. Prevalence estimates and sex differences within the whole SMI cohort and SSD and BD subgroups.

Supplemental Table 3. Prevalence for each condition by age ranges at first SMI diagnosis for the whole SMI cohort and SSD and BD subgroups.

Supplemental Tables 4a, 4b and 4c. Prevalence for each condition across ethnicities for the whole SMI cohort and SSD and BD subgroups.

Supplemental Table 5. Social deprivation prevalence for each condition for the whole cohort and within SSD and BD.

Supplemental Table 6. Sociodemographic features of cohort in HoNOS subsample analysis

Supplemental Table 7. Associations between comorbidities and HoNOS scores in SSD.

Supplemental Table 8. Associations between comorbidities and HoNOS scores in BD.

Supplemental Figures

Supplemental Figure 1. Distribution of all conditions in the SMI cohort by SMI group.

Supplemental Figure 2. Prevalence rates for age at SMI diagnoses per condition and comparison between individuals with BD and SSD.

Appendix 1. SNOMED Container and Concept Level Groupings for physical health conditions included in this study.

For peer review only

Supplemental Tables

Supplemental Table 1. Meta-annotations performance results for Diagnosis and Status

Meta-Annotation	Values	F1 (macro/weighted)	P (macro/weighted)	R (macro/weighted)
Diagnosis	Patient / Other	0.94 / 0.94	0.95 / 0.95	0.92 / 0.94
Status	Affirmed / Other	0.89 / 0.98	0.94 / 0.98	0.85 / 0.98

For peer review only

Supplemental Table 2. Prevalence estimates and sex differences within the whole SMI cohort and SSD and BD subgroups.

	Total Cohort		SSD			BD		
	Female	Male	Female	Male	p-value	Female	Male	p- value
**Diabetes**	1282 (15.8)	1403 (15)	1021 (18.8)	1187 (15.6)	<.001	261 (9.7)	216 (12.1)	.190
**Hypertension *****	1325 (16.3)	1212 (12.9)	1060 (19.6)	1010 (13.3)	<.001	265 (9.8)	202 (11.4)	1.000
**Asthma ****	869 (10.7)	852 (9.1)	559 (10.3)	732 (9.6)	1.000	310 (11.5)	120 (6.7)	<.001
**Arthritis *****	657 (8.1)	297 (3.2)	471 (8.7)	231 (3.0)	<.001	186 (6.9)	66 (3.7)	<.001
**Epilepsy**	349 (4.3)	450 (4.8)	253 (4.7)	399 (5.3)	1.000	96 (3.6)	51 (2.9)	1.000
**Cerebrovascular diseases**	367 (4.5)	361 (3.9)	265 (4.9)	308 (4.1)	.499	102 (3.8)	53 (3.0)	1.000
**Eczema ***	331 (4.1)	285 (3.0)	238 (4.4)	241 (3.2)	<.01	93 (3.4)	44 (2.5)	1.000
**Migraine *****	369 (4.5)	194 (2.1)	217 (4.0)	155 (2.0)	<.001	152 (5.6)	39 (2.2)	<.001
**Ischaemic heart disease**	249 (3.1)	312 (3.3)	196 (3.6)	239 (3.1)	1.000	53 (2.0)	73 (4.2)	<.001
**Chronic Obstructive Lung diseases**	237 (2.9)	239 (2.5)	153 (2.8)	189 (2.5)	1.000	84 (3.1)	50 (2.8)	1.000
**Chronic Kidney disease**	158 (1.9)	121 (1.3)	104 (1.9)	75 (1.0)	<.001	54 (2.0)	46 (2.6)	1.000
**Parkinson disease**	113 (1.4)	153 (1.6)	81 (1.5)	120 (1.6)	1.000	32 (1.2)	33 (1.9)	1.000
**Heart failure**	123 (1.5)	99 (1.0)	105 (1.9)	82 (1.1)	.001	18 (0.7)	17 (1.0)	1.000
**Psoriasis**	76 (0.9)	103 (1.1)	45 (0.8)	84 (1.1)	1.000	31 (1.1)	19 (1.1)	1.000
**Atrial fibrillation**	65 (0.8)	68 (0.7)	50 (0.9)	50 (0.7)	1.000	15 (0.6)	18 (1.0)	1.000
**Transient Ischaemic Attack**	79 (1.0)	51 (0.5)	53 (1.0)	38 (0.5)	.037	26 (1.0)	13 (0.7)	1.000
**Inflammatory Bowel diseases**	26 (0.3)	14 (0.1)	18 (0.3)	7 (0.1)	.080	8 (0.3)	7 (0.4)	1.000

For peer review only

**Multiple Sclerosis**	17 (0.2)	15 (0.2)	9 (0.2)	10 (0.1)	-	8 (0.3)	5 (0.3)	-
**Chronic liver disease**	10 (0.1)	12 (0.1)	9	11 (0.1)	-	1 (0.0)	1 (0.1)	-
**Chronic Sinusitis**	3 (0.0)	3 (0.0)	3	3 (0.0)	-	0 (0.0)	0 (0.0)	-

Note.· *** $p < .001$; ** $p < .01$; * $p < .05$ for comparisons between male and female groups in the whole SMI cohort

For peer review only

Supplemental Table 3. Prevalence for each condition for the whole cohort and diagnoses subgroups and across age ranges at first SMI diagnosis.

For peer review only

	Total					SSD						BD					
	15-34	35-44	45-54	55-64	65+	15-34	35-44	45-54	55-64	65+		15-34	35-44	45-54	55-64	65+	
**Total**	7497	3736	2783	1525	1959	5607	2792	2057	1067	1496		1890	944	726	458	463	
**Diabetes*****	697 (9.3)	559 (15.0)	531 (19.1)	371 (24.3)	528 (27.0)	591 (10.5)	479 (17.2)	439 (21.3)	284 (26.6)	415 (27.7)	$p < 0.001$	106 (5.6)	80 (8.5)	92 (12.7)	87 (19.0)	113 (24.4)	$p < 0.001$
**Hypertension*****	472 (6.3)	426 (11.4)	514 (18.5)	416 (27.)3	709 (36.2)	412 (7.3)	372 (13.3)	411 (20.0)	313 (29.3)	562 (37.6)	$p < 0.001$	60 (3.2)	54 (5.7)	103 (14.2)	103 (22.5)	147 (31.7)	$p < 0.001$
**Asthma*****	820 (10.9)	347 (9.3)	297 (10.7)	126 (8.3)	132 (6.7)	635 (11.3)	248 (8.9)	218 (10.6)	89 (8.3)	101 (6.8)	$p < 0.001$	185 (9.8)	99 (10.5)	79 (10.9)	37 (8.1)	31 (6.7)	$p = 1.000$
**Arthritis*****	125 (1.7)	154 (4.1)	229 (8.2)	167 (11.0)	279 (14.2)	93 (1.7)	120 (4.3)	159 (7.7)	118 (11.1)	212 (14.2)	$p < 0.001$	32 (1.7)	34 (3.6)	70 (9.6)	49 (10.7)	67 (14.5)	$p < 0.001$
**Epilepsy**	339 (4.5)	188 (5.0)	133 (4.8)	67 (4.4)	72 (3.7)	276 (4.9)	154 (5.5)	112 (5.4)	55 (5.2)	55 (3.7)	1.000	63 (3.3)	34 (3.6)	21 (2.9)	12 (2.6)	17 (3.7)	1.000
**CVA*****	166 (2.2)	112 (3.0)	122 (4.4)	114 (7.5)	214 (10.9)	143 (2.6)	87 (3.1)	95 (4.6)	84 (7.9)	164 (11.0)	$p < 0.001$	23 (1.2)	25 (2.6)	27 (3.7)	30 (6.6)	50 (10.8)	$p < 0.001$
**Eczema**	310 (4.1)	115 (3.1)	79 (2.8)	50 (3.3)	62 (3.2)	244 (4.4)	88 (3.2)	58 (2.8)	37 (3.5)	52 (3.5)	0.120	66 (3.5)	27 (2.9)	21 (2.9)	13 (2.8)	10 (2.2)	1.000
**Migraine*****	300 (4.0)	122 (3.3)	87 (3.1)	32 (2.1)	23 (1.2)	207 (3.7)	73 (2.6)	53 (2.6)	24 (2.2)	15 (1.0)	$p < 0.001$	93 (4.9)	49 (5.2)	34 (4.7)	8 (1.7)	8 (1.7)	0.011
**Isc heart disease*****	111 (1.5)	73 (2.0)	105 (3.8)	95 (6.2)	177 (9.0)	96 (1.7)	60 (2.1)	80 (3.9)	65 (6.1)	134 (9.0)	$p < 0.001$	15 (0.8)	13 (1.4)	25 (3.4)	30 (6.6)	43 (9.3)	$p < 0.001$
**COPD*****	39 (0.5)	58 (1.6)	119 (4.3)	115 (7.5)	145 (7.4)	29 (0.5)	40 (1.4)	87 (4.2)	82 (7.7)	104 (7.0)	$p < 0.001$	10 (0.5)	18 (1.9)	32 (4.4)	33 (7.2)	41 (8.9)	$p < 0.001$
**CKD*****	12 (0.2)	18 (0.5)	41 (1.5)	55 (3.6)	153 (7.8)	9 (0.2)	14 (0.5)	28 (1.4)	33 (3.1)	95 (6.4)	$p < 0.001$	3 (0.2)	4 (0.4)	13 (1.8)	22 (4.8)	58 (12.5)	-
**PD*****	70 (0.9)	33 (0.9)	37 (1.3)	41 (2.7)	85 (4.3)	57 (1.0)	22 (0.8)	27 (1.3)	32 (3.0)	63 (4.2)	$p < 0.001$	13 (0.7)	11 (1.2)	10 (1.4)	9 (2.0)	22 (4.8)	$p < 0.001$
**HF*****	18 (0.2)	30 (0.8)	42 (1.5)	40 (2.6)	92 (4.7)	17 (0.3)	30 (1.1)	38 (1.8)	29 (2.7)	73 (4.9)	$p < 0.001$	1 (0.1)	0 (0.0)	4 (0.6)	11 (2.4)	19 (4.1)	-
**Psoriasis**	67 (0.9)	33 (0.9)	37 (1.3)	17 (1.1)	25 (1.3)	54 (1.0)	25 (0.9)	25 (1.2)	9 (0.8)	16 (1.1)	1.000	13 (0.7)	8 (0.8)	12 (1.7)	8 (1.7)	9 (1.9)	0.450
**Atrial fibrillation*****	14 (0.2)	7 (0.2)	12 (0.4)	19 (1.2)	81 (4.1)	11 (0.2)	6 (0.2)	8 (0.4)	12 (0.1)	63 (4.2)	$p < 0.001$	3 (0.20	1 (0.1)	4 (0.6)	7 (1.5)	18 (3.9)	-
**TIA*****	20 (0.3)	18 (0.5)	19 (0.7)	23 (1.5)	50 (2.6)	15 (0.3)	15 (0.5)	13 (0.6)	14 (1.3)	34 (2.3)	$p < 0.001$	5 (0.3)	3 (0.3)	6 (0.8)	9 (2.0)	16 (3.5)	-
**IBD**	9 (0.1)	8 (0.2)	12 (0.4)	6 (0.4)	5 (0.3)	4 (0.1)	6 (0.2)	7 (0.3)	3 (0.3)	5 (0.3)	-	5 (0.3)	2 (0.2)	5 (0.7)	3 (0.7)	0 (0.0)	-
**MS**	6 (0.1)	8 (0.2)	11 (0.4)	5 (0.3)	2 (0.1)	4 (0.1)	4 (0.1)	8 (0.4)	3 (0.3)	0 (0.0)	-	2 (0.1)	4 (0.4)	3 (0.4)	2 (0.4)	2 (0.4)	-

For peer review only

**Chronic liver disease**	5 (0.1)	7 (0.2)	6 (0.2)	3 (0.2)	1 (0.1)	5 (0.1)	7 (0.3)	5 (0.2)	2 (0.2)	1 (0.1)	-	0 (0.0)	0 (0.0)	1 (0.1)	1 (0.2)	0 (0.0)	-
**Chronic sinusitis**	5 (0.1)	0 (0.0)	1 (0.0)	0 (0.0)	0 (0.0)	5 (0.1)	0 (0.0)	1 (0.0)	0 (0.0)	0 (0.0)	-	0 (0.0)	0 (0.0)	0 (0.0)	0 (0.0)	0 (0.0)	-

Note. *** p < .001; ** p < .01; * p < .05 for comparisons between age groups within the SMI cohort.

For peer review only

Supplemental Tables 4. Prevalence for each condition for the whole cohort and diagnoses subgroups and across ethnicities

Table 4a. Prevalence for each condition for the whole cohort and ethnicity.

	White British n=6243 (35.7%)	Irish n=346 (2.0%)	Black Caribbean n=3182 (18.2%)	Black African n=2094 (12.0%)	South Asian n=549 (3.1%)	Other§ n=2846 (22.0%)	Unknown§ n=1240 (7.1%)	Statistics
**Diabetes**	837 (13.4)	55 (15.9)	757 (23.8)	382 (18.2)	125 (22.8)	461 (12.0)	69 (5.6)	χ^2 (4) = 172.49; *p* <0.001
**Hypertension**	804 (12.9)	63 (18.2)	678 (21.3)	430 (20.5)	102 (18.6)	400 (10.4)	60 (4.8)	χ^2 (4) = 137.98; *p* <0.001
**Asthma**	651 (10.4)	40 (11.6)	484 (15.2)	156 (7.4)	42 (7.7)	296 (7.7)	53 (4.3)	χ^2 (4) = 92.58; *p* <0.001
**Arthritis**	402 (6.4)	29 (8.4)	246 (7.7)	90 (4.3)	36 (6.6)	139 (3.6)	12 (1.0)	χ^2 (4) = 26.80; *p* <0.001
**Epilepsy**	339 (5.4)	26 (7.5)	164 (5.2)	77 (3.7)	17 (3.1)	147 (3.8)	29 (2.4)	χ^2 (4) = 19.02; *p* = 0.010
**Cerebrovascular accident**	274 (4.4)	20 (5.8)	184 (5.8)	92 (4.4)	25 (4.6)	116 (3.0)	17 (1.4)	χ^2 (4) = 10.60; *p* = 0.408
**Eczema**	222 (3.6)	7 (2.0)	185 (5.8)	61 (2.9)	17 (3.1)	108 (2.8)	16 (1.3)	χ^2 (4) = 41.93; *p* <0.001
**Migraine**	235 (3.8)	12 (3.5)	129 (4.1)	61 (2.9)	17 (3.1)	98 (2.5)	12 (1.0)	χ^2 (4) = 5.44; *p* >.99
**Ischemic heart disease**	261 (4.2)	13 (3.8)	108 (3.4)	48 (2.3)	19 (3.5)	99 (2.6)	13 (1.0)	χ^2 (4) = 16.74; *p* = 0.028
**COPD**	271 (4.3)	32 (9.2)	63 (2.0)	21 (1.0)	14 (2.6)	60 (1.6)	15 (1.2)	χ^2 (4) = 114.69; *p* <0.001
**CKD**	125 (2.0)	9 (2.6)	56 (1.8)	32 (1.5)	13 (2.4)	37 (1.0)	7 (0.6)	χ^2 (4) = 3.81; *p* >.99
**Parkinson's disease**	117 (1.9)	6 (1.7)	56 (1.8)	25 (1.2)	11 (2.0)	45 (1.2)	6 (0.5)	χ^2 (4) = 4.56; *p* >.99
**Heart failure**	88 (1.4)	8 (2.3)	55 (1.7)	29 (1.4)	8 (1.5)	31 (0.8)	3 (0.2)	χ^2 (4) = 3.16; *p* >.99
**Psoriasis**	103 (1.6)	4 (1.2)	12 (0.4)	6 (0.3)	5 (0.9)	44 (1.1)	5 (0.4)	*
**Atrial fibrillation**	69 (1.1)	9 (2.6)	23 (0.7)	11 (0.5)	2 (0.4)	15 (0.4)	4 (0.3)	*

For peer review only

**TIA**	58 (0.9)	7 (2.0)	24 (0.8)	15 (0.7)	7 (1.3)	13 (0.3)	6 (0.5)	*
**IBD**	24 (0.4)	1 (0.3)	2 (0.1)	2 (0.1)	1 (0.2)	9 (0.2)	1 (0.1)	*
**Multiple sclerosis**	21 (0.3)	1 (0.3)	3 (0.1)	1 (0.0)	0 (0.0)	4 (0.1)	2 (0.2)	*
**Chronic liver disease**	9 (0.1)	1 (0.3)	1 (0.0)	6 (0.3)	0 (0.0)	5 (0.1)	0 (0.0)	*
**Chronic sinusitis**	1 (0.0)	0 (0.0)	3 (0.1)	1 (0.0)	0 (0.0)	1 (0.0)	0 (0.0)	*

§Categories were dropped for statistical analysis. *χ2 test not performed due to small population sizes

For peer review only

Table 4b. Prevalence for each condition across ethnicities in SSD.

	**White British n=4008 (30.8%)**	**Irish n=240 (1.8%)**	**Black Caribbean n=2799 (21.5%)**	**Black African n=1886 (14.5%)**	**South Asian n=421 (3.2%)**	**Other§ n=2822 (21.7%)**	**Unknown§ n=843 (6.5%)**	**Statistics**
**Diabetes**	602 (15.0)	37 (15.4)	682 (24.4)	351 (18.6)	87 (20.7)	390 (13.8)	59 (7.0)	χ^2 (4) = 97.10; *p* <0.001
**Hypertension**	573 (14.3)	47 (19.6)	606 (21.7)	396 (21.0)	77 (18.3)	322 (11.4)	49 (5.8)	χ^2 (4) = 73.74; *p* <0.001
**Asthma**	443 (11.1)	23 (9.6)	408 (14.6)	134 (7.1)	26 (6.2)	217 (7.7)	40 (4.7)	χ^2 (4) = 75.96; *p* <0.001
**Arthritis**	252 (6.3)	17 (7.1)	206 (7.4)	86 (4.6)	29 (6.9)	103 (3.6)	9 (1.1)	χ^2 (4) = 15.48; *p* = 0.038
**Epilepsy**	261 (6.5)	23 (9.6)	141 (5.0)	70 (3.7)	14 (3.3)	117 (4.1)	26 (3.1)	χ^2 (4) = 32.45; *p* <0.001
**Cerebrovascular accident**	188 (4.7)	15 (6.2)	159 (5.7)	82 (4.3)	18 (4.3)	96 (3.4)	15 (1.8)	χ^2 (4) = 6.48; *p* >0.99
**Eczema**	145 (3.6)	4 (1.7)	159 (5.7)	54 (2.9)	12 (2.9)	93 (3.3)	12 (1.4)	χ^2 (4) = 33.32; *p* <0.001
**Migraine**	126 (3.1)	10 (4.2)	110 (3.9)	48 (2.5)	12 (2.9)	59 (2.1)	7 (0.8)	χ^2 (4) = 8.03; *p* = 0.904
**Ischemic heart disease**	187 (4.7)	9 (3.8)	98 (3.5)	46 (2.4)	13 (3.1)	74 (2.6)	8 (0.9)	χ^2 (4) = 19.15; *p* = 0.007
**COPD**	190 (4.7)	18 (7.5)	51 (1.8)	16 (0.8)	9 (2.1)	44 (1.6)	14 (1.7)	χ^2 (4) = 101.62; *p* <0.001
**CKD**	64 (1.6)	5 (2.1)	45 (1.6)	26 (1.4)	10 (2.4)	24 (0.9)	5 (0.6)	*
**Parkinson's disease**	79 (2.0)	4 (1.7)	49 (1.8)	21 (1.1)	6 (1.4)	36 (1.3)	6 (0.7)	*

**Heart failure**	65 (1.6)	8 (3.3)	50 (1.8)	28 (1.5)	7 (1.7)	26 (0.9)	3 (0.4)	*
**Psoriasis**	66 (1.6)	2 (0.8)	9 (0.3)	6 (0.3)	4 (1.0)	37 (1.3)	5 (0.6)	*
**Atrial fibrillation**	46 (1.1)	7 (2.9)	22 (0.8)	11 (0.6)	1 (0.2)	10 (0.4)	3 (0.4)	*
**TIA**	34 (0.8)	5 (2.1)	21 (0.8)	13 (0.7)	6 (1.4)	8 (0.3)	4 (0.5)	*
**IBD**	12 (0.3)	1 (0.4)	2 (0.1)	2 (0.1)	1 (0.2)	6 (0.2)	1 (0.1)	*
**Multiple sclerosis**	12 (0.3)	1 (0.4)	3 (0.1)	0 (0.0)	0 (0.0)	2 (0.1)	1 (0.1)	*
**Chronic liver disease**	7 (0.2)	1 (0.4)	1 (0.0)	6 (0.3)	0 (0.0)	5 (0.2)	0 (0.0)	*
**Chronic sinusitis**	1 (0.0)	0 (0.0)	3 (0.1)	1 (0.1)	0 (0.0)	1 (0.0)	0 (0.0)	*

§Categories were dropped for statistical analysis. *$\chi 2$ test not performed due to small population sizes

Table 4c. Prevalence for each condition across ethnicities in BD.

	White British n=2235 (49.9%)	Irish n=106 (2.4%)	Black Caribbean n=383 (8.5%)	Black African n=208 (4.6%)	South Asian n=128 (2.9%)	Other§ n=1024 (22.9%)	Unknown§ n=397 (8.9%)	Statistics
**Diabetes**	235 (10.5)	18 (17.0)	75 (19.6)	31 (14.9)	38 (29.7)	71 (6.9)	10 (2.5)	χ^2 (4) = 60.65; p <0.001
**Hypertension**	231 (10.3)	16 (15.1)	72 (18.8)	34 (16.3)	25 (19.5)	78 (7.6)	11 (2.8)	χ^2 (4) = 32.99; p <0.001
**Asthma**	208 (9.3)	17 (16.0)	76 (19.8)	22 (10.6)	16 (12.5)	79 (7.7)	13 (3.3)	χ^2 (4) = 39.95; p <0.001
**Arthritis**	150 (6.7)	12 (11.3)	40 (10.4)	4 (1.9)	7 (5.5)	36 (3.5)	3 (0.8)	χ^2 (4) = 19.09; p = 0.004
**Epilepsy**	78 (3.5)	3 (2.8)	23 (6.0)	7 (3.4)	3 (2.3)	30 (2.9)	3 (0.8)	*
**Cerebrovascular accident**	86 (3.8)	5 (4.7)	25 (6.5)	10 (4.8)	7 (5.5)	20 (2.0)	2 (0.5)	*
**Eczema**	77 (3.4)	3 (2.8)	26 (6.8)	7 (3.4)	5 (3.9)	15 (1.5)	4 (1.0)	*
**Migraine**	109 (4.9)	2 (1.9)	19 (5.0)	13 (6.2)	5 (3.9)	39 (3.8)	5 (1.3)	χ^2 (4) = 3.17; p >0.99
**Ischemic heart disease**	74 (3.3)	4 (3.8)	10 (2.6)	2 (1.0)	6 (4.7)	25 (2.4)	5 (1.3)	*

For peer review only

**COPD**	81 (3.6)	14 (13.2)	12 (3.1)	5 (2.4)	5 (3.9)	16 (1.6)	1 (0.3)	*
**CKD**	61 (2.7)	4 (3.8)	11 (2.9)	6 (2.9)	3 (2.3)	13 (1.3)	2 (0.5)	*
**Parkinson's disease**	38 (1.7)	2 (1.9)	7 (1.8)	4 (1.9)	5 (3.9)	9 (0.9)	0 (0.0)	*
**Heart failure**	23 (1.0)	0 (0.0)	5 (1.3)	1 (0.5)	1 (0.8)	5 (0.5)	0 (0.0)	*
**Psoriasis**	37 (1.7)	2 (1.9)	3 (0.8)	0 (0.0)	1 (0.8)	7 (0.7)	0 (0.0)	*
**Atrial fibrillation**	23 (1.0)	2 (1.9)	1 (0.3)	0 (0.0)	1 (0.8)	5 (0.5)	1 (0.3)	*
**TIA**	24 (1.1)	2 (1.9)	3 (0.8)	2 (1.0)	1 (0.8)	5 (0.5)	2 (0.5)	*
**IBD**	12 (0.5)	0 (0.0)	0 (0.0)	0 (0.0)	0 (0.0)	3 (0.3)	0 (0.0)	*
**Multiple sclerosis**	9 (0.4)	0 (0.0)	0 (0.0)	1 (0.5)	0 (0.0)	2 (0.2)	1 (0.3)	*
**Chronic liver disease**	2 (0.1)	0 (0.0)	0 (0.0)	0 (0.0)	0 (0.0)	0 (0.0)	0 (0.0)	*
**Chronic sinusitis**	0 (0.0)	0 (0.0)	0 (0.0)	0 (0.0)	0 (0.0)	0 (0.0)	0 (0.0)	*

[§]Categories were dropped for statistical analysis. *$\chi 2$ test not performed due to small population sizes

For peer review only

Supplemental Table 5. Social deprivation prevalence for each condition for the whole cohort and within SSD and BD.

	Total cohort						SSD							AD						
	**1 - least deprived n (%)**	**2 n (%)**	**3 n (%)**	**4 n (%)**	**5 - most deprived n (%)**	**Not stated§ n (%)**	**1 - least deprived n (%)**	**2 n (%)**	**3 n (%)**	**4 n (%)**	**5 - most deprived n (%)**	**Not stated§ n (%)**	**Chi square**	**1 - least deprived n (%)**	**2 n (%)**	**3 n (%)**	**4 n (%)**	**5 - most deprived n (%)**	**Not stated§ n (%)**	**Chi square**
**Total**	742 (100)	1384 (100)	3575 (100)	7073 (100)	4033 (100)	693 (100)	412 (100)	887 (100)	2503 (100)	5476 (100)	3204 (100)	537 (100)		330 (100)	497 (100)	1072 (100)	1597 (100)	829 (100)	156 (100)	
**Diabetes *****	62 (8.4)	169 (12.2)	528 (14.8)	1201 (17.0)	698 (17.3)	28 (4.0)	49 (11.9)	117 (13.2)	409 (16.3)	1019 (18.6)	594 (18.5)	20 (3.7)	$X^2(4)$ = 29.7; p<0.001	13 (3.9)	52 (10.5)	119 (11.1)	182 (11.4)	104 (12.5)	8 (5.1)	$X^2(4)$ = 19.4; p=0.007
**Hypertension *****	77 (10.4)	154 (11.1)	468 (13.1)	1189 (16.8)	627 (15.5)	22 (3.2)	51 (12.4)	108 (12.2)	368 (14.7)	1008 (18.4)	517 (16.1)	18 (3.4)	$X^2(4)$ = 37.8; p<0.001	26 (7.9)	46 (9.3)	100 (9.3)	181 (11.3)	110 (13.3)	4 (2.6)	$X^2(4)$ = 12.3; p=0.17
**Asthma *****	42 (5.7)	95 (6.9)	361 (10.1)	758 (10.7)	432 (10.7)	34 (4.9)	27 (6.6)	61 (6.9)	251 (10.0)	589 (10.8)	337 (10.5)	26 (4.8)	$X^2(4)$ = 19.0; p=0.01	15 (4.5)	34 (6.8)	110 (10.3)	169 (10.6)	95 (11.5)	8 (5.1)	$X^2(4)$ = 19.2; p=0.008
**Arthritis**	36 (4.9)	56 (4.0)	188 (5.3)	411 (5.8)	256 (6.3)	7 (1.0)	18 (4.4)	36 (4.1)	135 (5.4)	312 (5.7)	197 (6.1)	4 (0.7)	$X^2(4)$ = 7.30; p>0.99	18 (5.5)	20 (4.0)	53 (4.9)	99 (6.2)	59 (7.1)	3 (1.9)	$X^2(4)$ = 7.51; p>0.99
**Epilepsy**	32 (4.3)	50 (3.6)	160 (4.5)	344 (4.9)	202 (5.0)	11 (1.2)	24 (5.8)	40 (4.5)	123 (4.9)	292 (5.3)	164 (5.1)	9 (1.7)	$X^2(4)$ = 1.79; p>0.99	8 (2.4)	10 (2.0)	37 (3.5)	52 (3.3)	38 (4.6)	2 (1.3)	$X^2(4)$ = 7.59; p>0.99
**CVA**	19 (2.6)	52 (3.8)	146 (4.1)	328 (4.6)	180 (4.5)	3 (0.4)	12 (2.9)	33 (3.7)	107 (4.3)	272 (5.0)	146 (4.6)	3 (0.6)	$X^2(4)$ = 6.55; p>0.99	7 (2.1)	19 (3.8)	39 (3.6)	56 (3.5)	34 (4.1)	0	$X^2(4)$ = 2.80; p>0.99
**Eczema**	22 (3.0)	45 (3.3)	120 (3.4)	273 (3.9)	147 (3.6)	9 (1.3)	17 (4.1)	34 (3.8)	86 (3.4)	212 (3.9)	123 (3.8)	7 (1.3)	$X^2(4)$ = 1.11; p>0.99	5 (1.5)	11 (2.2)	34 (3.2)	61 (3.8)	24 (2.9)	2 (1.3)	$X^2(4)$ = 6.90; p>0.99
**Migraine**	17 (2.3)	39 (2.8)	121 (3.4)	243 (3.4)	140 (3.5)	4 (0.6)	11 (2.7)	20 (2.3)	63 (2.5)	176 (3.2)	99 (3.1)	3 (0.6)	$X^2(4)$ = 4.79; p>0.99	6 (1.8)	19 (3.8)	58 (5.4)	67 (4.2)	41 (4.9)	1 (0.6)	$X^2(4)$ = 8.94; p=0.69
**Ischaemic heart disease**	20 (2.7)	48 (3.5)	103 (2.9)	240 (3.4)	143 (3.5)	7 (1.0)	9 (2.2)	33 (3.7)	74 (3.0)	194 (3.5)	120 (3.7)	5 (0.9)	$X^2(4)$ = 4.99; p>0.99	11 (3.3)	15 (3.0)	29 (2.7)	46 (2.9)	23 (2.8)	2 (1.3)	$X^2(4)$ = 0.43; p>0.99
**COPD ****	6 (0.8)	35 (2.5)	83 (2.3)	208 (2.9)	139 (3.4)	5 (0.7)	3 (0.7)	22 (2.5)	59 (2.4)	159 (2.9)	95 (3.0)	4 (0.7)	$X^2(4)$ = 9.07; p=0.77	3 (0.9)	13 (2.6)	24 (2.2)	49 (3.1)	44 (5.3)	1 (0.6)	$X^2(4)$ = 21.9; p=0.002

For peer review only

**CKD**	8 (1.1)	22 (1.6)	46 (1.3)	128 (1.8)	74 (1.8)	1 (0.1)	4 (1.0)	9 (1.0)	28 (1.1)	81 (1.5)	56 (1.7)	1 (0.2)	$X^2(4)$ = 5.83; p>0.99	4 (1.2)	13 (2.6)	18 (1.7)	47 (2.9)	18 (2.2)	0	$X^2(4)$ = 6.76; p>0.99
**PD**	9 (1.2)	22 (1.6)	51 (1.4)	121 (1.7)	60 (1.5)	3 (0.4)	5 (1.2)	16 (1.8)	33 (1.3)	97 (1.8)	48 (1.5)	2 (0.4)	$X^2(4)$ = 3.13; p>0.99	4 (1.2)	6 (1.2)	18 (1.7)	24 (1.5)	12 (1.4)	1 (0.6)	---
**Heart failure**	10 (1.3)	12 (0.9)	47 (1.3)	92 (1.3)	58 (1.4)	3 (0.4)	7 (1.7)	10 (1.1)	39 (1.6)	78 (1.4)	52 (1.6)	1 (0.2)	$X^2(4)$ = 1.54; p=0.82	3 (0.9)	2 (0.4)	8 (0.7)	14 (0.9)	6 (0.7)	2 (1.3)	---
**Psoriasis**	11 (1.5)	12 (0.9)	33 (0.9)	71 (1.0)	48 (1.2)	4 (0.6)	4 (1.0)	6 (0.7)	24 (1.0)	54 (1.0)	37 (1.2)	4 (0.7)	---	7 (2.1)	6 (1.2)	9 (0.8)	17 (1.1)	11 (1.3)	0	---
**Atrial fibrillation**	10 (1.3)	11 (0.8)	27 (0.8)	53 (0.7)	29 (0.7)	3 (0.4)	6 (1.5)	7 (0.8)	21 (0.8)	42 (0.8)	23 (0.7)	1 (0.2)	---	4 (1.2)	4 (0.8)	6 (0.6)	11 (0.7)	6 (0.7)	2 (1.3)	---
**TIA**	3 (0.3)	17 (1.0)	27 (0.6)	55 (0.6)	28 (0.5)	0	1 (0.2)	9 (1.0)	16 (0.6)	43 (0.8)	22 (0.7)	0	---	2 (0.6)	8 (1.6)	11 (1.0)	12 (0.8)	6 (0.7)	0	---
**IBD**	1 (0.1)	4 (0.3)	10 (0.3)	12 (0.2)	13 (0.3)	0	0	1 (0.1)	7 (0.3)	7 (0.1)	10 (0.3)	0	---	1 (0.3)	3 (0.6)	3 (0.3)	5 (0.3)	3 (0.4)	0	---
**MS**	4 (0.5)	2 (0.1)	7 (0.2)	14 (0.2)	4 (0.1)	1 (0.1)	2 (0.5)	2 (0.2)	2 (0.1)	9 (0.2)	3 (0.1)	1 (0.2)	---	2 (0.6)	0	5 (0.5)	5 (0.3)	1 (0.1)	0	---
**Chronic liver disease**	0	1 (0.1)	8 (0.2)	8 (0.1)	5 (0.1)	0	0	0	8 (0.3)	8 (0.1)	4 (0.1)	0	---	0	1 (0.2)	0	0	1 (0.1)	0	---
**Chronic sinusitis**	0	0	1 (0.0)	2 (0.0)	3 (0.1)	0	0	0	1 (0.0)	2 (0.0)	3 (0.1)	0	---	0	0	0	0	0	0	---

*, p<0.05; **, p≤0.01; ***, p≤0.001; CVA, cerebrovascular accident; COPD, chronic obstructive lung disease; CKD, chronic kidney disease; PD, Parkinson's disease; TIA, transient ischemic attack; IBD, inflammatory bowel disease; MS, multiple sclerosis

For peer review only

For peer review only

Supplemental Table 6. Sociodemographic features of cohort in HoNOS subsample analysis

	Total n (%)	SSD n (%)	BD n (%)
**Totals n (%)**	13650 (100.0)	10384 (76.1)	3266 (23.9)
**Sex*** **			
Female	6537 (47.9)	4526 (69.2)	2011 (30.8)
Male	7112 (52.1)	5858 (82.4)	1254 (17.6)
**Age at first SMI diagnosis*** **			
15 – 34	5821 (42.6)	4420 (42.6)	1401 (42.9(
35 – 44	3430 (25.1)	2183 (75.3)	716 (24.7)
45 – 54	2082 (15.3)	1594 (76.6)	488 (23.4)
55 – 64	1089 (8.0)	811 (74.5)	278 (25.5)
65+	1759 (12.9)	1376 (13.3)	383 (11.7)
**Ethnicity*** **			
White British	4571 (33.5)	2961 (64.8)	1610 (35.2)
Black Caribbean	2849 (20.9)	2515 (88.3)	334 (11.7)
Black African	1852 (13.6)	1679 (90.7)	173 (9.3)
South Asian	436 (3.2)	338 (77.5)	98 (22.5)
Irish	286 (2.1)	197 (68.9)	89 (31.1)
Other§	3127 (22.9)	2327 (74.4)	800 (25.6)
Not stated§	529 (3.9)	367 (3.5)	162 (5.0)
**Index of multiple deprivation*** **			
1 (least deprivation)	380 (2.8)	237 (62.4)	143 (37.6)
2	883 (6.5)	581 (65.8)	302 (34.2)
3	2786 (20.4)	1994 (71.6)	792 (28.4)
4	5947 (43.6)	4654 (78.3)	1293 (21.7)
5 (most deprivation)	3308 (24.2)	2650 (80.1)	658 (19.9)
Unknown§	346 (2.5)	268 (77.5)	78 (22.5)
**Physical conditions*** **			
No mentions	7232 (53.0)	5320 (73.6)	1912 (26.4)
One	3295 (24.1)	2596 (78.8)	699 (21.2)
Two	1669 (12.2)	1340 (80.3)	329 (19.7)
Three or more	1454 (10.7)	1128 (77.6)	326 (22.4)

Note. *** $p < .001$ *for comparisons between BD and SSD groups.* §Not included in analyses.

For peer review only

Supplemental Table 7. Associations between comorbidities and HoNOS scores in SSD.

Comorbidity	HoNOS Score	Unadjusted	M1	M2a
	**Mean (SD)**	**B (95% CI)**	**B (95% CI)**	**B (95% CI)**
**Whole cohort**	10.75 (6.10)			
**Diabetes**	11.04 (6.10)	0.360 (0.067 – 0.654)**	0.224 (-0.073 – 0.522)	0.300 (-0.002 – 0.602)
Ref: No diabetes	10.68 (6.10)			
**Hypertension**	11.17 (6.05)	0.519 (0.220 – 0.818)***	0.264 (-0.048 – 0.575)	0.324 (0.006 – 0.641)*
Ref: No hypertension	10.65 (6.11)			
**Asthma**	11.19 (6.11)	0.492 (0.126 – 0.859)**	0.551 (0.185 – 0.918)**	0.530 (0.158 – 0.903)**
Ref: No asthma	10.70 (6.10)			
**Arthritis**	12.08 (6.21)	1.422 (0.948– 1.895)***	1.259 (0.775 – 1.742)***	1.274 (0.788 – 1.759)***
Ref: No arthritis	10.66 (6.08)			
**Epilepsy**	11.53 (6.24)	0.827 (0.316 – 1.337)***	0.850 (0.341 – 1.358)***	0.856 (0.341 – 1.370)***
Ref: No epilepsy	10.71 (6.09)			
**CVA**	11.87 (6.23)	1.178 (0.651 – 1.705)***	0.913 (0.383 – 1.443)***	0.921 (0.388 – 1.454)**
Ref: No CVA	10.69 (6.09)			
**Eczema**	11.48 (6.25)	0.763 (0.188 – 1.338)**	0.846 (0.273 – 1.420)**	0.754 (0.172 – 1.336)*
Ref: No eczema	10.72 (6.09)			
**Migraine**	10.71 (5.87)	-0.041 (-0.683 – 0.602)	0.196 (-0.447 – 0.839)	0.192 (-0.458 – 0.842)
Ref: No migraine	10.75 (6.11)			
**Ischaemic heart disease**	11.59 (5.91)	0.874 (0.274 – 1.474)**	0.520 (-0.083 – 1.124)	0.477 (-0.132 – 1.085)
Ref: No ischaemic heart disease	10.72 (6.11)			
**COPD**	12.15 (5.70)	1.444 (0.760– 2.128)***	1.045 (0.354 – 1.736)**	1.043 (0.343 – 1.742)**
Ref: No COPD	10.71 (6.11)			
**Number of comorbidities**		0.387 (0.294 – 0.481)***	0.328 (0.231 – 0.425)***	0.342 (0.243 – 0.441) ***
**1 or more comorbidities**	11.13 (6.11)	0.745 (0.511 –0.980)***	0.607 (0.367 – 0.847)***	0.657 (0.410 – 0.903)***
Ref: No comorbidities	10.39 (6.07)			
**2 or more comorbidities**	11.45 (6.16)	0.921 (0.646 – 1.196)***	0.747 (0.464 – 1.030)***	0.771 (0.484 – 1.059)***
Ref: Less than 1 comorbidities	10.53 (6.07)			

Supplemental Table8. Associations between comorbidities and HoNOS scores in BD.

Comorbidity	HoNOS Score	Unadjusted	M1	M2a
	Mean (SD)	B (95% CI)	B (95% CI)	B (95% CI)
**Whole cohort**	9.28 (5.77)			
**Diabetes**	10.941 (5.97)	1.304 (0.720 – 1.887)***	0.968 (0.370 – 1.565)**	0.961 (0.355 – 1.568)**
Ref: No diabetes	9.11 (5.72)			
**Hypertension**	10.13 (5.82)	0.972 (0.384 – 1.561)**	0.446 (-0.182 – 1.074)	0.277 (-0.359 – 0.912)
Ref: No hypertension	9.15 (5.76)			
**Asthma**	10.61 (6.27)	1.512 (0.900 – 2.124)***	1.597 (0.985 – 2.210)***	1.503 (0.887 – 2.120)***
Ref: No asthma	9.10 (5.68)			
**Arthritis**	11.81 (6.13)	2.725 (1.960 – 3.490)***	2.487 (1.702 – 3.273)***	2.396 (1.606 – 3.186)***
Ref: No arthritis	9.09 (5.70)			
**Epilepsy**	10.77 (5.96)	1.552 (0.513 – 2.591)**	1.587 (0.551 – 2.624)**	1.496 (0.466 – 2.527)**
Ref: No epilepsy	9.22 (5.76)			
**CVA**	11.60 (6.16)	2.425 (1.464 – 3.386)***	2.098 (1.124 – 3.071)***	2.030 (1.064 – 2.996)***
Ref: No CVA	9.17 (5.73)			
**Eczema**	9.83 (5.82)	0.578 (-0.446 – 1.603)	0.622 (-0.400 – 1.644)	0.427 (-0.603 – 1.458)
Ref: No eczema	9.26 (5.77)			
**Migraine**	9.55 (5.34)	0.284 (-0.586– 1.154)	0.523 (-0.350– 1.397)	0.527 (-0.346 – 1.400)
Ref: No migraine	9.26 (5.80)			
**Ischaemic heart disease**	11.84 (5.95)	2.649 (1.560 – 3.738)***	2.093 (0.987 – 3.200)***	2.204 (1.098 – 3.309)**
Ref: No ischaemic heart disease	9.19 (5.75)			
**COPD**	11.89 (5.99)	2.711 (1.679 – 3.743)***	2.298 (1.248 – 3.348)***	2.128 (1.077 – 3.179)***
Ref: No COPD	9.18 (5.74)			
**Number of comorbidities**		0.723 (0.566 – 0.879)***	0.661 (0.494 – 0.828)***	0.642 (0.473 – 0.811) ***
**1 or more comorbidities**	10.30 (5.91)	1.740 (1.343 –2.138)***	1.559 (1.143 – 1.974)***	1.457 (1.031 – 1.883)***
Ref: No comorbidities	8.56 (5.57)			
**2 or more comorbidities**	10.89 (5.99)	2.013 (1.524 – 2.503)***	1.797 (1.280 – 2.314)***	1.789 (1.267 – 2.311)***
Ref: Less than 1 comorbidities	8.88 (5.64)			

Supplemental Figure 1. Distribution of all conditions in the SMI cohort by SMI diagnoses

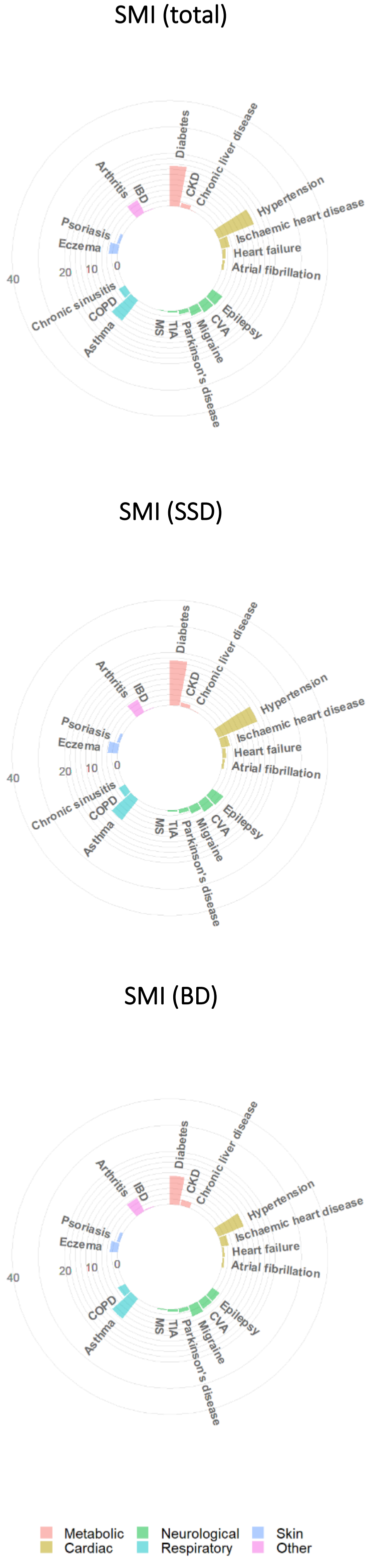

Supplemental Figure 2. Prevalence rates for age at SMI diagnoses per condition and comparison between individuals with BD and SSD.

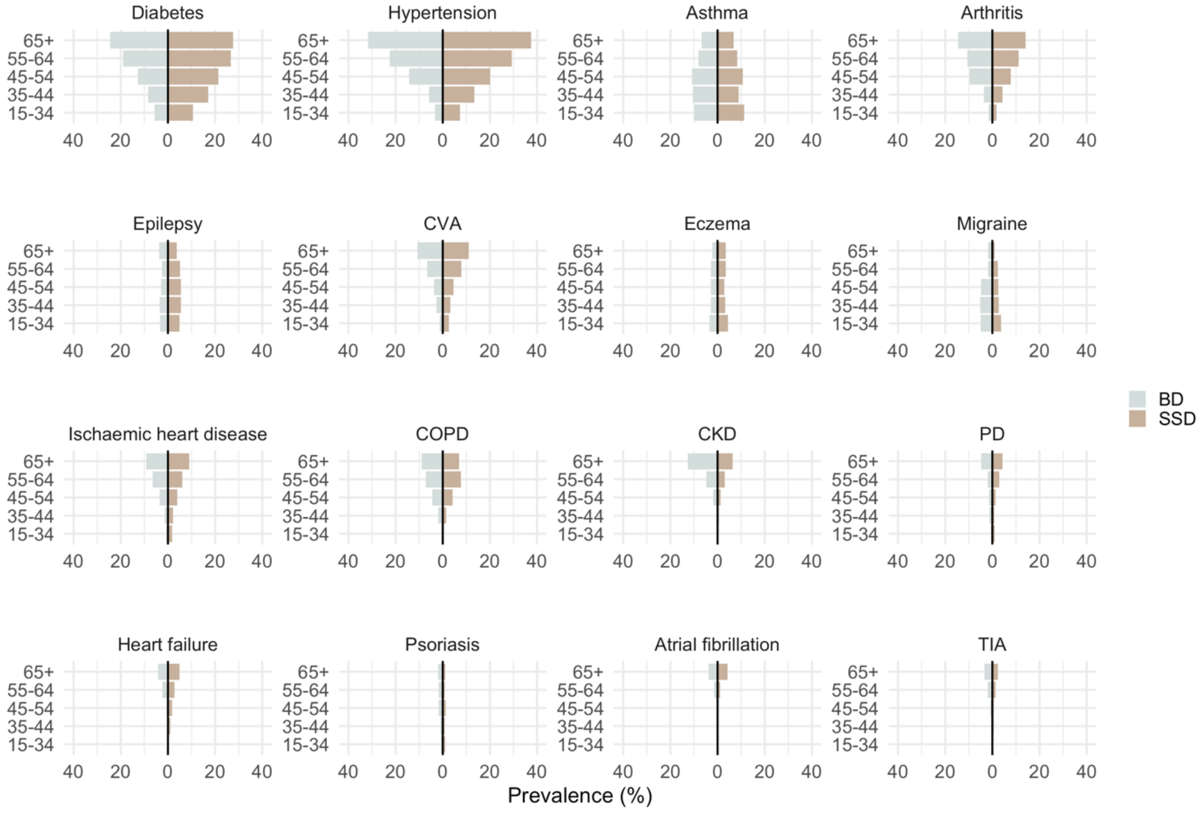

For peer review only - http://bmjopen.bmj.com/site/about/guidelines.xhtml

Appendix 1. SNOMED Container and Concept Level Groupings for physical health conditions included in this study.

Container Concept	Concepts
S-73211009 - Diabetes mellitus (disorder)	S-44054006 - Diabetes mellitus type 2 (disorder)<br>S-46635009 - Diabetes mellitus type 1 (disorder)<br>S-422088007 - Disorder of nervous system co-occurrent and due to diabetes mellitus (disorder)<br>S-25093002 - Disorder of eye co-occurrent and due to diabetes mellitus (disorder)<br>S-73211009 - Diabetes mellitus (disorder)
S-84114007 - Heart failure (disorder)	S-128404006 - Right heart failure (disorder)<br>S-48447003 - Chronic heart failure (disorder)<br>S-56675007 - Acute heart failure (disorder)<br>S-85232009 - Left heart failure (disorder)<br>S-42343007 - Congestive heart failure (disorder)<br>S-84114007 - Heart failure (disorder)
S-414545008 - Ischemic heart disease (disorder)	S-413439005 - Acute ischemic heart disease (disorder)<br>S-413838009 - Chronic ischemic heart disease (disorder)<br>S-194828000 - Angina (disorder)<br>S-22298006 - Myocardial infarction (disorder)<br>S-414545008 - Ischemic heart disease (disorder)
S-38341003 - Hypertensive disorder, systemic arterial (disorder)	S-31992008 - Secondary hypertension (disorder)<br>S-48146000 - Diastolic hypertension (disorder)<br>S-56218007 - Systolic hypertension (disorder)<br>S-59621000 - Essential hypertension (disorder)<br>S-38341003 - Hypertensive disorder, systemic arterial (disorder)
S-13645005 - Chronic obstructive lung disease (disorder)	S-195951007 - Acute exacerbation of chronic obstructive airways disease (disorder)<br>S-87433001 - Pulmonary emphysema (disorder)<br>S-13645005 - Chronic obstructive lung disease (disorder)
S-195967001 - Asthma (disorder)	S-195967001 - Asthma (disorder)
S-709044004 - Chronic kidney disease (disorder)	S-723190009 - Chronic renal insufficiency (disorder)<br>S-709044004 - Chronic kidney disease (disorder)

For peer review only

S-230690007 - Cerebrovascular accident (disorder)	S-25133001 - Completed stroke (disorder)<br>S-371040005 - Thrombotic stroke (disorder)<br>S-371041009 - Embolic stroke (disorder)<br>S-413102000 - Infarction of basal ganglia (disorder)<br>S-422504002 - Ischemic stroke (disorder)<br>S-723082006 - Silent cerebral infarct (disorder)<br>S-1078001000000105 - Haemorrhagic stroke (disorder)<br>S-230690007 - Cerebrovascular accident (disorder)
S-266257000 - Transient ischemic attack (disorder)	S-266257000 - Transient ischemic attack (disorder)
S-49049000 - Parkinson's disease (disorder)	S-49049000 - Parkinson's disease (disorder)
S-24700007 - Multiple sclerosis (disorder)	S-24700007 - Multiple sclerosis (disorder)
S-84757009 - Epilepsy (disorder)	S-352818000 - Tonic-clonic epilepsy (disorder)<br>S-19598007 - Generalized epilepsy (disorder)<br>S-230456007 - Status epilepticus (disorder)<br>S-509341000000107 - Petit-mal epilepsy (disorder)<br>S-84757009 - Epilepsy (disorder)
S-37796009 - Migraine (disorder)	S-37796009 - Migraine (disorder)<br>S-4473006 - Migraine with aura (disorder)<br>S-56097005 - Migraine without aura (disorder)
S-53741008 - Coronary arteriosclerosis (disorder)	S-810681000000101 - Coronary microvascular disease (disorder)<br><br>S-53741008 - Coronary arteriosclerosis (disorder)
S-49436004 - Atrial fibrillation (disorder)	S-49436004 - Atrial fibrillation (disorder)
S-40055000 - Chronic sinusitis (disorder)	S-40055000 - Chronic sinusitis (disorder)
S-24526004 - Inflammatory bowel disease (disorder)	S-24526004 - Inflammatory bowel disease (disorder)<br>S-397173003 - Crohn's disease of intestine (disorder)<br>S-64766004 - Ulcerative colitis (disorder)
S-328383001 - Chronic liver disease (disorder)	S-328383001 - Chronic liver disease (disorder)<br>S-76783007 - Chronic hepatitis (disorder)<br>S-79720007 - Chronic nonalcoholic liver disease (disorder)

For peer review only

	S-713181003 - Chronic alcoholic liver disease (disorder)
S-9014002 - Psoriasis (disorder)	S-9014002 - Psoriasis (disorder)
S-43116000 - Eczema (disorder)	S-43116000 - Eczema (disorder)
S-3723001 - Arthritis (disorder)	S-69896004 - Rheumatoid arthritis (disorder)<br>S-399112009 - Seronegative arthritis (disorder)<br>S-35908007 - Chronic arthritis (disorder)<br>S-11939005 - Acute arthritis (disorder)<br>S-3723001 - Arthritis (disorder)

For peer review only

STROBE Statement—checklist of items that should be included in reports of observational studies

	Item No	Recommendation	Page No
**Title and abstract**	1	(*a*) Indicate the study's design with a commonly used term in the title or the abstract	1
		(*b*) Provide in the abstract an informative and balanced summary of what was done and what was found	2
**Introduction**			
Background/rationale	2	Explain the scientific background and rationale for the investigation being reported	4,5
Objectives	3	State specific objectives, including any prespecified hypotheses	5
**Methods**			
Study design	4	Present key elements of study design early in the paper	5,6
Setting	5	Describe the setting, locations, and relevant dates, including periods of recruitment, exposure, follow-up, and data collection	5,6
Participants	6	(*a*) *Cohort study*—Give the eligibility criteria, and the sources and methods of selection of participants. Describe methods of follow-up<br>*Case-control study*—Give the eligibility criteria, and the sources and methods of case ascertainment and control selection. Give the rationale for the choice of cases and controls<br>*Cross-sectional study*—Give the eligibility criteria, and the sources and methods of selection of participants	5
		(*b*) *Cohort study*—For matched studies, give matching criteria and number of exposed and unexposed<br>*Case-control study*—For matched studies, give matching criteria and the number of controls per case	
Variables	7	Clearly define all outcomes, exposures, predictors, potential confounders, and effect modifiers. Give diagnostic criteria, if applicable	6-8
Data sources/ measurement	8*	For each variable of interest, give sources of data and details of methods of assessment (measurement). Describe comparability of assessment methods if there is more than one group	*6-8*
Bias	9	Describe any efforts to address potential sources of bias	6-9
Study size	10	Explain how the study size was arrived at	5,6
Quantitative variables	11	Explain how quantitative variables were handled in the analyses. If applicable, describe which groupings were chosen and why	6-8
Statistical methods	12	(*a*) Describe all statistical methods, including those used to control for confounding	8,9
		(*b*) Describe any methods used to examine subgroups and interactions	8,9
		(*c*) Explain how missing data were addressed	8,9
		(*d*) *Cohort study*—If applicable, explain how loss to follow-up was addressed<br>*Case-control study*—If applicable, explain how matching of cases and controls was addressed<br>*Cross-sectional study*—If applicable, describe analytical methods taking account of sampling strategy	8,9
		(*e*) Describe any sensitivity analyses	8,9

Continued on next page

For peer review only - http://bmjopen.bmj.com/site/about/guidelines.xhtml

For peer review only

**Results**			
Participants	13*	(a) Report numbers of individuals at each stage of study—eg numbers potentially eligible, examined for eligibility, confirmed eligible, included in the study, completing follow-up, and analysed	10
		(b) Give reasons for non-participation at each stage	
		(c) Consider use of a flow diagram	
Descriptive data	14*	(a) Give characteristics of study participants (eg demographic, clinical, social) and information on exposures and potential confounders	10
		(b) Indicate number of participants with missing data for each variable of interest	
		(c) *Cohort study*—Summarise follow-up time (eg, average and total amount)	
Outcome data	15*	*Cohort study*—Report numbers of outcome events or summary measures over time	
		Case-control study—Report numbers in each exposure category, or summary measures of exposure	
		Cross-sectional study—Report numbers of outcome events or summary measures	*9-13*
Main results	16	(*a*) Give unadjusted estimates and, if applicable, confounder-adjusted estimates and their precision (eg, 95% confidence interval). Make clear which confounders were adjusted for and why they were included	9-13
		(*b*) Report category boundaries when continuous variables were categorized	
		(*c*) If relevant, consider translating estimates of relative risk into absolute risk for a meaningful time period	
Other analyses	17	Report other analyses done—eg analyses of subgroups and interactions, and sensitivity analyses	Supp
**Discussion**			
Key results	18	Summarise key results with reference to study objectives	13,14
Limitations	19	Discuss limitations of the study, taking into account sources of potential bias or imprecision. Discuss both direction and magnitude of any potential bias	16
Interpretation	20	Give a cautious overall interpretation of results considering objectives, limitations, multiplicity of analyses, results from similar studies, and other relevant evidence	13-17
Generalisability	21	Discuss the generalisability (external validity) of the study results	16,17
**Other information**			
Funding	22	Give the source of funding and the role of the funders for the present study and, if applicable, for the original study on which the present article is based	18

*Give information separately for cases and controls in case-control studies and, if applicable, for exposed and unexposed groups in cohort and cross-sectional studies.

Note: An Explanation and Elaboration article discusses each checklist item and gives methodological background and published examples of transparent reporting. The STROBE checklist is best used in conjunction with this article (freely available on the Web sites of PLoS Medicine at http://www.plosmedicine.org/, Annals of Internal Medicine at http://www.annals.org/, and Epidemiology at http://www.epidem.com/). Information on the STROBE Initiative is available at www.strobe-statement.org.

For peer review only - http://bmjopen.bmj.com/site/about/guidelines.xhtml

BMJ Open

Mapping Multimorbidity in Individuals with Schizophrenia and Bipolar Disorders: Evidence from the South London and Maudsley NHS Foundation Trust Biomedical Research Centre (SLAM BRC) Case Register

Journal:	*BMJ Open*
Manuscript ID	bmjopen-2021-054414.R2
Article Type:	Original research
Date Submitted by the Author:	26-Nov-2021

Complete List of Authors:	Bendayan, Rebecca; King's College London, Biostatistics and Health Informatics, Institute of Psychiatry, Psychology and Neurosciences; King's College London, NIHR Biomedical Research Centre at South London and Maudsley NHS Foundation Trust, London, United Kingdom<br>Kraljevic, Zeljko; King's College London, Biostatistics and Health Informatics, Institute of Psychiatry, Psychology and Neuroscience<br>Shaari, Shaweena; King's College London, NIHR Biomedical Research Centre at South London and Maudsley NHS Foundation Trust, London, United Kingdom<br>Das-Munshi, Jayati; King's College London Institute of Psychiatry Psychology and Neuroscience, Psychological Medicine<br>Leipold, Leona; King's College London, NIHR Biomedical Research Centre at South London and Maudsley NHS Foundation Trust, London, United Kingdom<br>Chaturvedi, Jaya; King's College London, Biostatistics and Health Informatics, Institute of Psychiatry, Psychology and Neuroscience<br>Mirza, Luwaiza; King's College London, NIHR Biomedical Research Centre at South London and Maudsley NHS Foundation Trust, London, United Kingdom<br>Aldelemi, Sarah; King's College London, NIHR Biomedical Research Centre at South London and Maudsley NHS Foundation Trust, London, United Kingdom<br>Searle, Thomas; King's College London, Department of Biostatistics and Health Informatics<br>Chance, Natalia; King's College London, NIHR Biomedical Research Centre at South London and Maudsley NHS Foundation Trust and King's College London, London, United Kingdom<br>Mascio, Aurelie; King's College London, Biostatistics and Health Informatics, Institute of Psychiatry, Psychology and Neuroscience<br>Skiada, Naoko; King's College London, Biostatistics and Health Informatics, Institute of Psychiatry, Psychology and Neuroscience<br>Wang, Tao; Department of Biostatistics and Health Informatics, Institute of Psychiatry, Psychology and Neuroscience, King's College London, London, United Kingdom<br>Roberts, Angus; King's College London, Biostatistics and Health Informatics, Institute of Psychiatry, Psychology and Neuroscience; NIHR Biomedical Research Centre at South London and Maudsley NHS Foundation Trust and King's College London, London, United Kingdom

	Stewart, Robert; King's College London, Psychological Medicine; NIHR Biomedical Research Centre at South London and Maudsley NHS Foundation Trust and King’s College London, London, United Kingdom<br>Bean, Daniel; King's College London, Biostatistics and Health Informatics<br>Dobson, Richard; King's College London,
**Primary Subject Heading**:	Mental health
Secondary Subject Heading:	Public health, Epidemiology, Health informatics
Keywords:	Health informatics < BIOTECHNOLOGY & BIOINFORMATICS, EPIDEMIOLOGY, MENTAL HEALTH, PSYCHIATRY, PUBLIC HEALTH

SCHOLARONE™
Manuscripts

I, the Submitting Author has the right to grant and does grant on behalf of all authors of the Work (as defined in the below author licence), an exclusive licence and/or a non-exclusive licence for contributions from authors who are: i) UK Crown employees; ii) where BMJ has agreed a CC-BY licence shall apply, and/or iii) in accordance with the terms applicable for US Federal Government officers or employees acting as part of their official duties; on a worldwide, perpetual, irrevocable, royalty-free basis to BMJ Publishing Group Ltd ("BMJ") its licensees and where the relevant Journal is co-owned by BMJ to the co-owners of the Journal, to publish the Work in this journal and any other BMJ products and to exploit all rights, as set out in our licence.

The Submitting Author accepts and understands that any supply made under these terms is made by BMJ to the Submitting Author unless you are acting as an employee on behalf of your employer or a postgraduate student of an affiliated institution which is paying any applicable article publishing charge ("APC") for Open Access articles. Where the Submitting Author wishes to make the Work available on an Open Access basis (and intends to pay the relevant APC), the terms of reuse of such Open Access shall be governed by a Creative Commons licence – details of these licences and which Creative Commons licence will apply to this Work are set out in our licence referred to above.

Other than as permitted in any relevant BMJ Author's Self Archiving Policies, I confirm this Work has not been accepted for publication elsewhere, is not being considered for publication elsewhere and does not duplicate material already published. I confirm all authors consent to publication of this Work and authorise the granting of this licence.

For peer review only

Mapping Multimorbidity in Individuals with Schizophrenia and Bipolar Disorders: Evidence from the South London and Maudsley NHS Foundation Trust Biomedical Research Centre (SLAM BRC) Case Register

Rebecca Bendayan[1,2], Zeljko Kraljevic[2], Shaweena Shaari[1], Jayati Das-Munshi[3], Leona Leipold[1], Jaya Chaturvedi[2], Luwaiza Mirza[1], Sarah Aldelemi[1], Thomas Searle[2], Natalia Chance[1], Aurelie Mascio[2], Naoko Skiada[2], Tao Wang[2], Angus Roberts[1,2], Robert Stewart[1,3], Daniel Bean[2,4], Richard Dobson[1,2,5]

[1]NIHR Biomedical Research Centre at South London and Maudsley NHS Foundation Trust, London, United Kingdom

[2]Department of Biostatistics and Health Informatics, Institute of Psychiatry, Psychology and Neuroscience, King's College London, London, United Kingdom

[3]Department of Psychological Medicine, Institute of Psychiatry, Psychology and Neuroscience, King's College London, London, United Kingdom

[4]Health Data Research UK London, University College London, London, United Kingdom

[5]Institute of Health Informatics, University College London, London, United Kingdom

Word count: 3920

Correspondence:
Rebecca Bendayan / ORCID: 0000-0003-1461-556X
E-mail: rebecca.bendayan@kcl.ac.uk
NIHR Maudsley Biomedical Research Centre
Department of Biostatistics & Health Informatics
SGDP Centre, IoPPN, Box PO 80
De Crespigny Park, Denmark Hill
London SE5 8AF, UNITED KINGDOM

For peer review only

Abstract

Objectives: The first aim of this study was to design and develop a valid and replicable strategy to extract physical health conditions from clinical notes which are common in mental health services. Then, we examined the prevalence of these conditions in individuals with SMI and compared their individual and combined prevalence in individuals with bipolar (BD) and schizophrenia spectrum disorders (SSD).

Design: Observational study.

Setting: Secondary mental healthcare services from South London

Participants: Our maximal sample comprised 17,500 individuals aged 15 years or older who had received a primary or secondary SMI diagnosis (ICD-10, F20-31) between 2007 and 2018.

Measures: We designed and implemented a data extraction strategy for 21 common physical comorbidities using a natural language processing pipeline, MedCAT. Associations were investigated with sex, age at SMI diagnosis, ethnicity and social deprivation for the whole cohort and the BD and SSD subgroups. Linear regression models were used to examine associations with disability measured by the Health of Nations Outcome Scale (HoNOS).

Results: Physical health data was extracted, achieving precision rates (F1) above 0.90 for all conditions. The ten most prevalent conditions were diabetes, hypertension, asthma, arthritis, epilepsy, cerebrovascular accident, eczema, migraine, ischaemic heart disease and COPD. The most prevalent combination in this population included diabetes, hypertension and asthma, regardless of their SMI diagnoses.

Conclusions: Our data extraction strategy was found to be adequate to extract physical health data from clinical notes, which is essential for future multimorbidity research using text records. We found that around 40% of our cohort had multimorbidity from which 20% had complex

For peer review only

multimorbidity (two or more physical conditions besides SMI). Sex, age, ethnicity and social deprivation were found to be key to understand their heterogeneity and their differential contribution to disability levels in this population. These outputs have direct implications for researchers and clinicians.

Strengths and limitations of this study

- We designed and implemented a data extraction strategy which showed high performance rates and allowed us to unlock data from 21 physical health conditions from around 15m clinical documents with free text.
- We mapped how these health conditions are distributed across sex, age, ethnicity, social disadvantage and severe mental illness diagnoses in a sample of 17500 patients from one of Europe's largest providers of secondary mental healthcare, serving a geographic catchment of approximately 1.32 million residents in London.
- We investigated the association between multimorbidity (2 or more conditions) and disability using the Health of the Nation Outcome Scale (HoNOS) which is commonly collected in secondary mental health services in the UK.
- This study focuses on a cohort of individuals with SMI which limits direct comparisons with other mental health conditions and/or general population.
- Although some of the most frequent physical comorbidities were extracted, some specific health conditions required to compute standard comorbidity scores (e.g., Charlson and Exlihauser comorbidity indexes) were not included in this study.

For peer review only

Introduction

Two thirds of the deaths in individuals with severe mental illness (SMI) are potentially explained by the increased risk of multimorbidity in this population.[1–4] However, multimorbidity research in this population is still scarce[5] compounded by the limited availability of physical health data in SMI samples, increased non-response rates in surveys,[6] and physical health information in secondary mental health care data primarily hidden in free text fields.

Most research to date on physical health in SMI populations has focused on cardiometabolic risk factors, which are considered leading contributors to cardiovascular diseases in individuals with SMI,[7–10] or specific conditions such as immune-mediated inflammatory diseases (e.g., inflammatory bowel diseases, psoriasis),[11–13] multiple sclerosis, epilepsy and migraine.[14,15] This condition-specific vision limits our understanding of multimorbidity in SMI and studies that consider a larger number of conditions are needed. However, there are only a few studies which have considered multiple health conditions.[4,16,17] Woodhead et al.[4] showed an increased risk in multimorbidity in SMI patients, but only found epilepsy to be more prevalent as an individual condition. Kugathasan et al.[17] investigated combinations of diseases in schizophrenia at organ system level and found that 31% had complex multimorbidity with the most prevalent pairs including neurologic-endocrine, neurologic-respiratory and neurologic-viral. Similarly, epilepsy and arthritis was one of the most prevalent combinations in Dorrington et al.[16] Although these studies included multiple health conditions and confirmed the need of investigating further multimorbidity in SMI, they are still not comparable to multimorbidity studies in general populations[18–20] and they did not investigate potential differences between individuals with schizophrenia spectrum disorders (SSD) and bipolar disorders (BD). Understanding different

For peer review only

multimorbidity combinations between those groups could contribute to the ongoing debate around potential underlying biological mechanisms.[21–23] Ultimately, SSD and BD have been established as significant drivers of disability[24] and deficits in physical health have been implicated in the perpetuation of impairments in functional capacity and performance. [25,26] However, research into the relationship between multimorbidity and disability in SMI is limited.

Within this context, our first aim was to design and develop a suitable strategy to extract information on physical health conditions from free text mental health records data which could be easily replicated in future multimorbidity research using similar resources. Our second objective was to examine the prevalence of these conditions and their most prevalent combinations in SMI and any differences across relevant sociodemographic factors and across SMI diagnoses (SSD vs BD). Our third objective was to investigate the association of overall multimorbidity and specific physical health conditions with levels of disability measured using the Health of the Nation Outcome Scales (HoNOS).

Methods

Setting and sample

Patient data were extracted via the Clinical Record Interactive Search (CRIS), a case register platform that contains de-identified mental healthcare electronic health record data from the South London and Maudsley Trust NHS Foundation Trust (SLaM). SLaM is one of Europe's largest providers of secondary mental healthcare, serving a geographic catchment of approximately 1.32 million residents, and providing almost complete coverage of secondary mental healthcare provision to all age groups. Since 2007, fully electronic clinical records have been deployed in SLaM, and data from these are accessible via CRIS system which allows searching and retrieval of anonymized full records for over 500,000 cases currently represented in the system.[27]

For peer review only

Our sample (N=17500) consisted of all individuals aged 15 years or older who had received a primary or secondary SMI diagnosis between 2007 and 2018 (International Classification of Mental and Behavioural Disorders 10th edition [ICD-10][28] codes F20-31). Given that one of our objectives was to compare SSD (F20-29) and BD (F30-31), individuals who over those 10 years of follow-up had diagnoses within both categories were excluded (n=804). Excluded individuals were more likely to be female, under the age of 35 at first SMI diagnosis recorded, Black ethnicity and have higher levels of social deprivation.

Physical health conditions

Definitions and Information extraction. To maximise comparability we sought to extract the following 21 physical health conditions representing chronic conditions commonly collected in multimorbidity studies using primary care data [18–20]: diabetes mellitus, heart failure, ischemic heart diseases, hypertension, coronary arteriosclerosis, chronic obstructive pulmonary disease (COPD), asthma, chronic kidney disease, cerebrovascular accident, transient ischemic attack, Parkinson's disease, multiple sclerosis, epilepsy, migraine, atrial fibrillation, chronic sinusitis, inflammatory bowel disease, chronic liver diseases, psoriasis, eczema and arthritis. These were mapped to SNOMED codes where the top concept was the group identifier and then all direct children of that concept were examined and individually reviewed by two clinicians (Appendix 1). Physical health conditions were ascertained from data reported in text records from CRIS since 2007 until 1st August 2019 for each individual resulting in around 15m documents.

To extract SNOMED concepts from free text we used MedCAT,[29] a medical concept annotation toolkit capable of named entity recognition linking (NER+L) with contextualization. The base model used is described in Kraljevic et al.,[29] and has shown very good performance (F1=0.90). In a first step, the base model was enriched with concept names from UMLS with the purpose of

For peer review only

increasing recall and potentially catching all different name-forms for each concept. In a second step, MedCAT was trained in an unsupervised fashion on all the available documents to increase precision. In a third step, all the free text was annotated for the chosen SNOMED concepts. For each condition, 300 documents were randomly extracted, which resulted in a total of 6300 annotated documents.

Annotation of physical health conditions. To ensure consistent, high-quality gold standard and training data, we developed annotation guidelines based on series of iterative discussions including clinical and technical expertise. These guidelines, available upon request, were piloted and refined in preliminary stages. A relevant instance was defined as a mention of a physical health condition experienced by the patient and not negated. Each MedCAT detection was first validated as either correct/wrong - meaning the portion of text that was detected by MedCAT was either a correct/wrong detection of the relevant concept. Correct detections were further annotated with contextual annotations (or meta-annotations) for 'Diagnosis' and 'Status'. Diagnosis was used to determine if the detected concept is a patient related diagnosis, and Status if the detected concept is affirmed. Eight annotators were trained for this task and given the same instructions. MedCATtrainer[30] was used to facilitate manual annotations and each document was double annotated. Disagreements between annotators were further evaluated and resolved by a third annotator.

Training and validation. Once the dataset was annotated it was split into a training and validation set. For NER+L, 70% of the dataset was used for training and 30% for validation. For meta-annotations, 80% was used for training and 20% for validation. Hyperparameter optimization in both cases used a 10-fold cross validation on the training set.

For peer review only

Socio-demographics. Extracted data included sex, age at SMI diagnosis (15-24, 25-34, 35-44, 45-54, 55-64, 65-74, 75+), and ethnicity (White British, Irish, Black Caribbean (including mixed White and Black Caribbean and any other Black background), Black African (including mixed White and Black African), South Asian (Indian, Pakistani and Bangladeshi) and Other). Index of Multiple Deprivation (IMD) was extracted as a measure of neighbourhood socioeconomic status at the level of the 2011 Lower Layer Super Output Area (LSOA11; a standard postal unit with an average 1500 residents) corresponding to the individual's address at time of SMI diagnosis. Using the IMD, each LSOA11 is ranked from 1 (most deprived) to 32,844 (least deprived) based on seven Census-derived indicators, which was subsequently divided into quintiles.[31]

Disability. Disability was measured using Health of the Nation Outcome Scales (HoNOS) [32] which is a clinician-rated tool developed to measure health and social functioning. It includes 12 subscales: agitated behaviour; non-accidental self-injury; problem drinking or drug taking; cognitive problems; physical illness problems; problems associated with hallucinations or delusions; problems associated with depression; other mental and behaviour problems; problems with relationships; problems with activities of daily living; problems with living conditions; and problems with occupation and activities.[32] Total adjusted HoNOS scores of individuals at the first SMI diagnosis recorded in CRIS, or closest to that time, were used in this study. Higher scores for HoNOS indicate higher levels of impairment in the individual's functioning.

Statistical Analyses

To explore the suitability of MedCAT for extracting these physical health conditions from this cohort (objective 1), inter-rater agreement estimates were computed and performance, precision and recall per condition were estimated.

For peer review only

To examine the prevalence of these conditions in SMI across relevant factors and compare the most prevalent multimorbidity combinations for individuals with BD and SSD (objective 2). Descriptive statistics were derived for all the variables. Chi-square tests and Fisher's exact tests, with Bonferroni correction for multiple comparisons, were performed to explore associations and differences between BD and SSD.

To address our third objective (to investigate the association of multimorbidity and specific physical health with levels of disability), we performed series of hierarchical linear regressions. Models were adjusted by age and sex (Model 1), and then additionally adjusted by IMD (Model 2a) or SMI diagnosis (Model 2b). All analyses were performed using R 4.0.3 and RStudio 1.3.1093.

Patient and Public Involvement Statement

When designing this project, the Data Linkage Service User and Carer Advisory Group was consulted and followed up presenting preliminary results. This is a well-established Patient and Public Involvement Group set up by the Biomedical Research Center (BRC) at South London and Maudsley Trust NHS Foundation Trust (SLaM).[33]

Results

Inter-annotator agreement and model validation for data extraction

For each physical health condition, 300 documents were annotated to create a gold standard and training data specific to each condition. All 6300 instances across 21 health conditions were double annotated yielding an average inter-annotator agreement of 97% for NER+L, 82.70% for the meta-annotation Diagnosis and 78.08% for the meta-annotation Status. Precision, recall and F1 metrics of each modelled physical health condition are shown in Table 1. Coronary arteriosclerosis was not extracted as the number of positive mentions was too small for training and validation. Overall

For peer review only - http://bmjopen.bmj.com/site/about/guidelines.xhtml

meta-annotations performance results showed good performance for Diagnosis and Status (Supplemental Table 1).

PLEASE INSERT TABLE 1

Mapping of physical health conditions and comparison between SSD and BD

Our sample consisted of 17,500 individuals with SMI, of whom 74.4% were diagnosed with SSD and 25.6% with BD. A slight majority were male (53.6%), and most individuals had their first SMI diagnoses report under the age of 35 (42.8%). The White British group accounted for 35.7% of our sample, followed by Black Caribbean (18.2%) and Black African (12.0%). The South Asian and Irish groups were the smallest, with 3.1 and 2.0% respectively (Table 2). There were high levels of deprivation in the cohort, with over 60% falling into the lowest two national quintiles. Around 40% had at least one mention of a physical health condition and around 20% had two or more physical conditions. There were significant differences between BD and SSD for most of the socio-demographic characteristics and number of physical health conditions (Table 2 and Figure 1). Individuals with SSD were more likely to be men, from ethnic minorities, living in more deprived neighbourhoods and had a higher number of physical health conditions recorded compared to those with BD.

The three most common physical health conditions recorded were diabetes, hypertension and asthma (15.3%, 14.5% and 9.8% respectively), regardless of the specific SMI diagnoses (Supplemental Figure 1 within SSD and BD). When we compared individuals with SSD and BD, we found that the top 10 most prevalent health conditions were similar between groups but diabetes (SSD 17% vs BD 10.7%), hypertension (SSD 15.9% vs BD 10.4%) and epilepsy (SSD 5.0% vs BD 3.3%) prevalence rates were slightly higher for individuals with SSD while individuals with BD showed higher prevalence rates of migraine (BD 4.3% vs SSD 2.9%).

For peer review only - http://bmjopen.bmj.com/site/about/guidelines.xhtml

PLEASE INSERT TABLE 2 AND FIGURE 1 HERE

When we explored differences by sex in the whole cohort, we found that women were more likely to report hypertension, asthma, arthritis, eczema, and migraine compared to men (Supplemental Table 2). Within the individuals with SSD, women were found to be more likely to report higher rates of diabetes, chronic kidney disease (CKD), heart failure and transient ischaemic attack (TIA). Within individuals with BD, sex differences were only found for asthma, arthritis, migraine and ischemic heart disease. Women with BD were more likely to report asthma, arthritis and migraine while men with BD were more likely to report ischemic heart disease.

With regards to differences across age groups, we found higher prevalence rates of diabetes, hypertension, arthritis, cerebrovascular accident, ischaemic heart disease, COPD, CKD, Parkinson's disease, heart failure, atrial fibrillation and TIA in individuals in older age ranges, while asthma and migraine were more prevalent in younger age ranges (Supplemental Table 3, Supplemental Figure 2). We found similar results within individuals with SSD and BD (except for asthma and cerebrovascular accident in BD).

We found differences for ethnicity in individuals with diabetes, hypertension, asthma, arthritis, epilepsy, eczema, ischaemic heart disease, COPD and cerebrovascular accident (Figure 2, Supplemental Tables 4). Individuals from Black or South Asian minorities were more likely to show higher prevalence rates of diabetes and hypertension compared to those White British or Irish. Black Caribbean showed the highest prevalence rates of asthma or eczema among all other groups. Arthritis, COPD, epilepsy and IHD seem to be slightly more prevalent in White British or Irish, with epilepsy showing the highest prevalence rates among Irish. Similar trends were found within SSD and BD subgroups with diabetes rates higher in South Asians with BD (29.7%) compared to South Asians with SSD (20.7%); while diabetes rates in Black Caribbean with SSD

For peer review only

(24.4%) were higher than in those with BD (19.6%). With regards to social deprivation, we found that individuals with diabetes, hypertension, asthma, and COPD were more likely to be at higher levels of deprivation compared to those that did not have these specific conditions (Supplemental Table 5).

PLEASE INSERT FIGURE 2 AROUND HERE

Multimorbidity combinations for the whole cohort and SSD and BD subgroups

Table 3 summarizes the ten most common physical comorbidities in patients with SMI, their prevalence, the mean number of comorbidities, and the three most frequently associated comorbidities, for the total cohort and by SMI diagnosis. While there were no clear differences in the mean number of comorbidities by SMI diagnosis, the presence of one physical condition predisposed individuals to at least having one other condition. The mean number of comorbidities in the total cohort was 0.74 jumped to at least 2.20 in the presence of one of the ten most common comorbidities. The three most commonly associated physical comorbidities remained relatively consistent by SMI diagnosis, with a few exceptions. The prevalence of associated comorbidities with epilepsy were lower in BD than in SSD; there is a fairly different comorbidity profile in migraine between SMI diagnoses; and, a lower rate of diabetes in individuals with comorbid BD and COPD (when compared to SSD). The most common combination of conditions included diabetes, hypertension and asthma, regardless of their SMI diagnoses. Most individuals with these combinations of conditions were also likely to have arthritis. Figures 3, 4 and 5 show the most prevalent conditions for individuals with SMI and comorbid diabetes, hypertension and asthma, respectively.

PLEASE INSERT TABLE 3 AND FIGURES 3,4 AND 5 AROUND HERE

Association with disability

For peer review only

HoNOS descriptive statistics for the whole SMI cohort (Mean=10.40, SD=6.06) and for those with the ten most common physical comorbidities are shown in Table 4. Regression analyses showed that individuals with any of these conditions (except migraine) showed higher HoNOS scores, even after adjustments for age, sex, IMD or SMI diagnoses. We also examined whether simple and complex multimorbidity was associated with HoNOS total score and we found a strong positive association minimally attenuated after adjustments. Similar socio-demographics and trends were found within BD and SSD groups (Supplemental Tables 6-8). However, associations for diabetes, hypertension and ischaemic heart disease were fully attenuated after adjustments in the SSD group and associations for hypertension were also fully attenuated after adjustments in the BD group.

PLEASE INSERT TABLE 4 AROUND HERE

Discussion

The first objective of this study was to design and develop a suitable strategy to extract physical health conditions which could be easily replicated in future multimorbidity research using mental health electronic health records. The NLP strategy using MedCAT provided very good performance estimates for all the conditions extracted, which supports its suitability to extract data on physical health conditions from mental health clinical notes. These findings are consistent with previous research which has used MedCAT to extract data from hospital settings.[34,35] This resource should help to facilitate and promote research on multimorbidity using mental health records, in general, and has the potential for direct replication in other mental health trusts which have already deployed CRIS platforms.

Our second objective was to examine the prevalence of these conditions in SMI individuals and compare the most prevalent multimorbidity combinations between individuals with BD and SSD.

For peer review only - http://bmjopen.bmj.com/site/about/guidelines.xhtml

When we examined differences in socio-demographic variables by diagnosis, our findings were largely consistent with previous research,[3,36–39] although associations between ethnicity and BD are less established.[40] With regards to sociodemographic differences, we found that women with SSD were more likely to have diabetes, CKD, heart failure and TIA compared to men with SSD; and women with BD were more likely to have asthma, arthritis and migraine compared to men with BD. Previous research in this population showed mixed results. Some studies found higher prevalence of hypertension in women with SSD[41] and diabetes in women in BD,[42] and others did not find relevant sex differences.[43] Our findings suggest that there could be an increased risk for diabetes and hypertension for females with an SMI diagnosis, especially with SSD. Further research in this line is needed.

Ethnic differences were found for diabetes, hypertension, asthma, arthritis, epilepsy, eczema, ischaemic heart disease, COPD and cerebrovascular accident. Individuals from Black or South Asian minorities were more likely to show higher prevalence rates of diabetes and hypertension compared to White British or Irish. Black Caribbean showed the higher prevalence rates of asthma or eczema among all other groups. Arthritis, COPD, epilepsy and IHD seem to be slightly more prevalent in White British or Irish, with epilepsy showing the highest prevalence rates among Irish. Similar trends were found within SSD and BD subgroups with diabetes rates higher in South Asians with BD compared to South Asians with SSD, while diabetes rates in Black Caribbean with SSD were higher than in those with BD. These results largely mirror previous research in ethnicity.[44–50] When we examined social deprivation, individuals with diabetes, hypertension, asthma, and COPD were more likely to report the highest levels of deprivation regardless of their SMI diagnoses. Similar to ethnicity, these results are also consistent with findings in the general

For peer review only

population where higher levels of social deprivation are found in those with comorbid diabetes,[51,52] hypertension,[53] asthma[54] or COPD.[55]

Overall, in the whole SMI cohort, around 40% of the individuals had at least one mention of a physical health condition and close to 20% had two or more physical conditions, which could be labelled as complex multimorbidity. These findings provide evidence to support previous research suggestions about the increased probability of multimorbidity in this population.[3,17,56–59] Absolute numbers of physical health conditions were higher in patients with SSD than those with BD. Although direct comparisons require caution, our findings partially contrast with previous reports of higher number of physical comorbidities in individuals with BD.[3,38,39,41] Overall, the top ten most prevalent conditions in our SMI cohort were diabetes, hypertension, asthma, arthritis, epilepsy, cerebrovascular accident, eczema, migraine, ischaemic heart disease and COPD; and the most common combination of conditions included diabetes, hypertension and asthma, regardless of their SMI diagnoses. Moreover, those that had complex multimorbidity were also more likely to have cardiometabolic comorbidities such as diabetes and hypertension, which suggests that the cardiometabolic pathway might be one of the key explanatory mechanism underlying the association between physical multimorbidity and severe mental illness.[60,61] Future research should explore further the potential independent contribution of this pathway when focusing on individuals with complex multimorbidity. Furthermore, arthritis was the most frequent subsequent comorbidity for those with diabetes, hypertension and/or asthma, however for those with SSD and asthma, eczema slightly displaced arthritis in terms of prevalence. These findings might suggest that potential differences between SSD and BD phenotypes could be linked to underlying inflammatory pathways. Future research focusing on inflammatory biomarkers could be key to further our understanding of the potential differences between SSD and BD.

For peer review only

In addition, we examined the association between the top ten most prevalent conditions and disability levels. We found that not only multimorbidity was clearly associated with higher levels of disability but having any of these specific conditions was associated with higher levels of disability, even after adjusting for age, sex, deprivation or SMI diagnoses. Similar results were found when we examined the associations between multimorbidity and disability within SSD and BD groups. When we examined the independent association of each physical health condition and disability within groups, our results suggested that socio-demographic factors could have a greater impact in these associations in individuals with SSD. Although our results are not directly comparable with previous studies, they are in line with findings in previous research in ageing[62] or some specific SMI populations.[63,64] Further research is needed to understand the potential shared drivers of disability in individuals with BD and these conditions, in general, and diabetes and BD, in particular.

One of the main strengths of this study is the large comprehensive cohort of people with SMI drawn from a population with a high ethnic diversity, addressing the neglect of both ethnic minority groups and SMI in multimorbidity research. This is a key advantage of using EHRs from a large secondary mental health care provider and having the benefits of a data extraction strategy to access data on physical health conditions from the text fields of clinical notes. MedCAT development and deployment in CRIS text records will hopefully promote and facilitate future research in mental healthcare. However, further research is needed to validate this strategy in other EHRs sources using free text. Although our results are promising and the comparability of the findings with previous research provides some evidence of validity, further research is also needed to examine the cross-validity using primary care structured fields data. The present study is limited to individuals with SMI which does not allow us to compare comorbidity figures of SMI with other

For peer review only

common mental disorders and/or the general population. Future studies should replicate our data extraction strategy in other sex and age matched cohorts and explore the potential risks for subsequent health conditions maximizing the longitudinal nature of this data source. Furthermore, it is also important to note that individuals with more severe SMI may have more comprehensive textual data. Thus, our findings might be less representative of highly functioning individuals with less severe SMI. In addition, we acknowledge that although the conditions considered are within the most considered in multimorbidity research, future studies should consider a larger number of conditions and include rare diseases and all the conditions needed to calculate standard comorbidity scores such as Charlson or Exlihauser indexes which were not considered in this study (e.g., hemiplegia or paraplegia, peptic ulcer disease or AIDS/HIV). It should be noted that our study is one of the first, to our knowledge, to compare the associations between physical health comorbidities and disability in this traditionally neglected population and HoNOS is a widely used measure in secondary mental health services in the UK which provides us a general overview of disability in this population. However, we acknowledge that further research with more objective measures of disability is also needed to drive future policy in this population. To sum up, our study provides an overview of the most prevalent health conditions in SMI and underlines the need for further research into the origins of multimorbidity in this population, considering in more detail the nature of the SMI both in terms of severity and in terms of constituent diagnoses and/or symptomatic phenotype, given the apparent differences between BD and SSD. Our findings highlight multimorbidity as a driver of disability in this population, which also requires further mechanistic evaluation.

For peer review only

Acknowledgments: We would like to thank Megan Pritchard and Mathew Broadbent for their unvaluable contribution and support.

Funding statement:

RB is funded in part by grant MR/R016372/1 for the King's College London MRC Skills Development Fellowship programme funded by the UK Medical Research Council (MRC) and by grant IS-BRC-1215-20018 for the National Institute for Health Research (NIHR) Biomedical Research Centre at South London and Maudsley NHS Foundation Trust and King's College London. JD is funded by the Health Foundation working together with the Academy of Medical Sciences, for a Clinician Scientist Fellowship and by the ESRC in relation to the SEP-MD study [ES/S002715/1] and part supported by the ESRC Centre for Society and Mental Health at King's College London [ESRC Reference: ES/S012567/1]. DB is funded by a UKRI Innovation Fellowship as part of Health Data Research UK MR/S00310X/. AM is funded by Takeda California, Inc. RD, RS, AR are part-funded by the National Institute for Health Research (NIHR) Biomedical Research Centre at South London and Maudsley NHS Foundation Trust and King's College London. RD's work is supported by 1.National Institute for Health Research (NIHR) Biomedical Research Centre at South London and Maudsley NHS Foundation Trust and King's College London. 2. Health Data Research UK, which is funded by the UK Medical Research Council, Engineering and Physical Sciences Research Council, Economic and Social Research Council, Department of Health and Social Care (England), Chief Scientist Office of the Scottish Government Health and Social Care Directorates, Health and Social Care Research and Development Division (Welsh Government), Public Health Agency (Northern Ireland), British Heart Foundation and Wellcome Trust. 3. The National Institute for Health Research University College London Hospitals Biomedical Research Centre. This paper represents independent

For peer review only

research funded by the National Institute for Health Research (NIHR) Biomedical Research Centre at South London and Maudsley NHS Foundation Trust and King's College London and by the Health Foundation. The views expressed are those of the author(s) and not necessarily those of the NHS, the NIHR, the Department of Health, the MRC, ESRC or King's College London.

Authors contributions: RB conceived and designed the study. ZK, RB, AR and RS designed, validated the data extraction strategy. ZK, TS and AM developed the natural language processing algorithm and interface for the annotations. RB, ZK, JC, LM, NC, TS, AM, NS and TW were annotators. RB, ZK, SS, LL, JC, SA, RD and DB performed the data analyses and/or interpreted the results. RS and JD provided clinically relevant input over all the stages. RB, ZK and SS drafted the first version of the manuscript and all authors critically reviewed the manuscript and contributed to writing the final version.

Data sharing statement: Due to the confidential nature of free-text data, we are unable to make patient-level data available. CRIS was developed with extensive involvement from service users and adheres to strict governance frameworks managed by service users. It has passed a robust ethics approval pro-cess acutely attentive to the use of patient data. Specifically, this system was approved as a dataset for secondary data analysis on this basis by Oxfordshire Research Ethics Committee C (08/H06060/71). The data is de-identified and used in a data-secure format and all patients have the choice to opt-out of their anonymized data being used. Approval for data access can only be provided from the CRIS Oversight Committee at SLaM.

Competing interest statements: No competing interests.

Ethics statement: This project was approved by the CRIS Oversight Committee which is responsible for ensuring all research applications comply with ethical and legal guidelines. The

For peer review only

CRIS system enables access to anonymised electronic patient records for secondary analysis from SLaM and has full ethical approvals.

References

1 Firth J, Rosenbaum S, Galletly C, *et al.* Protecting physical health in people with mental illness – Authors' reply. *Lancet Psychiatry* 2019;**6**:890–1. doi:10.1016/S2215-0366(19)30387-6

2 Lawrence D, Kisely S, Pais J. The Epidemiology of Excess Mortality in People with Mental Illness. *Can J Psychiatry* 2010;**55**:752–60. doi:10.1177/070674371005501202

3 Reilly S, Olier I, Planner C, *et al.* Inequalities in physical comorbidity: a longitudinal comparative cohort study of people with severe mental illness in the UK. *BMJ Open* 2015;**5**:e009010. doi:10.1136/bmjopen-2015-009010

4 Woodhead C, Ashworth M, Schofield P, *et al.* Patterns of physical co-/multi-morbidity among patients with serious mental illness: a London borough-based cross-sectional study. *BMC Fam Pract* 2014;**15**:117. doi:10.1186/1471-2296-15-117

5 Filipcic IŠ, Bajic Ž, Filipcic I. The onset and accumulation of physical multimorbidity in severe and common mental disorders. *Curr Opin Psychiatry* 2020;**33**:484–90. doi:10.1097/YCO.0000000000000635

6 Cheung KL, ten Klooster PM, Smit C, *et al.* The impact of non-response bias due to sampling in public health studies: A comparison of voluntary versus mandatory recruitment in a Dutch national survey on adolescent health. *BMC Public Health* 2017;**17**. doi:10.1186/s12889-017-4189-8

For peer review only

7 Correll CU, Detraux J, Lepeleire JD, *et al.* Effects of antipsychotics, antidepressants and mood stabilizers on risk for physical diseases in people with schizophrenia, depression and bipolar disorder. *World Psychiatry* 2015;**14**:119–36. doi:https://doi.org/10.1002/wps.20204

8 Correll CU, Solmi M, Veronese N, *et al.* Prevalence, incidence and mortality from cardiovascular disease in patients with pooled and specific severe mental illness: a large-scale meta-analysis of 3,211,768 patients and 113,383,368 controls. *World Psychiatry* 2017;**16**:163–80. doi:https://doi.org/10.1002/wps.20420

9 DE HERT M, CORRELL CU, BOBES J, *et al.* Physical illness in patients with severe mental disorders. I. Prevalence, impact of medications and disparities in health care. *World Psychiatry* 2011;**10**:52–77.

10 Fan Z, Wu Y, Shen J, *et al.* Schizophrenia and the risk of cardiovascular diseases: A meta-analysis of thirteen cohort studies. *J Psychiatr Res* 2013;**47**:1549–56. doi:10.1016/j.jpsychires.2013.07.011

11 Pouget JG, Schizophrenia Working Group of the Psychiatric Genomics Consortium, Han B, *et al.* Cross-disorder analysis of schizophrenia and 19 immune-mediated diseases identifies shared genetic risk. *Hum Mol Genet* 2019;**28**:3498–513. doi:10.1093/hmg/ddz145

12 Rosenblat JD, McIntyre RS. Are medical comorbid conditions of bipolar disorder due to immune dysfunction? *Acta Psychiatr Scand* 2015;**132**:180–91. doi:https://doi.org/10.1111/acps.12414

13 Tylee DS, Sun J, Hess JL, *et al.* Genetic correlations among psychiatric and immune-related phenotypes based on genome-wide association data. *Am J Med Genet B Neuropsychiatr Genet* 2018;**177**:641–57. doi:https://doi.org/10.1002/ajmg.b.32652

For peer review only

14 Leo RJ, Singh J. Migraine headache and bipolar disorder comorbidity: A systematic review of the literature and clinical implications. *Scand J Pain* 2016;**11**:136–45. doi:10.1016/j.sjpain.2015.12.002

15 McKay KA, Tremlett H, Fisk JD, *et al.* Psychiatric comorbidity is associated with disability progression in multiple sclerosis. *Neurology* 2018;**90**:e1316–23. doi:10.1212/WNL.0000000000005302

16 Dorrington S, Carr E, Stevelink SAM, *et al.* Multimorbidity and fit note receipt in working-age adults with long-term health conditions. *Psychol Med* undefined/ed;:1–10. doi:10.1017/S0033291720002937

17 Kugathasan P, Wu H, Gaughran F, *et al.* Association of physical health multimorbidity with mortality in people with schizophrenia spectrum disorders: Using a novel semantic search system that captures physical diseases in electronic patient records. *Schizophr Res* 2020;**216**:408–15. doi:10.1016/j.schres.2019.10.061

18 Barnett K, Mercer SW, Norbury M, *et al.* Epidemiology of multimorbidity and implications for health care, research, and medical education: a cross-sectional study. *The Lancet* 2012;**380**:37–43. doi:10.1016/S0140-6736(12)60240-2

19 Cassell A, Edwards D, Harshfield A, *et al.* The epidemiology of multimorbidity in primary care: a retrospective cohort study. *Br J Gen Pract* 2018;**68**:e245–51. doi:10.3399/bjgp18X695465

20 Kuan V, Denaxas S, Gonzalez-Izquierdo A, *et al.* A chronological map of 308 physical and mental health conditions from 4 million individuals in the English National Health Service. *Lancet Digit Health* 2019;**1**:e63–77. doi:10.1016/S2589-7500(19)30012-3

For peer review only

21 Altamura AC, Buoli M, Pozzoli S. Role of immunological factors in the pathophysiology and diagnosis of bipolar disorder: Comparison with schizophrenia. *Psychiatry Clin Neurosci* 2014;**68**:21–36. doi:https://doi.org/10.1111/pcn.12089

22 Sahu A, Chowdhury HA, Gaikwad M, *et al.* Integrative network analysis identifies differential regulation of neuroimmune system in Schizophrenia and Bipolar disorder. *Brain Behav Immun - Health* 2020;**2**:100023. doi:10.1016/j.bbih.2019.100023

23 Walker J, Curtis V, Shaw P, *et al.* Schizophrenia and bipolar disorder are distinguished mainly by differences in neurodevelopment. *Neurotox Res* 2002;**4**:427. doi:10.1080/1029842021000022070

24 WHO | The world health report 2001 - Mental Health: New Understanding, New Hope. WHO. https://www.who.int/whr/2001/en/ (accessed 4 Jun 2021).

25 Prediction of disability in schizophrenia: Symptoms, cognition, and self-assessment - Philip D. Harvey, Martin T. Strassnig, Juliet Silberstein, 2019. https://journals.sagepub.com/doi/full/10.1177/2043808719865693 (accessed 4 Jun 2021).

26 Sanchez-Moreno J, Martinez-Aran A, Tabarés-Seisdedos R, *et al.* Functioning and Disability in Bipolar Disorder: An Extensive Review. *Psychother Psychosom* 2009;**78**:285–97. doi:10.1159/000228249

27 Stewart R, Soremekun M, Perera G, *et al.* The South London and Maudsley NHS Foundation Trust Biomedical Research Centre (SLAM BRC) case register: development and descriptive data. *BMC Psychiatry* 2009;**9**:51. doi:10.1186/1471-244X-9-51

28 ICD-10 Version:2016. https://icd.who.int/browse10/2016/en (accessed 9 Jun 2021).

For peer review only

29 Kraljevic Z, Searle T, Shek A, *et al.* Multi-domain Clinical Natural Language Processing with MedCAT: the Medical Concept Annotation Toolkit. *ArXiv201001165 Cs* Published Online First: 25 March 2021.http://arxiv.org/abs/2010.01165 (accessed 4 Jun 2021).

30 Searle T, Kraljevic Z, Bendayan R, *et al.* MedCATTrainer: A Biomedical Free Text Annotation Interface with Active Learning and Research Use Case Specific Customisation. *ArXiv190707322 Cs* Published Online First: 16 July 2019.http://arxiv.org/abs/1907.07322 (accessed 19 Aug 2020).

31 English indices of deprivation 2019: technical report. GOV.UK. https://www.gov.uk/government/publications/english-indices-of-deprivation-2019-technical-report (accessed 4 Jun 2021).

32 Wing JK, Beevor AS, Curtis RH, *et al.* Health of the Nation Outcome Scales (HoNOS): Research and development. *Br J Psychiatry* 1998;**172**:11–8. doi:10.1192/bjp.172.1.11

33 Jewell A, Pritchard M, Barrett K, *et al.* The Maudsley Biomedical Research Centre (BRC) data linkage service user and carer advisory group: creating and sustaining a successful patient and public involvement group to guide research in a complex area. *Res Involv Engagem* 2019;**5**:20. doi:10.1186/s40900-019-0152-4

34 Bean DM, Kraljevic Z, Searle T, *et al.* Angiotensin-converting enzyme inhibitors and angiotensin II receptor blockers are not associated with severe COVID-19 infection in a multi-site UK acute hospital trust. *Eur J Heart Fail* 2020;**22**:967–74. doi:https://doi.org/10.1002/ejhf.1924

35 Carr E, Bendayan R, Bean D, *et al.* Evaluation and improvement of the National Early Warning Score (NEWS2) for COVID-19: a multi-hospital study. *BMC Med* 2021;**19**:23. doi:10.1186/s12916-020-01893-3

For peer review only

36 Birgenheir DG, Ilgen MA, Bohnert ASB, *et al.* Pain conditions among veterans with schizophrenia or bipolar disorder. *Gen Hosp Psychiatry* 2013;**35**:480–4. doi:10.1016/j.genhosppsych.2013.03.019

37 Hardoon S, Hayes JF, Blackburn R, *et al.* Recording of Severe Mental Illness in United Kingdom Primary Care, 2000–2010. *PLOS ONE* 2013;**8**:e82365. doi:10.1371/journal.pone.0082365

38 Smith DJ, Langan J, McLean G, *et al.* Schizophrenia is associated with excess multiple physical-health comorbidities but low levels of recorded cardiovascular disease in primary care: cross-sectional study. *BMJ Open* 2013;**3**:e002808. doi:10.1136/bmjopen-2013-002808

39 Smith DJ, Martin D, McLean G, *et al.* Multimorbidity in bipolar disorder and undertreatment of cardiovascular disease: a cross sectional study. *BMC Med* 2013;**11**:263. doi:10.1186/1741-7015-11-263

40 Halvorsrud K, Nazroo J, Otis M, *et al.* Ethnic inequalities in the incidence of diagnosis of severe mental illness in England: a systematic review and new meta-analyses for non-affective and affective psychoses. *Soc Psychiatry Psychiatr Epidemiol* 2019;**54**:1311–23. doi:10.1007/s00127-019-01758-y

41 Oreški I, Jakovljević M, Aukst-Margetić B, *et al.* Comorbidity and multimorbidity in patients with schizophrenia and bipolar disorder: similarities and differencies. *Psychiatr Danub* 2012;**24**:80–5.

42 Patel RS, Virani S, Saeed H, *et al.* Gender Differences and Comorbidities in U.S. Adults with Bipolar Disorder. *Brain Sci* 2018;**8**:168. doi:10.3390/brainsci8090168

43 Severe mental illness (SMI) and physical health inequalities: briefing. GOV.UK. https://www.gov.uk/government/publications/severe-mental-illness-smi-physical-health-

For peer review only

inequalities/severe-mental-illness-and-physical-health-inequalities-briefing (accessed 4 Jun 2021).

44 Das-Munshi J, Ashworth M, Dewey ME, *et al.* Type 2 diabetes mellitus in people with severe mental illness: inequalities by ethnicity and age. Cross-sectional analysis of 588 408 records from the UK. *Diabet Med* 2017;**34**:916–24. doi:https://doi.org/10.1111/dme.13298

45 Gaughran F, Stahl D, Stringer D, *et al.* Effect of lifestyle, medication and ethnicity on cardiometabolic risk in the year following the first episode of psychosis: prospective cohort study. *Br J Psychiatry* 2019;**215**:712–9. doi:10.1192/bjp.2019.159

46 Lusignan S de, Alexander H, Broderick C, *et al.* The epidemiology of eczema in children and adults in England: A population-based study using primary care data. *Clin Exp Allergy* 2021;**51**:471–82. doi:https://doi.org/10.1111/cea.13784

47 Gilkes A, Ashworth M, Schofield P, *et al.* Does COPD risk vary by ethnicity? A retrospective cross-sectional study. *Int J Chron Obstruct Pulmon Dis* 2016;**11**:739–46. doi:10.2147/COPD.S96391

48 Godsland IF, Johnston DG, Chaturvedi N. Mechanisms of Disease: lessons from ethnicity in the role of triglyceride metabolism in ischemic heart disease. *Nat Clin Pract Endocrinol Metab* 2007;**3**:530–8. doi:10.1038/ncpendmet0530

49 Health Survey for England - 2004: Health of ethnic minorities, Headline results. NHS Digit. https://digital.nhs.uk/data-and-information/publications/statistical/health-survey-for-england/health-survey-for-england-2004-health-of-ethnic-minorities-headline-results (accessed 4 Jun 2021).

50 Public Health Profiles - PHE. https://fingertips.phe.org.uk/ (accessed 4 Jun 2021).

For peer review only

51 Zghebi SS, Steinke DT, Carr MJ, *et al.* Examining trends in type 2 diabetes incidence, prevalence and mortality in the UK between 2004 and 2014. *Diabetes Obes Metab* 2017;**19**:1537–45. doi:https://doi.org/10.1111/dom.12964

52 Diabetes Prevention Programme, 2017-18 Diagnoses and Demographics. NHS Digit. https://digital.nhs.uk/data-and-information/publications/statistical/national-diabetes-audit/diabetes-prevention-programme-2017-18 (accessed 4 Jun 2021).

53 Leng B, Jin Y, Li G, *et al.* Socioeconomic status and hypertension: a meta-analysis. *J Hypertens* 2015;**33**:221–9. doi:10.1097/HJH.0000000000000428

54 Gupta RP, Mukherjee M, Sheikh A, *et al.* Persistent variations in national asthma mortality, hospital admissions and prevalence by socioeconomic status and region in England. *Thorax* 2018;**73**:706–12. doi:10.1136/thoraxjnl-2017-210714

55 Simpson CR, Hippisley-Cox J, Sheikh A. Trends in the epidemiology of chronic obstructive pulmonary disease in England: a national study of 51 804 patients. *Br J Gen Pract* 2010;**60**:e277–84. doi:10.3399/bjgp10X514729

56 Carney CP, Jones L, Woolson RF. Medical comorbidity in women and men with schizophrenia. *J Gen Intern Med* 2006;**21**:1133–7. doi:10.1111/j.1525-1497.2006.00563.x

57 Gabilondo A, Alonso-Moran E, Nuño-Solinis R, *et al.* Comorbidities with chronic physical conditions and gender profiles of illness in schizophrenia. Results from PREST, a new health dataset. *J Psychosom Res* 2017;**93**:102–9. doi:10.1016/j.jpsychores.2016.12.011

58 Kugathasan P, Stubbs B, Aagaard J, *et al.* Increased mortality from somatic multimorbidity in patients with schizophrenia: a Danish nationwide cohort study. *Acta Psychiatr Scand* 2019;**140**:340–8. doi:10.1111/acps.13076

For peer review only

59 Severe mental illness (SMI) and physical health inequalities: briefing. GOV.UK. https://www.gov.uk/government/publications/severe-mental-illness-smi-physical-health-inequalities/severe-mental-illness-and-physical-health-inequalities-briefing (accessed 3 Mar 2020).

60 Firth J, Siddiqi N, Koyanagi A, *et al.* The Lancet Psychiatry Commission: a blueprint for protecting physical health in people with mental illness. *Lancet Psychiatry* 2019;**6**:675–712. doi:10.1016/S2215-0366(19)30132-4

61 Henderson DC, Vincenzi B, Andrea NV, *et al.* Pathophysiological mechanisms of increased cardiometabolic risk in people with schizophrenia and other severe mental illnesses. *Lancet Psychiatry* 2015;**2**:452–64. doi:10.1016/S2215-0366(15)00115-7

62 Quiñones AR, Markwardt S, Botoseneanu A. Multimorbidity Combinations and Disability in Older Adults. *J Gerontol A Biol Sci Med Sci* 2016;**71**:823–30. doi:10.1093/gerona/glw035

63 Desalegn D, Girma S, Abdeta T. Quality of life and its association with current substance use, medication non-adherence and clinical factors of people with schizophrenia in Southwest Ethiopia: a hospital-based cross-sectional study. *Health Qual Life Outcomes* 2020;**18**. doi:10.1186/s12955-020-01340-0

64 McIntyre RS, Konarski JZ, Soczynska JK, *et al.* Medical Comorbidity in Bipolar Disorder: Implications for Functional Outcomes and Health Service Utilization. *Psychiatr Serv* 2006;**57**:1140–4. doi:10.1176/ps.2006.57.8.1140

For peer review only

Table 1. MedCAT performance F1, precision and recall estimates for each physical health conditions.

**Physical Health Condition**	**F1**	**Precision**	**Recall**
Diabetes mellitus	0.98	0.99	0.98
Heart failure	0.97	0.97	0.96
Ischemic heart disease	0.98	0.97	0.99
Hypertensive disorder, systemic arterial	0.97	0.97	0.96
Chronic obstructive lung disease	0.94	0.97	0.92
Asthma	1.00	1.00	1.00
Chronic kidney disease	1.00	1.00	0.99
Cerebrovascular accident	0.96	0.94	0.98
Transient ischemic attack	0.91	0.82	1.00
Parkinson's disease	0.94	0.88	1.00
Multiple sclerosis	1.00	1.00	1.00
Epilepsy	0.93	1.00	0.85
Migraine	1.00	1.00	1.00
Atrial fibrillation	0.98	1.00	0.96
Chronic sinusitis	0.98	0.97	1.00
Inflammatory bowel disease	0.96	1.00	0.92
Chronic liver disease	1.00	1.00	1.00
Psoriasis	1.00	1.00	1.00
Eczema	0.94	1.00	0.88
Arthritis	1.00	1.00	1.00

For peer review only

Table 2. Socio-demographic characteristics and prevalence for physical health conditions for total cohort (N=17500) and by SMI diagnosis.

	**Total**	**SSD**	**BD**
**N (%)**	17500	13019 (74.4)	4481 (25.6)
**Sex*** **			
Female	8123 (46.4)	5421 (41.6)	2702 (60.3)
Male	9374 (53.6)	7596 (58.3)	1778 (39.7)
**Age at first SMI diagnosis*** **			
15 – 34	7497 (42.8)	5607 (43.1)	1890 (42.2)
35 – 44	3736 (21.3)	2792 (21.4)	944 (21.1)
45 – 54	2783 (15.9)	2057 (15.8)	726 (16.2)
55 – 64	1525 (8.7)	1067 (8.2)	458 (10.2)
65+	1959 (11.2)	1496 (11.5)	463 (10.3)
**Ethnicity*** **			
White British	6243 (35.7)	4008 (30.8)	2235 (49.9)
Black Caribbean	3182 (18.2)	2799 (21.5)	383 (8.5)
Black African	2094 (12.0)	1886 (14.5)	208 (4.6)
South Asian	549 (3.1)	421 (3.2)	128 (2.9)
Irish	346 (2.0)	240 (1.8)	106 (2.4)
Other	3846 (22.0)	2822 (21.7)	1024 (22.9)
Not stated	1240 (7.1)	843 (6.5)	397 (8.9)
**Index of multiple deprivation*** **			
1 (less deprived)	742 (4.2)	412 (3.2)	330 (7.4)
2	1384 (7.9)	887 (6.8)	497 (11.1)
3	3575 (20.4)	2503 (19.2)	1072 (23.9)
4	7073 (40.4)	5476 (42.1)	1597 (35.6)
5 (more deprived)	4033 (23.0)	3204 (24.6)	829 (18.5)
Unknown	693 (4.0)	537 (4.1)	156 (3.5)
**Number of conditions*** **			
No mentions	10468 (59.8)	7540 (57.9)	2928 (65.3)
One	3733 (21.3)	2888 (22.2)	845 (18.9)
Two	1795 (10.3)	1429 (11.0)	366 (8.2)
Three or more	1504 (8.6)	1162 (8.9)	342 (7.6)
**Physical conditions*** **			
Diabetes***	2686 (15.3)	2208 (17.0)	478 (10.7)
Hypertension***	2537 (14.5)	2070 (15.9)	467 (10.4)
Asthma	1722 (9.8)	1291 (9.9)	431 (9.6)
Arthritis	954 (5.5)	702 (5.4)	252 (5.6)
Epilepsy***	799 (4.6)	652 (5.0)	147 (3.3)
Cerebrovascular accident	728 (4.2)	573 (4.4)	155 (3.5)
Eczema	616 (3.5)	479 (3.7)	137 (3.1)

For peer review only

Migraine***	564 (3.2)	372 (2.9)	192 (4.3)
Ischemic heart disease	561 (3.2)	435 (3.3)	126 (2.8)
Chronic Obstructive Pulmonary disease	476 (2.7)	342 (2.6)	134 (3.0)
Chronic kidney disease*	279 (1.6)	179 (1.4)	100 (2.2)
Parkinson's disease	266 (1.5)	201 (1.5)	65 (1.5)
Heart failure**	222 (1.3)	187 (1.4)	35 (0.8)
Psoriasis	179 (1.0)	129 (1.0)	50 (1.1)
Atrial fibrillation	133 (0.8)	100 (0.8)	33 (0.7)
Transient Ischaemic Attack	130 (0.7)	91 (0.7)	39 (0.9)
Inflammatory Bowel Disease	40 (0.2)	25 (0.2)	15 (0.3)
Multiple sclerosis	32 (0.2)	19 (0.1)	13 (0.3)
Chronic liver disease	22 (0.1)	20 (0.2)	2 (0.0)
Chronic sinusitis	6 (0.0)	6 (0.0)	0 (0.0)

Note. *** $p < .001$; ** $p < .01$; * $p < .05$ for comparisons between BD and SSD groups

For peer review only

Table 3. Ten most prevalent conditions and associated comorbidities of the total cohort and by SMI diagnosis.

Condition		Preva-lence	Mean # of comorbidities	Three most frequent associated comorbidities		
	Total	-	0.74	1. Diabetes (15.3%)	2. HTN (14.5%)	3. Asthma (9.8%)
	SSD	-	0.77	1. Diabetes (17.0%)	2. HTN (15.9%)	3. Asthma (9.9%)
	BD	-	0.64	1. Diabetes (10.7%)	2. HTN (10.4%)	3. Asthma (9.6%)
**Diabetes**	*Total*	15.3%	2.35	1. HTN (42.1%)	2. Asthma (16.9%)	3. Arthritis (13.2%)
	SSD	17.0%	2.33	1. HTN (42.9%)	2. Asthma (16.3%)	3. Arthritis (12.7%)
	BD	10.7%	2.45	1. HTN (38.3%)	2. Asthma (19.5%)	3. Arthritis (15.5%)
**Hypertension**	*Total*	14.5%	2.50	1. Diabetes (44.5%)	2. Asthma (16.3%)	3. Arthritis (15.2%)
	SSD	15.9%	2.47	1. Diabetes (45.7%)	2. Asthma (16.1%)	3. Arthritis (14.7%)
	BD	10.4%	2.62	1. Diabetes (39.2%)	2. Arthritis (17.3 %)	3. Asthma (17.1%)
**Asthma**	*Total*	9.8%	2.27	1. Diabetes (26.3%)	2. HTN (24.0%)	3. Arthritis (11.9%)
	SSD	9.9%	2.27	1. Diabetes (27.9%)	2. HTN (25.9%)	3. Eczema (11.2%)
	BD	9.6%	2.29	1. Diabetes (21.6%)	2. HTN (18.6%)	3. Arthritis (15.1%)
**Arthritis**	*Total*	5.5%	2.78	1. HTN (40.5%)	2. Diabetes (37.1%)	3. Asthma (21.5%)
	SSD	5.4%	2.78	1. HTN (43.4%)	2. Diabetes (39.9%)	3. Asthma (19.9%)
	BD	5.6%	2.80	1. HTN (32.1%)	2. Diabetes (29.4%)	3. Asthma (25.8%)
**Epilepsy**	*Total*	4.6%	2.40	1. HTN (25.5%)	2. Diabetes (24.8%)	3. Asthma (21.4%)
	SSD	5.0%	2.42	1. HTN (27.3%)	2. Diabetes (26.4%)	3. Asthma (21.8%)
	BD	3.3%	2.31	1. Asthma (19.7%)	2. Diabetes (17.7%)	2. HTN (17.7%)
**CVA**	*Total*	4.2%	2.89	1. HTN (42.3%)	2. Diabetes (38.6%)	3. Asthma (15.4%)
	SSD	4.4%	2.83	1. HTN (42.8%)	2. Diabetes (38.9%)	3. Asthma (14.0%)
	BD	3.5%	3.09	1. HTN (40.6%)	2. Diabetes (37.4%)	3. Asthma (21.9)
**Eczema**	*Total*	3.5%	2.50	1. Asthma (32.5%)	2. Diabetes (26.9%)	3. HTN (23.1%)
	SSD	3.7%	2.45	1. Asthma (30.3%)	2. Diabetes (28.4%)	3. HTN (22.5%)
	BD	3.1%	2.64	1. Asthma (40.1%)	2. HTN (24.8%)	3. Diabetes (21.9%)
**Migraine**	*Total*	3.2%	2.20	1. Asthma (23.6%)	2. Diabetes (20.0%)	3. HTN (18.4%)
	SSD	2.9%	2.23	1. Diabetes (23.4%)	2. Asthma (21.5%)	3. HTN (20.2%)
	BD	4.3%	2.15	1. Asthma (27.6%)	2. HTN (15.1%)	3. Arthritis (14.1%)
**Ischaemic heart disease**	*Total*	3.2%	3.27	1. HTN (49.0%)	2. Diabetes (43.3%)	3. Asthma (20.3%)
	SSD	3.3%	3.28	1. HTN (51.0%)	2. Diabetes (44.6%)	3. Asthma (20.2%)
	BD	2.8%	3.24	1. HTN (42.1%)	2. Diabetes (38.9%)	3. Arthritis (22.2%)
**COPD**	*Total*	2.7%	3.22	1. HTN (39.7%)	2. Diabetes (38.9%)	3. Asthma (35.7%)
	SSD	2.6%	3.20	1. Diabetes (41.2%)	2. HTN (39.2%)	3. Asthma (35.7%)
	BD	3.0%	3.25	1. HTN (41.0%)	2. Asthma (35.8%)	3. Diabetes (32.8%)

Note. SSD: Schizophrenia Spectrum disorders. BD: Bipolar disorders. HTN: hypertension

Table 4. Associations between specific comorbidities, multimorbidity and HoNOS scores. Models were adjusted by age and sex (Model 1), and then additionally adjusted by IMD (Model 2a) or SMI diagnosis (Model 2b).

	HoNOS Mean (SD)	Unadjusted B (95% CI)	Model 1 B (95% CI)	Model 2a B (95% CI)	Model 2b B (95% CI)
**Physical comorbidities**					
**Diabetes**	10.93 (6.08)	0.652 (0.389 – 0.914)***	0.464 (0.198 – 0.730)***	0.485 (0.215 – 0.755)***	0.359 (0.094 – 0.625)**
Ref: No diabetes	10.28 (6.05)				
**Hypertension**	10.99 (6.02)	0.713 (0.446 – 0.980)***	0.401 (0.123 – 0.680)**	0.368 (0.084 – 0.652)*	0.297 (0.019 – 0.576)*
Ref: No hypertension	10.27 (6.06)				
**Asthma**	11.05 (6.16)	0.734 (0.417 – 1.051)***	0.806 (0.490 – 1.121)***	0.770 (0.450 – 1.091)***	0.804 (0.489 – 1.118)***
Ref: No asthma	10.31 (6.04)				
**Arthritis**	12.01 (6.19)	1.728 (1.322 – 2.134)***	1.570 (1.156 – 1.984)***	1.558 (1.142 – 1.974)***	1.567 (1.155 – 1.980)***
Ref: No arthritis	10.28 (6.03)				
**Epilepsy**	11.40 (6.19)	1.055 (0.596 – 1.514)***	1.066 (0.609 – 1.523)***	1.089 (0.628 – 1.549)***	0.982 (0.527 – 1.437)***
Ref: No epilepsy	10.35 (6.05)				
**CVA**	11.81 (6.22)	1.487 (1.022 – 1.951)***	1.195 (0.728 – 1.662)***	1.180 (0.712 – 1.649)***	1.162 (0.697 – 1.627)***
Ref: No CVA	10.33 (6.04)				
**Eczema**	11.12 (6.19)	0.753 (0.249 – 1.257)**	0.838 (0.336 – 1.340)***	0.728 (0.220 – 1.237)**	0.799 (0.299 – 1.299)**
Ref: No eczema	10.37 (6.05)				
**Migraine**	10.33 (5.73)	-0.078 (-0.600 – 0.445)	0.212 (-0.311 – 0.734)	0.227 (-0.300 – 0.754)	0.302 (-0.218 – 0.823)
Ref: No migraine	10.40 (6.07)				
**Ischaemic heart disease**	11.64 (5.92)	1.294 (0.766 – 1.822)***	0.864 (0.332 – 1.395)***	0.853 (0.318 – 1.387)**	0.849 (0.320 – 1.379)**
Ref: No ischaemic heart disease	10.35 (6.06)				
**COPD**	12.08 (5.78)	1.733 (1.158 – 2.309)***	1.326 (0.745 – 1.908)***	1.336 (0.750 – 1.923)***	1.397 (0.818 – 1.977)***
Ref: No COPD	10.34 (6.06)				
**Multimorbidity**					

**Number of comorbidities**		0.487 (0.406 – 0.567)***	0.423 (0.339 – 0.507)***	0.424 (0.339 – 0.510) ***	0.404 (0.320 – 0.488)***
**1 or more comorbidities**	10.96 (6.08)	1.053 (0.850 – 1.256)***	0.893 (0.685 – 1.101)***	0.895 (0.681 – 1.109)***	0.823 (0.615 – 1.031)***
Ref: No comorbidities	9.90 (5.99)				
**2 or more comorbidities**	11.34 (6.13)	1.214 (0.973 – 1.455)***	1.021 (0.772 – 1.269)***	1.020 (0.768 – 1.272)***	0.968 (0.720 – 1.216)***
Ref: Less than 1 comorbidities	10.12 (6.01)				

Note. *** $p < .001$; ** $p < .01$; * $p < .05$

For peer review only

Figure 1. Comparison of number of physical health comorbidities (a) and specific physical comorbidities (b) by SMI diagnosis.

Figure 2. Prevalence of the most prevalent physical health conditions across ethnicities within the SMI cohort and the SSD and BD subgroups.

Figure 3. Visualization of most prevalent comorbidities in individuals with SMI and comorbid diabetes.

Figure 4. Visualization of most prevalent comorbidities in individuals with SMI and comorbid hypertension.

Figure 5. Visualization of most prevalent comorbidities in individuals with SMI and comorbid asthma.

For peer review only

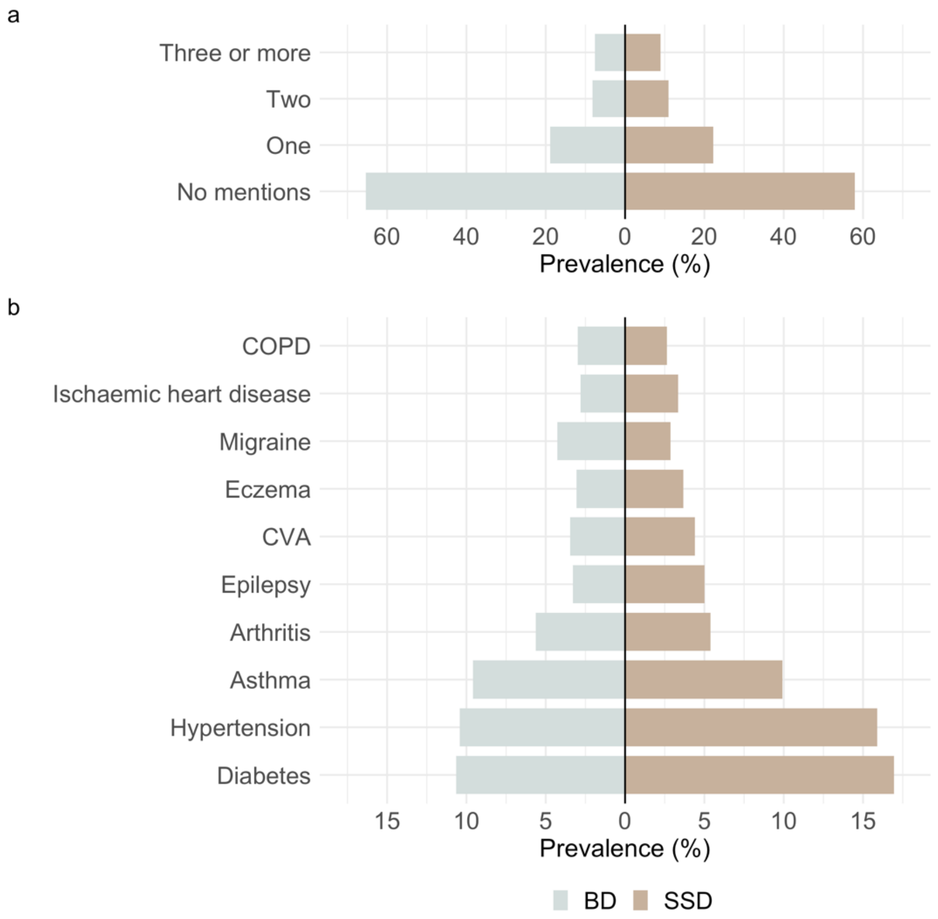
a
Three or more
Two
One
No mentions
60
40
20
0
20
40
60
Prevalence (%)
b
COPD
Ischaemic heart disease
Migraine
Eczema
CVA
Epilepsy
Arthritis
Asthma
Hypertension
Diabetes
15
10
5
0
5
10
15
Prevalence (%)
BD
SSD

Figure 2. Prevalence of the most prevalent physical health conditions across ethnicities within the SMI cohort and the SSD and BD subgroups.

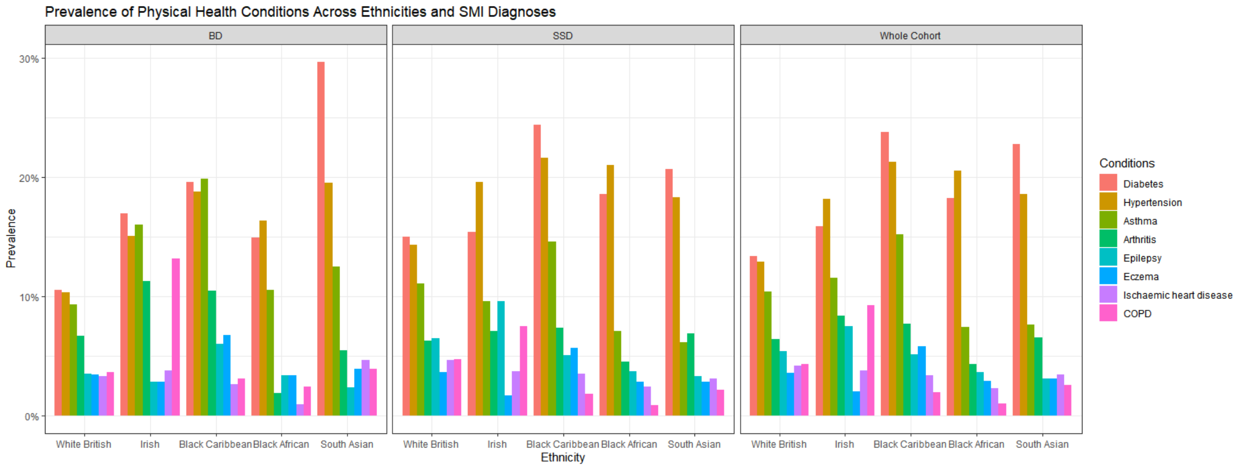

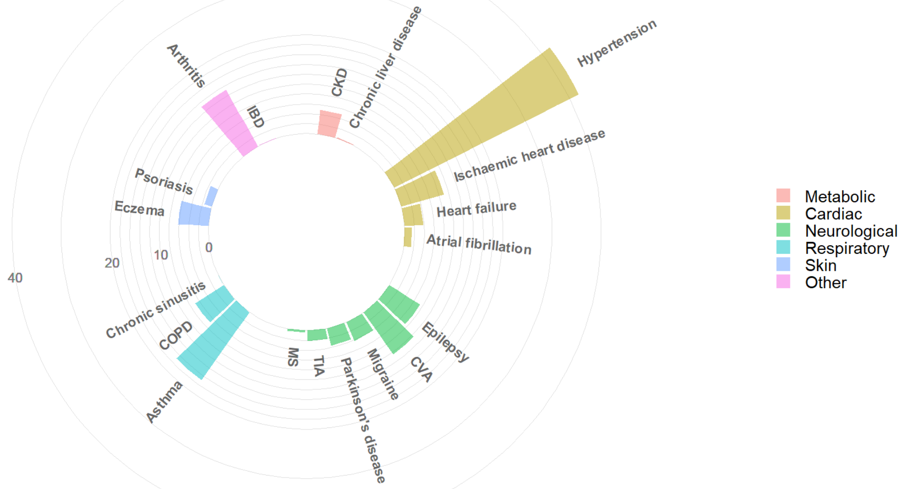
Hypertension
Ischaemic heart disease
Heart failure
Atrial fibrillation
Epilepsy
CVA
Migraine
Parkinson's disease
TIA
MS
Asthma
COPD
Chronic sinusitis
Eczema
Psoriasis
IBD
Arthritis
CKD
Chronic liver disease
40
20
10
0
Metabolic
Cardiac
Neurological
Respiratory
Skin
Other

review only

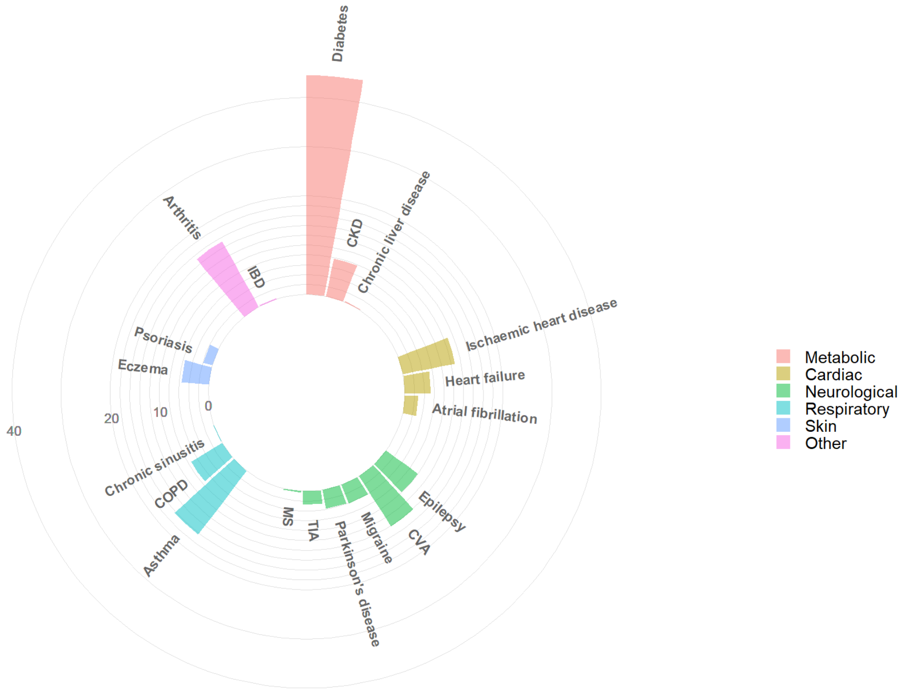

For peer review only - http://bmjopen.bmj.com/site/about/guidelines.xhtml

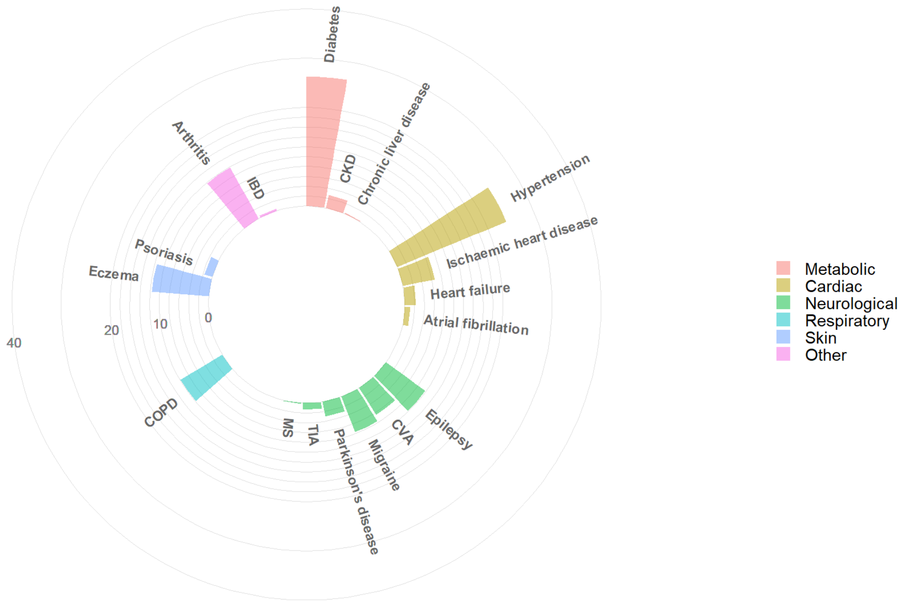
Diabetes
CKD
Chronic liver disease
Hypertension
Ischaemic heart disease
Heart failure
Atrial fibrillation
Epilepsy
CVA
Migraine
Parkinson's disease
TIA
MS
COPD
Eczema
Psoriasis
IBD
Arthritis
40
20
10
0
Metabolic
Cardiac
Neurological
Respiratory
Skin
Other

review only

Supplemental Material

Supplemental Tables

Supplemental Table 1. Meta-annotations performance results for Diagnosis and Status

Supplemental Table 2. Prevalence estimates and sex differences within the whole SMI cohort and SSD and BD subgroups.

Supplemental Table 3. Prevalence for each condition by age ranges at first SMI diagnosis for the whole SMI cohort and SSD and BD subgroups.

Supplemental Tables 4a, 4b and 4c. Prevalence for each condition across ethnicities for the whole SMI cohort and SSD and BD subgroups.

Supplemental Table 5. Social deprivation prevalence for each condition for the whole cohort and within SSD and BD.

Supplemental Table 6. Sociodemographic features of cohort in HoNOS subsample analysis

Supplemental Table 7. Associations between comorbidities and HoNOS scores in SSD.

Supplemental Table 8. Associations between comorbidities and HoNOS scores in BD.

Supplemental Figures

Supplemental Figure 1. Distribution of all conditions in the SMI cohort by SMI group.

Supplemental Figure 2. Prevalence rates for age at SMI diagnoses per condition and comparison between individuals with BD and SSD.

Appendix 1. SNOMED Container and Concept Level Groupings for physical health conditions included in this study.

For peer review only

Supplemental Tables

Supplemental Table 1. Meta-annotations performance results for Diagnosis and Status

Meta-Annotation	Values	F1 (macro/weighted)	P (macro/weighted)	R (macro/weighted)
Diagnosis	Patient / Other	0.94 / 0.94	0.95 / 0.95	0.92 / 0.94
Status	Affirmed / Other	0.89 / 0.98	0.94 / 0.98	0.85 / 0.98

For peer review only

Supplemental Table 2. Prevalence estimates and sex differences within the whole SMI cohort and SSD and BD subgroups.

	Total Cohort		SSD			BD		
	Female	Male	Female	Male	p-value	Female	Male	p- value
**Diabetes**	1282 (15.8)	1403 (15)	1021 (18.8)	1187 (15.6)	<.001	261 (9.7)	216 (12.1)	.190
**Hypertension *****	1325 (16.3)	1212 (12.9)	1060 (19.6)	1010 (13.3)	<.001	265 (9.8)	202 (11.4)	1.000
**Asthma ****	869 (10.7)	852 (9.1)	559 (10.3)	732 (9.6)	1.000	310 (11.5)	120 (6.7)	<.001
**Arthritis *****	657 (8.1)	297 (3.2)	471 (8.7)	231 (3.0)	<.001	186 (6.9)	66 (3.7)	<.001
**Epilepsy**	349 (4.3)	450 (4.8)	253 (4.7)	399 (5.3)	1.000	96 (3.6)	51 (2.9)	1.000
**Cerebrovascular diseases**	367 (4.5)	361 (3.9)	265 (4.9)	308 (4.1)	.499	102 (3.8)	53 (3.0)	1.000
**Eczema ***	331 (4.1)	285 (3.0)	238 (4.4)	241 (3.2)	<.01	93 (3.4)	44 (2.5)	1.000
**Migraine *****	369 (4.5)	194 (2.1)	217 (4.0)	155 (2.0)	<.001	152 (5.6)	39 (2.2)	<.001
**Ischaemic heart disease**	249 (3.1)	312 (3.3)	196 (3.6)	239 (3.1)	1.000	53 (2.0)	73 (4.2)	<.001
**Chronic Obstructive Lung diseases**	237 (2.9)	239 (2.5)	153 (2.8)	189 (2.5)	1.000	84 (3.1)	50 (2.8)	1.000
**Chronic Kidney disease**	158 (1.9)	121 (1.3)	104 (1.9)	75 (1.0)	<.001	54 (2.0)	46 (2.6)	1.000
**Parkinson disease**	113 (1.4)	153 (1.6)	81 (1.5)	120 (1.6)	1.000	32 (1.2)	33 (1.9)	1.000
**Heart failure**	123 (1.5)	99 (1.0)	105 (1.9)	82 (1.1)	.001	18 (0.7)	17 (1.0)	1.000
**Psoriasis**	76 (0.9)	103 (1.1)	45 (0.8)	84 (1.1)	1.000	31 (1.1)	19 (1.1)	1.000
**Atrial fibrillation**	65 (0.8)	68 (0.7)	50 (0.9)	50 (0.7)	1.000	15 (0.6)	18 (1.0)	1.000
**Transient Ischaemic Attack**	79 (1.0)	51 (0.5)	53 (1.0)	38 (0.5)	.037	26 (1.0)	13 (0.7)	1.000
**Inflammatory Bowel diseases**	26 (0.3)	14 (0.1)	18 (0.3)	7 (0.1)	.080	8 (0.3)	7 (0.4)	1.000

For peer review only

**Multiple Sclerosis**	17 (0.2)	15 (0.2)	9 (0.2)	10 (0.1)	-	8 (0.3)	5 (0.3)	-
**Chronic liver disease**	10 (0.1)	12 (0.1)	9	11 (0.1)	-	1 (0.0)	1 (0.1)	-
**Chronic Sinusitis**	3 (0.0)	3 (0.0)	3	3 (0.0)	-	0 (0.0)	0 (0.0)	-

Note.· *** p < .001; ** p < .01; * p < .05 for comparisons between male and female groups in the whole SMI cohort

For peer review only

Supplemental Table 3. Prevalence for each condition for the whole cohort and diagnoses subgroups and across age ranges at first SMI diagnosis.

For peer review only

	Total					SSD						BD					
	**15-34**	**35-44**	**45-54**	**55-64**	**65+**	**15-34**	**35-44**	**45-54**	**55-64**	**65+**		**15-34**	**35-44**	**45-54**	**55-64**	**65+**	
**Total**	7497	3736	2783	1525	1959	5607	2792	2057	1067	1496		1890	944	726	458	463	
**Diabetes*** **	697 (9.3)	559 (15.0)	531 (19.1)	371 (24.3)	528 (27.0)	591 (10.5)	479 (17.2)	439 (21.3)	284 (26.6)	415 (27.7)	$p < 0.001$	106 (5.6)	80 (8.5)	92 (12.7)	87 (19.0)	113 (24.4)	$p < 0.001$
**Hypertension*** **	472 (6.3)	426 (11.4)	514 (18.5)	416 (27.)3	709 (36.2)	412 (7.3)	372 (13.3)	411 (20.0)	313 (29.3)	562 (37.6)	$p < 0.001$	60 (3.2)	54 (5.7)	103 (14.2)	103 (22.5)	147 (31.7)	$p < 0.001$
**Asthma*** **	820 (10.9)	347 (9.3)	297 (10.7)	126 (8.3)	132 (6.7)	635 (11.3)	248 (8.9)	218 (10.6)	89 (8.3)	101 (6.8)	$p < 0.001$	185 (9.8)	99 (10.5)	79 (10.9)	37 (8.1)	31 (6.7)	$p = 1.000$
**Arthritis*** **	125 (1.7)	154 (4.1)	229 (8.2)	167 (11.0)	279 (14.2)	93 (1.7)	120 (4.3)	159 (7.7)	118 (11.1)	212 (14.2)	$p < 0.001$	32 (1.7)	34 (3.6)	70 (9.6)	49 (10.7)	67 (14.5)	$p < 0.001$
**Epilepsy**	339 (4.5)	188 (5.0)	133 (4.8)	67 (4.4)	72 (3.7)	276 (4.9)	154 (5.5)	112 (5.4)	55 (5.2)	55 (3.7)	1.000	63 (3.3)	34 (3.6)	21 (2.9)	12 (2.6)	17 (3.7)	1.000
**CVA*** **	166 (2.2)	112 (3.0)	122 (4.4)	114 (7.5)	214 (10.9)	143 (2.6)	87 (3.1)	95 (4.6)	84 (7.9)	164 (11.0)	$p < 0.001$	23 (1.2)	25 (2.6)	27 (3.7)	30 (6.6)	50 (10.8)	$p < 0.001$
**Eczema**	310 (4.1)	115 (3.1)	79 (2.8)	50 (3.3)	62 (3.2)	244 (4.4)	88 (3.2)	58 (2.8)	37 (3.5)	52 (3.5)	0.120	66 (3.5)	27 (2.9)	21 (2.9)	13 (2.8)	10 (2.2)	1.000
**Migraine*** **	300 (4.0)	122 (3.3)	87 (3.1)	32 (2.1)	23 (1.2)	207 (3.7)	73 (2.6)	53 (2.6)	24 (2.2)	15 (1.0)	$p < 0.001$	93 (4.9)	49 (5.2)	34 (4.7)	8 (1.7)	8 (1.7)	0.011
**Isc heart disease*** **	111 (1.5)	73 (2.0)	105 (3.8)	95 (6.2)	177 (9.0)	96 (1.7)	60 (2.1)	80 (3.9)	65 (6.1)	134 (9.0)	$p < 0.001$	15 (0.8)	13 (1.4)	25 (3.4)	30 (6.6)	43 (9.3)	$p < 0.001$
**COPD*** **	39 (0.5)	58 (1.6)	119 (4.3)	115 (7.5)	145 (7.4)	29 (0.5)	40 (1.4)	87 (4.2)	82 (7.7)	104 (7.0)	$p < 0.001$	10 (0.5)	18 (1.9)	32 (4.4)	33 (7.2)	41 (8.9)	$p < 0.001$
**CKD*** **	12 (0.2)	18 (0.5)	41 (1.5)	55 (3.6)	153 (7.8)	9 (0.2)	14 (0.5)	28 (1.4)	33 (3.1)	95 (6.4)	$p < 0.001$	3 (0.2)	4 (0.4)	13 (1.8)	22 (4.8)	58 (12.5)	-
**PD*** **	70 (0.9)	33 (0.9)	37 (1.3)	41 (2.7)	85 (4.3)	57 (1.0)	22 (0.8)	27 (1.3)	32 (3.0)	63 (4.2)	$p < 0.001$	13 (0.7)	11 (1.2)	10 (1.4)	9 (2.0)	22 (4.8)	$p < 0.001$
**HF*** **	18 (0.2)	30 (0.8)	42 (1.5)	40 (2.6)	92 (4.7)	17 (0.3)	30 (1.1)	38 (1.8)	29 (2.7)	73 (4.9)	$p < 0.001$	1 (0.1)	0 (0.0)	4 (0.6)	11 (2.4)	19 (4.1)	-
**Psoriasis**	67 (0.9)	33 (0.9)	37 (1.3)	17 (1.1)	25 (1.3)	54 (1.0)	25 (0.9)	25 (1.2)	9 (0.8)	16 (1.1)	1.000	13 (0.7)	8 (0.8)	12 (1.7)	8 (1.7)	9 (1.9)	0.450
**Atrial fibrillation*** **	14 (0.2)	7 (0.2)	12 (0.4)	19 (1.2)	81 (4.1)	11 (0.2)	6 (0.2)	8 (0.4)	12 (0.1)	63 (4.2)	$p < 0.001$	3 (0.20	1 (0.1)	4 (0.6)	7 (1.5)	18 (3.9)	-
**TIA*** **	20 (0.3)	18 (0.5)	19 (0.7)	23 (1.5)	50 (2.6)	15 (0.3)	15 (0.5)	13 (0.6)	14 (1.3)	34 (2.3)	$p < 0.001$	5 (0.3)	3 (0.3)	6 (0.8)	9 (2.0)	16 (3.5)	-
**IBD**	9 (0.1)	8 (0.2)	12 (0.4)	6 (0.4)	5 (0.3)	4 (0.1)	6 (0.2)	7 (0.3)	3 (0.3)	5 (0.3)	-	5 (0.3)	2 (0.2)	5 (0.7)	3 (0.7)	0 (0.0)	-
**MS**	6 (0.1)	8 (0.2)	11 (0.4)	5 (0.3)	2 (0.1)	4 (0.1)	4 (0.1)	8 (0.4)	3 (0.3)	0 (0.0)	-	2 (0.1)	4 (0.4)	3 (0.4)	2 (0.4)	2 (0.4)	-

For peer review only

**Chronic liver disease**	5 (0.1)	7 (0.2)	6 (0.2)	3 (0.2)	1 (0.1)	5 (0.1)	7 (0.3)	5 (0.2)	2 (0.2)	1 (0.1)	-	0 (0.0)	0 (0.0)	1 (0.1)	1 (0.2)	0 (0.0)	-
**Chronic sinusitis**	5 (0.1)	0 (0.0)	1 (0.0)	0 (0.0)	0 (0.0)	5 (0.1)	0 (0.0)	1 (0.0)	0 (0.0)	0 (0.0)	-	0 (0.0)	0 (0.0)	0 (0.0)	0 (0.0)	0 (0.0)	-

Note.· *** $p < .001$; ** $p < .01$; * $p < .05$ for comparisons between age groups within the SMI cohort.

For peer review only

Supplemental Tables 4. Prevalence for each condition for the whole cohort and diagnoses subgroups and across ethnicities

Table 4a. Prevalence for each condition for the whole cohort and ethnicity.

	White British n=6243 (35.7%)	Irish n=346 (2.0%)	Black Caribbean n=3182 (18.2%)	Black African n=2094 (12.0%)	South Asian n=549 (3.1%)	Other[§] n=2846 (22.0%)	Unknown[§] n=1240 (7.1%)	Statistics
**Diabetes**	837 (13.4)	55 (15.9)	757 (23.8)	382 (18.2)	125 (22.8)	461 (12.0)	69 (5.6)	χ^2 (4) = 172.49; *p* <0.001
**Hypertension**	804 (12.9)	63 (18.2)	678 (21.3)	430 (20.5)	102 (18.6)	400 (10.4)	60 (4.8)	χ^2 (4) = 137.98; *p* <0.001
**Asthma**	651 (10.4)	40 (11.6)	484 (15.2)	156 (7.4)	42 (7.7)	296 (7.7)	53 (4.3)	χ^2 (4) = 92.58; *p* <0.001
**Arthritis**	402 (6.4)	29 (8.4)	246 (7.7)	90 (4.3)	36 (6.6)	139 (3.6)	12 (1.0)	χ^2 (4) = 26.80; *p* <0.001
**Epilepsy**	339 (5.4)	26 (7.5)	164 (5.2)	77 (3.7)	17 (3.1)	147 (3.8)	29 (2.4)	χ^2 (4) = 19.02; *p* = 0.010
**Cerebrovascular accident**	274 (4.4)	20 (5.8)	184 (5.8)	92 (4.4)	25 (4.6)	116 (3.0)	17 (1.4)	χ^2 (4) = 10.60; *p* = 0.408
**Eczema**	222 (3.6)	7 (2.0)	185 (5.8)	61 (2.9)	17 (3.1)	108 (2.8)	16 (1.3)	χ^2 (4) = 41.93; *p* <0.001
**Migraine**	235 (3.8)	12 (3.5)	129 (4.1)	61 (2.9)	17 (3.1)	98 (2.5)	12 (1.0)	χ^2 (4) = 5.44; *p* >.99
**Ischemic heart disease**	261 (4.2)	13 (3.8)	108 (3.4)	48 (2.3)	19 (3.5)	99 (2.6)	13 (1.0)	χ^2 (4) = 16.74; *p* = 0.028
**COPD**	271 (4.3)	32 (9.2)	63 (2.0)	21 (1.0)	14 (2.6)	60 (1.6)	15 (1.2)	χ^2 (4) = 114.69; *p* <0.001
**CKD**	125 (2.0)	9 (2.6)	56 (1.8)	32 (1.5)	13 (2.4)	37 (1.0)	7 (0.6)	χ^2 (4) = 3.81; *p* >.99
**Parkinson's disease**	117 (1.9)	6 (1.7)	56 (1.8)	25 (1.2)	11 (2.0)	45 (1.2)	6 (0.5)	χ^2 (4) = 4.56; *p* >.99
**Heart failure**	88 (1.4)	8 (2.3)	55 (1.7)	29 (1.4)	8 (1.5)	31 (0.8)	3 (0.2)	χ^2 (4) = 3.16; *p* >.99
**Psoriasis**	103 (1.6)	4 (1.2)	12 (0.4)	6 (0.3)	5 (0.9)	44 (1.1)	5 (0.4)	*
**Atrial fibrillation**	69 (1.1)	9 (2.6)	23 (0.7)	11 (0.5)	2 (0.4)	15 (0.4)	4 (0.3)	*

For peer review only

**TIA**	58 (0.9)	7 (2.0)	24 (0.8)	15 (0.7)	7 (1.3)	13 (0.3)	6 (0.5)	*
**IBD**	24 (0.4)	1 (0.3)	2 (0.1)	2 (0.1)	1 (0.2)	9 (0.2)	1 (0.1)	*
**Multiple sclerosis**	21 (0.3)	1 (0.3)	3 (0.1)	1 (0.0)	0 (0.0)	4 (0.1)	2 (0.2)	*
**Chronic liver disease**	9 (0.1)	1 (0.3)	1 (0.0)	6 (0.3)	0 (0.0)	5 (0.1)	0 (0.0)	*
**Chronic sinusitis**	1 (0.0)	0 (0.0)	3 (0.1)	1 (0.0)	0 (0.0)	1 (0.0)	0 (0.0)	*

[§]Categories were dropped for statistical analysis. *χ2 test not performed due to small population sizes

Table 4b. Prevalence for each condition across ethnicities in SSD.

	White British n=4008 (30.8%)	Irish n=240 (1.8%)	Black Caribbean n=2799 (21.5%)	Black African n=1886 (14.5%)	South Asian n=421 (3.2%)	Other[§] n=2822 (21.7%)	Unknown[§] n=843 (6.5%)	Statistics
**Diabetes**	602 (15.0)	37 (15.4)	682 (24.4)	351 (18.6)	87 (20.7)	390 (13.8)	59 (7.0)	χ^2 (4) = 97.10; p <0.001
**Hypertension**	573 (14.3)	47 (19.6)	606 (21.7)	396 (21.0)	77 (18.3)	322 (11.4)	49 (5.8)	χ^2 (4) = 73.74; p <0.001
**Asthma**	443 (11.1)	23 (9.6)	408 (14.6)	134 (7.1)	26 (6.2)	217 (7.7)	40 (4.7)	χ^2 (4) = 75.96; p <0.001
**Arthritis**	252 (6.3)	17 (7.1)	206 (7.4)	86 (4.6)	29 (6.9)	103 (3.6)	9 (1.1)	χ^2 (4) = 15.48; p = 0.038
**Epilepsy**	261 (6.5)	23 (9.6)	141 (5.0)	70 (3.7)	14 (3.3)	117 (4.1)	26 (3.1)	χ^2 (4) = 32.45; p <0.001
**Cerebrovascular accident**	188 (4.7)	15 (6.2)	159 (5.7)	82 (4.3)	18 (4.3)	96 (3.4)	15 (1.8)	χ^2 (4) = 6.48; p >0.99
**Eczema**	145 (3.6)	4 (1.7)	159 (5.7)	54 (2.9)	12 (2.9)	93 (3.3)	12 (1.4)	χ^2 (4) = 33.32; p <0.001
**Migraine**	126 (3.1)	10 (4.2)	110 (3.9)	48 (2.5)	12 (2.9)	59 (2.1)	7 (0.8)	χ^2 (4) = 8.03; p = 0.904
**Ischemic heart disease**	187 (4.7)	9 (3.8)	98 (3.5)	46 (2.4)	13 (3.1)	74 (2.6)	8 (0.9)	χ^2 (4) = 19.15; p = 0.007
**COPD**	190 (4.7)	18 (7.5)	51 (1.8)	16 (0.8)	9 (2.1)	44 (1.6)	14 (1.7)	χ^2 (4) = 101.62; p <0.001
**CKD**	64 (1.6)	5 (2.1)	45 (1.6)	26 (1.4)	10 (2.4)	24 (0.9)	5 (0.6)	*
**Parkinson's disease**	79 (2.0)	4 (1.7)	49 (1.8)	21 (1.1)	6 (1.4)	36 (1.3)	6 (0.7)	*

For peer review only

**Heart failure**	65 (1.6)	8 (3.3)	50 (1.8)	28 (1.5)	7 (1.7)	26 (0.9)	3 (0.4)	*
**Psoriasis**	66 (1.6)	2 (0.8)	9 (0.3)	6 (0.3)	4 (1.0)	37 (1.3)	5 (0.6)	*
**Atrial fibrillation**	46 (1.1)	7 (2.9)	22 (0.8)	11 (0.6)	1 (0.2)	10 (0.4)	3 (0.4)	*
**TIA**	34 (0.8)	5 (2.1)	21 (0.8)	13 (0.7)	6 (1.4)	8 (0.3)	4 (0.5)	*
**IBD**	12 (0.3)	1 (0.4)	2 (0.1)	2 (0.1)	1 (0.2)	6 (0.2)	1 (0.1)	*
**Multiple sclerosis**	12 (0.3)	1 (0.4)	3 (0.1)	0 (0.0)	0 (0.0)	2 (0.1)	1 (0.1)	*
**Chronic liver disease**	7 (0.2)	1 (0.4)	1 (0.0)	6 (0.3)	0 (0.0)	5 (0.2)	0 (0.0)	*
**Chronic sinusitis**	1 (0.0)	0 (0.0)	3 (0.1)	1 (0.1)	0 (0.0)	1 (0.0)	0 (0.0)	*

§Categories were dropped for statistical analysis. *$\chi 2$ test not performed due to small population sizes

Table 4c. Prevalence for each condition across ethnicities in BD.

	**White British n=2235 (49.9%)**	**Irish n=106 (2.4%)**	**Black Caribbean n=383 (8.5%)**	**Black African n=208 (4.6%)**	**South Asian n=128 (2.9%)**	**Other§ n=1024 (22.9%)**	**Unknown§ n=397 (8.9%)**	**Statistics**
**Diabetes**	235 (10.5)	18 (17.0)	75 (19.6)	31 (14.9)	38 (29.7)	71 (6.9)	10 (2.5)	χ^2 (4) = 60.65; p <0.001
**Hypertension**	231 (10.3)	16 (15.1)	72 (18.8)	34 (16.3)	25 (19.5)	78 (7.6)	11 (2.8)	χ^2 (4) = 32.99; p <0.001
**Asthma**	208 (9.3)	17 (16.0)	76 (19.8)	22 (10.6)	16 (12.5)	79 (7.7)	13 (3.3)	χ^2 (4) = 39.95; p <0.001
**Arthritis**	150 (6.7)	12 (11.3)	40 (10.4)	4 (1.9)	7 (5.5)	36 (3.5)	3 (0.8)	χ^2 (4) = 19.09; p = 0.004
**Epilepsy**	78 (3.5)	3 (2.8)	23 (6.0)	7 (3.4)	3 (2.3)	30 (2.9)	3 (0.8)	*
**Cerebrovascular accident**	86 (3.8)	5 (4.7)	25 (6.5)	10 (4.8)	7 (5.5)	20 (2.0)	2 (0.5)	*
**Eczema**	77 (3.4)	3 (2.8)	26 (6.8)	7 (3.4)	5 (3.9)	15 (1.5)	4 (1.0)	*
**Migraine**	109 (4.9)	2 (1.9)	19 (5.0)	13 (6.2)	5 (3.9)	39 (3.8)	5 (1.3)	χ^2 (4) = 3.17; p >0.99
**Ischemic heart disease**	74 (3.3)	4 (3.8)	10 (2.6)	2 (1.0)	6 (4.7)	25 (2.4)	5 (1.3)	*

For peer review only

**COPD**	81 (3.6)	14 (13.2)	12 (3.1)	5 (2.4)	5 (3.9)	16 (1.6)	1 (0.3)	*
**CKD**	61 (2.7)	4 (3.8)	11 (2.9)	6 (2.9)	3 (2.3)	13 (1.3)	2 (0.5)	*
**Parkinson's disease**	38 (1.7)	2 (1.9)	7 (1.8)	4 (1.9)	5 (3.9)	9 (0.9)	0 (0.0)	*
**Heart failure**	23 (1.0)	0 (0.0)	5 (1.3)	1 (0.5)	1 (0.8)	5 (0.5)	0 (0.0)	*
**Psoriasis**	37 (1.7)	2 (1.9)	3 (0.8)	0 (0.0)	1 (0.8)	7 (0.7)	0 (0.0)	*
**Atrial fibrillation**	23 (1.0)	2 (1.9)	1 (0.3)	0 (0.0)	1 (0.8)	5 (0.5)	1 (0.3)	*
**TIA**	24 (1.1)	2 (1.9)	3 (0.8)	2 (1.0)	1 (0.8)	5 (0.5)	2 (0.5)	*
**IBD**	12 (0.5)	0 (0.0)	0 (0.0)	0 (0.0)	0 (0.0)	3 (0.3)	0 (0.0)	*
**Multiple sclerosis**	9 (0.4)	0 (0.0)	0 (0.0)	1 (0.5)	0 (0.0)	2 (0.2)	1 (0.3)	*
**Chronic liver disease**	2 (0.1)	0 (0.0)	0 (0.0)	0 (0.0)	0 (0.0)	0 (0.0)	0 (0.0)	*
**Chronic sinusitis**	0 (0.0)	0 (0.0)	0 (0.0)	0 (0.0)	0 (0.0)	0 (0.0)	0 (0.0)	*

[§]Categories were dropped for statistical analysis. *χ2 test not performed due to small population sizes

For peer review only

Supplemental Table 5. Social deprivation prevalence for each condition for the whole cohort and within SSD and BD.

	Total cohort						SSD							AD						
	1 - least deprived n (%)	2 n (%)	3 n (%)	4 n (%)	5 - most deprived n (%)	Not stated[§] n (%)	1 - least deprived n (%)	2 n (%)	3 n (%)	4 n (%)	5 - most deprived n (%)	Not stated[§] n (%)	Chi square	1 - least deprived n (%)	2 n (%)	3 n (%)	4 n (%)	5 - most deprived n (%)	Not stated[§] n (%)	Chi square
**Total**	742 (100)	1384 (100)	3575 (100)	7073 (100)	4033 (100)	693 (100)	412 (100)	887 (100)	2503 (100)	5476 (100)	3204 (100)	537 (100)		330 (100)	497 (100)	1072 (100)	1597 (100)	829 (100)	156 (100)	
**Diabetes *****	62 (8.4)	169 (12.2)	528 (14.8)	1201 (17.0)	698 (17.3)	28 (4.0)	49 (11.9)	117 (13.2)	409 (16.3)	1019 (18.6)	594 (18.5)	20 (3.7)	$X^2(4)$ = 29.7; p<0.001	13 (3.9)	52 (10.5)	119 (11.1)	182 (11.4)	104 (12.5)	8 (5.1)	$X^2(4)$ = 19.4; p=0.007
**Hypertension *****	77 (10.4)	154 (11.1)	468 (13.1)	1189 (16.8)	627 (15.5)	22 (3.2)	51 (12.4)	108 (12.2)	368 (14.7)	1008 (18.4)	517 (16.1)	18 (3.4)	$X^2(4)$ = 37.8; p<0.001	26 (7.9)	46 (9.3)	100 (9.3)	181 (11.3)	110 (13.3)	4 (2.6)	$X^2(4)$ = 12.3; p=0.17
**Asthma *****	42 (5.7)	95 (6.9)	361 (10.1)	758 (10.7)	432 (10.7)	34 (4.9)	27 (6.6)	61 (6.9)	251 (10.0)	589 (10.8)	337 (10.5)	26 (4.8)	$X^2(4)$ = 19.0; p=0.01	15 (4.5)	34 (6.8)	110 (10.3)	169 (10.6)	95 (11.5)	8 (5.1)	$X^2(4)$ = 19.2; p=0.008
**Arthritis**	36 (4.9)	56 (4.0)	188 (5.3)	411 (5.8)	256 (6.3)	7 (1.0)	18 (4.4)	36 (4.1)	135 (5.4)	312 (5.7)	197 (6.1)	4 (0.7)	$X^2(4)$ = 7.30; p>0.99	18 (5.5)	20 (4.0)	53 (4.9)	99 (6.2)	59 (7.1)	3 (1.9)	$X^2(4)$ = 7.51; p>0.99
**Epilepsy**	32 (4.3)	50 (3.6)	160 (4.5)	344 (4.9)	202 (5.0)	11 (1.2)	24 (5.8)	40 (4.5)	123 (4.9)	292 (5.3)	164 (5.1)	9 (1.7)	$X^2(4)$ = 1.79; p>0.99	8 (2.4)	10 (2.0)	37 (3.5)	52 (3.3)	38 (4.6)	2 (1.3)	$X^2(4)$ = 7.59; p>0.99
**CVA**	19 (2.6)	52 (3.8)	146 (4.1)	328 (4.6)	180 (4.5)	3 (0.4)	12 (2.9)	33 (3.7)	107 (4.3)	272 (5.0)	146 (4.6)	3 (0.6)	$X^2(4)$ = 6.55; p>0.99	7 (2.1)	19 (3.8)	39 (3.6)	56 (3.5)	34 (4.1)	0	$X^2(4)$ = 2.80; p>0.99
**Eczema**	22 (3.0)	45 (3.3)	120 (3.4)	273 (3.9)	147 (3.6)	9 (1.3)	17 (4.1)	34 (3.8)	86 (3.4)	212 (3.9)	123 (3.8)	7 (1.3)	$X^2(4)$ = 1.11; p>0.99	5 (1.5)	11 (2.2)	34 (3.2)	61 (3.8)	24 (2.9)	2 (1.3)	$X^2(4)$ = 6.90; p>0.99
**Migraine**	17 (2.3)	39 (2.8)	121 (3.4)	243 (3.4)	140 (3.5)	4 (0.6)	11 (2.7)	20 (2.3)	63 (2.5)	176 (3.2)	99 (3.1)	3 (0.6)	$X^2(4)$ = 4.79; p>0.99	6 (1.8)	19 (3.8)	58 (5.4)	67 (4.2)	41 (4.9)	1 (0.6)	$X^2(4)$ = 8.94; p=0.69
**Ischaemic heart disease**	20 (2.7)	48 (3.5)	103 (2.9)	240 (3.4)	143 (3.5)	7 (1.0)	9 (2.2)	33 (3.7)	74 (3.0)	194 (3.5)	120 (3.7)	5 (0.9)	$X^2(4)$ = 4.99; p>0.99	11 (3.3)	15 (3.0)	29 (2.7)	46 (2.9)	23 (2.8)	2 (1.3)	$X^2(4)$ = 0.43; p>0.99
**COPD ****	6 (0.8)	35 (2.5)	83 (2.3)	208 (2.9)	139 (3.4)	5 (0.7)	3 (0.7)	22 (2.5)	59 (2.4)	159 (2.9)	95 (3.0)	4 (0.7)	$X^2(4)$ = 9.07; p=0.77	3 (0.9)	13 (2.6)	24 (2.2)	49 (3.1)	44 (5.3)	1 (0.6)	$X^2(4)$ = 21.9; p=0.002

For peer review only

**CKD**	8 (1.1)	22 (1.6)	46 (1.3)	128 (1.8)	74 (1.8)	1 (0.1)	4 (1.0)	9 (1.0)	28 (1.1)	81 (1.5)	56 (1.7)	1 (0.2)	$X^2(4)$ = 5.83; p>0.99	4 (1.2)	13 (2.6)	18 (1.7)	47 (2.9)	18 (2.2)	0	$X^2(4)$ = 6.76; p>0.99
**PD**	9 (1.2)	22 (1.6)	51 (1.4)	121 (1.7)	60 (1.5)	3 (0.4)	5 (1.2)	16 (1.8)	33 (1.3)	97 (1.8)	48 (1.5)	2 (0.4)	$X^2(4)$ = 3.13; p>0.99	4 (1.2)	6 (1.2)	18 (1.7)	24 (1.5)	12 (1.4)	1 (0.6)	---
**Heart failure**	10 (1.3)	12 (0.9)	47 (1.3)	92 (1.3)	58 (1.4)	3 (0.4)	7 (1.7)	10 (1.1)	39 (1.6)	78 (1.4)	52 (1.6)	1 (0.2)	$X^2(4)$ = 1.54; p=0.82	3 (0.9)	2 (0.4)	8 (0.7)	14 (0.9)	6 (0.7)	2 (1.3)	---
**Psoriasis**	11 (1.5)	12 (0.9)	33 (0.9)	71 (1.0)	48 (1.2)	4 (0.6)	4 (1.0)	6 (0.7)	24 (1.0)	54 (1.0)	37 (1.2)	4 (0.7)	---	7 (2.1)	6 (1.2)	9 (0.8)	17 (1.1)	11 (1.3)	0	---
**Atrial fibrillation**	10 (1.3)	11 (0.8)	27 (0.8)	53 (0.7)	29 (0.7)	3 (0.4)	6 (1.5)	7 (0.8)	21 (0.8)	42 (0.8)	23 (0.7)	1 (0.2)	---	4 (1.2)	4 (0.8)	6 (0.6)	11 (0.7)	6 (0.7)	2 (1.3)	---
**TIA**	3 (0.3)	17 (1.0)	27 (0.6)	55 (0.6)	28 (0.5)	0	1 (0.2)	9 (1.0)	16 (0.6)	43 (0.8)	22 (0.7)	0	---	2 (0.6)	8 (1.6)	11 (1.0)	12 (0.8)	6 (0.7)	0	---
**IBD**	1 (0.1)	4 (0.3)	10 (0.3)	12 (0.2)	13 (0.3)	0	0	1 (0.1)	7 (0.3)	7 (0.1)	10 (0.3)	0	---	1 (0.3)	3 (0.6)	3 (0.3)	5 (0.3)	3 (0.4)	0	---
**MS**	4 (0.5)	2 (0.1)	7 (0.2)	14 (0.2)	4 (0.1)	1 (0.1)	2 (0.5)	2 (0.2)	2 (0.1)	9 (0.2)	3 (0.1)	1 (0.2)	---	2 (0.6)	0	5 (0.5)	5 (0.3)	1 (0.1)	0	---
**Chronic liver disease**	0	1 (0.1)	8 (0.2)	8 (0.1)	5 (0.1)	0	0	0	8 (0.3)	8 (0.1)	4 (0.1)	0	---	0	1 (0.2)	0	0	1 (0.1)	0	---
**Chronic sinusitis**	0	0	1 (0.0)	2 (0.0)	3 (0.1)	0	0	0	1 (0.0)	2 (0.0)	3 (0.1)	0	---	0	0	0	0	0	0	---

*, p<0.05; **, p≤0.01; ***, p≤0.001; CVA, cerebrovascular accident; COPD, chronic obstructive lung disease; CKD, chronic kidney disease; PD, Parkinson's disease; TIA, transient ischemic attack; IBD, inflammatory bowel disease; MS, multiple sclerosis

For peer review only

For peer review only

Supplemental Table 6. Sociodemographic features of cohort in HoNOS subsample analysis

	Total n (%)	SSD n (%)	BD n (%)
**Totals n (%)**	13650 (100.0)	10384 (76.1)	3266 (23.9)
**Sex*** **			
Female	6537 (47.9)	4526 (69.2)	2011 (30.8)
Male	7112 (52.1)	5858 (82.4)	1254 (17.6)
**Age at first SMI diagnosis*** **			
15 – 34	5821 (42.6)	4420 (42.6)	1401 (42.9(
35 – 44	3430 (25.1)	2183 (75.3)	716 (24.7)
45 – 54	2082 (15.3)	1594 (76.6)	488 (23.4)
55 – 64	1089 (8.0)	811 (74.5)	278 (25.5)
65+	1759 (12.9)	1376 (13.3)	383 (11.7)
**Ethnicity*** **			
White British	4571 (33.5)	2961 (64.8)	1610 (35.2)
Black Caribbean	2849 (20.9)	2515 (88.3)	334 (11.7)
Black African	1852 (13.6)	1679 (90.7)	173 (9.3)
South Asian	436 (3.2)	338 (77.5)	98 (22.5)
Irish	286 (2.1)	197 (68.9)	89 (31.1)
Other[§]	3127 (22.9)	2327 (74.4)	800 (25.6)
Not stated[§]	529 (3.9)	367 (3.5)	162 (5.0)
**Index of multiple deprivation*** **			
1 (least deprivation)	380 (2.8)	237 (62.4)	143 (37.6)
2	883 (6.5)	581 (65.8)	302 (34.2)
3	2786 (20.4)	1994 (71.6)	792 (28.4)
4	5947 (43.6)	4654 (78.3)	1293 (21.7)
5 (most deprivation)	3308 (24.2)	2650 (80.1)	658 (19.9)
Unknown[§]	346 (2.5)	268 (77.5)	78 (22.5)
**Physical conditions*** **			
No mentions	7232 (53.0)	5320 (73.6)	1912 (26.4)
One	3295 (24.1)	2596 (78.8)	699 (21.2)
Two	1669 (12.2)	1340 (80.3)	329 (19.7)
Three or more	1454 (10.7)	1128 (77.6)	326 (22.4)

Note. *** $p < .001$ *for comparisons between BD and SSD groups.* [§]Not included in analyses.

For peer review only

Supplemental Table 7. Associations between comorbidities and HoNOS scores in SSD.

Comorbidity	HoNOS Score	Unadjusted	M1	M2a
	Mean (SD)	B (95% CI)	B (95% CI)	B (95% CI)
**Whole cohort**	10.75 (6.10)			
**Diabetes**	11.04 (6.10)	0.360 (0.067 – 0.654)**	0.224 (-0.073 – 0.522)	0.300 (-0.002 – 0.602)
Ref: No diabetes	10.68 (6.10)			
**Hypertension**	11.17 (6.05)	0.519 (0.220 – 0.818)***	0.264 (-0.048 – 0.575)	0.324 (0.006 – 0.641)*
Ref: No hypertension	10.65 (6.11)			
**Asthma**	11.19 (6.11)	0.492 (0.126 – 0.859)**	0.551 (0.185 – 0.918)**	0.530 (0.158 – 0.903)**
Ref: No asthma	10.70 (6.10)			
**Arthritis**	12.08 (6.21)	1.422 (0.948– 1.895)***	1.259 (0.775 – 1.742)***	1.274 (0.788 – 1.759)***
Ref: No arthritis	10.66 (6.08)			
**Epilepsy**	11.53 (6.24)	0.827 (0.316 – 1.337)***	0.850 (0.341 – 1.358)***	0.856 (0.341 – 1.370)***
Ref: No epilepsy	10.71 (6.09)			
**CVA**	11.87 (6.23)	1.178 (0.651 – 1.705)***	0.913 (0.383 – 1.443)***	0.921 (0.388 – 1.454)**
Ref: No CVA	10.69 (6.09)			
**Eczema**	11.48 (6.25)	0.763 (0.188 – 1.338)**	0.846 (0.273 – 1.420)**	0.754 (0.172 – 1.336)*
Ref: No eczema	10.72 (6.09)			
**Migraine**	10.71 (5.87)	-0.041 (-0.683 – 0.602)	0.196 (-0.447 – 0.839)	0.192 (-0.458 – 0.842)
Ref: No migraine	10.75 (6.11)			
**Ischaemic heart disease**	11.59 (5.91)	0.874 (0.274 – 1.474)**	0.520 (-0.083 – 1.124)	0.477 (-0.132 – 1.085)
Ref: No ischaemic heart disease	10.72 (6.11)			
**COPD**	12.15 (5.70)	1.444 (0.760– 2.128)***	1.045 (0.354 – 1.736)**	1.043 (0.343 – 1.742)**
Ref: No COPD	10.71 (6.11)			
**Number of comorbidities**		0.387 (0.294 – 0.481)***	0.328 (0.231 – 0.425)***	0.342 (0.243 – 0.441) ***
**1 or more comorbidities**	11.13 (6.11)	0.745 (0.511 –0.980)***	0.607 (0.367 – 0.847)***	0.657 (0.410 – 0.903)***
Ref: No comorbidities	10.39 (6.07)			
**2 or more comorbidities**	11.45 (6.16)	0.921 (0.646 – 1.196)***	0.747 (0.464 – 1.030)***	0.771 (0.484 – 1.059)***
Ref: Less than 1 comorbidities	10.53 (6.07)			

Supplemental Table8. Associations between comorbidities and HoNOS scores in BD.

Comorbidity	HoNOS Score	Unadjusted	M1	M2a
	Mean (SD)	B (95% CI)	B (95% CI)	B (95% CI)
**Whole cohort**	9.28 (5.77)			
**Diabetes**	10.941 (5.97)	1.304 (0.720 – 1.887)***	0.968 (0.370 – 1.565)**	0.961 (0.355 – 1.568)**
Ref: No diabetes	9.11 (5.72)			
**Hypertension**	10.13 (5.82)	0.972 (0.384 – 1.561)**	0.446 (-0.182 – 1.074)	0.277 (-0.359 – 0.912)
Ref: No hypertension	9.15 (5.76)			
**Asthma**	10.61 (6.27)	1.512 (0.900 – 2.124)***	1.597 (0.985 – 2.210)***	1.503 (0.887 – 2.120)***
Ref: No asthma	9.10 (5.68)			
**Arthritis**	11.81 (6.13)	2.725 (1.960 – 3.490)***	2.487 (1.702 – 3.273)***	2.396 (1.606 – 3.186)***
Ref: No arthritis	9.09 (5.70)			
**Epilepsy**	10.77 (5.96)	1.552 (0.513 – 2.591)**	1.587 (0.551 – 2.624)**	1.496 (0.466 – 2.527)**
Ref: No epilepsy	9.22 (5.76)			
**CVA**	11.60 (6.16)	2.425 (1.464 – 3.386)***	2.098 (1.124 – 3.071)***	2.030 (1.064 – 2.996)***
Ref: No CVA	9.17 (5.73)			
**Eczema**	9.83 (5.82)	0.578 (-0.446 – 1.603)	0.622 (-0.400 – 1.644)	0.427 (-0.603 – 1.458)
Ref: No eczema	9.26 (5.77)			
**Migraine**	9.55 (5.34)	0.284 (-0.586– 1.154)	0.523 (-0.350– 1.397)	0.527 (-0.346 – 1.400)
Ref: No migraine	9.26 (5.80)			
**Ischaemic heart disease**	11.84 (5.95)	2.649 (1.560 – 3.738)***	2.093 (0.987 – 3.200)***	2.204 (1.098 – 3.309)**
Ref: No ischaemic heart disease	9.19 (5.75)			
**COPD**	11.89 (5.99)	2.711 (1.679 – 3.743)***	2.298 (1.248 – 3.348)***	2.128 (1.077 – 3.179)***
Ref: No COPD	9.18 (5.74)			
**Number of comorbidities**		0.723 (0.566 – 0.879)***	0.661 (0.494 – 0.828)***	0.642 (0.473 – 0.811) ***
**1 or more comorbidities**	10.30 (5.91)	1.740 (1.343 –2.138)***	1.559 (1.143 – 1.974)***	1.457 (1.031 – 1.883)***
Ref: No comorbidities	8.56 (5.57)			
**2 or more comorbidities**	10.89 (5.99)	2.013 (1.524 – 2.503)***	1.797 (1.280 – 2.314)***	1.789 (1.267 – 2.311)***
Ref: Less than 1 comorbidities	8.88 (5.64)			

Supplemental Figure 1. Distribution of all conditions in the SMI cohort by SMI diagnoses

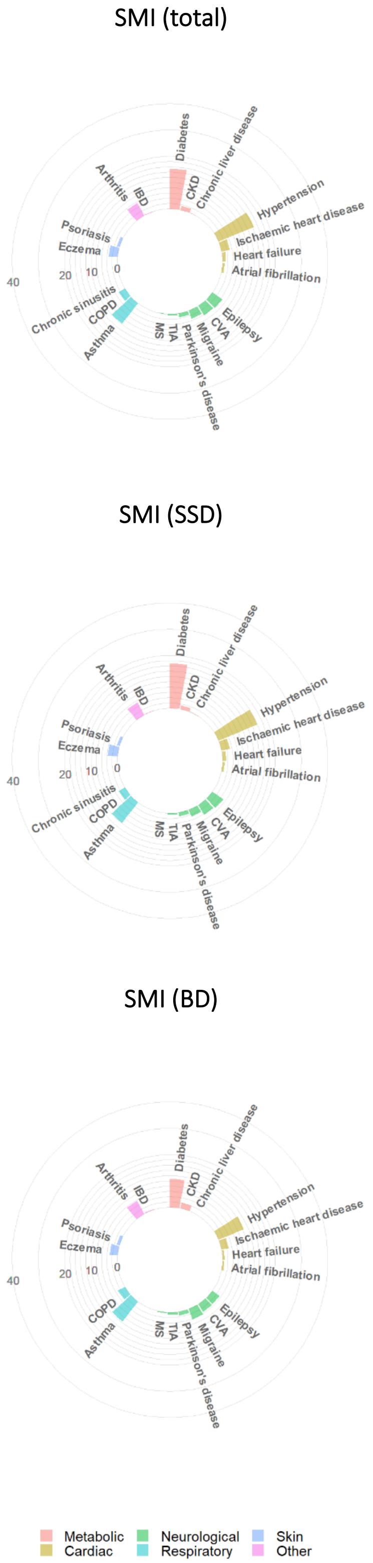

Supplemental Figure 2. Prevalence rates for age at SMI diagnoses per condition and comparison between individuals with BD and SSD.

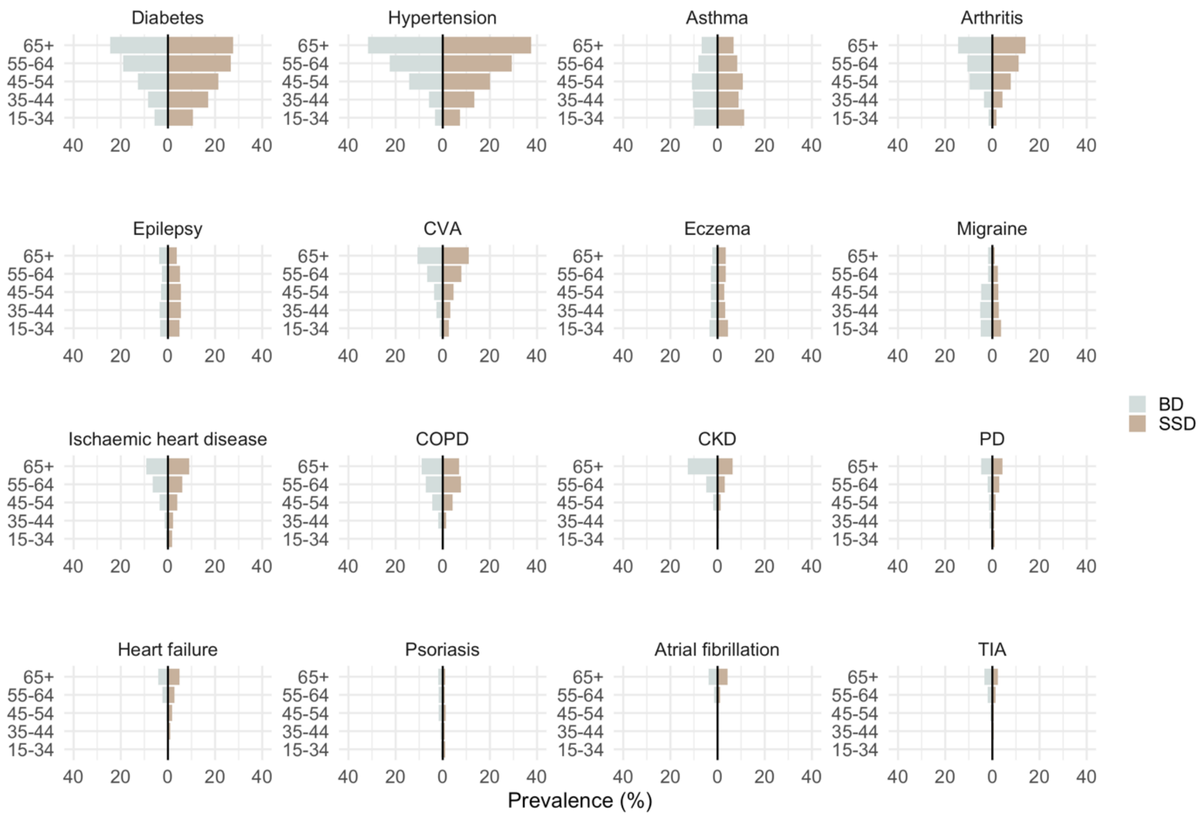

For peer review only

Appendix 1. SNOMED Container and Concept Level Groupings for physical health conditions included in this study.

Container Concept	Concepts
S-73211009 - Diabetes mellitus (disorder)	S-44054006 - Diabetes mellitus type 2 (disorder)<br>S-46635009 - Diabetes mellitus type 1 (disorder)<br>S-422088007 - Disorder of nervous system co-occurrent and due to diabetes mellitus (disorder)<br>S-25093002 - Disorder of eye co-occurrent and due to diabetes mellitus (disorder)<br>S-73211009 - Diabetes mellitus (disorder)
S-84114007 - Heart failure (disorder)	S-128404006 - Right heart failure (disorder)<br>S-48447003 - Chronic heart failure (disorder)<br>S-56675007 - Acute heart failure (disorder)<br>S-85232009 - Left heart failure (disorder)<br>S-42343007 - Congestive heart failure (disorder)<br>S-84114007 - Heart failure (disorder)
S-414545008 - Ischemic heart disease (disorder)	S-413439005 - Acute ischemic heart disease (disorder)<br>S-413838009 - Chronic ischemic heart disease (disorder)<br>S-194828000 - Angina (disorder)<br>S-22298006 - Myocardial infarction (disorder)<br>S-414545008 - Ischemic heart disease (disorder)
S-38341003 - Hypertensive disorder, systemic arterial (disorder)	S-31992008 - Secondary hypertension (disorder)<br>S-48146000 - Diastolic hypertension (disorder)<br>S-56218007 - Systolic hypertension (disorder)<br>S-59621000 - Essential hypertension (disorder)<br>S-38341003 - Hypertensive disorder, systemic arterial (disorder)
S-13645005 - Chronic obstructive lung disease (disorder)	S-195951007 - Acute exacerbation of chronic obstructive airways disease (disorder)<br>S-87433001 - Pulmonary emphysema (disorder)<br>S-13645005 - Chronic obstructive lung disease (disorder)
S-195967001 - Asthma (disorder)	S-195967001 - Asthma (disorder)
S-709044004 - Chronic kidney disease (disorder)	S-723190009 - Chronic renal insufficiency (disorder)<br>S-709044004 - Chronic kidney disease (disorder)

For peer review only

S-230690007 - Cerebrovascular accident (disorder)	S-25133001 - Completed stroke (disorder)<br>S-371040005 - Thrombotic stroke (disorder)<br>S-371041009 - Embolic stroke (disorder)<br>S-413102000 - Infarction of basal ganglia (disorder)<br>S-422504002 - Ischemic stroke (disorder)<br>S-723082006 - Silent cerebral infarct (disorder)<br>S-1078001000000105 - Haemorrhagic stroke (disorder)<br>S-230690007 - Cerebrovascular accident (disorder)
S-266257000 - Transient ischemic attack (disorder)	S-266257000 - Transient ischemic attack (disorder)
S-49049000 - Parkinson's disease (disorder)	S-49049000 - Parkinson's disease (disorder)
S-24700007 - Multiple sclerosis (disorder)	S-24700007 - Multiple sclerosis (disorder)
S-84757009 - Epilepsy (disorder)	S-352818000 - Tonic-clonic epilepsy (disorder)<br>S-19598007 - Generalized epilepsy (disorder)<br>S-230456007 - Status epilepticus (disorder)<br>S-509341000000107 - Petit-mal epilepsy (disorder)<br>S-84757009 - Epilepsy (disorder)
S-37796009 - Migraine (disorder)	S-37796009 - Migraine (disorder)<br>S-4473006 - Migraine with aura (disorder)<br>S-56097005 - Migraine without aura (disorder)
S-53741008 - Coronary arteriosclerosis (disorder)	S-810681000000101 - Coronary microvascular disease (disorder)<br><br>S-53741008 - Coronary arteriosclerosis (disorder)
S-49436004 - Atrial fibrillation (disorder)	S-49436004 - Atrial fibrillation (disorder)
S-40055000 - Chronic sinusitis (disorder)	S-40055000 - Chronic sinusitis (disorder)
S-24526004 - Inflammatory bowel disease (disorder)	S-24526004 - Inflammatory bowel disease (disorder)<br>S-397173003 - Crohn's disease of intestine (disorder)<br>S-64766004 - Ulcerative colitis (disorder)
S-328383001 - Chronic liver disease (disorder)	S-328383001 - Chronic liver disease (disorder)<br>S-76783007 - Chronic hepatitis (disorder)<br>S-79720007 - Chronic nonalcoholic liver disease (disorder)

For peer review only

	S-713181003 - Chronic alcoholic liver disease (disorder)
S-9014002 - Psoriasis (disorder)	S-9014002 - Psoriasis (disorder)
S-43116000 - Eczema (disorder)	S-43116000 - Eczema (disorder)
S-3723001 - Arthritis (disorder)	S-69896004 - Rheumatoid arthritis (disorder)<br>S-399112009 - Seronegative arthritis (disorder)<br>S-35908007 - Chronic arthritis (disorder)<br>S-11939005 - Acute arthritis (disorder)<br>S-3723001 - Arthritis (disorder)

For peer review only

STROBE Statement—checklist of items that should be included in reports of observational studies

	Item No	Recommendation	Page No
**Title and abstract**	1	(*a*) Indicate the study's design with a commonly used term in the title or the abstract	1
		(*b*) Provide in the abstract an informative and balanced summary of what was done and what was found	2
**Introduction**			
Background/rationale	2	Explain the scientific background and rationale for the investigation being reported	4,5
Objectives	3	State specific objectives, including any prespecified hypotheses	5
**Methods**			
Study design	4	Present key elements of study design early in the paper	5,6
Setting	5	Describe the setting, locations, and relevant dates, including periods of recruitment, exposure, follow-up, and data collection	5,6
Participants	6	(*a*) *Cohort study*—Give the eligibility criteria, and the sources and methods of selection of participants. Describe methods of follow-up<br>*Case-control study*—Give the eligibility criteria, and the sources and methods of case ascertainment and control selection. Give the rationale for the choice of cases and controls<br>*Cross-sectional study*—Give the eligibility criteria, and the sources and methods of selection of participants	5
		(*b*) *Cohort study*—For matched studies, give matching criteria and number of exposed and unexposed<br>*Case-control study*—For matched studies, give matching criteria and the number of controls per case	
Variables	7	Clearly define all outcomes, exposures, predictors, potential confounders, and effect modifiers. Give diagnostic criteria, if applicable	6-8
Data sources/ measurement	8*	For each variable of interest, give sources of data and details of methods of assessment (measurement). Describe comparability of assessment methods if there is more than one group	*6-8*
Bias	9	Describe any efforts to address potential sources of bias	6-9
Study size	10	Explain how the study size was arrived at	5,6
Quantitative variables	11	Explain how quantitative variables were handled in the analyses. If applicable, describe which groupings were chosen and why	6-8
Statistical methods	12	(*a*) Describe all statistical methods, including those used to control for confounding	8,9
		(*b*) Describe any methods used to examine subgroups and interactions	8,9
		(*c*) Explain how missing data were addressed	8,9
		(*d*) *Cohort study*—If applicable, explain how loss to follow-up was addressed<br>*Case-control study*—If applicable, explain how matching of cases and controls was addressed<br>*Cross-sectional study*—If applicable, describe analytical methods taking account of sampling strategy	8,9
		(*e*) Describe any sensitivity analyses	8,9

Continued on next page

For peer review only - http://bmjopen.bmj.com/site/about/guidelines.xhtml

For peer review only

**Results**			
Participants	13*	(a) Report numbers of individuals at each stage of study—eg numbers potentially eligible, examined for eligibility, confirmed eligible, included in the study, completing follow-up, and analysed	10
		(b) Give reasons for non-participation at each stage	
		(c) Consider use of a flow diagram	
Descriptive data	14*	(a) Give characteristics of study participants (eg demographic, clinical, social) and information on exposures and potential confounders	10
		(b) Indicate number of participants with missing data for each variable of interest	
		(c) *Cohort study*—Summarise follow-up time (eg, average and total amount)	
Outcome data	15*	*Cohort study*—Report numbers of outcome events or summary measures over time	
		Case-control study—Report numbers in each exposure category, or summary measures of exposure	
		Cross-sectional study—Report numbers of outcome events or summary measures	*9-13*
Main results	16	(*a*) Give unadjusted estimates and, if applicable, confounder-adjusted estimates and their precision (eg, 95% confidence interval). Make clear which confounders were adjusted for and why they were included	9-13
		(*b*) Report category boundaries when continuous variables were categorized	
		(*c*) If relevant, consider translating estimates of relative risk into absolute risk for a meaningful time period	
Other analyses	17	Report other analyses done—eg analyses of subgroups and interactions, and sensitivity analyses	Supp
**Discussion**			
Key results	18	Summarise key results with reference to study objectives	13,14
Limitations	19	Discuss limitations of the study, taking into account sources of potential bias or imprecision. Discuss both direction and magnitude of any potential bias	16
Interpretation	20	Give a cautious overall interpretation of results considering objectives, limitations, multiplicity of analyses, results from similar studies, and other relevant evidence	13-17
Generalisability	21	Discuss the generalisability (external validity) of the study results	16,17
**Other information**			
Funding	22	Give the source of funding and the role of the funders for the present study and, if applicable, for the original study on which the present article is based	18

*Give information separately for cases and controls in case-control studies and, if applicable, for exposed and unexposed groups in cohort and cross-sectional studies.

Note: An Explanation and Elaboration article discusses each checklist item and gives methodological background and published examples of transparent reporting. The STROBE checklist is best used in conjunction with this article (freely available on the Web sites of PLoS Medicine at http://www.plosmedicine.org/, Annals of Internal Medicine at http://www.annals.org/, and Epidemiology at http://www.epidem.com/). Information on the STROBE Initiative is available at www.strobe-statement.org.

For peer review only - http://bmjopen.bmj.com/site/about/guidelines.xhtml
